# Functionalized in Triplicate: A Ring‐By‐Ring Approach to Tailored Prodiginine Derivatives for Site‐Specific Conjugation Through Click Chemistry

**DOI:** 10.1002/chem.202502066

**Published:** 2025-07-14

**Authors:** T. Moritz Weber, Jörg Pietruszka

**Affiliations:** ^1^ Institute of Bioorganic Chemistry Heinrich Heine University Düsseldorf and Bioeconomy Science Centre (BioSC), im Forschungszentrum Jülich Geb. 15.8 52428 Jülich Germany; ^2^ Institute of Bio‐ and Geosciences (IBG‐1: Biotechnology) Forschungszentrum Jülich Wilhelm‐Johnen‐Straße 52428 Jülich Germany

**Keywords:** alkaloids, click chemistry, CuAAC, prodigiosin, pyrrole

## Abstract

The tripyrrole prototype alkaloid prodigiosin and members of the prodigiosin family are structurally diverse bacterial secondary metabolites. The privileged scaffold accounts for versatile biological activities, for example, antimicrobial, antitumoral, and immunosuppressive. Its Lewis‐basic lipophilic tripyrrole core and the aliphatic side chains allow for passive membrane diffusion, thereby trespassing the natural permeability barrier. However, diffusion‐controlled uptake is accompanied by low target specificity, hampering the development of tailored prodigiosin therapeutics and their selective delivery to target sites. To address this downside, this work focuses on providing the chemical methodology to synthesize prodiginines that are amenable to click chemistry on each of the three pyrrole moieties and facilitate the development of prodigiosin conjugates. Installing reactive azides and maleimides in the A‐, B‐, and C‐ring of the cytotoxic scaffold enables further functionalization per azide‐alkyne cycloaddition (CuAAC and SPAAC) and thiol‐maleimide addition, giving rise to protein‐conjugable prodiginines for the first time. The presented synthetic routes comprise yields of 3.24.7% over 1215 steps and grant access to valuable synthetic elements for expanding the toolbox of click chemistry to other pyrrole‐, pyrrolidinone‐, and tetramic acid‐containing natural products. Together, the devised methodology for prodiginine derivatization will collectively advance the development of alkaloid‐based conjugate therapeutics, eligible for target‐selective delivery.

## Graphical abstract

Prodigiosin is a potent cytotoxic alkaloid but lacks target selectivity. We advance functionalization of all three pyrrole rings of prodigiosin to enable site‐specific conjugation via click chemistry. The presented azide‐ and maleimide‐functionalized prodiginines are suitable for generating covalent prodiginine conjugates and attaching auspicious vectors for targeted drug delivery. The developed methodology promotes the expansion of conjugable pyrrole‐based building blocks and natural products in the future.

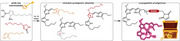

## 1 Introduction

**Scheme 1:**
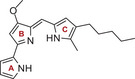
The cytotoxic tripyrrole alkaloid prodigiosin (**1**).

Conjugates of potential drugs and bioactive vector molecules provide expedient opportunities for target‐specific delivery of active agents.^[^ ^1^, ^2^ ^]^ So far, antibodies,^[^ ^3^, ^4^ ^]^ proteins, and peptides,^[^ ^2^, ^5^ ^]^ lipids,^[^ ^6^, ^7^ ^]^ and biological polymers just represent a small portion of carrier molecules,^[^ ^8^, ^9^, ^10^ ^]^ which have been functionalized to cotransport pharmacophores to fulfill their function as drug or regulatory element at a specific target site and circumnavigate off‐target effects.^[^ ^4^, ^11^, ^12^ ^]^ In this regard, Nature plays a vital role as a source of natural products and privileged scaffolds and has inspired chemists ever since to develop new drugs and drug candidates.^[^ ^13^, ^14^ ^]^ One prominent natural product from the class of alkaloids is the tripyrrolic red pigment prodigiosin (**1**, Scheme 1), the prototype compound of the prodigiosin family and a true Jack of all trades regarding biological activities.^[^ ^15^, ^16^, ^17^ ^]^

Numerous structure‐activity relationship (SAR) studies with natural and nonnatural derivatives of prodigiosin unraveled its underlying modes of action.^[^ ^18^, ^19^, ^20^ ^]^ To date, anticancer,^[^ ^21^, ^22^, ^23^ ^]^ antimicrobial,^[^ ^24^, ^25^, ^26^ ^]^ antifungal,^[^ ^27^ ^]^ antiprotozoal,^[^ ^28^ ^]^ and immunosuppressive properties have been ascribed to molecules akin to prodigiosin,^[^ ^29^ ^]^ illustrating the high interest in transferring the alkaloid into drug development.^[^ ^30^ ^]^ Many of these activities are observable on a low nanomolar concentration scale,^[^ ^21^, ^31^, ^32^ ^]^ which is most likely a result of superimposing effects. The protonated tripyrrolic scaffold was shown to form a tight lipophilic complex with small inorganic counterions (Cl^−^ and HCO_3_^−^), being able to diffuse passively across membranes and thereby uncoupling ion gradients by acting as an ionophore.^[^ ^33^ ^]^ Furthermore, prodigiosin can trap copper ions and induce DNA double‐strand breaks, causing DNA damage and cellular stress.^[^ ^34^, ^35^ ^]^ Anticancer activities against malignant tumor cell lines and the observed immunosuppressive and antiproliferative effects were partially attributed to the inhibition of several kinases in signal transduction and the ability to induce apoptosis.^[^ ^23^, ^36^, ^37^, ^38^ ^]^ Concurrently, the magnitude of biological activities exemplifies the downside of the alkaloid as a drug candidate: an apparent lack of target specificity and selectivity.^[^ ^19^, ^20^, ^39^ ^]^

Aiming at an increased target specificity and enhanced local effects of prodigiosin treatment, two unlike approaches have been pursued to attach the potent alkaloid to specificity‐mediating drug carriers: coating and encapsulation (noncovalent), and conjugation (covalent).^[^ ^39^, ^40^, ^41^, ^42^ ^]^ Generally, the noncovalent approaches are more straightforward, as they allow the use of the original drug (*e.g*., prodigiosin) and supersede chemical functionalization of the drug. However, although the noncovalent delivery approach has already enabled a continuous release of prodigiosin from carrier materials, such as microporous chitosan particles and peptide‐coated poly‐l‐lysine dendrigrafts, and increased the drug's antitumor activity in comparison to prodigiosin itself,^[^ ^40^, ^41^ ^]^ the exploitation of microscopic solid particles for drug transport prohibits a selective intracellular delivery across the cell membrane. For this purpose, covalent small‐molecule conjugates between drugs and target specificity‐mediating molecules have been developed, enabling active cellular uptake of the conjugated drug (*e.g*., antibody‐drug conjugates). In 2013, Thompson's group launched the first generation of covalent prodigiosin conjugates, focusing on exclusive chemical functionalization of prodigiosin's C‐ring and conjugation to small‐molecule carriers. By conjugating prodiginines to porphyrin, estrone, and 4‐hydroxytamoxifen, the researchers attempted to selectively target estrogen receptor‐positive breast cancer and thereby provide a strategy to overcome prodigiosin's lack of specificity.^[^ ^42^, ^43^ ^]^ While the first two generations of prodigiosin conjugates were based on an acid‐labile ester linkage with limited stability and an increased risk of premature drug release, later generations augmented the coupling chemistry to more stable amide and ether bonds, as well as the formation of nonhydrolyzable triazole units by means of copper‐catalyzed azide‐alkyne cycloaddition reactions (CuAAC).^[^ ^39^, ^42^, ^43^, ^44^ ^]^ In particular, the introduced azide‐substituted prodiginines bear great potential, as they could be coupled fairly easily in vitro or even in vivo with any alkyne in an azide‐alkyne cycloaddition via “click chemistry”,^[^ ^45^, ^46^, ^47^ ^]^ promoting target‐specific delivery of prodiginines for tailored applications. Adhering to the latter approach, this publication focuses on the systematic click‐compatible functionalization of all three pyrrole moieties of prodigiosin per organic total synthesis, facilitating the manufacturing of prodigiosin‐protein conjugates and thereby advancing site‐selective applications of prodigiosin and other pyrrole alkaloids.

## 2 Results and Discussion

**Scheme 2:**
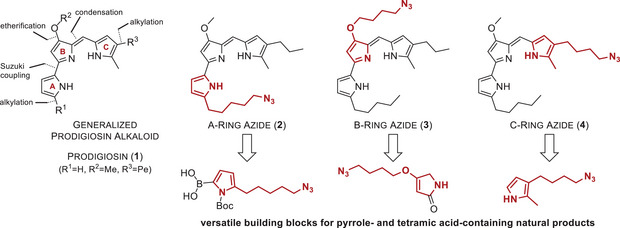
The retrosynthetic analysis of the prodiginine scaffold (shown as free base) uncovers essential synthetic equivalents for transferring organic azides into the natural product family.

Aiming to synthesize (bio)conjugable prodigiosin molecules through scalable chemical synthesis from scratch, meant to be used in targeted applications, we chose to implement the reliable azide‐alkyne cycloaddition (AAC) as a synthetic bridge between prodigiosin‐type alkaloids and potential linker or target molecules.^[^ ^48^, ^49^ ^]^ This type of click chemistry exploits the [3 + 2] cycloaddition between an azide‐ and an alkyne‐functionalized molecule to form the nonnatural, stable, and nonhydrolyzable 1,2,3‐triazole ring. Furthermore, the [3 + 2] cycloaddition reaction can be either performed in a copper‐catalyzed (CuAAC) or strain‐promoted (SPAAC) fashion, and its modular mode of operation allows for a simple exchange of conjugation partners.^[^ ^50^, ^51^ ^]^ Hence, we desired to produce azide‐functionalized prodigiosin derivatives for (bio)conjugation, carrying alkyl azide moieties in the A‐ **2**, B‐ **3**, or C‐ring **4** in positions that are known to appear functionalized in natural prodiginine derivatives, and are eligible for both CuAAC and SPAAC click chemistry (Scheme 2).

### 2.1 Synthesis of Prodigiosin A‐Ring Azide

Prodigiosin's pyrrolic A‐ring is essential for anticancer activity and oxidative DNA cleavage, and its functionalization with aliphatic substituents in the nitrogen‐adjacent 5‐position was recently shown to modulate the antitumoral activity against cisplatin‐resistant bladder cancer drastically.^[^ ^21^, ^52^, ^53^ ^]^ Yet, natural A‐ring derivatives of prodigiosin have been rarely found in Nature. The only two distinct examples that have been described in the literature, cyclononylprodigiosin and 2‐(*p*‐hydroxybenzyl)prodigiosin,^[^ ^54^, ^55^ ^]^ also bear substitutions in the 5‐position.^[^ ^54^, ^56^ ^]^ Based on these findings, we attempted to synthesize prodigiosin A‐ring azide **2**, harboring the alkyl azide moiety in the 5‐position of the A‐ring pyrrole (cf. Scheme 2).

**Scheme 3:**
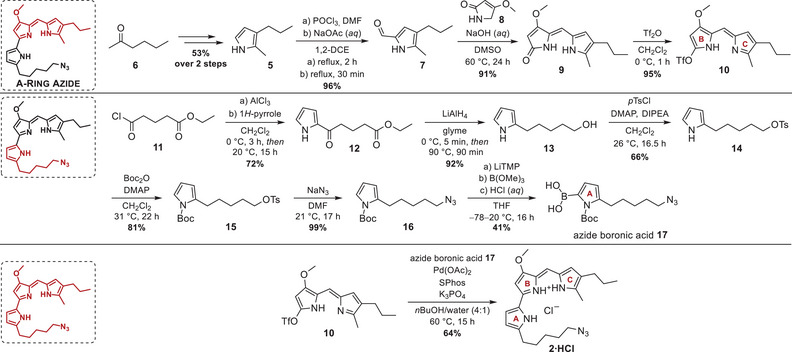
Synthesis scheme of prodigiosin A‐ring azide **2·HCl**. Omitted synthetic milestones from the precursor synthesis of the azide boronic acid **17** can be found in the Supporting Information.

Following Fürstner's synthetic approach, which commences with the synthesis of the B + C‐ring dipyrrinone and employs a late‐stage oxidative cross‐coupling for the A‐ring attachment (cf. Scheme 2),^[^ ^57^ ^]^ the first steps en route to the outlined prodigiosin A‐ring azide concentrated on generating the future alkyl‐substituted C‐ring precursor according to a short reaction sequence, previously reported by Weber et al.^[^ ^21^ ^]^ Here, the short‐chain pyrrole **5** was selected, as prodiginine derivatives with short‐chain substitution on the C‐ring were previously shown to bear the highest inhibitory effects against sensitive Gram‐negative bacteria, and induce cytotoxicity in eukaryotic cells by causing pronounced accumulation of autophagosomes.^[^ ^58^, ^59^ ^]^ In detail, hexan‐2‐one (**6**) was converted with hydroxylamine hydrochloride and sodium acetate to the corresponding *E/Z* mixture of oxime **S1** in quantitative yield and then subjected to a Trofimov reaction to provide the monopyrrole **5** in a yield of 53% over two steps (Scheme S1, Supporting Information).^[^ ^21^, ^60^, ^61^ ^]^ A Vilsmeier–Haack formylation of pyrrole **5** with phosphoryl chloride and DMF in 1,2‐dichloroethane (1,2‐DCE) furnished the 2‐formylated pyrrole **7** in an excellent yield of 96% (Scheme 3).^[^ ^57^ ^]^ Condensation of carbaldehyde **7** with the commercially available tetramic acid **8** in DMSO with aqueous NaOH at 60 °C provided dipyrrinone **9** in a yield of 91%, and its subsequent triflation with triflic anhydride gave access to the triflated azafulvene **10** in a formidable yield of 95%.^[^ ^57^ ^]^ In the latter synthesis, using freshly opened Tf_2_O was inevitable to allow the reaction to proceed to complete conversion, thereby superseding the need for purification other than extractive workup. With the triflated B + C‐ring precursor **10** at hand, we next focused on synthesizing the azide‐substituted A‐ring precursor. Based on the intended Suzuki–Miyaura cross‐coupling reaction to form the tripyrrolic scaffold, the A‐ring azide must be introduced through a pyrrole‐2‐boronic acid species endowed with an alkyl azide moiety (cf. Scheme 2). Although unprotected pyrrole‐2‐boronic acids are highly reactive in transmetalation and common intermediates in prodigiosin chemistry,^[^ ^21^, ^52^, ^57^, ^62^, ^63^ ^]^ their synthesis and isolation (this holds in particular true for the 2‐heteroaryl boronic acids,^[^ ^64^, ^65^, ^66^ ^]^ “2‐pyridyl problem”) pose a significant challenge due to their inherent lability.^[^ ^57^, ^67^ ^]^

Aware of the upcoming challenges, synthesizing this azide‐substituted boronic acid was first attempted through a route comprising alkene pyrrole **S2** and subsequent functional group interconversions to be able to introduce the reactive azide before attempting the borylation. However, this route was dismissed due to the lack of stability of alkene pyrrole **S2** and its tedious purification (Scheme S2, Supporting Information).^[^ ^57^, ^68^, ^69^ ^]^ Alternatively, exploitation of the terminally chlorinated 2‐acylpyrrole **S3** was attempted. But again, this route was not fruitful, and reduction of the acyl group of the chlorinated acyl pyrrole **S3** to provide the chlorinated alkyl pyrrole **S4** led to the formation of inseparable elimination side products as well as undesired reduction of the terminal halide (Scheme S2, Table S1, Supporting Information). Ultimately, we aimed for a shortcut to circumvent the delicate alkene **S2** intermediate. The solution to this problem was found in the acylation of 1*H*‐pyrrole with the ester‐functionalized ethyl glutaryl chloride (**11**), which already harbored the hydroxy group needed for activation, just in another oxidation state (Scheme 3). The acylation reaction with AlCl_3_ yielded 72% of the 2‐functionalized acyl pyrrole **12**,^[^ ^70^ ^]^ which was then subjected to hydride reduction to reduce the acyl group and the ester in a single step.

Several metal hydrides in ethereal (THF, glyme, or 1,4‐dioxane) solvents or *i*PrOH were screened for their suitability to facilitate the concomitant reduction of ketone and ester to give access to terminally hydroxylated pyrrole **13** (Table S2, Supporting Information). The best results were obtained in glyme with LiAlH_4_ (5 eq.) at 80 °C, providing a 92:8 ratio of hydroxylated pyrrole **13** and elimination product **S5** under consideration of the essential cooling step in the early phase of the reaction. The large‐scale reduction of pyrrole ester **12** was performed on a 20 g scale, and despite the usage of large amounts of highly reactive LiAlH_4_ (18.4 g) and the expected formation of vast amounts of hydrogen gas and insoluble lithium salts, quenching according to Fieser's protocol facilitated easy removal of floating lithium salts.^[^ ^71^ ^]^ The hydroxylated pyrrole **13** was obtained as a very sticky and polar oil in an excellent yield of 92%. Activation of the terminal alcohol via tosylation with *p*TsCl, diisopropyl ethylamine (DIPEA), and catalytic 4‐(dimethylamino)pyridine (DMAP) gave 66% yield of pyrrole alcohol **14**. A subsequent Boc‐protection of tosylate **14** with Boc_2_O and DMAP in CH_2_Cl_2_ and the following tosyl substitution with NaN_3_ in DMF furnished the *N*‐protected tosylate **15** and organic azide **16** in a yield of 81% and 99%, respectively.

Lastly, the azide boronic acid **17** was synthesized by sequential *ortho*‐metalation with lithium 2,2,6,6‐tetramethylpiperidide (LiTMP) at −78 °C, quenching of the reactive species with B(OMe)_3_, and boronic ester hydrolysis with diluted aqueous HCl, and it was isolated as a yellow solid in a yield of 41% (for comments on isolation, see Supporting Information).^[^ ^57^, ^72^, ^73^ ^]^ To our knowledge, it is the first successful preparation and isolation of an azide‐substituted heteroaryl‐2‐boronic acid and one of the rarely met examples of azide‐containing boronic acid derivatives,^[^ ^74^, ^75^, ^76^, ^77^ ^]^ and this is for a good reason: The boronic acid **17** was extremely delicate. It decomposed at −20 °C under an argon atmosphere within less than 3 days and instantly decomposed when exposed to high vacuum. Here, we hypothesize that the flexible terminal azide could potentially coordinate to the strongly positively polarized boron atom of the boronic acid functional group of heteroaryl intermediate **17**, triggering an intramolecular rearrangement reaction. This assumption is in agreement with recent reports of Florentino et al., who synthesized 2‐alkyl‐2‐methylpyrrolidines through a novel intramolecular carboborylation of alkyl azides under the release of molecular nitrogen.^[^ ^78^, ^79^ ^]^ This proposed mechanism could also clarify why azide‐substituted boronic acid **17** underwent rapid degradation upon compression of the solid product under vacuum, namely through a facilitated shift of the equilibrium per removal of nitrogen gas under reduced pressure. Nonetheless, the successful preparation of an azide‐substituted pyrrole‐2‐boronic acid is a synthetic milestone toward clickable prodiginines and other pyrrole‐based natural products.

Having established scalable synthetic routes giving access to triflate **10** and pyrrole‐2‐boronic acid **17**, only the oxidative coupling reaction between the B + C‐ring and the A‐ring precursors remained missing to finally yield the prodigiosin A‐ring azide **2**. In the past, Pd(PPh_3_)_4_ was the first choice catalyst for Suzuki–Miyaura cross‐couplings and was first established by Fürstner et al. in prodigiosin, tambjamine, and marineosin chemistry.^[^ ^31^, ^32^, ^57^, ^73^ ^]^ However, the commercially available Pd(0) precatalyst requires elevated temperatures (>80 °C for OTf and Br) to shed the gratuitous PPh_3_ ligands and leverage the oxidative addition of Pd(PPh_3_)_2_ into the carbon‐halide bond in the first step of the catalytic cycle.^[^ ^80^, ^81^, ^82^ ^]^ However, given the inherent lability of boronic acid **17** and the thermal instability of organic azides and pyrrole‐2‐boronic acids in general,^[^ ^57^, ^83^, ^84^, ^85^ ^]^ we set out to screen for Pd ligands that would allow coupling at a decreased temperature of 60 °C in reasonable yields (Table S3, Supporting Information).

In the screening reaction, investigating the coupling efficiency of unsubstituted pyrrole‐2‐boronic acid **S6** and triflate **10**, Buchwald's SPhos ligand stood out from the tested phosphine ligands (for structures see Scheme S3, Supporting Information) and rendered a yield of 84% compared to 55% yield with PPh_3_ (Table S3, Supporting Information, for additional comments on grease contaminants see Chapter “*Identification and Prevention Strategies for Grease Contaminations*”, Table S4, Figures S1, S2, Supporting Information).

Thus, the optimized catalyst system with Pd(OAc)_2_ and a small excess of SPhos, combined with K_3_PO_4_ as inorganic base and a solvent system of *n*BuOH/water, were applied for all further cross‐coupling reactions. Despite the labile nature of azide‐substituted boronic acid **17**, the cross‐coupling with alkyl triflate **10** resulted in a concomitant deprotection of the Boc‐protected pyrrolic A‐ring nitrogen and lastly yielded 64% of the drafted prodigiosin A‐ring azide **2·HCl** (Scheme 3). In summary, the prodigiosin A‐ring azide **2·HCl** was synthesized over 12 steps in 4.0% yield (79% per step), marking the first prodiginine with a polar noncarbon substituent on an alkylated pyrrolic A‐ring. Together, the developed synthetic intermediates enable the introduction of numerous heteroatoms and reactive groups (─OH, ─OTs, ─N_3_) or derivatives thereof (*e.g*., primary amines) into the prodiginine A‐ring and may advance the availability of atypical prodiginine substitution patterns for bioactivity testing and conjugation to target specificity‐mediating vectors.

### 2.2 Synthesis of Prodigiosin B‐Ring Azide

All known natural alkaloids from the prodiginine family, but also closely related tambjamines and marineosin spiroaminals, carry an *O*‐methoxy group in the 4′‐position of the B‐ring. However, synthetic B‐ring derivatives of prodigiosin have been reported, carrying *O*‐aryl functionalization in the 4′‐position,^[^ ^32^, ^86^ ^]^ as well as 4′‐alkyl, 3′,4′‐alkyl, or even no substitution.^[^ ^31^, ^32^, ^87^, ^88^, ^89^, ^90^ ^]^ Multiple SAR studies underlined that the 4′‐alkoxy group of prodigiosin is crucial for maintaining the anticancer and immunosuppressive activity of the alkaloid.^[^ ^32^, ^90^, ^91^, ^92^ ^]^ Based on these data, we desired to synthesize prodigiosin B‐ring azide **3** (cf. Scheme 2) and therefore investigated suitable options for introducing a 4′‐*O*‐alkyl substitution in the B‐ring, allowing for terminal azide incorporation and mimicking the natural substitution of prodiginine alkaloids.

**Scheme 4:**
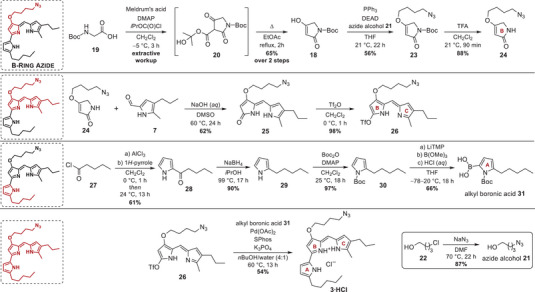
Synthesis scheme of prodigiosin B‐ring azide **3·HCl**.

In analogy to the unprotected B‐ring tetramic acid precursor **8** for the *O*‐methyl substitution pattern of prodigiosin, we used the *N‐*Boc‐protected tetramic acid **18** for further functionalization (Scheme 4). In the first step, *N*‐Boc glycine (**19**) was converted in a batch reaction to the mixed anhydride with isopropyl chloroformate in CH_2_Cl_2_. DMAP as a base and catalyst was applied to deprotonate Meldrum's acid to attack the anhydride on the sterically less hindered glycine flank. Intramolecular attack of the nitrogen and opening of the 6‐membered ring yielded the C‐acylated tetramic acid intermediate **20** in the keto form. Heating in EtOAc triggered the concerted elimination of acetone and decarboxylation to culminate in the stable Boc‐protected tetramic acid **18** in the enol form.^[^ ^93^, ^94^, ^95^, ^96^ ^]^ Based on the literature, we first attempted *O*‐functionalization of the tetramic acid **18** enol via *O*‐tosylation and subsequent etherification of tosylate **S7** under DABCO‐catalysis. However, no reaction was observed between an aliphatic alcohol lacking activating substituent effects and tetramic acid **18** toward the *O*‐alkylated intermediate **S8** (Scheme S4, Supporting Information). Instead, a procedure by Fort et al. for the direct *O*‐alkylation of tetronic acid was adapted, using the Mitsunobu etherification in conjunction with strong dehydrating azodicarboxylate reagents.^[^ ^93^, ^94^ ^]^ The Mitsunobu displacement with diethyl azodicarboxylate (DEAD) and PPh_3_ in THF between enol **18** and the azide alcohol **21**,^[^ ^97^ ^]^ which had been synthesized in a one‐step conversion from 4‐chloro‐1‐butanol (**22**),^[^ ^98^ ^]^ provided the azide‐functionalized Boc‐protected tetramic acid **23**. Even though tetramic acid **23** was difficult to purify from other reaction side products through normal‐phase chromatography, it was finally isolated on a multigram scale in a yield of 56%. Standard deprotection of tetramic acid **23** with trifluoroacetic acid (TFA) in CH_2_Cl_2_ provided the free base of azide‐functionalized *O*‐alkylated tetramic acid **24** in a reasonable yield of 88% as a direct B‐ring precursor for the introduction of the azide moiety into the tripyrrole structure.

The synthesis toward the prodigiosin B‐ring azide **3** proceeded with the B + C‐ring coupling via the earlier introduced base‐catalyzed condensation reaction between tetramic acid derivative **24** and the carbaldehyde **7** in a moderate yield of 62%. This was followed by triflation of dipyrrinone **25** in near‐quantitative yield with triflic anhydride in CH_2_Cl_2_, yielding the azide‐bearing triflate **26** as one of two precursors needed for the final Suzuki–Miyaura cross‐coupling reaction (Scheme 4).

The second precursor design was again based on a pyrrole‐2‐boronic acid, but this time endowed with an additional alkylation in the 5‐position of the future A‐ring. This pentyl chain alkylation was introduced via initial acylation of 1*H*‐pyrrole with pentanoyl chloride **27**,^[^ ^70^ ^]^ followed by a reduction of the acylation product **28** with NaBH_4_ in *i*PrOH,^[^ ^99^ ^]^ and an *N*‐Boc‐protection of the alkylated pyrrole **29** with Boc_2_O and catalytic DMAP.^[^ ^57^ ^]^ Finally, the Boc‐protected 2‐alkyl pyrrole **30**, was employed for generating alkyl boronic acid **31** through *ortho*‐metalation (Scheme 4). Following the approved procedure from the earlier preparation of azide boronic acid **17**, the alkylated boronic acid **31** was isolated as a relatively stable light‐yellow solid in a good yield of 66%. In the last step, the optimized SPhos‐assisted Pd‐catalyzed cross‐coupling reaction between triflate **26** and alkyl boronic acid **31** facilitated the isolation of prodigiosin B‐ring azide **3·HCl** in a yield of 54% (Scheme 4). Considering all performed synthetic transformations, the prodigiosin B‐ring azide **3·HCl** was synthesized in a yield of 3.2% over 12 steps (78% per step). Together, the devised synthetic approach allows for introducing numerous polar heteroatoms and reactive groups into the prodiginine B‐ring. Although prodigiosin B‐ring azide **3** is currently the only prodiginine carrying a polar *O*‐alkylated substituent in the 4′‐position, the drafted synthesis vastly expands the array of fabricable derivatives and may hence contribute to broadening the landscape of synthetic prodiginines in the future.

### 2.3 Synthesis of Prodigiosin C‐Ring Azide

From all natural prodiginines isolated and structurally characterized to date, the C‐ring shows by far the highest variance in substitution patterns. Together with the relatively low synthetic burden of derivatizing the C‐ring pyrrole, those prodiginines with a nonnatural C‐ring substitution are also the predominant group among all prodiginines of synthetic provenance. This is also the reason why all conjugable prodiginines, including the two reported azide‐functionalized prodiginine derivatives, have focused on C‐ring conjugation with a nonnatural 4‐alkyl‐3,5‐dimethyl substitution pattern.^[^ ^39^, ^42^, ^43^, ^44^ ^]^ To complement the existing clickable derivatives of prodigiosin **1** with a derivative that mimics the natural 5‐methyl‐4‐pentyl substitution of the natural product, we aimed to synthesize prodigiosin C‐ring azide **4** (cf. Scheme 2).

Starting with the synthesis of the C‐ring precursor, endowed with the reactive azide moiety, two independent routes, converging at the terminal alcohol **32**, were explored. A sequence involving a Trofimov pyrrole synthesis and hydroboration/oxidation of alkene pyrrole **S9** to terminal alcohol **32** revealed that the laborious purification of alkene pyrrole **S9** was a limiting factor for yield, resource efficiency, and reaction upscaling (Scheme S5, Supporting Information). Furthermore, the high cost of the alkene‐functionalized carboxylic acid **S10** as a starting material was diagnosed as a severe disadvantage.

**Scheme 5:**
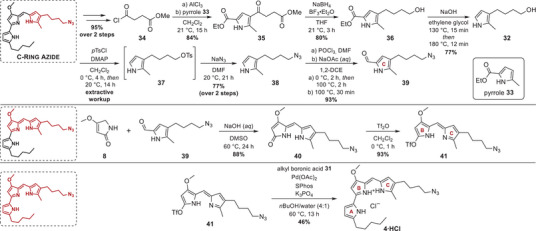
Synthesis scheme of prodigiosin C‐ring azide **4·HCl**.

Alternatively, we sought a practicable method to circumvent the alkene **S9** and developed a concept for functionalizing an existing pyrrole scaffold, rather than forming the heterocycle *de novo* through synthesis. For this purpose, the methyl‐substituted pyrrole ester (**33**), available from commercial suppliers in large quantities, can be functionalized regioselectively in the 4‐position by Lewis acid‐catalyzed acylation with monomethyl succinyl chloride **34** (here synthesized in two‐step steps from succinic anhydride **S11**, see Scheme S6, Supporting Information), as the ethyl ester sterically hinders the adjacent 3‐position (Scheme 5).^[^ ^56^ ^]^

Similar to the one‐step LiAlH_4_ reduction of pyrrole **12** under reflux conditions, reduction using NaBH_4_ and BF_3_ etherate in THF achieved a one‐step reduction of the acyl group and the methyl ester of pyrrole **35**, providing ethyl pyrrole‐2‐carboxylate **36** with the terminal primary hydroxy group in a yield of 80%.^[^ ^100^ ^]^ This reaction approach using diborane as a reactive species has significant advantages over the more reactive LiAlH_4_, as NaBH_4_/BF_3_·Et_2_O operates at room temperature, offering a milder alternative to highly reactive aluminium hydrides. However, due to the diminished reducing power of the formed diborane, the amide‐like conjugated carbonyl at the 2‐position of the heteroaromatic ring of intermediate **36** remained intact, necessitating further saponification and decarboxylation to yield pyrrolic alcohol **32** as a precursor to the C‐ring azide. For decarboxylation, serious effort was invested to forestall the competing polymerization, which was observed under literature conditions, namely heating with NaOH in ethylene glycol at 160 °C for 1 hour.^[^ ^56^ ^]^ Time‐resolved TLC analyses on small‐scale reactions revealed complete ester hydrolysis at 130 °C after 10 minutes (no decarboxylation) and thorough decarboxylation at 170 °C after 8 minutes. Despite complete conversion, further heating only caused rapid degradation. Since all optimizations were conducted on a small scale, the intrinsic obstacle of a preparative reaction would need to account for slower heat transfer in large reaction vessels. Thus, the reaction time was prolonged to 15 minutes at 130 °C for ester hydrolysis, followed by the decarboxylation step at 180 °C internal temperature for 12 minutes. Based on this streamlined procedure, the hydroxy‐functionalized pyrrole **32** was obtained in an improved yield of 77%, compared to only 41% yield under literature conditions (*vide supra*). As this reaction sequence was carried out on a multigram scale, providing 49% of the pyrrole alcohol **32** over three steps, this latter route is more scalable than the alkene route for three reasons: a) Chloride **34** and the pyrrole **33** are both commercially available and considerably cheaper than the 5‐hexenoic acid (**S10**) as starting material for the hydroboration route (cf. Scheme S5, Supporting Information). b) Target diversification can be easily accomplished by using differently substituted acid chlorides to functionalize pyrrole **33** in the first place, as, for example, performed in the work of Kancharla et al.^[^ ^56^ ^]^ c) The time‐consuming purification of alkene pyrrole **S9** in the hydroboration route is a clear disadvantage when aiming for analytically pure compounds (NMR grade).

With satisfactory access to intermediate **32**, further efforts were turned to getting hands on the azide‐functionalized C‐ring precursor without resorting to an additional protecting group strategy for the pyrrolic nitrogen. Transformation of the terminal hydroxy group into a tosylate leaving group was first attempted with tosyl chloride, as previously performed in the synthesis of the A‐ring azide **2·HCl** (cf. Scheme 3). A base screening in CH_2_Cl_2_ with pyridine, DMAP, or 1,8‐diazabicyclo(5.4.0)undec‐7‐ene (DBU) suggested only pyridine and DMAP as suitable bases for efficient conversion of pyrrole **32** into tosylate **37**, but only DMAP rendered complete conversion. However, every attempt at product isolation, either by aqueous workup or normal‐phase column chromatography, led to thickening and allegedly polymerization upon thorough solvent removal or contact with silica. Spiking the diluted reaction extract with DMF and further removal of CH_2_Cl_2_ under reduced pressure addressed this problem without causing decomposition. The unstable tosylate **37** in DMF was then supplemented with NaN_3_, and the organic azide **38** was isolated in 77% over two consecutive steps. A Vilsmeier–Haack formylation finally provided the azide‐functionalized carbaldehyde **39** in a very good yield of 93%.^[^ ^57^ ^]^ Next, the B + C‐ring dipyrrinone structure was built by the base‐catalyzed condensation reaction between the azide‐substituted C‐ring precursor carbaldehyde **39** and the tetramic acid building block **8** (Scheme 5). Both the condensation reaction and the follow‐up triflation proceeded with high yields and without the need for chromatographic purification, yielding 88% of the dipyrrinone intermediate **40** and 93% of the triflate **41** with azide‐modification on the future C‐ring. Ultimately, the earlier introduced alkyl boronic acid **31** was cross‐coupled with triflate **41** under the optimized reaction conditions in a moderate yield of 46% compared to the cross‐coupling reaction toward A‐ring and B‐ring azides, providing the prodigiosin C‐ring azide **4·HCl** in a yield of 4.6% over 15 consecutive steps (83% per step) (Scheme 5).

### 2.4 Functionalized Prodiginines Outline Future Applications of Prodiginine Conjugates

After streamlining and accomplishing the synthesis of the azide‐functionalized A‐, B‐, and C‐ring derivatives **2·HCl**, **3·HCl**, and **4·HCl** of prodigiosin, we set out to use CuAAC click chemistry for preparing protein‐conjugable prodiginines and small‐molecule conjugates of the tripyrrole alkaloid. Reliable and well‐established thiol‐maleimide chemistry was appointed as the foundation for protein conjugation to avoid directly labeling proteins with prodiginines via CuAAC and the risk of copper‐mediated protein oxidation.^[^ ^101^, ^102^, ^103^ ^]^ Therefore, the bifunctional alkyne‐maleimide linker **42** (for synthesis see Scheme S7, Supporting Information) was chosen as a suitable means to introduce the thiol‐reactive maleimide moiety into prodiginines via CuAAC.

**Scheme 6:**
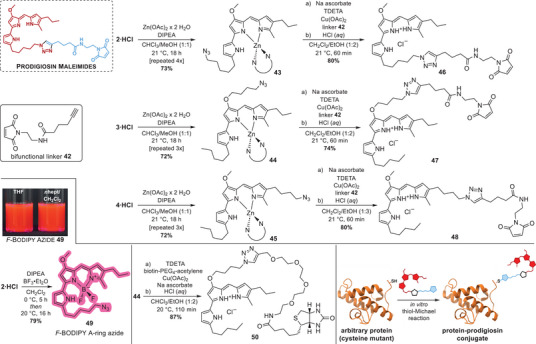
Click functionalization of prodigiosin azides and manufacturing of small‐molecule and protein conjugates. **Top**: Preparation and CuAAC‐functionalization of homo‐dimeric prodigiosin zinc complexes **43**, **44**, and **45** with the bifunctional alkyne‐maleimide linker **42** uncover the maleimide decorated A‐, B‐, and C‐ring prodiginines **46**, **47**, and **48**, respectively. **Bottom left**: Clickable prodigiosin *F*‐BODIPY A‐ring azide **49** with bright‐red fluorescence [here shown as an 80 µm solution in THF and *n*‐heptane/CH_2_Cl_2_ (99:1)]. **Bottom middle**: Prodigiosin‐d‐biotin conjugate facilitates selective binding to streptavidin, useful in pull‐down assays and target fishing approaches. **Bottom right**: Generic protein functionalization per thiol‐Michael addition with the novel prodiginine maleimides **46**, **47**, and **48**.

Lewis‐basic metal‐chelating heterocycles, such as prodigiosin, compete in CuAAC reactions with the alkyne in complexation of Cu(I) and Cu(II) species, as highlighted by self‐accelerated chelation‐assisted click reactions of *N*‐heterocycles.^[^ ^104^, ^105^ ^]^ To avoid this participation in copper chelation, the heterocycles must be protected prior to performing the click reaction.^[^ ^106^ ^]^ Furthermore, Marchal et al. previously reported on the decomposition of unprotected azide‐functionalized prodigiosin derivatives upon copper‐complexation in the dipyrrin structure.^[^ ^39^ ^]^ Hence, we exploited prodigiosin's strong metal‐chelation to prepare temporary homo‐dimeric zinc complexes of prodigiosin A‐, B‐, and C‐ring azides **2**, **3**, and **4**, respectively. By repeatedly adding Zn(OAc)_2_ and DIPEA to a solution of prodigiosin azides in CHCl_3_/MeOH, the three zinc complexes **43**, **44**, and **45** were isolated in reproducible yields of 72 −73% (Scheme 6).^[^ ^39^ ^]^

To date, the only CuAAC reactions performed with homo‐dimeric zinc complexes of prodiginine azides were performed with the hydrophobic tripodal copper ligand TBTA [tris(benzyltriazolylmethyl)amine].^[^ ^39^ ^]^ Although the CuAAC click chemistry itself is renowned for its reliability and typically provides excellent to near‐quantitative yields, the TBTA‐assisted CuAAC reactions allowed recovery of the triazole product only in moderate yields between 46% and 58%. Together with the opposing polarities of the hydrophobic prodiginines and the very polar bifunctional linker **42**, we opted for using our recently reported Cu(I)/Cu(II) ligand TDETA instead (for structure see Scheme S8, Supporting Information), which poses excellent solubility in a wide variety of protic and aprotic solvents, and features compatible reaction rate‐acceleration as TBTA for a broad scope of aromatic and aliphatic azides and alkynes.^[^ ^107^, ^108^ ^]^ As anticipated, the azide‐functionalized zinc complexes **43**, **44**, and **45** were fully converted with linker **42** to the corresponding triazole conjugates within 60 minutes, promoted by a catalytic system of Cu(I) and TDETA under reducing conditions. After hydrolysis of the zinc complexes with aqueous 1 m HCl and chromatographic purification, the maleimide‐functionalized click products were isolated in reasonable quantity. Here, the good yields of 80%, 74%, and 80% for the prodigiosin A‐, B‐, and C‐ring maleimides **46**, **47**, and **48**, respectively (Scheme 6, top), are rated as significant improvement in comparison to yields obtained with the widely applied TBTA ligand (vide supra).^[^ ^39^ ^]^ While the synthesized prodigiosin azides **2**, **3**, and **4** are the first holistic set of clickable prodiginines with clickable azide moieties installed in every of the three pyrrolic rings, the novel prodiginine‐maleimide conjugates **46**, **47**, and **48** are also the first of their kind and open up the new and unexplored field of protein‐conjugable prodiginines (for exemplary protein labelling see Chapter “*Protein‐Prodigiosin Conjugates*”, Figure S4, Table S5, Supporting Information; for absorption properties see Figures S5, S6, Table S6, Supporting Information). Reclaiming the extraordinarily high cytotoxicity of prodigiosin, coinciding with their lack of target selectivity, protein‐conjugable prodiginines could potentially overcome this limitation and provide an expedient means for targeted delivery through conjugation to target‐selective peptide or protein vectors (*e.g*., therapeutic monoclonal antibodies).

Aiming to streamline the application of CuAAC‐compatible prodiginines and CuAAC‐derived prodiginine conjugates in targeted therapies and target identification, we prepared two further examples of click‐related prodiginines that highlight the potential of our work. On the one hand, the clickable red‐fluorescent *F*‐BODIPY **49** with A‐ring azide substitution was synthesized from prodigiosin A‐ring azide **2·HCl** in a yield of 79% per trapping boron difluoride between the nitrogen atoms of the dipyrrin core (Scheme 6).^[^ ^109^ ^]^ The unique fluorescence properties of aryl‐ and alkyl‐azide‐harboring *F*‐BODIPYs have been proven beneficial in various imaging applications.^[^ ^109^, ^110^, ^111^ ^]^ However, in comparison to traditional *F*‐BODIPYs, the prodiginine BODIPY **49** is clickable and moreover characterized by a substantial red shift of the absorbance maximum (582594 nm) and a sharp fluorescence emission at 588603 nm (Figure S7, Supporting Information). BODIPYs are currently investigated as potent photosensitizers for photodynamic therapy, causing the generation of cell‐damaging singlet oxygen upon reaction between BODIPYs in the triplet state and ^3^O_2_.^[^ ^112^ ^]^ Combined with conjugation to vectors mediating a target‐selective delivery, conjugates of clickable prodigiosin *F*‐BODIPYs, such as *F*‐BODIPY A‐ring azide **49**, could hence be used for a concentrated release of cytotoxic reactive oxygen species (ROS) at the desired target site (*e.g*., cancerous tissue).

Another potential application of clickable prodiginines lies in identifying novel cellular targets of the alkaloid. Although several cellular targets have been identified over the last decades, it remains vague whether prodigiosin can additionally interact with other cellular structures (*e.g*., enzymes) and act as an inhibitor of regulatory metabolic processes other than those already known.^[^ ^113^ ^]^ One such molecule ideal for this kind of target fishing approach is d‐biotin. The d‐biotin moiety allows for high‐affinity binding to the proteins avidin and streptavidin,^[^ ^114^ ^]^ whose tight interaction is commonly used for pull‐down assays with streptavidin‐coated surfaces, such as agarose or magnetic beads.^[^ ^115^, ^116^, ^117^ ^]^ For this reason, the biotinylated prodigiosin derivative **50** was prepared through a CuAAC click reaction between the zinc complex of prodigiosin B‐ring azide **44** and the commercially available biotin‐PEG_4_‐acetylene (Scheme 6, bottom middle). Under the established CuAAC reaction conditions, using the tripodal ligand TDETA and reducing conditions, the biotinylated prodigiosin **50** was isolable in a good yield of 87%. Prodigiosin‐d‐biotin conjugates, such as the introduced derivative **50**, could be auxiliary in elucidating so far unknown interaction partners and targets of prodiginine alkaloids by coprecipitation of potential targets and biotinylated prodiginines on streptavidin‐coated surfaces.

## 3 Conclusion

Prodigiosin is a Jack of all trades and combines a variety of potent cytotoxic activities. However, the lack of target selectivity is a significant drawback of the privileged tripyrrole alkaloid, limiting therapeutic applications. Therefore, this work addressed the demand for chemical methodologies to expand the portfolio of synthetic prodiginine derivatives eligible for conjugation to target‐selective vectors and amenable to popular click chemistry. By identifying essential pyrrole and tetramic acid building blocks, we introduced reactive organic azides in the A‐, B‐, and C‐ring of the prodiginine scaffold through total synthesis. The corresponding prodigiosin azides were synthesized over 12 15 steps in yields of 3.2 4.7%, compatible with CuAAC and SPAAC click chemistry. The uncovered synthetic building blocks were synthesized on a multigram scale, proving the scalability of the reported procedures. Finally, copper‐catalyzed click chemistry was exploited to couple the prodigiosin azides to a bifunctional alkyne‐maleimide linker and a d‐biotin moiety, allowing for protein conjugation, surface immobilization, and target fishing. Collectively, this study emphasizes the vast potential of click chemistry in natural product derivatization. The devised methodologies will guide the future design of clickable prodiginines, tambjamines, marineosins, or other pyrrole‐ and tetramic acid‐containing natural products and will pave the way for targeted applications of these bioactive alkaloids.

## Supporting Information

Additional results, experimental procedures, analytics, and NMR spectra are available in the Supporting Information (PDF). The authors have cited additional references within the Supporting Information.^[^ ^118^, ^119^, ^120^, ^121^, ^122^, ^123^, ^124^, ^125^, ^126^, ^127^, ^128^, ^129^, ^130^, ^131^, ^132^, ^133^, ^134^, ^135^, ^136^, ^137^ ^]^

## Conflict of Interest

The authors declare no conflict of interest.

## Supporting information



Supporting Information

## Data Availability

The data that support the findings of this study are available in the supplementary material of this article.
