# Supplementary material for: Functionalized in Triplicate: A Ring‐By‐Ring Approach to Tailored Prodiginine Derivatives for Site‐Specific Conjugation Through Click Chemistry
Source: Chemistry. 2025 Jul 14;31(43):e202502066. doi: 10.1002/chem.202502066 (PMC12319383; doi:10.1002/chem.202502066)

Supporting Information

Functionalised in Triplicate: A Ring-by-Ring Approach to Tailored Prodigiosin Derivatives for Site-Specific Conjugation

T. Moritz Weber[a] and Jörg Pietruszka*[a,b]

[a] T. M. Weber, Prof. Dr. J. Pietruszka
Institute of Bioorganic Chemistry
Heinrich Heine University Düsseldorf and Bioeconomy Science Centre (BioSC)
im Forschungszentrum Jülich, Geb. 15.8, 52428 Jülich, Germany
E-mail: j.pietruszka@fz-juelich.de, tiweb103@hhu.de

[b] Prof. Dr. J. Pietruszka
Institute of Bio- and Geosciences (IBG-1: Biotechnology)
Forschungszentrum Jülich
Wilhelm-Johnen-Straße, 52428 Jülich, Germany

Table of Contents

Abbreviations........S5
Extended Data........S5
Synthesis of the Alkylated C-Ring Monopyrrole........S5
Synthesis of the Functionalized A-Ring Intermediates........S5
Optimization of the One-Step Reduction of A-Ring Intermediate Ethyl 5-oxo-5-(1*H*-pyrrol-2-yl)pentanoate........S6
Comments on the Isolation of the *N*-Boc-Pyrrole-2-Boronic Acids **17** and **31**........S7
Ligand Screening for Suzuki-Miyaura Cross-Coupling Reactions........S8
Identification and Prevention Strategies for Grease Contaminations........S9
O-Alkylation of Tetramic Acid via Tosylation........S12
Alternative Route Toward Hydroxylated Monopyrrole Via Hydroboration-Oxidation Sequence........S12
Preparation of Succinyl Monomethyl Ester........S13
Synthesis of the Bifunctional Alkyne-Maleimide Linker........S13
Structure of the Tripodal Cu(I)/Cu(II) Ligand TDETA........S13
Protein-Prodigiosin Conjugates........S14
Absorption Spectra and Molar Extinction Coefficients of Unlabeled Prodiginines........S15
Absorption and Fluorescence Spectra of *F*-BODIPY A-Ring Azide **49**........S17
Synthetic Procedures........S18
General information........S18
2-Methyl-3-propyl-1*H*-pyrrole (**5**)........S18
5-Methyl-4-propyl-1*H*-pyrrole-2-carbaldehyde (**7**)........S18
(*Z*)-4-Methoxy-5-((5-methyl-4-propyl-1*H*-pyrrol-2-yl)methylene)-1,5-dihydro-2*H*-pyrrol-2-one (**9**)........S19
(*Z*)-4-Methoxy-5-((5-methyl-4-propyl-2*H*-pyrrol-2-ylidene)methyl)-1*H*-pyrrol-2-yl trifluoromethanesulfonate (**10**)........S19
Ethyl 5-oxo-5-(1*H*-pyrrol-2-yl)pentanoate (**12**)........S19
5-(1*H*-Pyrrol-2-yl)pentan-1-ol (**13**)........S20
5-(1*H*-Pyrrol-2-yl)pentyl 4-methylbenzenesulfonate (**14**)........S20
tert-Butyl 2-(5-(tosyloxy)pentyl)-1*H*-pyrrole-1-carboxylate (**15**)........S21
tert-Butyl 2-(5-azidopentyl)-1*H*-pyrrole-1-carboxylate (**16**)........S21
(5-(5-Azidopentyl)-1-(*tert*-butoxycarbonyl)-1*H*-pyrrol-2-yl)boronic acid (**17**)........S21
(*Z*)-5-(5-Azidopentyl)-4'-methoxy-5'-((5-methyl-4-propyl-1*H*-pyrrol-2-yl)methylene)-1*H*,5'*H*-[2,2'-bipyrrol]-1'-ium chloride (**2·HCl**)........S22
3-(5-(5-Azidopentyl)-1*H*-pyrrol-2-yl)-5,5-difluoro-1-methoxy-7-methyl-8-propyl-5*H*-5λ^4,6λ^4-dipyrrolo[1,2-*c*:2',1'-*f*][1,3,2]diazaborinine (**49**)........S22
tert-Butyl 4-hydroxy-2-oxo-2,5-dihydro-1*H*-pyrrole-1-carboxylate (**18**)........S23
4-Azidobutan-1-ol (**21**)........S23
tert-Butyl 4-(4-azidobutoxy)-2-oxo-2,5-dihydro-1*H*-pyrrole-1-carboxylate (**23**)........S23
4-(4-Azidobutoxy)-1,5-dihydro-2*H*-pyrrol-2-one (**24**)........S23
(*Z*)-4-(4-Azidobutoxy)-5-((5-methyl-4-propyl-1*H*-pyrrol-2-yl)methylene)-1,5-dihydro-2*H*-pyrrol-2-one (**25**)........S24
(*Z*)-4-(4-Azidobutoxy)-5-((5-methyl-4-propyl-2*H*-pyrrol-2-ylidene)methyl)-1*H*-pyrrol-2-yl trifluoromethanesulfonate (**26**)........S24
(1*H*-Pyrrol-2-yl)pentan-1-one (**28**)........S24
2-Pentyl-1*H*-pyrrole (**29**)........S25
tert-Butyl 2-pentyl-1*H*-pyrrole-1-carboxylate (**30**)........S25
(1-(*tert*-Butoxycarbonyl)-5-pentyl-1*H*-pyrrol-2-yl)boronic acid (**31**)........S25
(*Z*)-4'-(4-Azidobutoxy)-5'-((5-methyl-4-propyl-1*H*-pyrrol-2-yl)methylene)-5-pentyl-1*H*,5'*H*-[2,2'-bipyrrol]-1'-ium chloride (**3·HCl**)........S26
4-Methoxy-4-oxobutanoic acid (**S23**)........S26
Methyl 4-chloro-4-oxobutanoate (**34**)........S26
Ethyl 4-(4-methoxy-4-oxobutanoyl)-5-methyl-1*H*-pyrrole-2-carboxylate (**35**)........S27
Ethyl 4-(4-hydroxybutyl)-5-methyl-1*H*-pyrrole-2-carboxylate (**36**)........S27
4-(2-Methyl-1*H*-pyrrol-3-yl)butan-1-ol (**32**)........S27
3-(4-Azidobutyl)-2-methyl-1*H*-pyrrole (**38**)........S28
4-(4-Azidobutyl)-5-methyl-1*H*-pyrrole-2-carbaldehyde (**39**)........S28
(*Z*)-5-((4-(4-Azidobutyl)-5-methyl-1*H*-pyrrol-2-yl)methylene)-4-methoxy-1,5-dihydro-2*H*-pyrrol-2-one (**40**)........S29
(*Z*)-5-((4-(4-Azidobutyl)-5-methyl-2*H*-pyrrol-2-ylidene)methyl)-4-methoxy-1*H*-pyrrol-2-yl trifluoromethanesulfonate (**41**)........S29
(*Z*)-5'-((4-(4-Azidobutyl)-5-methyl-1*H*-pyrrol-2-yl)methylene)-4'-methoxy-5-pentyl-1*H*,5'*H*-[2,2'-bipyrrol]-1'-ium chloride (**4·HCl**)........S29
Bifunctional Alkyne-Maleimide Linker........S30
Methyl 2,5-dioxo-2,5-dihydro-1*H*-pyrrole-1-carboxylate (**S25**)........S30
tert-Butyl (2-(2,5-dioxo-2,5-dihydro-1*H*-pyrrol-1-yl)ethyl)carbamate (**S27**)........S30
2-(2,5-Dioxo-2,5-dihydro-1*H*-pyrrol-1-yl)ethan-1-aminium 2,2,2-trifluoroacetate (**S28**)........S30

N-(2-(2,5-Dioxo-2,5-dihydro-1*H*-pyrrol-1-yl)ethyl)hex-5-ynamide (**42**) ... S31
Homo-Dimeric Zinc Complexes of Prodigiosin Azides ... S31
Prodigiosin A-Ring Azide Zinc Complex (**43**) ... S31
Prodigiosin B-Ring Azide Zinc Complex (**44**) ... S31
Prodigiosin C-Ring Azide Zinc Complex (**45**) ... S32
(*Z*)-5-(5-(4-(4-((2-(2,5-Dioxo-2,5-dihydro-1*H*-pyrrol-1-yl)ethyl)amino)-4-oxobutyl)-1*H*-1,2,3-triazol-1-yl)pentyl)-4'-methoxy-5'-((5-methyl-4-propyl-1*H*-pyrrol-2-yl)methylene)-1*H*,5'*H*-[2,2'-bipyrrol]-1'-ium chloride (**46**) ... S32
(*Z*)-4'-(4-(4-(4-((2-(2,5-Dioxo-2,5-dihydro-1*H*-pyrrol-1-yl)ethyl)amino)-4-oxobutyl)-1*H*-1,2,3-triazol-1-yl)butoxy)-5'-((5-methyl-4-propyl-1*H*-pyrrol-2-yl)methylene)-5-pentyl-1*H*,5'*H*-[2,2'-bipyrrol]-1'-ium chloride (**47**) ... S33
(*Z*)-5'-((4-(4-(4-(4-((2-(2,5-Dioxo-2,5-dihydro-1*H*-pyrrol-1-yl)ethyl)amino)-4-oxobutyl)-1*H*-1,2,3-triazol-1-yl)butyl)-5-methyl-1*H*-pyrrol-2-yl)methylene)-4'-methoxy-5-pentyl-1*H*,5'*H*-[2,2'-bipyrrol]-1'-ium chloride (**48**) ... S33
(*Z*)-5'-((5-Methyl-4-propyl-1*H*-pyrrol-2-yl)methylene)-4'-(4-(4-(15-oxo-19-((3a*S*,4*S*,6a*R*)-2-oxohexahydro-1*H*-thieno[3,4-*d*]imidazol-4-yl)-2,5,8,11-tetraoxa-14-azanonadecyl)-1*H*-1,2,3-triazol-1-yl)butoxy)-5-pentyl-1*H*,5'*H*-[2,2'-bipyrrol]-1'-ium chloride (**50**) ... S34
1-(1*H*-Pyrrol-2-yl)pent-4-en-1-one (**S14**) ... S34
2-(Pent-4-en-1-yl)-1*H*-pyrrole (**S2**) ... S35
5-Chloro-1-(1*H*-pyrrol-2-yl)pentan-1-one (**S3**) ... S35
tert-Butyl 2-oxo-4-(tosyloxy)-2,5-dihydro-1*H*-pyrrole-1-carboxylate (**S7**) ... S35
(*Z*)-4'-Methoxy-5'-((5-methyl-4-propyl-1*H*-pyrrol-2-yl)methylene)-1*H*,5'*H*-[2,2'-bipyrrol]-1'-ium chloride (**S17**) ... S35
N-Methoxy-*N*-methylhex-5-enamide (**S18**) ... S36
Hept-6-en-2-one (**S19**) ... S36
(*E*/*Z*)-Hept-6-en-2-one oxime (**S20**) ... S36
3-(But-3-en-1-yl)-2-methyl-1*H*-pyrrole (**S9**) ... S37
4-(2-Methyl-1*H*-pyrrol-3-yl)butan-1-ol (**32**) ... S37
Analytical Procedures ... S38
Coupled Gas Chromatography-Mass Spectrometry (GC-MS) ... S38
Physical Procedures ... S38
Experimental Determination of Molar Extinction Coefficients ... S38
Biochemical and Microbiological Procedures ... S38
General Information ... S38
Bacterial Strains and Plasmids ... S39
Heterologous Protein Synthesis ... S39
Purification of PyoS2 Derivatives ... S39
Maleimide Labeling of PyoS2 Cysteine Mutants ... S39
Protein Quantification ... S40
Sodium Dodecyl Sulfate Polyacrylamide Gel Electrophoresis (SDS-PAGE) ... S40
Electrospray Ionization Mass Spectrometry (ESI-MS) ... S40
References ... S41
NMR Spectra ... S44
(*E*/*Z*)-Hexan-2-one oxime (**S1**) ... S44
2-Methyl-3-propyl-1*H*-pyrrole (**5**) ... S45
5-Methyl-4-propyl-1*H*-pyrrole-2-carbaldehyde (**7**) ... S46
(*Z*)-4-Methoxy-5-((5-methyl-4-propyl-1*H*-pyrrol-2-yl)methylene)-1,5-dihydro-2*H*-pyrrol-2-one (**9**) ... S47
(*Z*)-4-Methoxy-5-((5-methyl-4-propyl-2*H*-pyrrol-2-ylidene)methyl)-1*H*-pyrrol-2-yl trifluoromethanesulfonate (**10**) ... S48
Ethyl 5-oxo-5-(1*H*-pyrrol-2-yl)pentanoate (**12**) ... S49
5-(1*H*-Pyrrol-2-yl)pentan-1-ol (**13**) ... S50
5-(1*H*-Pyrrol-2-yl)pentyl 4-methylbenzenesulfonate (**14**) ... S51
tert-Butyl 2-(5-(tosyloxy)pentyl)-1*H*-pyrrole-1-carboxylate (**15**) ... S52
tert-Butyl 2-(5-azidopentyl)-1*H*-pyrrole-1-carboxylate (**16**) ... S53
(5-(5-Azidopentyl)-1-(*tert*-butoxycarbonyl)-1*H*-pyrrol-2-yl)boronic acid (**17**) ... S54
(*Z*)-5-(5-Azidopentyl)-4'-methoxy-5'-((5-methyl-4-propyl-1*H*-pyrrol-2-yl)methylene)-1*H*,5'*H*-[2,2'-bipyrrol]-1'-ium chloride (**2·HCl**) ... S55
3-(5-(5-Azidopentyl)-1*H*-pyrrol-2-yl)-5,5-difluoro-1-methoxy-7-methyl-8-propyl-5*H*-5λ^4,6λ^4-dipyrrolo[1,2-*c*:2',1'-*f*][1,3,2]diazaborinine (**49**) ... S56
tert-Butyl 4-hydroxy-2-oxo-2,5-dihydro-1*H*-pyrrole-1-carboxylate (**18**) ... S57
4-Azidobutan-1-ol (**21**) ... S58
tert-Butyl 4-(4-azidobutoxy)-2-oxo-2,5-dihydro-1*H*-pyrrole-1-carboxylate (**23**) ... S59
4-(4-Azidobutoxy)-1,5-dihydro-2*H*-pyrrol-2-one (**24**) ... S60
(*Z*)-4-(4-Azidobutoxy)-5-((5-methyl-4-propyl-1*H*-pyrrol-2-yl)methylene)-1,5-dihydro-2*H*-pyrrol-2-one (**25**) ... S61
(*Z*)-4-(4-Azidobutoxy)-5-((5-methyl-4-propyl-2*H*-pyrrol-2-ylidene)methyl)-1*H*-pyrrol-2-yl trifluoromethanesulfonate (**26**) ... S62
(1*H*-Pyrrol-2-yl)pentan-1-one (**28**) ... S63
2-Pentyl-1*H*-pyrrole (**29**) ... S64
tert-Butyl 2-pentyl-1*H*-pyrrole-1-carboxylate (**30**) ... S65

(1-(*tert*-Butoxycarbonyl)-5-pentyl-1*H*-pyrrol-2-yl)boronic acid (**31**)....S66
(*Z*)-4'-(4-Azidobutoxy)-5'-((5-methyl-4-propyl-1*H*-pyrrol-2-yl)methylene)-5-pentyl-1*H*,5'*H*-[2,2'-bipyrrol]-1'-ium chloride (**3·HCl**)....S67
4-Methoxy-4-oxobutanoic acid (**S23**)....S68
Methyl 4-chloro-4-oxobutanoate (**34**)....S69
Ethyl 4-(4-methoxy-4-oxobutanoyl)-5-methyl-1*H*-pyrrole-2-carboxylate (**35**)....S70
Ethyl 4-(4-hydroxybutyl)-5-methyl-1*H*-pyrrole-2-carboxylate (**36**)....S71
4-(2-Methyl-1*H*-pyrrol-3-yl)butan-1-ol (**32**) (from decarboxylation of pyrrole **36**)....S72
3-(4-Azidobutyl)-2-methyl-1*H*-pyrrole (**38**)....S73
4-(4-Azidobutyl)-5-methyl-1*H*-pyrrole-2-carbaldehyde (**39**)....S74
(*Z*)-5-((4-(4-Azidobutyl)-5-methyl-1*H*-pyrrol-2-yl)methylene)-4-methoxy-1,5-dihydro-2*H*-pyrrol-2-one (**40**)....S75
(*Z*)-5-((4-(4-Azidobutyl)-5-methyl-2*H*-pyrrol-2-ylidene)methyl)-4-methoxy-1*H*-pyrrol-2-yl trifluoromethanesulfonate (**41**)....S76
(*Z*)-5'-((4-(4-Azidobutyl)-5-methyl-1*H*-pyrrol-2-yl)methylene)-4'-methoxy-5-pentyl-1*H*,5'*H*-[2,2'-bipyrrol]-1'-ium chloride (**4·HCl**)...S77
Methyl 2,5-dioxo-2,5-dihydro-1*H*-pyrrole-1-carboxylate (**S25**)....S78
tert-Butyl (2-(2,5-dioxo-2,5-dihydro-1*H*-pyrrol-1-yl)ethyl)carbamate (**S27**)....S79
2-(2,5-Dioxo-2,5-dihydro-1*H*-pyrrol-1-yl)ethan-1-aminium 2,2,2-trifluoroacetate (**S28**)....S80
N-(2-(2,5-Dioxo-2,5-dihydro-1*H*-pyrrol-1-yl)ethyl)hex-5-ynamide (**42**)....S81
Prodigiosin A-Ring Azide Zinc-Complex (**43**)....S82
Prodigiosin B-Ring Azide Zinc-Complex (**44**)....S83
Prodigiosin C-Ring Azide Zinc-Complex (**45**)....S84
(*Z*)-5-(5-(4-(4-((2-(2,5-Dioxo-2,5-dihydro-1*H*-pyrrol-1-yl)ethyl)amino)-4-oxobutyl)-1*H*-1,2,3-triazol-1-yl)pentyl)-4'-methoxy-5'-((5-methyl-4-propyl-1*H*-pyrrol-2-yl)methylene)-1*H*,5'*H*-[2,2'-bipyrrol]-1'-ium chloride (**46**)....S85
(*Z*)-4'-(4-(4-(4-((2-(2,5-Dioxo-2,5-dihydro-1*H*-pyrrol-1-yl)ethyl)amino)-4-oxobutyl)-1*H*-1,2,3-triazol-1-yl)butoxy)-5'-((5-methyl-4-propyl-1*H*-pyrrol-2-yl)methylene)-5-pentyl-1*H*,5'*H*-[2,2'-bipyrrol]-1'-ium chloride (**47**)....S86
(*Z*)-5'-((4-(4-(4-(4-((2-(2,5-Dioxo-2,5-dihydro-1*H*-pyrrol-1-yl)ethyl)amino)-4-oxobutyl)-1*H*-1,2,3-triazol-1-yl)butyl)-5-methyl-1*H*-pyrrol-2-yl)methylene)-4'-methoxy-5-pentyl-1*H*,5'*H*-[2,2'-bipyrrol]-1'-ium chloride (**48**)....S87
(*Z*)-5'-((5-Methyl-4-propyl-1*H*-pyrrol-2-yl)methylene)-4'-(4-(4-(15-oxo-19-((3a*S*,4*S*,6a*R*)-2-oxohexahydro-1*H*-thieno[3,4-*d*]imidazol-4-yl)-2,5,8,11-tetraoxa-14-azanonadecyl)-1*H*-1,2,3-triazol-1-yl)butoxy)-5-pentyl-1*H*,5'*H*-[2,2'-bipyrrol]-1'-ium chloride (**50**)....S88
2-(Pent-4-en-1-yl)-1*H*-pyrrole (**S2**)....S89
5-Chloro-1-(1*H*-pyrrol-2-yl)pentan-1-one (**S3**)....S90
tert-Butyl 2-oxo-4-(tosyloxy)-2,5-dihydro-1*H*-pyrrole-1-carboxylate (**S7**)....S91
3-(But-3-en-1-yl)-2-methyl-1*H*-pyrrole (**S9**)....S92
1-(1*H*-Pyrrol-2-yl)pent-4-en-1-one (**S14**)....S93
(*Z*)-4'-Methoxy-5'-((5-methyl-4-propyl-1*H*-pyrrol-2-yl)methylene)-1*H*,5'*H*-[2,2'-bipyrrol]-1'-ium chloride (**S17**)....S94
N-Methoxy-*N*-methylhex-5-enamide (**S18**)....S95
Hept-6-en-2-one (**S19**)....S96
(*E*/*Z*)-Hept-6-en-2-one oxime (**S20**)....S97
4-(2-Methyl-1*H*-pyrrol-3-yl)butan-1-ol (**32**) (from hydroboration-oxidation of alkene pyrrole **S9**)....S98

Abbreviations

BSA – bovine serum albumin, CuAAC – copper-catalyzed azide-alkyne cycloaddition, 1,2-DCE – 1,2-dichloroethane, DCVC – dry column vacuum chromatography, DEAD – diethyl azodicarboxylate, DIBAL-H – diisobutyl aluminium hydride, DIPEA – diisopropylethylamine, DMAP – 4-(dimethylamino)pyridine, DMF – dimethylformamide, dppf – 1,1'-bis(diphenylphosphanyl)ferrocene, DPS – 2,2'-dipyridyl disulfide, DTT – dithiothreitol, EDC – 1-ethyl-3-(3-dimethylaminopropyl)carbodiimide, IPTG – isopropyl-β-D-thiogalactopyranoside, LB – lysogeny broth, NMM – *N*-methyl morpholine, PyBOP – benzotriazol-1-yloxytripyrrolidinophosphonium hexafluorophosphate, PyoS2 – pyocin S2, RPM – revolutions per minute, RuPhos – [2',6'-bis(propan-2-yloxy)-[1,1'-biphenyl]-2-yl]dicyclohexylphosphane, SDS-PAGE – sodium dodecyl sulfate-polyacrylamide gel electrophoresis, SPhos – dicyclohexyl(2',6'-dimethoxy[1,1'-biphenyl]-2-yl)phosphane, TB – terrific broth, TDETA – tris dimethylethyl triazolyl amine, TEA – triethylamine, TFA – trifluoroacetic acid, THF – tetrahydrofuran, TLC – thin layer chromatography, TMP – 2,2,6,6-tetramethylpiperidine, Tris – tris(hydroxymethyl)aminomethane, XPhos – dicyclohexyl[2',4',6'-tris(propan-2-yl)[1,1'-biphenyl]-2-yl]phosphane.

Extended Data

Synthesis of the Alkylated C-Ring Monopyrrole

The first steps in synthesizing the prodigiosin A-ring azide **2** and the B-ring azide **3** comprise the preparation of a 2,3-alkylated monopyrrole as precursor for the future C-ring. As recently reported by Weber *et al*., hexan-2-one (**6**) was converted on a multigram scale with hydroxylamine hydrochloride and sodium acetate to the corresponding *E*/*Z* mixture of oxime **S1** in quantitative yield and then subjected to a Trofimov pyrrole synthesis under superbasic conditions with degassed DMSO/KOH, and 1,2-dichloroethane (1,2-DCE) as an electrophile, to provide the short-chain monopyrrole **5** in a yield of 54% (Scheme S1).[1-3]

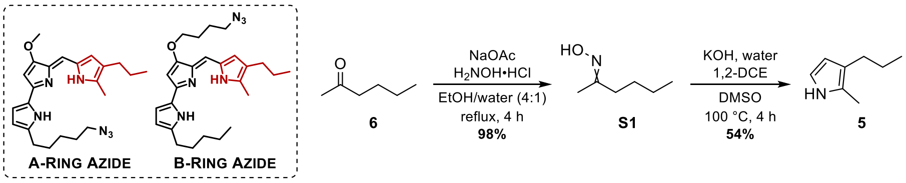

Scheme S1. Synthesis of the alkylated monopyrrole precursor **5** from hexan-2-one (**6**) as a common ground for the prodigiosin A-ring and B-ring azides **2** and **3**. Abbreviations: 1,2-DCE – 1,2-dichloroethane.

Synthesis of the Functionalized A-Ring Intermediates

Early attempts to synthesize the hydroxylated pyrrole **13** as a precursor of the azide-functionalized boronic acid **17** were based on alkene pyrrole **S2**. This was generated from the acylation of 1*H*-pyrrole with activated 4-pentenoic acid **S12** (Scheme S2). The latter was first transformed into the thioester by reacting with 2,2'-dipyridyl disulfide (DPS) and PPh_3 in toluene at ambient temperature.[4] Pyrrolylmagnesium chloride **S13** was then allowed to react with the thioester at −78 °C, giving 81% of the acylated alkene pyrrole **S14**. Although the reaction conditions rendered complete conversion, triphenylphosphine oxide was hard to remove by chromatography on a large scale, accounting for the reduced yield. The subsequent reduction with $NaBH_4$ in *i*PrOH provided the instable alkene pyrrole **S2**.[4-6] Despite the fact that a Brown hydroboration-oxidation sequence with 9-BBN and $NaOH/H_2O_2$ could have been feasible to transform the terminal double bond into a terminal hydroxy group,[7-8] thereby providing the desired intermediate **13**, we sought alternative measures to circumvent the instable alkene intermediate **S2**. Alternatively, we utilized a chlorinated acid chloride for the introduction of a terminal halide. Starting from commercially available 5-chloro-pentanoic acid chloride (**S15**), we were able to avoid the use of PPh_3 and the subsequent generation of PPh_3=O, as in the previous activation of carboxylic acid **S12** via an intermediate thioester. 2-Acylation of 1*H*-pyrrole with pyrrolylmagnesium bromide (**S13**) allowed isolation of the chlorinated acylpyrrole **S3** in a yield of 52%. However, isolation of the acylpyrrole was rather difficult, as chromatographic separation of the two product isomers, presumably 2- and 3-acylated pyrrole, was not possible in conventional two-component eluents (PE/EtOAc, *n*-pentane/CH_2Cl_2, *n*-pentane/MTBE, *n*-pentane/Et_2O). This holds true for TLC and preparative chromatography on silica. Tedious optimization of purification conditions revealed that the addition of 5–10% CH_2Cl_2 to a mixture of *n*-pentane/MTBE is essential, as only this adjustment leads to separation of the isomers and long enough retention on the column for polar interaction with the stationary phase. However, the following reduction of chlorinated acylpyrrole **S3** with $NaBH_4$ with optional supplementation of $CeCl_3$ only resulted in product mixtures of numerous reduction and elimination products [fully reduced alkyl pyrrole **29** (*m/z* 137.12), acyl pyrrole **28** (*m/z* 151.10), chlorinated alkyl pyrrole

S4 (*m/z* 171.08), and the chlorinated elimination product **S16** (*m/z* 169.07, both *E*/*Z* isomers detected)] (cf. Table S1). A brief screening for suitable solvents and additives only led to a maximum of 40% (product ratios determined by GC-MS) of the chlorinated target **S4** in an inseparable mixture of other side products (Table S1). Concludingly, this route was dismissed owing to an unreasonable and uneconomic need for tedious product purification.

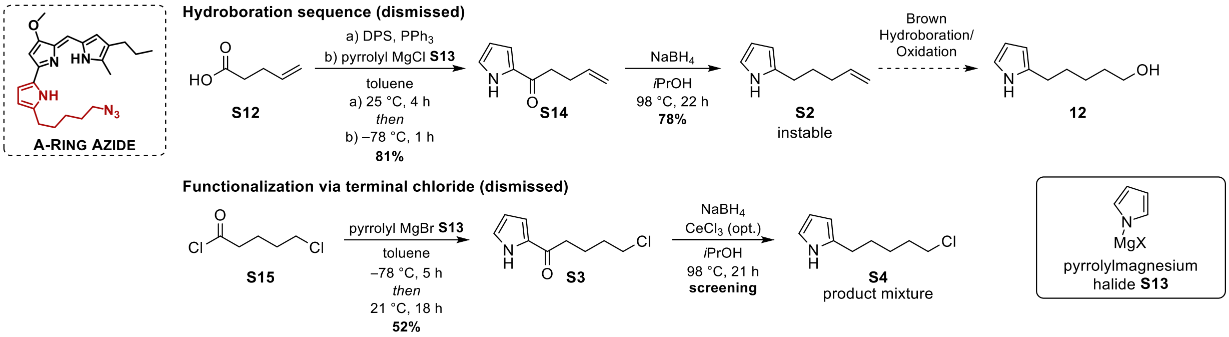

Scheme S2. Synthesis of terminally functionalized 2-alkylated pyrroles as precursors of the prodigiosin A-ring azide **2**. Both routes were dismissed due to a lack of stability (alkene pyrrole **S2**) or non-separable reduction products emerging from the $NaBH_4$-reduction of chlorinated acylpyrrole **S3**. Abbreviations: DPS – 2,2'-dipyridyl disulfide.

Table S1. Screening for the $NaBH_4$/$CeCl_3$ reduction of chlorinated acylpyrrole **S3**.

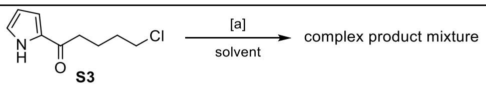

**Reagents**[b]		$NaBH_4$ (2.8) $CeCl_3$ (1.1)	$NaBH_4$ (1.5) $CeCl_3$ (1.1) NaOH (1.5)	$NaBH_4$ (1.5) $CeCl_3$ (1.1)	$NaBH_4$ (1.5)		
**Solvent**		*i*PrOH	*i*PrOH	THF/*i*PrOH (2:1)	*i*PrOH	retention time [min][c]	*m/z*[d]
**Products**	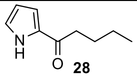 28	33	44	2	43	8.32	151.10
	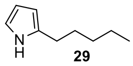 29	3	1	0	2	7.01	137.12
	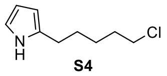 S4	40	15	8	30	9.55	171.08
	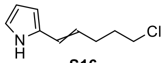 S16	24	40	90	25	9.74 + 9.98	169.07

[a] Conditions: Chlorinated acylpyrrole **S3** (50.0 mg, 269 µmol) was dissolved in 3 mL dry solvent. At 0 °C, $NaBH_4$ (and optional $CeCl_3$) were added. Solutions were refluxed under an inert atmosphere at 98 °C for 21 h, quenched with water, and extracted with MTBE. Area% from GC-MS measurements of reaction extracts in MTBE; [b] Equivalents are indicated in parentheses. [c] Retention time on the GC-MS. The starting material elutes at 10.28 min with an m/z ratio of 185.06. [d] Observed mass/charge ratio (*m/z*) of the detected molecule peaks.

Optimization of the One-Step Reduction of A-Ring Intermediate Ethyl 5-oxo-5-(1*H*-pyrrol-2-yl)pentanoate

Although hydroboration of alkene pyrrole **S2** could be feasible, we aimed for a shortcut to circumvent the delicate alkene intermediate. The solution found for this objective was based on an ethyl ester functionalized acid chloride **11**, which intrinsically already harbored the hydroxy group in another oxidation state. The 2-functionalized acylpyrrole **12** with terminal ethyl ester was subjected to hydride reduction to reduce the ketone and ester functionality in one step. Several metal hydrides in ethereal solvents (THF, glyme, or 1,4-dioxane) or *i*PrOH as an alcoholic solvent were screened for their suitability to facilitate the concomitant reduction of ketone and ester to give access to hydroxylated pyrrole **13** (Table S2). Initially, the usage of $LiAlH_4$ (2.2 eq.) in THF (entry 1) left the starting material

unconverted, while increased amounts of $LiAlH_4$ (5.0 eq.) rendered complete conversion after 4 h. However, GC-MS analyses revealed two reaction products: the fully reduced pyrrole **13** (*m/z* 153.12) and the undesired elimination product **S5** (*m/z* 151.10) in a ratio of 70:30 (entry 2). Less reactive borohydrides ($LiBH_4$ and $NaBH_4$, entries 3 and 4) in *i*PrOH either led to 18% conversion toward the ethyl ester elimination product **AcO-S5** (*m/z* 193.11) or to near-full conversion with exclusive formation of elimination product **S5**, accompanied by severe side products forming. Performing the reaction with $LiAlH_4$ in 1,4-dioxane, with its elevated boiling point, provided a favorable product ratio of 89:11 (entry 5), however, severe formation of unknown side products occurred as well, which would impede downstream purification. No conversion was obtained with diisobutyl aluminium hydride (DIBAL-H) in THF (entry 9). The best results were finally achieved with glyme at 80 °C in the presence of $LiAlH_4$ (5.0 eq.), which gave smooth conversion and a product ratio of 92:8 (entry 8). For all $LiAlH_4$-containing reactions so far (entries 1, 2, 5–8), the metal hydride was fed at 0 °C and stirring at 0 °C was continued for 15 min until hydrogen gas development had ceased, before the solvent was refluxed. To investigate the effect of direct heating, the reaction solution was already heated at 70 °C before $LiAlH_4$ was added to the reaction (entries 10 and 11). Through this experiment, we found that initial cooling of the reaction is crucial to obtain a high amount of the target molecule **13**, as initial heating to 70 °C resulted in unfavorable ratios of 67:33 (at 80 °C reaction temperature) or 42:58 (at 100 °C reaction temperature). In conclusion, the one-step reduction of pyrrole **12** with $LiAlH_4$ (5.0 eq.) in glyme, according to entry 8, gave the best results to attempt a scale-up reaction and to shortcut the instable alkene derivative **S2**.

Table S2. Screening of suitable metal hydrides for the attempted one-step reduction of 2-acylated pyrrole ester **12** to the corresponding 2-alkylated hydroxypentyl pyrrole **13** in ethereal and alcoholic solvents.

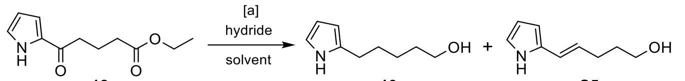

12 **13** **S5**

Entry	Metal hydride (eq.)	Solvent	Temp. [°C]	Time [h]	Ratio[b] (**13**:**S5**)
1[c]	$LiAlH_4$ (2.2)	THF	80	12	no conversion
2[c]	$LiAlH_4$ (5.0)	THF	85	4	70:30
3	$LiBH_4$ (3.0)	*i*PrOH	98	3	0:0[f]
4	$NaBH_4$ (3.0)	*i*PrOH	98	4	0:100[g]
5[c]	$LiAlH_4$ (5.0)	1,4-dioxane	120	3	89:11[g]
6[c],[e]	$LiAlH_4$ (5.0)	THF	98	3	30:70
7[c]	$LiAlH_4$ (5.0)	THF	98	2	52:48
8[c]	$LiAlH_4$ (5.0)	glyme	90	2	92:8
9[c]	DIBAL-H (5.0)	THF	85	1	no conversion
10[d]	$LiAlH_4$ (5.0)	glyme	80	1	67:33
11[d]	$LiAlH_4$ (5.0)	glyme	100	1	42:58

[a] Conditions: Pyrrole **12** (100 mg, 478 µmol, 1.00 eq., 0.25 M in ethereal or alcoholic solvent). The reactants were heated after the addition of metal hydride at the specified temperature under an inert atmosphere. Complete conversion was typically achieved unless stated otherwise; [b] Determined via GC-MS after Fieser workup and extraction with MTBE;[9] [c] $LiAlH_4$ addition at 0 °C and stirring for 15 min at 0 °C before heating; [d] Heated to 70 °C before $LiAlH_4$ addition; [e] $LiAlH_4$ suspension in THF added over 30 min via syringe pump while refluxing the pyrrole in THF; [f] 18% Conversion with the ethyl esters of **13** and **S5** in a 0:100 ratio; [g] With additional side products.

Comments on the Isolation of the *N*-Boc-Pyrrole-2-Boronic Acids 17 and 31

First and foremost, the isolation of pure azide and alkyl boronic acids **17** and **31**, respectively, was the encountered bottleneck of attempts to acquire the A-ring precursors (cf. Scheme 3 and Scheme 4, main article). Although pyrrole-2-boronic acids are stated in the literature to precipitate upon slow solvent removal after methyl ester hydrolysis with diluted HCl and extractive workup with Et_2O,[4, 10] we ended up isolating impure boronic acid as an oily residue in multiple attempts. Finally, repeating the washing step with dilute HCl after isolation of the oil was found to do the trick and allow precipitation of the delicate alkyl boronic acid at first. We hypothesize that residual boric acid impurities, which stem from hydrolysis of excess $B(OMe)_3$, and incomplete protonation of the boronic acid functional group, might hamper precipitation in the first step. Furthermore, the typically used Et_2O is not ideal for boronic acid precipitation, as the pyrrole-2-boronic acids are considerably soluble in ethereal solvents. Substitution by even less polar hydrocarbon solvents (*n*-pentane, *n*-heptane, PE) was met with failure, as the products immediately decomposed. Instead, stabilizer-free iPr_2O (stabilizer BHT removed by rotary evaporation) was the most suitable compromise between a less polar solvent than diethyl ether, but a more polar solvent than

hydrocarbon solvents. With this optimized procedure of boronic acid purification, pure azide boronic acid **17** and alkyl boronic acid **31** were isolated in yields of 41% and 66% as light-yellow solids. While the alkyl boronic acid **31** was relatively stable and enabled storage at −20 °C for multiple months, the very delicate azide boronic acid **17** decomposed within less than three days at −20 °C and underwent rapid degradation under vacuum.

Ligand Screening for Suzuki-Miyaura Cross-Coupling Reactions

Modern synthetic routes that aim to build the tripyrrole scaffold of prodigiosin typically utilize a Palladium-catalyzed Suzuki-Miyaura cross-coupling to merge boronic acid derivatives of the A-ring pyrrole with brominated or triflated precursors of the B-ring pyrrole.[4, 11-18] For this purpose, tetrakis(triphenylphosphine)palladium(0) was shown to be an appropriate catalyst for the cross-coupling of pyrrole-2-boronic acids,[19] before being first applied by Fürstner *et al.* in the total synthesis of nonylprodigiosin.[4] Since then, the precatalyst has been used throughout numerous synthetic contributions related to prodigiosin, tambjamine, or marineosin chemistry.[15, 20-24] However, the commercially available tetra-substituted Pd(0) precatalyst requires elevated temperatures (>80 °C for OTf and Br) to shed the gratuitous triphenylphosphine ligands and leverage the oxidative addition of $Pd(PPh_3)_2$ into the carbon-halide bond (reactivity I > OTf ≈ Br >> Cl) in the first step of the catalytic cycle.[25-27] As organic azides and pyrrole-2-boronic acids in particular are known to be extremely delicate,[4, 28-33] we desired to screen for Pd ligands that would allow coupling at a decreased temperature of 60 °C in reasonable yields. Although MIDA boronates are air-stable boronic acid derivatives and increase the overall yield of Suzuki-Miyaura cross-couplings with unstable boronic acids through the slow release of boronic acid under mild basic conditions,[28] their preparation requires the free pyrrole-2-boronic acids and temperatures >70 °C when using the dehydrated MIDA anhydride.[34] As MIDA boronate protection is concomitant with a significant loss of yield even for the most stable unsubstituted *N*-Boc-pyrrole-2-boronic acid (**S6**),[28] we did not pursue this strategy to increase the stability of the utilized boronic acids. Hence, triflate **10** with connatural alkyl substitution to prodigiosin and commercially available *N*-Boc-pyrrole-2-boronic acid **S6** were chosen as stable dummy substrates and heated at 60 °C in the presence of unactivated $Pd(OAc)_2$ (5 mol%) and a small excess of ligand (6 mol%). Besides the tetrakis(triphenylphosphine)palladium precatalyst, we decided to use the common dppf ligand and ligands from the Buchwald group (RuPhos,[35] XPhos,[35-37] and SPhos[35-37]) for the screening with K_3PO_4 as inorganic base in a solvent system of degassed *n*BuOH/water (4:1) (for ligand structures see Scheme S3). With the $Pd(PPh_3)_4$ precatalyst (Table S3, entry 1), a yield of 55% was achieved for the synthesis of the prodigiosin analog **S17** and this was used as a reference to rate the other phosphine ligands. With the ferrocene ligand dppf, the yield dropped to 35% (entry 2) and utilization of XPhos only led to an isolated yield of 18% (entry 3). RuPhos instead could increase the yield to 76% (entry 4) and with SPhos the yield rose even further to 84% (entry 5), being an improvement by approximately 50%pt compared to $Pd(PPh_3)_4$. A further decrease in reaction temperature from 60 °C to 40 °C caused the yield in the SPhos-promoted coupling to drop to 60% (entry 6), proving the need for elevated temperatures of ≥60 °C to obtain good conversions and yields >80% for the coupling of aliphatic triflates and unsubstituted pyrrole-2-boronic acid. Consequently, the reaction system with $Pd(OAc)_2$/SPhos at 60 °C was used for all upcoming Suzuki-Miyaura cross-couplings throughout this work without further investigations on catalyst loading or the Pd/ligand ratio. In addition, we noted recurring contaminations with persistent and non-removable *H*-grease, especially in small-scale reactions with prodigiosin. After screening common laboratory equipment for the origin of *H*-grease, we found that the cotton wool for filtration purposes was the dominant source of *H*-grease. Nonetheless, many other daily expendable laboratory goods (unprocessed solvents, foldable septa, gloves, etc.) were found to contribute to this problem. For this reason, we developed a laboratory practice strategy and custom glassware to eliminate any traces of *H*-grease beforehand (Table S4, Figure S1−S3).

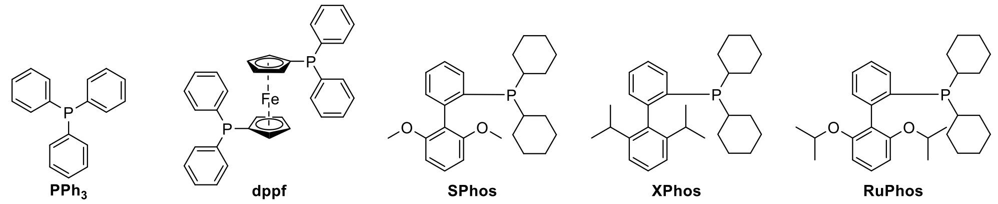

Scheme S3. Structures of the phosphine-based Pd ligands for Suzuki-Miyaura cross-coupling screening reactions.

Table S3. Ligand screening for the Suzuki-Miyaura coupling between boronic acid **S6** and triflate **10** under mild reaction conditions.

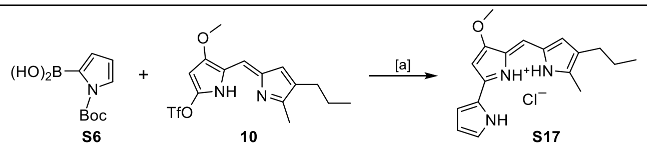

S6 **10** **S17**

Entry	Ligand	Temp. [°C]	Yield[b]
1	$Pd(PPh_3)_4$[c]	60	55
2	dppf[d]	60	35
3	XPhos	60	18
4	RuPhos	60	76
5	SPhos	60	84
6	SPhos	40	60

[a] Conditions: Triflate **10** (50.0 mg, 132 µmol, 1.00 eq.), *N*-Boc-pyrrole-2-boronic acid (**S6**, 2.00 eq.), ligand (see Table S3, 0.06 eq.), $Pd(OAc)_2$ (0.05 eq.), and K_3PO_4 (2.00 eq.) in degassed *n*BuOH/water (4:1, 910 µL). Reactions were stirred under inert atmosphere at 60 °C (40 °C for entry 6) for 18 h; [b] Isolated yields; [c] Tetrakis(triphenylphosphine)palladium(0) (0.05 eq.) was used to replace $Pd(OAc)_2$ and additional ligand supplementation; [d] 1,1'-Bis(diphenylphosphanyl)ferrocene.

Identification and Prevention Strategies for Grease Contaminations

In the last cross-coupling steps of the prodiginine synthesis, we encountered recurrent and severe *H*-grease impurities of unknown origin, which could not be removed by chromatography or washing with distilled *n*-pentane. Non-distilled solvents and in particular cotton wool for filtration of crude reaction products were found to contain significant amounts of non-volatile *H*-grease which can then be transferred into reaction products as prodiginines or the alkyne maleimide linker without further chance of removal. For this reason, purchased solvents were distilled on lab-scale rotary evaporators to determine the amount of residual *H*-grease and other insoluble impurities present (without prior distillation). In addition, 5 g of commercial cotton wool (Jean Carol Cosmetic-Pleats Naturelle, exp. date 20.03.2029, Lot 0642) was washed with distilled *n*-pentane (2 x 350 mL) and distilled CH_2Cl_2 (2 x 250 mL) and the solvent was evaporated for quantification. Insoluble residues were dissolved in 500 µL $CDCl_3$ and analyzed by ^{1}H NMR spectroscopy (600 MHz, 16 scans, Figure S1). From 2 L of solvent, the following quantities of grease were obtained (and other non-volatile polymers) (single determination, Table S4). Furthermore, 20.82 mg of non-volatile impurities were obtained from 5 g of domestic cotton wool.

Table S4. Quantification of non-volatile impurities from commercial organic solvents.

Entry	Solvent	Grade	Supplier	Lot	Non-volatile impurities [mg L^{-1}]
1	MTBE	99.5%, for synthesis, 25 L tin barrel	Carl Roth	240296596	19.51
2	*n*-Pentane	>95%, extra pure, 25 L tin barrel	Carl Roth	391311713	17.75
3	PE	40–65 °C, anhydrous, 25 L tin barrel	VWR	21J114009	14.78
4	EtOAc	>99%, technical, 25 L plastic container	VWR	21K024016	17.93
5	CH_2Cl_2	99%, lab. reagent grade, stab. with amylene, 25 L tin barrel	Fisher Chemical	2100396	94.31

Additionally, we analyzed other standard laboratory consumables for grease impurities, such as foldable rubber and silicone septa (Saint Gobain), nitrile gloves and hand cream (Th. Geyer), commercially available degreased cotton wool (Brand), or Baysilone silicone paste (Bayer) for airtight connection of glass joints (Figure S2). As expected, silicone paste and silicone septa contained mostly silicone grease. All other samples showed detectable amounts of *H*-grease. While the nitrile gloves and degreased cotton wool still contained minor amounts of *H*-grease, rubber septa and the laboratory hand cream contained significant amounts of the uninvited guest.

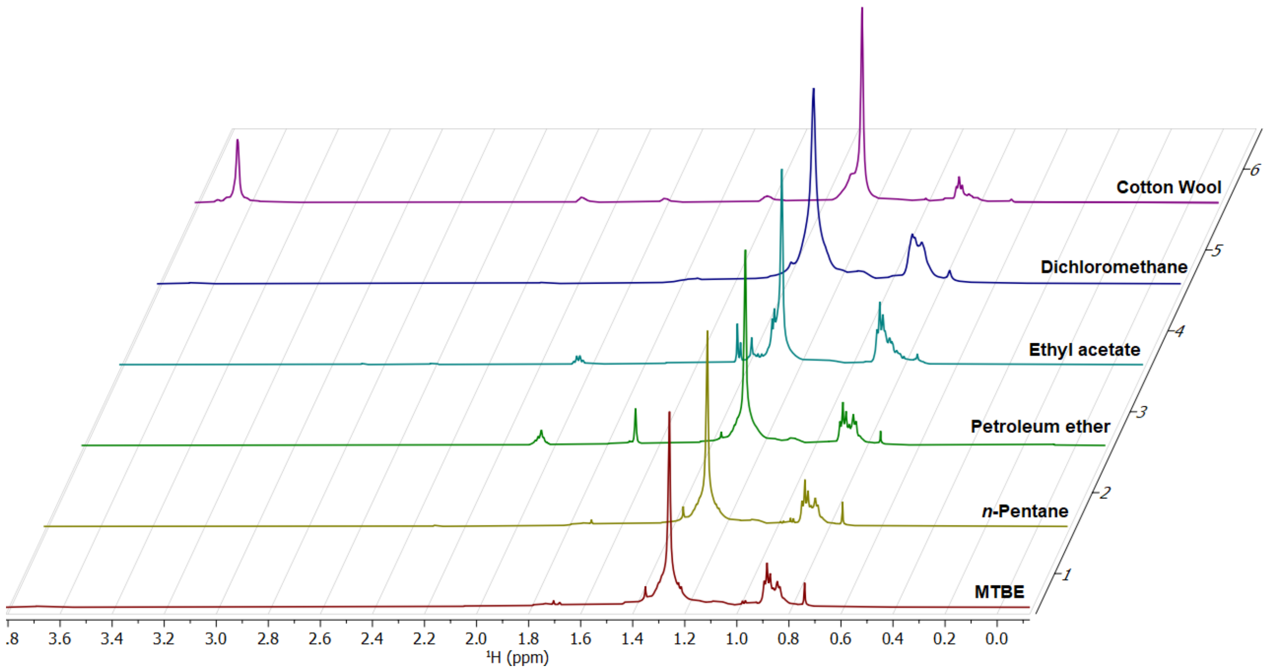

Figure S1. Stacked excerpts of ^{1}H NMR spectra of non-volatile residues from solvent evaporation of 2 L of organic solvents or 5 g of domestic cotton wool in $CDCl_3$. *H*-Grease gives a triplet at 0.88 ppm and a broad singlet at 1.26 ppm.[38]

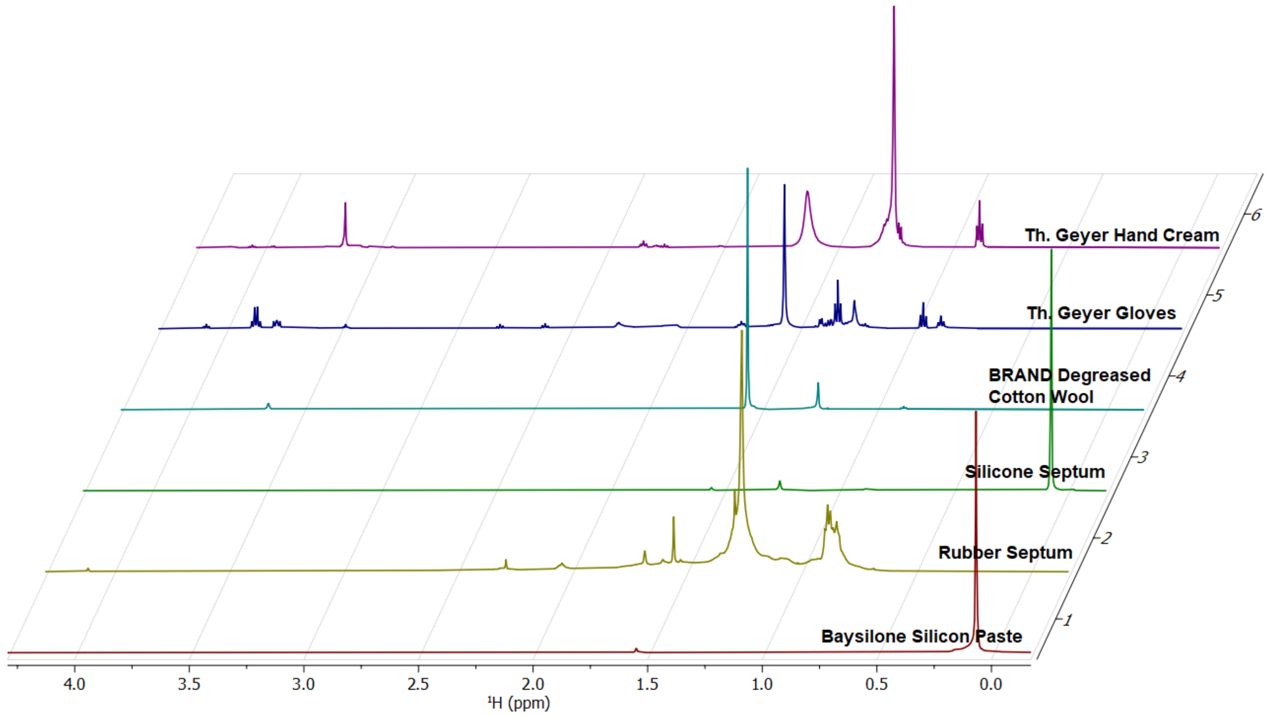

Figure S2. Stacked excerpts of ^{1}H NMR spectra of non-volatile residues from common laboratory utensils in $CDCl_3$. *H*-Grease gives a triplet at 0.88 ppm and a broad singlet at 1.26 ppm, while silicone grease is characterized by a single singlet at 0.07 ppm.[38]

Those observations prompted us to develop the following laboratory practice strategies to avoid the introduction of non-removable grease impurities into prodiginine samples:

a) All organic solvents were distilled before usage and stored in clean glass containers.

b) Crude reaction products were filtered over commercially available degreased cotton wool (Brand, Product No. BR28205, cotton roving, degreases cotton wool, approximately 1.3 g m^{-1}), which had been degreased again by washing with distilled *n*-pentane, PE, or CH_2Cl_2.

c) Plastic articles (pipettes, measuring cylinders, spray bottles) were avoided wherever possible and replaced by adequate glass substitutes.

d) Prodigiosin-containing reactions were not sealed with foldable rubber or silicone septa (especially in combination with chlorinated solvents) but with a custom-made adapter that allows utilization of disposable PTFE-coated septa and avoids long-term exposure of septa to vapor of organic solvents through a PTFE stopcock (Figure S3).

e) Greasing of solvent-exposed glass joints was avoided if not essential.

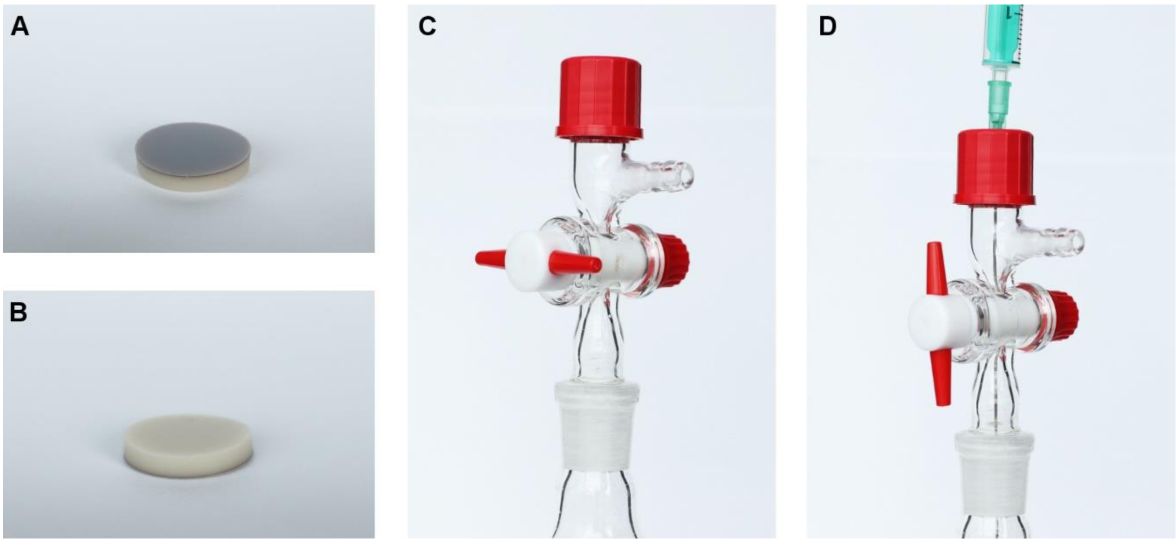

Figure S3. Custom-made glass adapter. If connected to a Schlenk flask, solids can be added under inert atmosphere upon removal of the adapter. If connected to a round bottom flask instead, every flask can be converted into a Schlenk-type flask for air-sensitive reactions. **A**. BOLA cap seal (PTFE + silicone, Bohlender, Product No. H973-18, 16.8 mm diameter, 3.3 mm thickness), bottom view. **B**. BOLA cap seal, top view. **C**. Custom-made glass adapter with standard ground joint cone (14/23), a one-way PTFE stopcock, an olive with 8 mm outer diameter for connection to the Schlenk line, and a screw thread (GL18) for mounting a screw cap (GL18) with disposable septa (cf. **A** and **B**). **D**. Addition of liquids through the borehole of the PTFE stopcock with a syringe.

O-Alkylation of Tetramic Acid via Tosylation

For introducing the azide moiety into the tetramic acid B-ring precursor, we first attempted *O*-functionalization via *O*-tosylation of enol **18** and subsequent etherification under DABCO-catalysis with an aliphatic alcohol based on literature reports (Scheme S4, supporting information). No reaction was observed between tosylate **S7** and the tested aliphatic alcohol toward the *O*-alkylated intermediate **S8**, which we attributed to the lack of nucleophilicity of the aliphatic alcohol. As the protocol was initially developed for aromatic benzothiols as nucleophiles and already negatively affected by electron-withdrawing substituents on the benzothiol nucleophile,[39] this outcome is unsurprising.

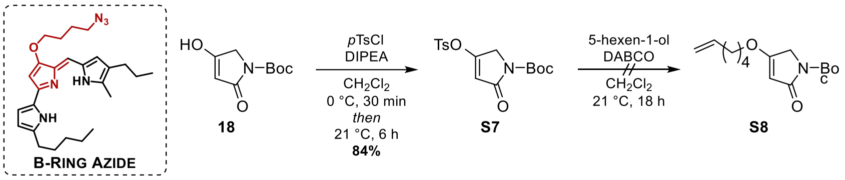

Scheme S4. Unproductive route for the *O*-alkylation of the prodigiosin B-ring azide precursors **18**. Abbreviations: DABCO – 1,4-diazabicyclo(2.2.2)octane, DIPEA – diisopropylethylamine.

Alternative Route Toward Hydroxylated Monopyrrole Via Hydroboration-Oxidation Sequence

For introducing the C-ring azide, we aimed to mimic the natural 5-methyl-4-pentyl substitution of the natural product prodigiosin (**1**). In the first place, we adapted the synthetic route of Scheme S1 to introduce a terminal double bond for further functionalization of the monopyrrole. Unfortunately, the limited commercial availability of functionalized ketones necessitated the synthesis to start from 5-hexenoic acid (**S10**) (Scheme S5). Activation of the carboxylic acid with the HCl salt of 1-ethyl-3-(3-dimethylaminopropyl)carbodiimide (EDC) facilitated conversion to the corresponding Weinreb amide **S18**, which could be reduced with methylmagnesium chloride to release 6-hepten-2-one (**S19**) upon acidic workup with aqueous ammonium chloride.[40] The subsequent reaction with hydroxylamine in the presence of sodium acetate as base rendered the *E*/*Z* mixture of oxime **S20** in high yield.[3] This route benefits from complete conversion and water solubility of all reactants so far, making purification other than aqueous extraction dispensable. In fact, the volatility of ketone **S19** and oxime **S20** entails the observed loss of product. Trofimov conditions allowed isolation of the alkene pyrrole **S9** in a good yield of 59% but required tedious purification effort,[1] including distillation and column chromatography to isolate the desired product in reasonable purity. The *anti*-Markovnikov alcohol **32** was lastly obtained in 87% by a Brown hydroboration-oxidation involving the *syn*-addition of 9-borabicyclo(3.3.1)nonane (9-BBN) to the alkene and the subsequent reduction of the intermediate borane with NaOH and hydrogen peroxide.[7-8]

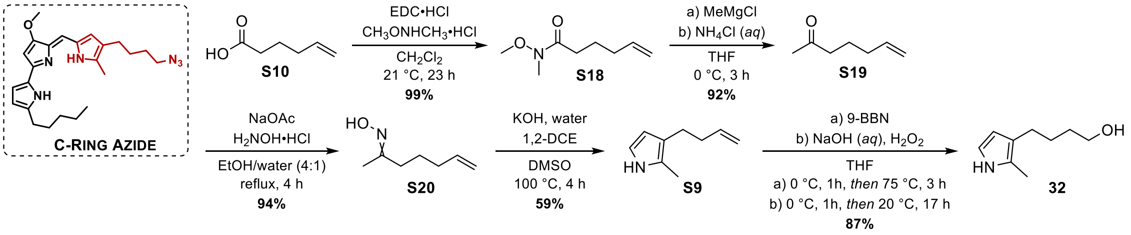

Scheme S5. Alternative route toward the hydroxylated pyrrole **32** from alkene pyrrole **S9** hydroboration. Abbreviations: 9-BBN – 9-borabicyclo(3.3.1)nonane, EDC·HCl – 1-ethyl-3-(3-dimethylaminopropyl)carbodiimide.

Preparation of Succinyl Monomethyl Ester

For acylation of 5-methyl-1*H*-pyrrole-2-carboxylate (**33**) freshly synthesized succinyl monomethyl ester **S34** was prepared by a short sequence, involving ring opening of succinic anhydride (**S11**) through the reaction with anhydrous methanol in quantitative yield, followed by the conversion of the carboxylic acid monomethyl ester **S23** with thionyl chloride into the volatile acid chloride **34** (Scheme S6).[41]

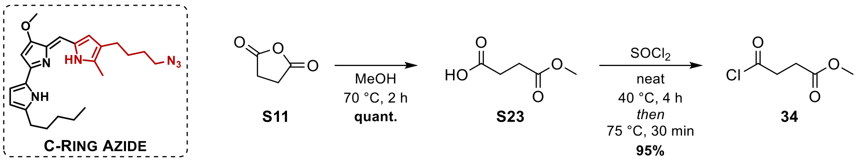

Scheme S6. Preparation of succinyl monomethyl chloride **33** from succinic anhydride **S10**.

Synthesis of the Bifunctional Alkyne-Maleimide Linker

In the first step of the linker synthesis, maleimide (**S24**) was transformed into the methyl carbamate **S25** by reacting with *N*-methyl morpholine (NMM) and methyl chloroformate in EtOAc (Scheme S7).[42] Nucleophilic attack of the carbamate under mild basic conditions in aqueous $NaHCO_3$ solution by *N*-Boc-ethylenediamine (**S26**) allowed ring opening and intramolecular attack of the unprotected diamine nitrogen to form a five-membered carbamate **S27** again and release methyl carbamate from the acyclic intermediate.[43] Standard deprotection of the Boc-protecting group with trifluoracetic acid (TFA) in CH_2Cl_2 provided the ammonium salt **S28** in 98% yield by simple filtration, which was then joined with 5-hexynoic acid in a PyBOP-mediated (benzotriazol-1-yloxytripyrrolidinophosphonium hexafluorophosphate) coupling reaction in an adapted procedure from Buschbeck and Christoffers, originally used with the coupling reagent HATU.[42, 44] The bifunctional linker **42** was synthesized on a gram scale in a yield of 48% over four consecutive steps and was used for further synthetic modification of the prodigiosin azides **2·HCl**, **3·HCl**, and **4·HCl** via CuAAC click chemistry, introducing the protein-conjugable maleimide moiety into the alkaloid structure.

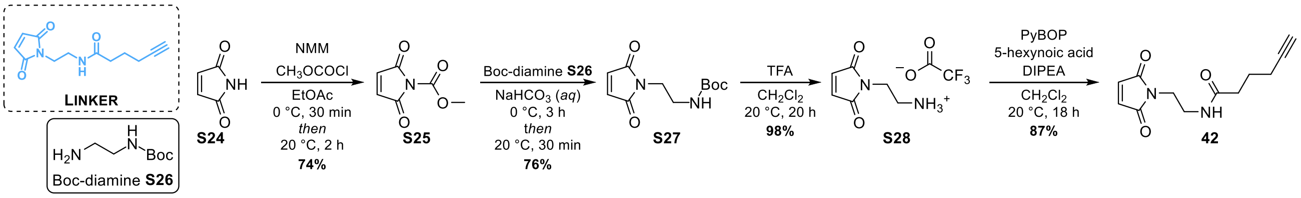

Scheme S7. Synthesis scheme of the bifunctional alkyne-maleimide linker **42** for click-functionalization of prodigiosin azides and conjugation with cysteine-harboring proteins via thiol-Michael addition. Abbreviations: DIPEA – diisopropylethylamine, NMM – *N*-methyl morpholine, PyBOP - benzotriazol-1-yloxytripyrrolidinophosphonium hexafluorophosphate, TFA – trifluoroacetic acid.

Structure of the Tripodal Cu(I)/Cu(II) Ligand TDETA

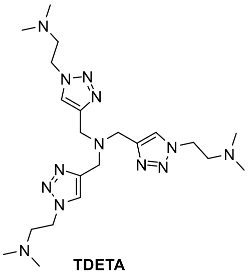

TDETA

Scheme S8. Structure of the Cu(I)/Cu(II) ligand TDETA (**t**ris **d**imethyl**e**thyl **t**riazolyl **a**mine) used for the acceleration of CuAAC click reactions.[45]

Protein-Prodigiosin Conjugates

To briefly showcase the protein-conjugability of the novel prodigiosin maleimides **46**, **47**, and **48** via thiol-Michael chemistry *in vitro*, we chose the protein pyocin S2 from *Pseudomonas aeruginosa* due to its in-house availability. Pyocin S2 itself does not carry cysteine residues needed for site-selective labelling, which is why the surface-accessible cysteine mutation Q570C was introduced by site-directed mutagenesis. After protein purification and reduction of free thiols on the protein surface, the protein was labelled with prodiginine maleimides **46**, **47**, and **48** *in vitro* (Figure S4). After removing excess labeling reagent by desalting, the successful protein conjugation was confirmed by coupled LC-MS measurements (Table S5).

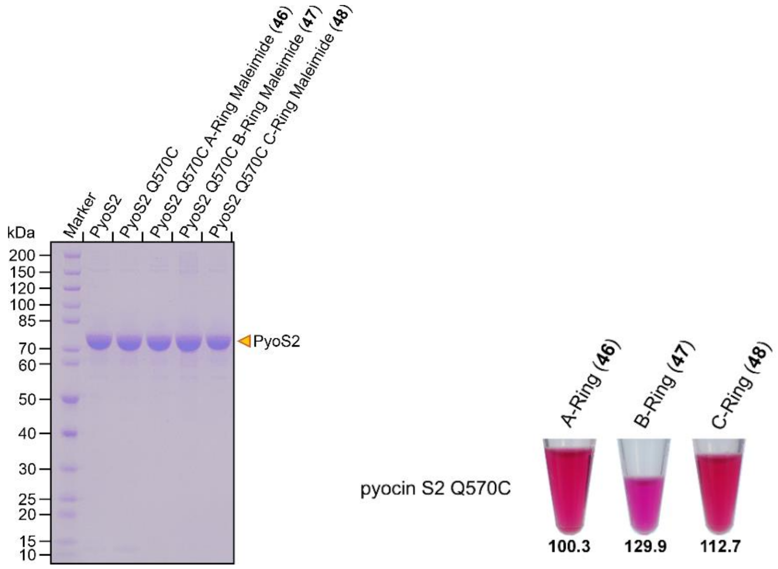

Figure S4. SDS gel of purified pyocins and pyocin-prodigiosin conjugates and photographs of pink protein conjugates after maleimide labeling. The SDS gel shows unlabeled purified pyocin S2 (wild-type), the cysteine mutant Q570C, and the prodigiosin-labeled conjugates of mutant Q570C with prodigiosin maleimides **46**, **47**, and **48**. 2 µg of PyoS2 derivatives were loaded per well. Protein size: PyoS2 – 74 kDa (wild-type). Pink protein solutions (in 25 mM Tris-HCl, 150 mM NaCl, pH 8) indicate labeling of the colorless wild-type protein with pink-purple prodigiosin maleimides. Numbers below the tubes represent protein concentrations (µM), determined with the Pierce 660 nm assay.

During MS measurements, several prodiginine species were detected, corresponding to the masses of the free prodigiosin base, the protonated prodiginine without counter anion, and the prodigiosin HCl salt. Although this LC-MS-based method does not estimate the labeling efficiency and the proportion of labeled and unlabeled protein, it does verify the thiol-Michael addition of pyocin S2 derivatives to the prodigiosin maleimides **46**, **47**, and **48**.

Table S5. Mass spectrometric analysis of pyocin-prodigiosin-conjugates.

Proteins and Protein Conjugates	Label	Average Mass[a],[b] [calc.]	Mass [detect.]	SD
PyoS2	None	73722.52	73722.90	0.88
PyoS2 Q570C	None	73697.55	73697.56	2.06
PyoS2 Q570C conjugate	A-Ring maleimide (**46**)	74338.44[e]	74337.71	0.98
PyoS2 Q570C conjugate	B-Ring maleimide (**47**)	74416.98[c]	74420.01	6.61
PyoS2 Q570C conjugate	C-Ring maleimide (**48**)	74353.47[d]	74355.05	3.43

[a] All proteins and masses were calculated and detected with the initial methionine missing; [b] For PyoS2 conjugates, the average mass was calculated from the sum of the PyoS2 molecular weight (without initial methionine) and masses of the prodigiosin maleimides **46**, **47**, and **48**; [c] Protein + prodigiosin HCl salt; [d] Protein + protonated prodigiosin (no counter ion); [e] Protein + prodigiosin (free base). SD: Standard deviation.

Absorption Spectra and Molar Extinction Coefficients of Unlabeled Prodiginines

Absorption spectra and molar extinction coefficients of the prodigiosin maleimides **46**, **47**, and **48** were measured in acidified EtOH and in aqueous Buffer + 20% EtOH to estimate their absorption behavior in different solvents and at different pH values. Knowledge about these properties is essential to quantify prodiginines and prodiginine conjugates in solution, based on their experimental molar extinction coefficients. All prodiginines exhibited a maximum absorption at 546–548 nm in acidified EtOH and minor absorption at 280 nm (Figure S5, top row). When measuring the same species in aqueous buffer, supplemented with 20% EtOH as co-solvent, the absorption maxima shifted and the absolute absorption decreased by approximately 70%, compared to measurements in acidified EtOH. Concurrently, the relative absorption at 280 nm significantly increased, making UV/Vis-based quantification of both the prodigiosin label and putative proteins of interest challenging. Absorption maxima and extinction coefficients (determined from the slopes of linear regression in Figure S6) are summarized in Table S6.

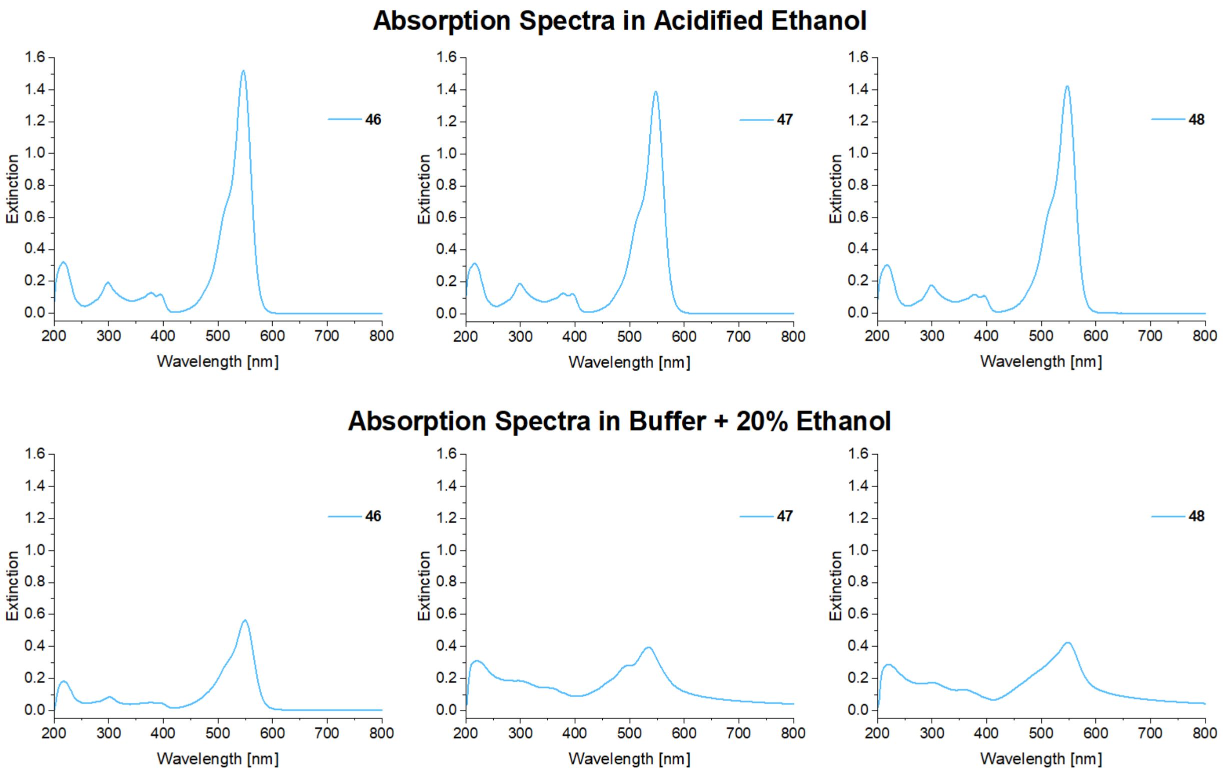

Figure S5. Absorption spectra of prodigiosin maleimides **46**, **47**, and **48**. **Top row**: Spectra of 10 µM dilution in acidified EtOH (+4% 1 M HCl) were recorded from 200–800 nm. **Bottom row**: Spectra of 10 µM dilution in dialysis buffer (25 mM Tris-HCl, 150 mM NaCl, pH 8.0) + 20% (v/v) EtOH were recorded from 200–800 nm. All spectra are plotted in the same y-range for better comparability, clearly highlighting the significant drop in absorption in the aqueous buffer system. Respective absorption maxima and extinction coefficients are summarized in Table S6.

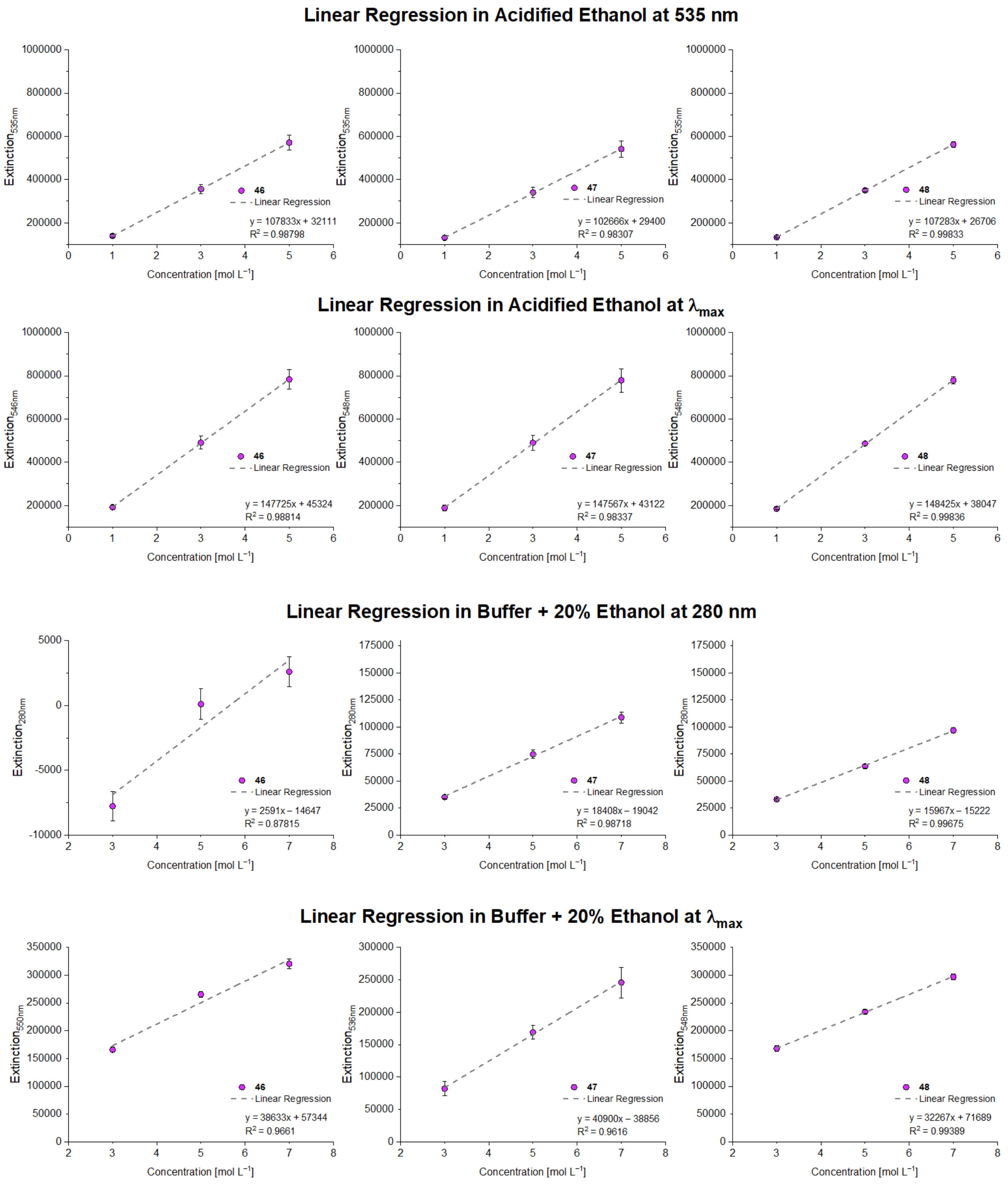

Figure S6. Linear regression fits for determining molar extinction coefficients of prodigiosin maleimides **46**, **47**, and **48**. Shown are the mean values (from triplicate samples) and standard deviations of absorption measurements at various wavelengths, plotted against the molar concentration of the respective prodiginine in acidified EtOH (+4% 1 M HCl) or in dialysis buffer (25 mM Tris-HCl, 150 mM NaCl, pH 8.0) + 20% (v/v) EtOH. According to the Beer-Lambert law, the slope of the linear regression fit equals the molar extinction coefficient. The corresponding absorption maxima and extinction coefficients are summarized in Table S6.

Table S6. Molar extinction coefficients of prodigiosin maleimides **46**, **47**, and **48**.

Acidified Ethanol (+4% 1 M HCl)					
Compound	λ_{max} [nm]	ε_{535} [M^{-1} cm^{-1}]	ε_{546} [M^{-1} cm^{-1}]	ε_{547} [M^{-1} cm^{-1}]	ε_{548} [M^{-1} cm^{-1}]
**46**	546	107833±4496	147725±6117	n.d.	
**47**	548	96400±866	n.d.	n.d.	147567±7254
**48**	548	107283±1656	n.d.	n.d.	148425±2272
**Buffer[a] + 20% Ethanol**					
Compound	λ_{max} [nm]	ε_{280} [M^{-1} cm^{-1}]	ε_{536} [M^{-1} cm^{-1}]	ε_{548} [M^{-1} cm^{-1}]	ε_{550} [M^{-1} cm^{-1}]
**46**	550	2592±365	n.d.	n.d.	38633±2735
**47**	536	18408±793	40900±3089	n.d.	n.d.
**48**	548	15967±344	n.d.	32267±949	n.d.

[a] Dialysis buffer (25 mM Tris-HCl, 150 mM NaCl, pH 8.0); n.d.: not determined. Molar extinction coefficients ε were determined from the slope of a linear regression fit. All samples were measured in triplicate. Mean values and standard deviations are given.

Absorption and Fluorescence Spectra of *F*-BODIPY A-Ring Azide 49

When the purple powder of *F*-BODIPY **49** was dissolved in organic solvents, bright-red fluorescent solutions were obtained. The absorption maxima ranged from 582 nm (EtOH) to 594 nm (CH_2Cl_2), while fluorescence emission occurred from 588–603 nm (Figure S7). The apolar solvent *n*-heptane (incl. 1% CH_2Cl_2 for solubility) provided the largest Stokes shift of 14 nm, however, the Stokes shift in general was relatively small, ranging from 5–14 nm. Full absorption and fluorescence emission spectra in CH_2Cl_2, EtOH, THF, and *n*-heptane/CH_2Cl_2 (99:1) are presented (Figure S7).

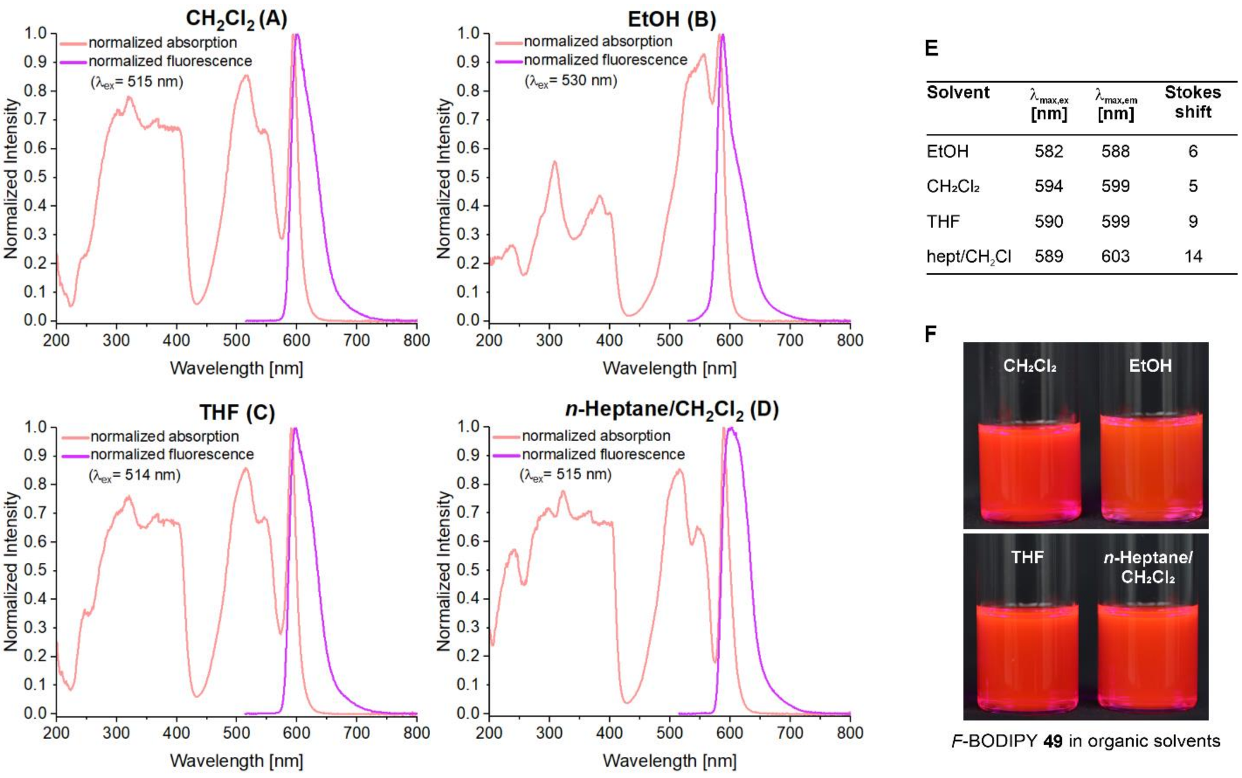

Figure S7. Normalized absorption and fluorescence spectra of the bright-red fluorescent *F*-BODIPY A-ring azide **49** in various organic solvents. The BODIPY was dissolved in CH_2Cl_2, EtOH, THF, and *n*-heptane/CH_2Cl_2 (99:1) at 80 μM and absorption spectra (orange) were measured in a range from 200–800 nm. Fluorescence emission (pink) was measured after excitation at 514 nm (THF), 515 nm (CH_2Cl_2 and *n*-heptane/CH_2Cl_2), or 530 nm (EtOH). All spectra were corrected for the solvent background signal. The photograph shows the red fluorescence of *F*-BODIPY **49** in various organic solvents at 80 μM under exposure to sunlight and the

adjacent table summarizes the maximum wavelengths of absorption ($\lambda_{max,ex}$) and fluorescence emission ($\lambda_{max,em}$), and the corresponding Stokes shift in these solvents.

Synthetic Procedures

General information

All commercial chemicals were purchased from Sigma Aldrich (Merck), Tokyo Chemical Industry (TCI), ThermoScientific, VWR, BLDpharm, Fluorochem, Biosynth, and Th. Geyer in purities >97%. Purification of technical-grade organic solvents from VWR, Carl Roth, or Fisher Chemical was achieved by distillation. Anhydrous toluene, THF, Et_2O, and CH_2Cl_2 were obtained from the MBraun MB-SPS 800 solvent purification system. Other solvents (*i*PrOH, MeOH, EtOAc, DMSO, DMF, and 1,2-dichloroethane) were dried over activated molecular sieve for >72 h. Dry glyme (99.5+%, extra dry over molecular sieve) was purchased from Thermo Scientific Chemicals. Degreased cotton wool (Brand) for filtration purposes was again degreased by washing with distilled *n*-pentane, petroleum ether, or CH_2Cl_2. Reactions under inert conditions were performed under N_2 atmosphere with pre-dried glassware under the standard laboratory Schlenk technique. If stated in the synthetic instructions, solvents were degassed using the freeze-pump-thaw procedure (3x). 2,2,6,6-Tetramethylpiperidine (TMP) was refluxed for 4 h over CaH_2 and then distilled under normal pressure and stored over 4 Å molecular sieve. All synthesized compounds were stored under Ar atmosphere at −20 °C. ^{1}H, ^{11}B, ^{13}C, ^{19}F, DEPT135, ^{1}H-^{1}H-COSY, ^{1}H-^{13}C-HSQC, ^{1}H-^{13}C-HMBC, ^{1}H-^{1}H-ROESY, and ^{1}H-^{1}H-TOCSY NMR data were collected on a Bruker Avance/DRX 600 or Avance/DRX 300 spectrometer. For this purpose, $CDCl_3$, DMSO-*d*$_6$, CD_2Cl_2, or MeOD were used as solvents and their respective chemical shifts ($CDCl_3$: ^{1}H – 7.26 ppm, ^{13}C – 77.16 ppm; DMSO-*d*$_6$: ^{1}H – 2.50 ppm, ^{13}C – 39.52 ppm, CD_2Cl_2: ^{1}H – 5.32 ppm, ^{13}C – 53.84 ppm; MeOD: ^{1}H – 3.31 ppm, ^{13}C – 49.00 ppm) used for referencing.[38] The atom numbers in NMR assignments refer to the corresponding atom numbering of the structures within the NMR spectra (supporting information). High-resolution mass spectrometry (HRMS) data were recorded on a Bruker maXis instrument (at CeMSA in Düsseldorf) with a standard collision energy of 8 eV. However, for Boc-protected 1*H*-pyrroles, a reduction to 2–4 eV was obligatory, as Boc-protecting groups showed fragmentation towards the carbamic acid $[M{-}56.0626]^+$ at 8 eV. For melting point determination, the Büchi melting point apparatus B-540 was employed with a temperature increment of 2 °C/min. Thin layer chromatography (TLC) was performed using coated Macherey-Nagel POLYGRAM SIL G/UV_{254} plates (40 x 80 x 0.2 mm) and for standard preparative flash chromatography Macherey-Nagel silica gel 60 (40–63 µm, 230–400 mesh) or ACROS Organics neutral aluminium oxide (Brockmann I, 40–300 µm) was used. Dry column vacuum chromatography (DCVC) was performed using Merck silica gel 60 (15–40 µm). Visualization of different compounds on TLC plates was achieved using various staining methods, including visible light, excitation under UV light, $KMnO_4$ stain (7.5 g L^{-1} $KMnO_4$, 50 g L^{-1} K_2CO_3, 625 mg L^{-1} NaOH in water), or *p*-anisaldehyde stain (300 mL glacial acetic acid, 6 mL conc. H_2SO_4, 3 mL *p*-anisaldehyde). The copper ligand TDETA (Scheme S8, supporting information) was synthesized as published earlier.[45]

2-Methyl-3-propyl-1*H*-pyrrole (5)

(*E*/*Z*)-Hexan-2-one oxime (**6**, 12.0 g, 104 mmol, 1.00 eq.), pestled KOH (29.2 g, 521 mmol, 5.00 eq.), and water (1.43 mL, 13.7 µL $mmol^{-1}$ oxime) were sequentially added to a three-necked flask under N_2 atmosphere. Degassed and dried DMSO (200 mL, 1.92 mL $mmol^{-1}$ oxime) was added under N_2 atmosphere. The reaction was refluxed at 100 °C and a solution of 1,2-dichloroethane (28.80 mL, 365 mmol, 3.50 eq.) in degassed DMSO (22.0 mL, 0.21 mL $mmol^{-1}$ oxime) was added with a syringe pump over 2 h. A second batch of KOH (29.2 g, 521 mmol, 5.00 eq.) was added under ice cooling after the first hour of 1,2-dichloroethane addition (dichloroethane-feeding is paused during KOH addition) before the dichloroethane addition was continued for a further 1 h. Once the addition of dichloroethane was completed, the reaction was refluxed for 2 h. The reaction was cooled to 0 °C and ice water was added for quenching. Extraction was performed with Et_2O (3 x 100 mL). Combined organic phases were dried over $MgSO_4$, filtered over degreased cotton wool and the solvent was removed *in vacuo*. Chromatographic purification on silica with PE/CH_2Cl_2 (85:15) + 1% triethylamine (TEA) provided the product as yellow oil (6.99 g, 56.7 mmol, 54%). R_f (PE/EtOAc 80:20): 0.57 (UV and $KMnO_4$). ^{1}H NMR (600 MHz, $CDCl_3$, δ): 7.70 (s, 1H, 1-H), 6.60 (t, *J*=2.7, 1H, 5-H), 6.02 (t, *J*=2.7, 1H, 4-H), 2.37 (t, *J*=7.5, 2H, 7-H), 2.19 (s, 3H, 6-H), 1.56 (h, *J*=7.5, 2H, 8-H), 0.95 (t, *J*=7.3, 3H, 9-H). ^{13}C NMR (151 MHz, $CDCl_3$, δ): 123.4 (C-2), 119.7 (C-3), 114.9 (C-5), 109.0 (C-4), 28.2 (C-7), 24.6 (C-8), 14.2 (C-9), 11.2 (C-6). FT-IR (neat, cm^{-1}): 3378, 2956, 2925, 2869, 1464, 1455, 1376, 1249, 1106, 1066, 955, 904, 889, 832, 801, 712, 663, 549. HRMS (ESI) *m/z:* $[M + H]^+$ calcd for $C_8H_{14}N$, 124.1121; found, 124.1120.

5-Methyl-4-propyl-1*H*-pyrrole-2-carbaldehyde (7)

To a 250 mL Schlenk flask with stirring bar under N_2 atmosphere was added dry DMF (2.10 mL, 27.3 mmol, 1.20 eq.) and cooled to 0 °C. Phosphoryl chloride (2.54 mL, 27.3 mmol, 1.20 eq.) was added dropwise at 0 °C and the Vilsmeier reagent solidified as a white

clump. The reagent was suspended in dry 1,2-dichloroethane (6.8 mL, 0.3 mL $mmol^{-1}$ pyrrole) and stirred for 30 min at 0 °C. A solution of 2-methyl-3-propyl-1*H*-pyrrole (**5**, 2.80 g, 22.7 mmol, 1.00 eq.) in 1,2-dichloroethane (13.6 mL, 0.6 mL $mmol^{-1}$ pyrrole) was added dropwise and the reaction mixture was refluxed for 2 h after completion of the addition. A solution of sodium acetate (11.2 g, 136 mmol, 6.00 eq.) in water (34.1 mL, 4 mol L^{-1}) was slowly added and the mixture was refluxed for 30 min. The reaction was cooled to ambient temperature and extracted with CH_2Cl_2 (4 x 100 mL). Merged organic phases were dried over Na_2SO_4, filtered and the solvent removed *in vacuo* to yield a brown residue. Chromatographic purification on silica with PE/CH_2Cl_2 (92:8) + 1% TEA provided the product (3.29 g, 21.8 mmol, 96%) as a light-yellow crystalline solid. R_f (PE/EtOAc 80:20): 0.28 (UV and $KMnO_4$). Mp 74.8–76.4 °C. ^{1}H NMR (600 MHz, $CDCl_3$, δ): 9.78 (s, 1H, H-1), 9.30 (s, 1H, H-6), 6.75 (d, *J*=2.6, 1H, 3-H), 2.37 (t, *J*=7.6, 2H, 8-H), 2.28 (s, 3H, 11-H), 1.56 (h, *J*=7.4, 2H, 9-H), 0.94 (t, *J*=7.3, 3H, 10-H). ^{13}C NMR (151 MHz, $CDCl_3$, δ): 177.7 (C-6), 135.9 (C-5), 130.6 (C-2), 124.5 (C-4), 122.6 (C-3), 27.8 (C-8), 23.9 (C-9), 14.0 (C-10), 11.7 (C-11). FT-IR (neat, cm^{-1}): 3166, 3091, 3062, 2961, 2932, 2869, 2844, 1622, 1615, 1514, 1464, 1414, 1378, 1342, 1276, 1157, 1143, 1050, 839, 812, 741, 730, 673, 635, 625, 518. HRMS (ESI) *m/z:* [M + H]$^+$ calcd for $C_9H_{14}NO$, 152.1070; found, 152.1073.

(*Z*)-4-Methoxy-5-((5-methyl-4-propyl-1*H*-pyrrol-2-yl)methylene)-1,5-dihydro-2*H*-pyrrol-2-one (9)

To a 500 mL Schlenk flask under N_2 atmosphere were added 5-methyl-4-propyl-1*H*-pyrrole-2-carbaldehyde (**7**, 4.00 g, 26.5 mmol, 1.00 eq.) and 4-methoxy-3-pyrrolin-2-one (**8**, 5.98 g, 52.9 mmol, 2.00 eq.) and dissolved in degassed DMSO (92.6 mL, 3.5 mL $mmol^{-1}$ carbaldehyde). Degassed 2 M NaOH (*aq.*) (74.1 mL, 2.8 mL $mmol^{-1}$ carbaldehyde) was added and the reaction was stirred for 24 h at 60 °C. A yellow solid precipitated during the reaction. The product was partitioned between EtOAc (150 mL) and water (150 mL), the phases were separated, and the aqueous phase was subsequently extracted with EtOAc (5 x 100 mL). Merged organic phases were washed with water (100 mL) and brine (150 mL), dried over Na_2SO_4, filtered, and the solvent was removed *in vacuo*. The canary yellow crude product was then suspended in *n*-heptane and filtered. The filter cake was washed repeatedly with *n*-heptane, dropwise with *i*Pr_2O/acetone (1:1), and again with *n*-heptane to remove a brown impurity. After drying under high vacuum, the desired product (5.96 g, 24.2 mmol, 91%) was obtained as gold-yellow powder. Mp 195.1 °C (Decomposition). ^{1}H NMR (600 MHz, $CDCl_3$, δ): 10.87 (brs, 1H, 1-H), 10.30 (brs, 1H, 8-H), 6.28 (s, 1H, 6-H), 6.24 (d, *J*=2.5, 1H, 11-H), 5.09 (d, *J*=1.1, 1H, 3-H), 3.88 (s, 3H, 14-H), 2.35 (t, *J*=7.6, 2H, 16-H), 2.34 (s, 3H, 15-H), 1.55 (h, *J*=7.4, 2H, 17-H), 0.94 (t, *J*=7.3, 3H, 18-H). ^{13}C NMR (151 MHz, $CDCl_3$, δ): 173.2 (C-2), 168.0 (C-4), 132.5 (C-9), 124.3 (C-7), 122.6 (C-5), 122.5 (C-10), 118.0 (C-11), 102.9 (C-6), 90.0 (C-3), 58.3 (C-14), 27.8 (C-16), 24.2 (C-17), 14.1 (C-18), 11.5 (C-15). FT-IR (neat, cm^{-1}): 3345, 3144, 2952, 2918, 1680, 1643, 1574, 1484, 1438, 1384, 1347, 1219, 1131, 1007, 976, 870, 802, 768, 704, 680, 642, 630, 546, 474, 467. HRMS (ESI) *m/z:* [M + H]$^+$ calcd for $C_{14}H_{19}N_2O_2$, 247.1441; found, 247.1447.

(*Z*)-4-Methoxy-5-((5-methyl-4-propyl-2*H*-pyrrol-2-ylidene)methyl)-1*H*-pyrrol-2-yl trifluoromethanesulfonate (10)

In a 250 mL Schlenk flask under N_2 atmosphere, trifluoromethanesulfonic anhydride (984 µL, 5.85 mmol, 1.20 eq., freshly opened**!**) was added dropwise to a solution of dipyrrinone **9** (1.20 g, 4.87 mmol, 1.00 eq.) in CH_2Cl_2 (95 mL, 19.5 mL $mmol^{-1}$) at 0 °C. After stirring at this temperature for 1 h, the dark brown reaction mixture was poured into aqueous 2% $NaHCO_3$ (250 mL), the phases were separated and the aqueous phase was repeatedly extracted with MTBE (3 x 20 mL). The combined organic layers were washed with brine (200 mL), dried over Na_2SO_4, filtered, and the solvent was evaporated *in vacuo*. Before complete removal of the solvent, *n*-pentane was repeatedly added to assist in the precipitation of the product. Thorough solvent removal provided the pure triflate (1.75 g, 4.62 mmol, 95%) without requiring purification as a deep brown-yellow amorphous solid. Mp 67.4–68.7 °C. ^{1}H NMR (300 MHz, $CDCl_3$, δ): 10.70 (brs, 1H, 1-H), 6.97 (s, 1H, 6-H), 6.52 (s, 1H, 11-H), 5.41 (s, 1H, 3-H), 3.86 (s, 3H, 18-H), 2.35 (t, *J*=7.5, 2H, 20-H), 2.30 (s, 3H, 19-H), 1.55 (h, *J*=7.4, 2H, 21-H), 0.93 (td, *J*=7.3, 2.9, 3H, 22-H). ^{13}C NMR (76 MHz, $CDCl_3$, δ): 167.6 (C-4), 160.4 (C-2), 138.6 (C-9), 131.5 (C-5), 127.5 (C-7), 125.7 (C-10), 123.3 (C-11), 122.0 (C-6), 118.8 (q, *J*=321.0, C-16), 86.9 (C-3), 58.8 (C-18), 27.8 (C-20), 23.9 (C-21), 13.9 (C-22), 12.3 (C-19). ^{19}F NMR (282 MHz, $CDCl_3$, δ): –72.71 (s, –C*F*$_3$). FT-IR (neat, cm^{-1}): 3347, 2958, 2935, 2873, 1619, 1544, 1505, 1474, 1451, 1416, 1371, 1332, 1260, 1234, 1210, 1147, 1126, 1085, 1053, 1007, 970, 926, 887, 821, 797, 766, 747, 734, 713, 690, 650, 637, 621, 600, 571, 543, 519, 483. HRMS (ESI) *m/z:* [M + H]$^+$ calcd for $C_{15}H_{18}F_3N_2O_4S$, 379.0934; found, 379.0938.

Ethyl 5-oxo-5-(1*H*-pyrrol-2-yl)pentanoate (12)

In a 2 L two-necked flask under N_2 atmosphere, aluminium(III) chloride (22.4 g, 168 mmol, 1.20 eq.) was added to dry CH_2Cl_2 (1 L) and cooled to 0 °C. Ethyl glutaryl chloride (**11**, 21.9 mL, 140 mmol, 1.00 eq.) was added slowly to the transparent suspension, and the mixture was stirred for 30 min at 0 °C until the salt had completely dissolved. A solution of 1*H*-pyrrole (10.7 mL, 154 mmol, 1.10 eq.) in dry CH_2Cl_2 (70 mL) was transferred to the acid chloride solution, causing a color shift from yellow to orange. The reaction was stirred for a further 3 h at 0 °C, then thawed to 20 °C overnight and stirred at this temperature for 15 h, yielding a deep orange/brown colored solution. At 0 °C, saturated NH_4Cl (*aq.*) was slowly introduced for reaction quenching. The phases were separated and the aqueous

phase was subsequently extracted with CH_2Cl_2 (3 x 100 mL). The organic phases were merged, washed with saturated $NaHCO_3$ (2 x 250 mL) and brine (1 x 250 mL), dried over $MgSO_4$, filtered, and the solvent removed *in vacuo*. The brown oily residue was dissolved in EtOAc, immobilized on Celite by evaporation and applied to dry column vacuum chromatography (8 cm bed height, 9 cm column diameter, DCVC) on Merck silica 60 (15–40 µm) with a PE/MTBE gradient (0 to 80% MTBE with 3% increment per fraction and 100 mL fraction volume) to afford the product (20.9 g, 100 mmol, 72%) as orange oil. R_f (PE/MTBE 60:40): 0.32 ($KMnO_4$). ^1H NMR (300 MHz, $CDCl_3$, δ): 9.88 (brs, 1H, 1-H), 7.04 (td, *J*=2.7, 1.3, 1H, 5-H), 6.93 (ddd, *J*=3.8, 2.4, 1.3, 1H, 3-H), 6.26 (dt, *J*=3.8, 2.5, 1H, 4-H), 4.13 (q, *J*=7.1, 2H, 14-H), 2.84 (t, *J*=7.3, 2H, 8-H), 2.40 (t, *J*=7.3, 2H, 10-H), 2.05 (p, *J*=7.3, 2H, 9-H), 1.24 (t, *J*=7.1, 3H, 15-H). ^{13}C NMR (76 MHz, $CDCl_3$, δ): 190.1 (C-6), 173.4 (C-11), 132.0 (C-2), 124.9 (C-5), 116.5 (C-3), 110.7 (C-4), 60.5 (C-14), 36.9 (C-8), 33.7 (C-10), 20.3 (C-9), 14.3 (C-15). FT-IR (neat, cm^{-1}): 3284, 2978, 2939, 2906, 2875, 1729, 1635, 1546, 1403, 1375, 1302, 1245, 1212, 1185, 1159, 1138, 1111, 1043, 934, 914, 882, 873, 853, 843, 753, 606, 515, 505. HRMS (ESI) *m/z:* $[M + H]^+$ calcd for $C_{11}H_{16}NO_3$, 210.1125; found, 210.1127.

5-(1*H*-Pyrrol-2-yl)pentan-1-ol (13)

Highly optimized procedure: To a 1 L three-necked flask with reflux condenser under N_2 atmosphere was added the pyrrole ethyl ester (**12**, 20.3 g, 97.0 mmol, 1.00 eq.) and was dissolved in dry 1,2-dimethoxy ethane (388 mL, 4 mL $mmol^{-1}$ pyrrole). The orange solution was degassed by consecutive freeze-pump-thaw cycles (3x) and finally cooled to 0 °C for 20 min. Under a slow stream of N_2, $LiAlH_4$ (18.4 g, 485 mmol, 5.00 eq.) was added carefully in a single batch (vigorous gas and local heat development) and the solution stirred for 5 min at 0 °C (gas development had stopped). Afterwards, the slurry was refluxed at 90 °C for 90 min (preheated heating block). For the workup procedure according to Fieser,[9] the reaction mixture was cooled to 0 °C for 10 min before 18.4 mL of ice water (1 mL g^{-1} $LiAlH_4$) were added dropwise to the well-ventilated solution (condenser still in place! If the stirring bar starts to get clogged, the solution is diluted with Et_2O). After vigorous stirring for 15 min, 18.4 mL of cold 15% (w/v) NaOH (1 mL g^{-1} $LiAlH_4$) was slowly added, followed by 55.2 mL of ice water (3 mL g^{-1} $LiAlH_4$). The solution was stirred for 15 min at 0 °C and then for a further 30 min at 20 °C to give a pale-yellow slurry with fine white precipitate. The precipitated aluminium salts were filtered off over a fritted filter (P3) and thoroughly suspended in MTBE, followed by several washing steps with MTBE. Brine (500 mL) was added to the merged clear yellow filtrate and wash fractions, the phases were separated and the aqueous phase was then extracted with MTBE (2 x 150 mL). Merged organic phases were dried over $MgSO_4$, filtered, and the solvent removed *in vacuo*. The viscous yellow oil was dissolved in MTBE and immobilized on Celite by careful evaporation of the solvent. The solid was applied to DCVC (7 cm bed height, 8 cm column diameter, column equilibrated in PE + 1.5% TEA) on Merck silica 60 (15–40 µm) with a PE/MTBE gradient (0 to 70% MTBE with 4.5% increment per fraction, then constant elution at PE/MTBE 30:70 over 15 fractions and 8 fractions with PE/EtOAc 20:80, 100 mL fraction size volume) to afford the product (13.7 g, 89.4 mmol, 92%) as viscous yellow. R_f (PE/MTBE 10:90): 0.53 ($KMnO_4$). R_f (PE/MTBE 40:60): 0.24 ($KMnO_4$). ^1H NMR (300 MHz, $CDCl_3$, δ): 8.08 (brs, 1H, 1-H), 6.67 (td, *J*=2.6, 1.5, 1H, 5-H), 6.14 (q, *J*=2.9, 1H, 4-H), 5.95 – 5.90 (m, 1H, 3-H), 3.65 (td, *J*=6.8, 2.1, 2H, 10-H), 2.62 (t, *J*=7.6, 2H, 6-H), 1.73 – 1.54 (m, 5H, 7-H, 9-H, 11-OH), 1.50 – 1.36 (m, 2H, 8-H). ^{13}C NMR (76 MHz, $CDCl_3$, δ): 132.6 (C-2), 116.2 (C-5), 108.3 (C-4), 105.0 (C-3), 62.9 (C-10), 32.5 (C-9), 29.5 (C-7), 27.7 (C-6), 25.5 (C-8). FT-IR (neat, cm^{-1}): 3370, 3145, 3098, 2931, 2857, 1568, 1472, 1462, 1429, 1403, 1349, 1311, 1302, 1276, 1259, 1228, 1118, 1096, 1065, 1048, 1025, 958, 884, 787, 718, 607, 582, 570. HRMS (ESI) *m/z:* $[M + H]^+$ calcd for $C_9H_{16}NO$, 154.1226; found, 154.1229.

5-(1*H*-Pyrrol-2-yl)pentyl 4-methylbenzenesulfonate (14)

In a 500 mL Schlenk flask under N_2 atmosphere, pyrrole (**13**, 7.59 g, 49.5 mmol, 1.00 eq.) and DMAP (605 mg, 4.95 mmol, 0.10 eq.) were dissolved in dry CH_2Cl_2 (165 mL, 3.34 mL $mmol^{-1}$ pyrrole) and DIPEA (25.4 mL, 149 mmol, 3.00 eq.) was added under cooling to 0 °C. At 0 °C, a solution of recrystallized *p*TsCl (14.2 g, 74.3 mmol, 1.50 eq.) in dry CH_2Cl_2 (83 mL) was slowly added to the pyrrolic solution and the solution was allowed to warm to ambient temperature. The reaction was stirred for 16.5 h at 26 °C. Ice water (250 mL) was added for quenching, the phases were separated, and the aqueous phase was extracted with CH_2Cl_2 (3 x 50 mL). Merged organic phases were dried over $MgSO_4$, filtered, and the solvent was removed *in vacuo*. Column chromatography on silica using a PE/CH_2Cl_2 gradient (70:30, then 60:40, then 30:70) as eluent provided the product (10.1 g, 32.9 mmol, 66%) as light-yellow oil. Considering re-isolation of unconverted pyrrole (1.04 g, 6.81 mmol), a total yield of 77% was achieved. R_f (PE/MTBE 40:60): 0.48 ($KMnO_4$). ^1H NMR (600 MHz, $CDCl_3$, δ): 7.97 (brs, 1H, 1-H), 7.78 (d, *J*=8.4, 2H, 16-H, 20-H), 7.35 (d, *J*=8.1, 2H, 17-H, 19-H), 6.66 (td, *J*=2.6, 1.5, 1H, 5-H), 6.12 (q, *J*=2.9, 1H, 4-H), 5.87 (d, *J*=3.0, 1H, 3-H), 4.02 (t, *J*=6.4, 2H, 10-H), 2.56 (t, *J*=7.6, 2H, 6-H), 2.45 (s, 3H, 21-H), 1.71 – 1.63 (m, 2H, 9-H), 1.58 (p, *J*=7.7, 2H, 7-H), 1.38 (dp, *J*=7.5, 6.3, 2H, 8-H). ^{13}C NMR (151 MHz, $CDCl_3$, δ): 144.8 (C-18), 133.6 (C-15), 132.2 (C-2), 130.0 (C-17, C-19), 128.0 (C-16, C-20), 116.3 (C-5), 108.4 (C-4), 105.2 (C-3), 70.6 (C-10), 29.1 (C-7), 28.8 (C-9), 27.6 (C-6), 25.2 (C-8), 21.7 (C-21). FT-IR (neat, cm^{-1}): 3409, 2933, 2859, 1597, 1570, 1495, 1463, 1350, 1306, 1292, 1211, 1188, 1173, 1120, 1097, 1021, 943, 904, 884, 814, 782, 713, 691, 662, 574, 553, 496. HRMS (ESI) *m/z:* $[M + H]^+$ calcd for $C_{16}H_{22}NO_3S$, 308.1315; found, 308.1318.

tert-Butyl 2-(5-(tosyloxy)pentyl)-1H-pyrrole-1-carboxylate (15)

To a 250 mL Schlenk flask under N_2 atmosphere were added 5-(1*H*-pyrrol-2-yl)pentyl 4-methylbenzenesulfonate (**14**, 9.24 g, 30.1 mmol, 1.00 eq.) and DMAP (367 mg, 3.01 mmol, 0.10 eq.) and dissolved in dry CH_2Cl_2 (25 mL). A solution of Boc_2O (7.87 g, 36.1 mmol, 1.20 eq.) in CH_2Cl_2 (100 mL) was added to the prior solution at 31 °C and the reaction was stirred for 22 h at 31 °C. The solvent was removed under reduced pressure and the orange oily residue purified by column chromatography on silica using a gradient of PE/CH_2Cl_2 (60:40 up to 20:80). The product was afforded as a colorless viscous oil (9.93 g, 24.4 mmol, 81%). R_f (PE/MTBE 70:30): 0.52 (UV and $KMnO_4$). ^{1}H NMR (300 MHz, $CDCl_3$, δ): 7.81 – 7.75 (m_c, 2H, 23-H, 27-H), 7.37 – 7.30 (m_c, 2H, 24-H, 26-H), 7.17 (dd, *J*=3.4, 1.8, 1H, 5-H), 6.05 (t, *J*=3.3, 1H, 4-H), 5.89 (ddt, *J*=3.4, 1.9, 1H, 3-H), 4.02 (t, *J*=6.5, 2H, 17-H), 2.78 (t, 2H, 13-H), 2.44 (s, 3H, 28-H), 1.74 – 1.62 (m, 2H, 16-H), 1.61 – 1.48 (m, 11H, 10-H, 11-H, 12-H, 14-H), 1.45 – 1.31 (m, 2H, 15-H). ^{13}C NMR (76 MHz, $CDCl_3$, δ): 149.5 (C-6), 144.8 (C-25), 135.9 (C-2), 133.3 (C-22), 129.9 (C-24, C-26), 128.0 (C-23, C-27), 120.9 (C-5), 111.0 (C-3), 109.9 (C-4), 83.4 (C-9), 70.6 (C-17), 28.8 (C-16), 28.6 (C-13), 28.2 (C-14), 28.1 (C-10, C-11, C-12), 25.2 (C-15), 21.7 (C-28). FT-IR (neat, cm^{-1}): 2977, 2935, 2864, 1737, 1598, 1495, 1477, 1458, 1407, 1396, 1367, 1359, 1329, 1255, 1235, 1210, 1188, 1176, 1127, 1098, 1060, 1019, 1012, 950, 906, 882, 842, 829, 815, 773, 759, 727, 689, 664, 600, 577, 555, 493. HRMS (ESI) *m/z:* $[M + H]^+$ calcd for $C_{21}H_{30}NO_5S$, 408.1839; found, 408.1845.

tert-Butyl 2-(5-azidopentyl)-1H-pyrrole-1-carboxylate (16)

In a 100 mL Schlenk flask under N_2 atmosphere, the tosyl-protected 4-(2-methyl-1*H*-pyrrol-3-yl)butyl 4-methylbezenesulfonate (**15**, 8.75 g, 21.5 mmol, 1.00 eq.) was dissolved in dry DMF (85.8 mL, 4 mL $mmol^{-1}$ pyrrole). Sodium azide (4.19 g, 64.4 mmol, 3.00 eq.) was added and the reaction was furnished by adding five catalytic drops of DIPEA. The mixture was stirred for 17 h at 21 °C, after which time a white solid had precipitated. The reaction was quenched by the addition of ice water (50 mL) and the product was afterwards extracted with Et_2O (5 x 50 mL). Merged organic phases were washed with water (2 x 100 mL), dried over $MgSO_4$, filtered, and the solvent was removed *in vacuo* to give a yellow residue. Purification of the crude product by column chromatography on silica with *n*-pentane/CH_2Cl_2 (80:20) yielded the desired product as a colorless oil (5.94 g, 21.3 mmol, 99%). R_f (*n*-pentane/CH_2Cl_2 60:40): 0.36 (UV and $KMnO_4$). ^{1}H NMR (600 MHz, $CDCl_3$, δ): 7.18 (dd, *J*=3.4, 1.8, 1H, 5-H), 6.07 (t, *J*=3.3, 1H, 4-H), 5.95 (ddt, *J*=3.4, 1.8, 1H, 3-H), 3.27 (t, *J*=7.1, 2H, 17-H), 2.86 (t, *J*=7.6, 2H, 13-H), 1.65 (pd, *J*=7.4, 4H, 14-H, 16-H), 1.59 (s, 9H, 10-H, 11-H, 12-H), 1.49 – 1.42 (m, 2H, 15-H). ^{13}C NMR (151 MHz, $CDCl_3$, δ): 149.6 (C-6), 136.1 (C-2), 121.0 (C-5), 111.0 (C-3), 110.0 (C-4), 83.4 (C-9), 51.6 (C-17), 28.9 (C-13), 28.8 (C-16), 28.6 (C-14), 28.2 (C-10, C-11, C-12), 26.6 (C-15). FT-IR (neat, cm^{-1}): 3159, 3109, 2979, 2935, 2862, 2093, 1738, 1495, 1478, 1457, 1408, 1395, 1370, 1329, 1319, 1255, 1236, 1169, 1156, 1125, 1059, 1011, 883, 851, 804, 772, 721, 674, 599, 558, 495, 463. HRMS (ESI) *m/z:* $[M + H]^+$ calcd for $C_{14}H_{23}N_4O_2$, 279.1816; found, 279.1814.

(5-(5-Azidopentyl)-1-(tert-butoxycarbonyl)-1H-pyrrol-2-yl)boronic acid (17)

In a 100 mL Schlenk flask under N_2 atmosphere, TMP (7.02 mL, 41.6 mmol, 2.00 eq.) was dissolved in dry THF (49.1 mL, 2.36 mL $mmol^{-1}$ pyrrole) and cooled to −78 °C. *n*-Butyl lithium (17.8 mL, 43.7 mmol, 2.10 eq., 2.46 M in *n*-hexane) was added dropwise to the TMP solution in THF at 0.5 mL min^{-1} (syringe pump). After completion of the addition and further stirring for 30 min at −78 °C, the mixture was allowed to warm to 0 °C over 30 min and was then cooled again to −78 °C. In a 500 mL three-necked flask under N_2 atmosphere, equipped with cold thermometer and connection to the line, was *tert*-butyl 2-(5-azidopentyl)-1*H*-pyrrole-1-carboxylate (**16**, 5.79 g, 20.8 mmol, 1.00 eq.) dissolved in dry THF (83.2 mL, 4.00 mL $mmol^{-1}$ pyrrole) and cooled to −78 °C. The LiTMP was then added dropwise with a transfer cannula to the glass surface of the flask with the pyrrolic THF solution at the slowest possible speed by adding positive pressure of argon to the Schlenk flask. The internal temperature was constantly kept below −70 °C and the rate was adjusted if needed. The reaction mixture was stirred for 2 h at −78 °C, before a solution of $B(OMe)_3$ (6.96 mL, 62.4 mmol, 3.00 eq.) in dry THF (22.3 mL, 1.07 mL $mmol^{-1}$ pyrrole) was added dropwise at 1.00 mL min^{-1} (syringe pump). The solution was stirred for 1 h at −78 °C, before excess dry ice was removed from the cooling bath. The reaction was allowed to warm to 21 °C over 15 h. The reaction was quenched at 0 °C by the dropwise addition of 0.20 M HCl (260 mL, 52.0 mmol, 2.50 eq.). Phases were separated and the aqueous phase was extracted with Et_2O (3 x 150 mL). Merged organic phases were washed with water (2 x 100 mL) and brine (2 x 100 mL), dried over a minimal amount $MgSO_4$, filtered, and the solvent was removed *in vacuo*. As the product typically did not precipitate upon removal of the ethereal solvent, the residue was taken up in Et_2O and washed again with 0.2 M HCl (2 x 50 mL). After drying over $MgSO_4$, filtration, and slow concentration under reduced pressure, stabilizer-free *i*Pr_2O was added when the Et_2O had been reduced to a minimal amount to ease product precipitation (the product is less soluble in *i*Pr_2O than Et_2O, but undergoes decomposition in even less polar hydrocarbon solvents, such as *n*-pentane or *n*-heptane). The solution was cooled to 0 °C and the solid recovered by filtration, followed by washing with a minimal amount of cold *i*Pr_2O. The product was obtained as delicate yellow powder (2.75 g, 8.55 mmol, 41%) after quick (**!**) drying under vacuum (prolonged drying under vacuum provokes black spots to appear in the solid product, which propagate quickly until the product has completely decomposed). Longer storage at −20 °C under Ar atmosphere is not feasible and

the product decomposes within <3 days. Mp 65.4–66.6 °C. ^{1}H NMR (300 MHz, $CDCl_3$, δ): 7.06 (brs, 2H, 14-OH, 15-OH), 7.01 (d, *J*=3.4, 1H, 3-H), 6.04 (d, *J*=3.3, 1H, 4-H), 3.28 (t, *J*=6.8, 2H, 20-H), 2.82 (t, *J*=7.6, 2H, 16-H), 1.63 (s, 9H, 10-H, 11-H, 12-H), 1.71 – 1.57 (m, 4H, 17-H, 19-H), 1.52 – 1.38 (m, 2H, 18-H). ^{13}C NMR (76 MHz, $CDCl_3$, δ): 153.3 (C-6), 141.9 (C-5), 127.4 (C-3), 112.8 (C-4), 86.0 (C-9), 51.4 (C-20), 30.7 (C-16), 29.0 (C-17), 28.9 (C-19), 28.1 (C-10, C-11, C-12), 26.6 (C-18). ^{11}B NMR (96 MHz, $CDCl_3$, δ): 25.3 [s, –*B*$(OH)_2$]. FT-IR (neat, cm^{-1}): 3358, 3152, 3010, 2986, 2975, 2942, 2882, 2864, 2127, 2082, 1705, 1699, 1571, 1494, 1471, 1460, 1429, 1400, 1375, 1369, 1356, 1344, 1289, 1265, 1256, 1235, 1213, 1202, 1166, 1131, 1120, 1084, 1056, 1022, 1002, 948, 891, 863, 835, 806, 780, 763, 731, 696, 681, 656, 615, 582, 559, 510, 465. HRMS (ESI) *m/z:* [M + H]$^+$ calcd for $C_{14}H_{23}BN_4NaO_4$, 345.1705; found, 345.1705.

(*Z*)-5-(5-Azidopentyl)-4'-methoxy-5'-((5-methyl-4-propyl-1*H*-pyrrol-2-yl)methylene)-1*H*,5'*H*-[2,2'-bipyrrol]-1'-ium chloride (2·HCl)

A 50 mL Schlenk flask with magnetic stirring bar was sequentially charged with $Pd(OAc)_2$ (32.6 mg, 145 µmol, 0.05 eq.), SPhos (71.6 mg, 174 µmol, 0.06 eq.), triflate (**10**, 1.10 g, 2.91 mmol, 1.00 eq.), azide boronic acid (**17**, 2.81 g, 8.72 mmol, 3.00 eq.) and evacuated/N_2-refilled three times. Degassed *n*BuOH (16.0 mL) was added, and the mixture was stirred at 20 °C until homogeneous. A solution of K_3PO_4 (1.23 g, 5.81 mmol, 2.00 eq., 1.45 M) in degassed H_2O (4.00 mL) was added in one portion to initiate the Suzuki reaction. The reaction vessel was lowered into a pre-heated heating mantle at 60 °C and the orange solution was stirred under argon for 15 h. The red-orange solution was diluted with CH_2Cl_2, and brine (50 mL) was added. The phases were separated and the aqueous phase was repeatedly extracted with CH_2Cl_2 (25 mL portions) until the organic phase remained nearly colorless. Merged organic phases were dried over $MgSO_4$, filtered over degreased cotton wool (**!**), and the solvent was removed *in vacuo*. The deep red film was chromatographed on silica using CH_2Cl_2 + 0.7–3% NH_3 (7 N in MeOH) as eluent. Acidification of product fractions with 1 M HCl in Et_2O, immobilization on Celite, and chromatography on neutral aluminium oxide (Brockmann I) with *n*-pentane/EtOAc (0–100% EtOAc) provided the product as purple film. The film was dissolved in a minimal amount of CH_2Cl_2 and acidified again. Repeated treatment with *n*-pentane assisted precipitation of the HCl salt as an amorphous purple powder (827 mg, 1.87 mmol, 64%). Mp 75.4–79.2 °C. ^{1}H NMR (300 MHz, $CDCl_3$, δ): 12.52 (brs, 1H, 13-H), 12.47 (brs, 1H, 1-H), 12.39 (brs, 1H, 7-H), 6.84 – 6.77 (m, 2H, 4-H, 11-H), 6.58 (d, *J*=2.2, 1H, 16-H), 6.05 (dd, *J*=3.9, 2.2, 1H, 3-H), 5.98 (d, *J*=1.9, 1H, 10-H), 3.92 (s, 3H, 24-H), 3.25 (t, *J*=6.9, 2H, 21-H), 2.75 (t, *J*=7.6, 2H, 17-H), 2.49 (s, 3H, 25-H), 2.33 (t, *J*=7.5, 2H, 26-H), 1.81 (p, *J*=7.6, 2H, 18-H), 1.69 – 1.55 (m, *J*=7.3, 1H, 20-H) 1.53 (h, *J*=7.5, 2H, 27-H), 1.49 – 1.36 (m, 3H, 19-H), 0.91 (t, *J*=7.3, 3H, 28-H). ^{13}C NMR (76 MHz, $CDCl_3$, δ): 165.7 (C-9), 147.6 (C-6), 145.2 (C-14), 143.8 (C-2), 127.5 (C-15), 127.3 (C-16), 124.9 (C-12), 121.15 (C-8), 121.09 (C-5), 118.6 (C-4), 114.6 (C-11), 110.6 (C-3), 92.6 (C-10), 58.7 (C-24), 51.4 (C-21), 28.6 (C-18, C-20), 28.1 (C-17), 27.5 (C-26), 26.4 (C-19), 23.4 (C-27), 13.8 (C-28), 12.3 (C-25). FT-IR (neat, cm^{-1}): 3162, 3139, 3113, 3070, 2930, 2868, 2178, 2102, 1738, 1705, 1629, 1601, 1537, 1495, 1464, 1448, 1437, 1414, 1403, 1358, 1337, 1292, 1247, 1216, 1201, 1183, 1161, 1128, 1081, 1063, 1044, 998, 990, 976, 965, 902, 884, 838, 790, 778, 762, 731, 659, 634, 622, 556, 552, 509, 475. HRMS (ESI) *m/z:* [M – Cl]$^+$ calcd for $C_{23}H_{31}N_6O$, 407.2554; found, 407.2556.

3-(5-(5-Azidopentyl)-1*H*-pyrrol-2-yl)-5,5-difluoro-1-methoxy-7-methyl-8-propyl-5*H*-5λ^4,6λ^4-dipyrrolo[1,2-*c*:2',1'-*f*][1,3,2]diazaborinine (49)

To a tin foil-covered 25 mL Schlenk flask under N_2 atmosphere was added the prodigiosin A-ring azide HCl salt (**2·HCl**, 101 mg, 227 µmol, 1.00 eq.). The solid was dissolved in dry CH_2Cl_2 (10 mL) (pink solution) before dry DIPEA (194 µL, 1.14 mmol, 5.00 eq.) (red-orange) was added at 20 °C. After stirring for 15 min, the reaction was cooled to 0 °C. Boron trifluoride etherate (112 µL, 909 µmol, 4.00 eq.) was added dropwise at 0 °C to the solution. Once the addition was completed, stirring at 0 °C was continued for 5 h before the reaction was slowly warmed to 20 °C overnight. After a total reaction time of 21 h, the reaction was quenched at 0 °C with 5 mL of ice water. Additional ice water (25 mL) was added and the phases were separated. The aqueous phase was extracted with $CHCl_3$ (3 x 5 mL). After drying of the combined organic phases over $MgSO_4$ and filtration over degreased cotton wool, the flask was covered with tin foil and the solvent was evaporated under reduced pressure. The crude purple product was chromatographed on silica using PE/CH_2Cl_2 (80:20) to provide the BODIPY A-ring azide **49** as a pink fluorescent liquid that solidified to a purple solid (81.3 mg, 179 µmol, 79%) after evaporation of solvent and precipitation with *n*-pentane. R_f (PE/CH_2Cl_2 80:20): 0.47 (visible light). Mp 99.9–100.8 °C. ^{1}H NMR (600 MHz, $CDCl_3$, δ): 10.26 (t, *J*=9.9, 1H, 1-H), 6.92 (s, 1H, 11-H), 6.83 (dd, *J*=3.8, 2.5, 1H, 4-H), 6.54 (s, 1H, 16-H), 6.07 (dd, *J*=2.6, 1H, 3-H), 6.04 (s, 1H, 10-H), 3.93 (s, 3H, 24-H), 3.29 (t, *J*=7.0, 2H, 21-H), 2.76 (t, *J*=7.5, 2H, 17-H), 2.49 (s, 3H, 25-H), 2.38 (t, *J*=7.6, 2H, 26-H), 1.75 (p, *J*=7.6, 2H, 18-H), 1.66 (p, *J*=7.2, 2H, 20-H), 1.57 (h, *J*=7.4, 2H, 27-H), 1.53 – 1.44 (m, 2H, 19-H), 0.97 (t, *J*=7.3, 3H, 28-H). ^{13}C NMR (151 MHz, $CDCl_3$, δ): 163.5 (C-9), 149.3 (C-6), 147.2 (C-14), 141.0 (C-2), 130.5 (C-12), 130.1 (C-15), 128.0 (C-8), 122.6 (C-5), 122.3 (C-16), 118.2 (C-4), 115.9 (C-11), 109.5 (C-3), 96.2 (C-10), 58.5 (C-24), 51.5 (C-21), 28.8 (C-20), 28.7 (C-18), 28.1 (C-26), 28.1 (C-17), 26.4 (C-19), 23.6 (C-27), 14.1 (C-28), 12.4 (C-25). ^{11}B NMR (96 MHz, $CDCl_3$, δ): 1.35 (t, *J*=36.9). ^{19}F NMR (282 MHz, $CDCl_3$, δ): –139.7 (dd, *J*=72.5, 34.7). FT-IR (neat, cm^{-1}): 3430, 2957, 2926, 2872, 2864, 2135, 2084, 1606, 1585, 1546, 1510, 1466, 1457, 1448, 1420, 1397, 1370, 1355, 1302, 1278, 1238, 1312, 1278, 1220, 1205, 1185, 1155, 1141, 1086, 1075, 1050,

1024, 1014, 983, 960, 907, 891, 880, 856, 829, 809, 795, 779, 738, 692, 676, 660, 636, 615, 556, 520, 482, 455. HRMS (ESI) *m/z:* $[M + Na]^+$ calcd for $C_{23}H_{29}BF_2N_6NaO$, 477.2356; found, 477.2353.

***tert*-Butyl 4-hydroxy-2-oxo-2,5-dihydro-1*H*-pyrrole-1-carboxylate (18)**

To a 1 L Schlenk flask under N_2 atmosphere was added Boc-Gly-OH (**19**, 15.0 g, 85.6 mmol, 1.00 eq.), dissolved in dry CH_2Cl_2 (428 mL, 0.2 M solution of Boc-Gly-OH), and cooled to −5 °C [NH_4Cl/ice bath with (25:75 w/w) ratio]. Recrystallized Meldrum's acid (13.6 g, 94.2 mmol, 1.10 eq.) and DMAP (23.0 g, 188 mmol, 2.20 eq.) were added and stirring was continued until dissolved. Isopropyl chloroformate (47.1 mL, 94.2 mmol, 1.10 eq., 2 M in toluene) was added at −5 °C over 60 min with a syringe pump. The weak yellow solution was stirred for 3 h at −5 °C. Afterwards, the mixture was quenched and washed with cold 10% citric acid (2 x 500 mL), cold water (2 x 500 mL), and cold brine (2 x 500 mL). The organic layer was dried over $MgSO_4$, filtered, and the solvent evaporated to give a slurry solid. Drying under high vacuum provided the crude acylated Meldrum's acid as a dry yellow amorphous solid. This crude material was then refluxed in N_2-bubbled dry EtOAc (500 mL) for 2 h to give an intense yellow solution with some precipitate before the solvent was removed under reduced pressure. The product was filtered, then washed with Et_2O, and dried overnight under high vacuum. The Boc-protected tetramic acid **18** (11.1 g, 55.5 mmol, 65%) was obtained as an amorphous beige powder. Mp >320 °C (Decomposition). ^{1}H NMR (600 MHz, DMSO-*d6*) 12.13 (s, 1H, 7-OH), 4.88 (s, 1H, 3-H), 4.14 (s, 2H, 5-H), 1.44 (s, 9H, 12-H, 13-H, 14-H). ^{13}C NMR (151 MHz, DMSO-*d6*, δ): 174.4 (C-4), 169.2 (C-2), 148.9 (C-8), 94.3 (C-3), 80.8 (C-11), 49.4 (C-5), 27.8 (C-12, C-13, C-14). FT-IR (neat, cm^{-1}): 2980, 2937, 1756, 1599, 1432, 1413, 1364, 1302, 1240, 1209, 1151, 1077, 1033, 847, 800, 774, 752, 741, 730, 626, 604, 592, 482, 460. HRMS (ESI) *m/z:* $[M + Na]^+$ calcd for $C_9H_{13}NNaO_4$, 222.0737; found, 222.0737.

4-Azidobutan-1-ol (21)

In a 250 mL Schlenk flask under N_2 atmosphere, 4-chloro-1-butanol (**22**, 5.00 g, 46.1 mmol, 1.00 eq., >85% with variable traces of THF) was dissolved in dry DMF (92.1 mL, 0.5 M). Sodium azide (4.49 g, 69.1 mmol, 1.50 eq.) was added in a single batch, followed by five catalytic drops of DIPEA. The reaction was stirred for 22 h at 70 °C. After cooling to ambient temperature, the reaction was quenched by the slow introduction of ice water (100 mL) and the aqueous phase was repeatedly extracted with Et_2O (6 x 100 mL). Merged organic phases were dried over $MgSO_4$, filtered, and the solvent was removed *in vacuo*. Chromatographic purification of the crude product on silica with *n*-pentane/Et_2O (50:50) provided the product (4.63 g, 40.2 mmol, 87%) as a light-yellow oil. R_f (PE/EtOAc 60:40): 0.29 ($KMnO_4$). ^{1}H NMR (600 MHz, $CDCl_3$, δ): 3.66 (q, *J*=5.1, 2H, 2-H), 3.31 (t, *J*=6.7, 2H, 5-H), 1.74 (d, *J*=5.3, 1H, 1-H), 1.68 (h, *J*=6.7, 1H, 4-H), 1.64 (h, *J*=6.7, 2H, 3-H). ^{13}C NMR (151 MHz, $CDCl_3$, δ): 62.4 (C-2), 51.5 (C-5), 30.0 (C-3), 25.6 (C-4). FT-IR (neat, cm^{-1}): 3333, 2941, 2870, 2505, 2090, 1666, 1451, 1351, 1250, 1173, 1058, 1033, 992, 949, 924, 824, 745, 659, 636, 558, 504. HRMS (ESI) *m/z:* $[M + Na]^+$ calcd for $C_4H_9N_3NaO$, 138.0638; found, 138.0636.

***tert*-Butyl 4-(4-azidobutoxy)-2-oxo-2,5-dihydro-1*H*-pyrrole-1-carboxylate (23)**

In a 500 mL Schlenk flask under N_2 atmosphere, Boc-protected tetramic acid **18** (5.00 g, 25.1 mmol, 1.00 eq.), PPh_3 (7.24 g, 27.6 mmol, 1.10 eq.), and 4-azido-1-butanol (**21**, 4.34 g, 37.7 mmol, 1.50 eq.) were dissolved in dry THF (83.7 mL) at 21 °C (yellow slurry). Diethyl azodicarboxylate (DEAD, 4.3 mL, 27.6 mmol, 1.10 eq.) was added under vigorous stirring to give a brown homogenous solution and stirring was continued for 22 h. The solvent was then evaporated under reduced pressure and the remaining oil was purified by column chromatography on silica with a gradient of *n*-pentane/Et_2O (50:50 to 0:100) to give the desired product as a yellow oil with high viscosity and water content. The product was lyophilized overnight to yield a light yellow amorphous solid (4.14 g, 14.0 mmol, 56%). R_f (PE/EtOAc 40:60): 0.35 (UV and $KMnO_4$). R_f (EtOAc): 0.66 (UV and $KMnO_4$). Mp 31.8–33.8 °C. ^{1}H NMR (600 MHz, $CDCl_3$, δ): 5.07 (s, 1H, 3-H), 4.18 (s, 2H, 5-H), 4.00 (t, *J*=6.2, 2H, 15-H), 3.35 (t, *J*=6.7, 2H, 18-H), 1.91 – 1.82 (m, 2H, 16-H), 1.77 – 1.68 (m, 2H, 17-H), 1.53 (s, 9H, 12-H, 13-H, 14-H). ^{13}C NMR (151 MHz, $CDCl_3$, δ): 173.6 (C-4), 169.3 (C-2), 149.5 (C-8), 95.5 (C-3), 82.8 (C-11), 71.2 (C-15), 51.0 (C-18), 49.5 (C-5), 28.3 (C-12, C-13, C-14), 26.0 (C-16), 25.6 (C-17). FT-IR (neat, cm^{-1}): 2977, 2935, 2874, 2094, 1774, 1734, 1706, 1622, 1451, 1394, 1368, 1322, 1296, 1256, 1240, 1159, 1091, 1071, 1036, 978, 914, 843, 806, 773, 733, 670. HRMS (ESI) *m/z:* $[M + H]^+$ calcd for $C_{13}H_{21}N_4O_4$, 297.1557; found, 297.1556.

4-(4-Azidobutoxy)-1,5-dihydro-2*H*-pyrrol-2-one (24)

In a 250 mL Schlenk flask under N_2 atmosphere, the Boc-protected pyrrolidinone azide (**23**, 3.80 g, 12.8 mmol, 1.00 eq.) was dissolved in dry CH_2Cl_2 (64.1 mL, 5.0 mL $mmol^{-1}$ pyrrolidinone) and cooled to 0 °C for 30 min. Trifluoroacetic acid (64.1 mL, equal volume as CH_2Cl_2) was added slowly and the reaction was warmed to 21 °C. After 90 min stirring at room temperature, the reaction was quenched by slowly adding saturated aqueous $NaHCO_3$ until pH 7 and then extracted with CH_2Cl_2 (3 x 100 mL). The combined organic layers

were washed with water (50 mL) and brine (50 mL), dried over $MgSO_4$, and then filtered. After evaporation of the solvent under reduced pressure, the nearly pure pyrrolinone was obtained as a weak yellow solid. Column chromatography on silica with CH_2Cl_2/MeOH (97:3) furnished the desired product as a yellow solid (2.21 g, 11.3 mmol, 88%). R_f (EtOAc): 0.11 (UV and $KMnO_4$). Mp 80.0–82.7 °C. ^{1}H NMR (600 MHz, $CDCl_3$, δ): 5.66 (brs, 1H, 1-H), 5.06 (s, 1H, 3-H), 3.99 (t, *J*=6.2, 2H, 8-H), 3.93 (s, 2H, 5-H), 3.35 (t, *J*=6.7, 2H, 11-H), 1.90 – 1.83 (m, 2H, 9-H), 1.77 – 1.71 (m, 2H, 10-H). ^{13}C NMR (151 MHz, $CDCl_3$, δ): 175.5 (C-2), 175.0 (C-4), 94.6 (C-3), 70.9 (C-8), 51.1 (C-11), 46.9 (C-5), 26.0 (C-9), 25.6 (C-10). FT-IR (neat, cm^{-1}): 3185, 3070, 2954, 2883, 2857, 2092, 1657, 1614, 1454, 1402, 1345, 1300, 1286, 1228, 1204, 1191, 1090, 1038, 985, 943, 933, 918, 799, 717, 651, 633, 542, 507. HRMS (ESI) *m/z:* [M + H]$^+$ calcd for $C_8H_{13}N_4O_2$, 197.1033; found, 197.1035.

(*Z*)-4-(4-Azidobutoxy)-5-((5-methyl-4-propyl-1*H*-pyrrol-2-yl)methylene)-1,5-dihydro-2*H*-pyrrol-2-one (25)

To a 100 mL Schlenk flask under N_2 atmosphere were added 5-methyl-4-propyl-1*H*-pyrrole-2-carbaldehyde (**6**, 900 mg, 5.95 mmol, 1.00 eq.) and 4-(4-azidobutoxy)-1,5-dihydro-2*H*-pyrrol-2-one (**24**, 2.10 g, 10.7 mmol, 1.80 eq.) and the solids were dissolved in degassed DMSO (20.8 mL, 3.5 mL mmol^{-1} carbaldehyde). Degassed 2 M NaOH (*aq.*) (16.7 mL, 2.8 mL mmol^{-1} carbaldehyde) was added and the reaction was stirred for 24 h at 60 °C. A yellow solid precipitated during the reaction. The product was then partitioned between EtOAc (75 mL) and water (50 mL), the phases were separated, and the aqueous phase was extracted with EtOAc (5 x 75 mL). Merged organic phases were washed with water (100 mL) and brine (150 mL), dried over Na_2SO_4, filtered, and the solvent was removed *in vacuo*. The canary yellow crude product was suspended in *n*-heptane and filtered. The filter cake was washed repeatedly with *n*-heptane, dropwise with *i*Pr_2O/acetone (1:1), and again with *n*-heptane to remove a brown impurity. After drying under high vacuum, the desired product (1.22 g, 3.71 mmol, 62%) was obtained as gold-yellow powder. Mp 150.6–153.3 °C. ^{1}H NMR (600 MHz, $CDCl_3$, δ): 10.63 (brs, 1H, 1-H), 10.15 (brs, 1H, 8-H), 6.27 (s, 1H, 6-H), 6.26 (d, *J*=3.0, 1H, 11-H), 5.07 (d, *J*=1.6, 1H, 3-H), 4.06 (t, *J*=6.1, 2H, 14-H), 3.39 (t, *J*=6.7, 2H, 17-H), 2.35 (t, *J*=7.5, 2H, 20-H), 2.33 (s, 3H, 19-H), 1.96 – 1.89 (m, 2H, 15-H), 1.83 – 1.76 (m, 2H, 16-H), 1.55 (h, *J*=7.4, 2H, 21-H), 0.93 (t, *J*=7.3, 3H, 22-H). ^{13}C NMR (151 MHz, $CDCl_3$, δ): 173.2 (C-2), 166.9 (C-4), 132.3 (C-9), 124.3 (C-7), 122.8 (C-5), 122.7 (C-10), 117.8 (C-11), 102.7 (C-6), 90.5 (C-3), 70.6 (C-14), 51.2 (C-17), 27.9 (C-20), 26.2 (C-15), 25.7 (C-16), 24.2 (C-21), 14.1 (C-22), 11.5 (C-19). FT-IR (neat, cm^{-1}): 3345, 3158, 3030, 2953, 2927, 2868, 2153, 2126, 2080, 1660, 1635, 1566, 1481, 1471, 1415, 1355, 1263, 1233, 1212, 1131, 1114, 1075, 1039, 1007, 1001, 974, 880, 814, 785, 718, 678, 653, 627, 579, 485. HRMS (ESI) *m/z:* [M + H]$^+$ calcd for $C_{17}H_{24}N_5O_2$, 330.1925; found, 330.1923.

(*Z*)-4-(4-Azidobutoxy)-5-((5-methyl-4-propyl-2*H*-pyrrol-2-ylidene)methyl)-1*H*-pyrrol-2-yl trifluoromethanesulfonate (26)

A 100 mL Schlenk flask under N_2 atmosphere was charged with dipyrrinone (**25**, 1.00 g, 3.04 mmol, 1.00 eq.) and the solid dissolved in dry CH_2Cl_2 (60 mL, 19.5 mL mmol^{-1}). At 0 °C, trifluoromethanesulfonic anhydride (613 µL, 3.64 mmol, 1.20 eq., freshly opened**!**) was added dropwise to the solution of dipyrrinone **25** over 30 min. After additional stirring at this temperature for 1 h, the dark brown reaction mixture was poured into 2% $NaHCO_3$ (200 mL) and the aqueous phase was repeatedly extracted with MTBE (3 x 50 mL). The combined organic layers were washed with brine (200 mL), dried over Na_2SO_4, filtered, and the solvent was removed *in vacuo* to a minimal volume. Excess *n*-pentane was added repeatedly during evaporation to support precipitation. After complete evaporation of the solvent, the pure triflate was obtained as a brown-yellow amorphous solid (1.37 g, 2.96 mmol, 98%) after extensive drying. Mp 47.9–49.3 °C. ^{1}H NMR (300 MHz, $CDCl_3$, δ): 10.70 (brs, 1H, 1-H), 6.98 (s, 1H, 6-H), 6.53 (s, 1H, 11-H), 5.38 (s, 1H, 3-H), 4.02 (t, *J*=5.9, 2H, 18-H), 3.38 (t, *J*=6.5, 2H, 21-H), 2.35 (t, *J*=7.5, 2H, 24-H), 2.30 (s, 3H, 23-H), 1.96 – 1.84 (m, 2H, 19-H), 1.84 – 1.71 (m, 2H, 20-H), 1.55 (h, *J*=7.4, 2H, 25-H), 0.93 (t, *J*=7.3, 3H, 26-H). ^{13}C NMR (76 MHz, $CDCl_3$, δ): 166.4 (C-4), 160.5 (C-2), 138.6 (C-9), 131.7 (C-5), 127.4 (C-7), 125.7 (C-10), 123.3 (C-11), 121.9 (C-6), 118.8 (q, *J*=321.0, C-16), 87.2 (C-3), 71.1 (C-18), 51.2 (C-21), 27.8 (C-24), 26.3 (C-19), 25.7 (C-20), 23.8 (C-25), 13.9 (C-26), 12.3 (C-23). ^{19}F NMR (282 MHz, $CDCl_3$, δ): −72.7 (s, −C*F*$_3$). FT-IR (neat, cm^{-1}): 3351, 3137, 2959, 2938, 2875, 2846, 2189, 2097, 1622, 1568, 1547, 1503, 1468, 1421, 1397, 1366, 1336, 1257, 1242, 1231,1207, 1150, 1139, 1125, 1078, 1010, 996, 974, 931, 916, 896, 823, 810, 765, 757, 738, 684, 654, 637, 623, 592, 576, 555, 520, 494. HRMS (ESI) *m/z:* [M + H]$^+$ calcd for $C_{18}H_{23}F_3N_5O_4S$, 462.1417; found, 462.1422.

(1*H*-Pyrrol-2-yl)pentan-1-one (28)

In a 1 L Schlenk flask under N_2 atmosphere, aluminium(III) chloride (15.9 g, 119 mmol, 1.20 eq.) was suspended in dry CH_2Cl_2 (450 mL) and pentanoyl chloride (**27**, 11.8 mL, 99.5 mmol, 1.00 eq.) was added slowly to the aluminium salt. After stirring for 30 min at 21 °C to dissolve the inorganic salt, the red solution was cooled to 0 °C and a solution of 1*H*-pyrrole (7.60 mL, 110 mmol, 1.10 eq.) in dry CH_2Cl_2 (50 mL) was slowly transferred to the acid chloride solution, causing a color shift from brown to deep orange. The reaction was stirred for a further 1 h at 0 °C, then slowly thawed to 24 °C and stirred at this temperature for 13 h, yielding a deep orange colored solution. At 0 °C, saturated NH_4Cl (*aq.*) was used for reaction quenching. The phases were separated and the aqueous phase was subsequently

extracted with CH_2Cl_2 (3 x 150 mL). The organic phases were merged, washed with saturated $NaHCO_3$ (2 x 150 mL) and brine (1 x 150 mL), dried over $MgSO_4$, filtered, and concentrated *in vacuo*. The oily residue was purified by column chromatography on silica with *n*-pentane/MTBE (90:10) to afford the product (9.14 g, 60.5 mmol, 61%) as orange oil. R_f (PE/EtOAc 80:20): 0.38 (UV and $KMnO_4$). ^{1}H NMR (600 MHz, $CDCl_3$, δ): 10.22 (brs, 1H, 1-H), 7.05 (td, *J*=2.7, 1.3, 1H, 5-H), 6.93 (ddd, *J*=3.8, 2.4, 1.3, 1H, 3-H), 6.27 (dt, *J*=3.8, 2.5, 1H, 4-H), 2.78 (t, *J*=7.6, 2H, 8-H), 1.72 (p, *J*=7.6, 2H, 9-H), 1.40 (h, *J*=7.4, 2H, 10-H), 0.94 (t, *J*=7.4, 3H, 11-H). ^{13}C NMR (151 MHz, $CDCl_3$, δ): 191.6 (C-6), 132.1 (C-2), 125.0 (C-5), 116.4 (C-3), 110.5 (C-4), 37.9 (C-8), 27.6 (C-9), 22.7 (C-10), 14.0 (C-11). FT-IR (neat, cm^{-1}): 3276, 2958, 2931, 2872, 1710, 1631, 1545, 1427, 1402, 1307, 1294, 1126, 1093, 1046, 923, 883, 844, 748, 606. HRMS (ESI) *m/z:* $[M + H]^+$ calcd for $C_9H_{14}NO$, 152.1070; found, 152.1068.

2-Pentyl-1*H*-pyrrole (29)

In a 500 mL Schlenk flask under N_2 atmosphere, $NaBH_4$ (6.17 g, 163 mmol, 2.80 eq.) was suspended in dry *i*PrOH (100 mL) and cooled to 0 °C. A solution of the acylpyrrole (**28**, 8.81 g, 58.3 mmol, 1.00 eq.) in dry *i*PrOH (200 mL) was added to the sodium borohydride suspension at 0 °C. Afterwards, the solution was refluxed for 17 h at 99 °C. The reaction was quenched with water at 0 °C and after the addition of more water (300 mL), the product was extracted with MTBE (3 x 250 mL). Merged organic phases were washed with brine (250 mL), dried over $MgSO_4$, filtered, and the solvent removed *in vacuo*. The residual oil was purified by column chromatography on silica with *n*-pentane/MTBE (98:2) + 0.5% TEA, and the alkylpyrrole was obtained as a colorless oil (7.16 g, 52.2 mmol, 90%). R_f (PE/EtOAc 80:20): 0.58 ($KMnO_4$). ^{1}H NMR (600 MHz, $CDCl_3$, δ): 7.90 (brs, 1H, 1-H), 6.70 – 6.66 (m, 1H, 5-H), 6.16 (p, *J*=2.6, 1H, 4-H), 5.94 (t, *J*=2.7, 2H, 3-H), 2.65 – 2.59 (m, 2H, 6-H), 1.69 – 1.61 (m, 2H, 7-H), 1.40 – 1.32 (m, 4H, 8-H, 9-H), 0.96 – 0.89 (m, 3H, 10-H). ^{13}C NMR (151 MHz, $CDCl_3$, δ): 133.0 (C-2), 116.1 (C-5), 108.3 (C-4), 105.0 (C-3), 31.7 (C-8), 29.5 (C-7), 27.8 (C-6), 22.6 (C-9), 14.2 (C-10). FT-IR (neat, cm^{-1}): 3383, 3096, 2956, 2926, 2857, 1567, 1467, 1459, 1426, 1378, 1119, 1096, 1024, 957, 883, 783, 710, 562. HRMS (ESI) *m/z:* $[M + H]^+$ calcd for $C_9H_{16}N$, 138.1277; found, 138.1275.

tert-Butyl 2-pentyl-1*H*-pyrrole-1-carboxylate (30)

In a 250 mL Schlenk flask under N_2 atmosphere, 2-pentyl-1*H*-pyrrole (**29**, 6.77 g, 49.4 mmol, 1.00 eq.) and DMAP (603 mg, 4.94 mmol, 0.10 eq.) were dissolved in dry CH_2Cl_2 (40 mL). A solution of Boc_2O (12.9 g, 59.2 mmol, 1.20 eq.) in dry CH_2Cl_2 (100 mL) was added to the prior solution at 25 °C, turning instantly yellow, and the reaction was stirred for 18 h at 25 °C. The solvent was removed under reduced pressure and the orange residue purified by column chromatography on silica using *n*-pentane/MTBE (99:1) as eluent. The product was afforded as a pale-yellow liquid (11.4 g, 48.1 mmol, 97%). R_f (PE/EtOAc 80:20): 0.88 ($KMnO_4$). ^{1}H NMR (600 MHz, $CDCl_3$, δ): 7.19 (dd, *J*=3.4, 1.8, 1H, 5-H), 6.07 (t, *J*=3.3, 1H, 4-H), 5.95 (ddt, *J*=3.3, 1.9, 1H, 3-H), 2.85 – 2.80 (m, 2H, 13-H), 1.66 – 1.55 (m, 11H, 10-H, 11-H, 12-H, 14-H), 1.40 – 1.31 (m, 4H, 15-H, 16-H), 0.93 – 0.87 (m, 3H, 17-H). ^{13}C NMR (151 MHz, $CDCl_3$, δ): 149.7 (C-6), 136.7 (C-2), 120.9 (C-5), 110.8 (C-3), 110.0 (C-4), 83.3 (C-9), 31.8 (C-15), 29.0 (C-13), 28.8 (C-14), 28.2 (C-10, C-11, C-12), 22.7 (C-16), 14.2 (C-17). FT-IR (neat, cm^{-1}): 2957, 2931, 2871, 2861, 1738, 1495, 1478, 1458, 1408, 1394, 1369, 1326, 1317, 1254, 1235, 1166, 1125, 1059, 1010, 963, 883, 851, 844, 800, 772, 715, 599, 560, 497, 461. HRMS (ESI) *m/z:* $[M + H]^+$ calcd for $C_{14}H_{24}NO_2$, 238.1802; found, 238.1801.

(1-(*tert*-Butoxycarbonyl)-5-pentyl-1*H*-pyrrol-2-yl)boronic acid (31)

In a 250 mL Schlenk flask under N_2 atmosphere, TMP (10.7 mL, 63.2 mmol, 2.00 eq.) was dissolved in dry THF (74.6 mL, 2.36 mL $mmol^{-1}$ pyrrole) and cooled to −78 °C. *n*-Butyl lithium (27.1 mL, 66.4 mmol, 2.10 eq., 2.45 M in *n*-hexane) was added dropwise to the TMP solution in THF at 0.9 mL min^{-1} (syringe pump). After completion of the addition and further stirring for 15 min at −78 °C, the mixture was allowed to warm to 0 °C over 30 min and was then cooled again to −78 °C. In a 500 mL three-necked flask under N_2 atmosphere, equipped with a cold thermometer and connection to the line, was *tert*-butyl 2-pentyl-1*H*-pyrrole-1-carboxylate (**31**, 7.50 g, 31.6 mmol, 1.00 eq.) dissolved in dry THF (126.4 mL, 4.00 mL $mmol^{-1}$ pyrrole) and cooled to −78 °C. The LiTMP was then added dropwise with a transfer cannula to the glass surface of the flask with the pyrrolic THF solution at the slowest possible speed by adding positive pressure of argon to the Schlenk flask. The internal temperature was constantly kept below −70 °C and the rate was adjusted if needed. The reaction mixture was stirred for 2 h at −78 °C, before a solution of trimethyl borate (10.6 mL, 94.8 mmol, 3.00 eq.) in dry THF (33.8 mL, 1.07 mL $mmol^{-1}$ pyrrole) was added dropwise at 0.75 mL min^{-1} (syringe pump). The solution was stirred for 15 min at −78 °C, then 30 min at 0 °C, and finally stirred for 12 h at 23 °C. The reaction was quenched at 0 °C by the dropwise addition of 0.20 M HCl (332 mL, 66.4 mmol, 2.10 eq.). Phases were separated and the aqueous phase was extracted with Et_2O (3 x 150 mL). Merged organic phases were washed with water (2 x 250 mL) and brine (2 x 250 mL), dried over a minimal amount $MgSO_4$, filtered, and the solvent removed *in vacuo*. As the product typically did not precipitate upon removal of the ethereal solvent, the residue was taken up in stabilizer-free *i*Pr_2O (the product is less soluble in *i*Pr_2O than Et_2O, but undergoes decomposition in even less polar hydrocarbon

solvents, such as *n*-pentane or *n*-heptane) and washed again with 0.2 M HCl (2 x 150 mL). After drying over $MgSO_4$, filtration, and slow solvent removal, the boronic acid started to precipitate as a light-yellow solid. The suspension was cooled to −20 °C and the boronic acid was recovered by filtration and washed with cold *i*Pr_2O. When the *i*Pr_2O wash fraction was treated with water and left to evaporate the ether, more boronic acid crystals formed at the surface as a brown solid. Trituration with cold toluene provided another fraction of pure product as a white solid. The isolated boronic acid was obtained as an amorphous white solid (5.84 g, 20.8 mmol, 66%) after drying. Storage for a longer period at −20 °C under Ar atmosphere is feasible. Mp 75.2–75.8 °C. ^{1}H NMR (300 MHz, $CDCl_3$, δ): 7.35 (brs, 2H, 14-OH, 15-OH), 7.02 (d, *J*=3.4, 1H, 3-H), 6.04 (d, *J*=3.4, 1H, 4-H), 2.79 (t, *J*=7.7, 2H, 16-H), 1.70 – 1.51 (m, 11H, 10-H, 11-H, 12-H, 17-H), 1.34 (dt, *J*=7.3, 3.7, 4H, 18-H, 19-H), 0.96 – 0.85 (m_c, *J*=6.7, 3H, 20-H). ^{13}C NMR (76 MHz, $CDCl_3$, δ): 153.4 (C-6), 142.6 (C-5), 127.5 (C-3), 112.7 (C-4), 85.9 (C-9), 31.8 (C-18), 30.9 (C-16), 29.2 (C-17), 28.1 (C-10, C-11, C-12), 22.7 (C-19), 14.2 (C-20). ^{11}B NMR (96 MHz, $CDCl_3$, δ): 25.5 [s, −*B*$(OH)_2$]. FT-IR (neat, cm^{-1}): 3364, 3190, 2960, 2936, 2928, 2873, 2861, 1697, 1572, 1494, 1467, 1428, 1399, 1378, 1362, 1347, 1337, 1266, 1256, 1239, 1198, 1198, 1166, 1111, 1060, 1048, 1018, 1002, 949, 863, 832, 795, 779, 761, 732, 710, 682, 657, 615, 583, 510, 466. HRMS (ESI) *m/z:* $[M + H]^+$ calcd for $C_{14}H_{25}BNO_4$, 282.1871; found, 282.1874.

(*Z*)-4'-(4-Azidobutoxy)-5'-((5-methyl-4-propyl-1*H*-pyrrol-2-yl)methylene)-5-pentyl-1*H*,5'*H*-[2,2'-bipyrrol]-1'-ium chloride (3·HCl)

A 100 mL Schlenk flask with magnetic stirring bar was sequentially charged with $Pd(OAc)_2$ (28.0 mg, 125 µmol, 0.05 eq.), SPhos (61.4 mg, 150 µmol, 0.06 eq.), triflate (**26**, 1.15 g, 2.49 mmol, 1.00 eq.), alkyl boronic acid (**31**, 1.40 g, 4.98 mmol, 2.00 eq.) and evacuated/N_2-refilled three times. Degassed *n*BuOH (13.7 mL) was added and the mixture was stirred at 20 °C until homogeneous. A solution of K_3PO_4 (1.06 g, 4.98 mmol, 2.00 eq., 1.45 M) in degassed H_2O (3.43 mL) was added in one portion to initiate the Suzuki reaction. The reaction vessel was lowered into a pre-heated heating mantle at 60 °C and the orange solution was stirred under argon for 13 h. The red-orange solution was diluted with CH_2Cl_2, and brine (50 mL) was added. The phases were separated and the aqueous phase was repeatedly extracted with CH_2Cl_2 (25 mL portions) until the organic phase remained nearly colorless. Merged organic phases were dried over $MgSO_4$, filtered over degreased cotton wool (**!**), and the solvent was removed *in vacuo*. The deep red film was chromatographed on silica using CH_2Cl_2 + 0.7% NH_3 (7 N in MeOH) as eluent. Acidification of product fractions with 1 M HCl in Et_2O, immobilization on Celite, and chromatography on neutral aluminium oxide (Brockmann I) with *n*-pentane/EtOAc (0–100% EtOAc) provided the product as purple film. The film was dissolved in a minimal amount of CH_2Cl_2 and acidified again. Repeated treatment with *n*-pentane assisted precipitation of the HCl salt as an amorphous purple powder (651 mg, 1.34 mmol, 54%) during solvent removal. Mp 72.4–75.8 °C. ^{1}H NMR (300 MHz, $CDCl_3$, δ): 12.60 (brs, 1H, 13-H), 12.50 (brs, 2H, 1-H, 7-H), 6.85 (s, 1H, 11-H), 6.84 (dd, *J*=3.8, 2.5, 1H, 4-H), 6.65 (d, *J*=2.6, 1H, 16-H), 6.09 (dd, *J*=3.9, 2.2, 1H, 3-H), 5.99 (d, *J*=1.8, 1H, 10-H), 4.16 (t, *J*=6.1, 2H, 23-H), 3.40 (t, *J*=6.5, 2H, 26-H), 2.76 (t, *J*=7.7, 2H, 17-H), 2.52 (s, 3H, 28-H), 2.37 (t, *J*=7.5, 2H, 29-H), 2.01 – 1.88 (m, 2H, 25-H), 1.87 – 1.72 (m, 4H, 18-H, 24-H), 1.56 (h, *J*=7.4, 2H, 30-H), 1.41 – 1.29 (m, 4H, 19-H, 20-H), 0.93 (t, *J*=7.3, 3H, 31-H), 0.88 (d, *J*=6.9, 3H, 21-H). ^{13}C NMR (76 MHz, $CDCl_3$, δ): 164.6 (C-9), 147.8 (C-6), 145.4 (C-14), 145.0 (C-2), 127.6 (C-15), 127.4 (C-16), 125.0 (C-12), 121.4 (C-8), 121.0 (C-5), 118.7 (C-4), 114.5 (C-11), 110.6 (C-3), 93.0 (C-10), 71.2 (C-23), 51.1 (C-26), 31.6 (C-19), 28.9 (C-18), 28.3 (C-17), 27.6 (C-29), 26.3 (C-25), 25.6 (C-24), 23.5 (C-30), 22.5 (C-20), 14.2 (C-21), 13.9 (C-31), 12.4 (C-28). FT-IR (neat, cm^{-1}): 3161, 3137, 3107, 3060, 2956, 2927, 2869, 2861, 2096, 1628, 1603, 1576, 1545, 1533, 1493, 1441, 1336, 1245, 1199, 1152, 1128, 1078, 1042, 999, 966, 893, 846, 820, 784, 770, 734, 684, 664, 624, 566, 479. HRMS (ESI) *m/z:* $[M - Cl]^+$ calcd for $C_{26}H_{37}N_6O$, 449.3023; found, 449.3031.

4-Methoxy-4-oxobutanoic acid (S23)

To a 250 mL three-necked flask under N_2 atmosphere, succinic anhydride (**S11**, 20.0 g, 200 mmol, 1.00 eq.) and dry MeOH (70 mL) were added. The suspension was subsequently heated and refluxed for 2 h at 70 °C. After cooling to ambient temperature, the solvent was removed under reduced pressure to yield a colorless liquid with a sweet odor. The title compound solidified as a white crystalline (26.3 g, 199 mmol, >99%) solid at room temperature in quantitative yield and is used without further purification after drying under high vacuum. R_f (CH_2Cl_2/MeOH 96:4): 0.35 (concentration dependent, $KMnO_4$). Mp 54.8–58.1 °C. ^{1}H NMR (600 MHz, $CDCl_3$, δ): 11.49 (brs, 1H, 6-OH), 3.70 (s, 3H, 9-H), 2.69 (t, *J*=6.8, 2H, 2-H), 2.63 (t, *J*=6.8, 2H, 3-H). ^{13}C NMR (151 MHz, $CDCl_3$, δ): 178.3 (C-1), 172.7 (C-4), 52.1 (C-9), 29.1 (C-2), 28.8 (C-3). FT-IR (neat, cm^{-1}): 3010, 2980, 2956, 2933, 2756, 2668, 2620, 2591, 2570, 1728, 1688, 1437, 1416, 1365, 1344, 1299, 1253, 1232, 1198, 1171, 1068, 1003, 971, 943, 852, 843, 688, 669, 641, 580, 554, 533. HRMS (ESI) *m/z:* $[M + H]^+$ calcd for $C_5H_9O_4$, 133.0495; found, 133.0496.

Methyl 4-chloro-4-oxobutanoate (34)

To a 100 mL round-bottom flask under N_2 atmosphere were added fine powdered 4-methoxy-4-oxobutanoic acid (**S23**, 12.0 g, 90.8 mmol, 1.00 eq.) and thionyl chloride (45.4 mL, 627 mmol, 6.90 eq., 0.50 mL $mmol^{-1}$ acid) before the clear solution was heated to

40 °C for 4 h and then to 75 °C for 30 min. Afterwards, excess thionyl chloride was removed under reduced pressure and traces forced out by stepwise co-evaporation with CH_2Cl_2 (4 x 5 mL) to yield the title compound as a volatile, light-yellow liquid (13.0 g, 86.6 mmol, 95%), which was used without further purification. R_f (CH_2Cl_2/MeOH 95:5): 0.70 ($KMnO_4$). ^{1}H NMR (600 MHz, $CDCl_3$, δ): 3.71 (s, 3H, 9-H), 3.22 (t, *J*=6.5, 2H, 2-H), 2.68 (t, *J*=6.6, 2H, 3-H). ^{13}C NMR (151 MHz, $CDCl_3$, δ): 173.2 (C-1), 171.5 (C-4), 52.3 (C-9), 41.9 (C-2), 29.2 (C-3). FT-IR (neat, cm^{-1}): 3004, 2956, 2851, 1789, 1735, 1439, 1410, 1397, 1369, 1346, 1305, 1264, 1236, 1207, 1175, 1062, 1050, 1025, 986, 962, 917, 886, 839, 789, 761, 712, 658, 583, 488.

Ethyl 4-(4-methoxy-4-oxobutanoyl)-5-methyl-1*H*-pyrrole-2-carboxylate (35)

A 500 mL Schlenk flask under N_2 atmosphere was charged with anhydrous $AlCl_3$ (17.4 g, 131 mmol, 2.00 eq.) and was suspended in dry CH_2Cl_2 (120 mL, 1.86 mL $mmol^{-1}$ pyrrole) and subsequently cooled to 0 °C. Methyl 4-chloro-4-oxobutanoate (**34**, 12.5 g, 82.9 mmol, 1.27 eq.) was added at 0 °C, and the suspension was stirred for 30 min at 21 °C, after which time the salt had mostly dissolved and the solution had turned brown yellow. Then, a solution of ethyl 5-methyl-1*H*-pyrrole-2-carboxylate (**33**, 10.0 g, 65.3 mmol, 1.00 eq.) in dry CH_2Cl_2 (60 mL, 0.93 mL $mmol^{-1}$ pyrrole) was added and the reaction mixture was stirred at 21 °C for 15 h. The reaction was quenched at 0 °C with ice water (150 mL), the phases were separated and the aqueous phase was extracted with CH_2Cl_2 (3 × 75 mL). The combined organic layers were washed with brine (2 x 150 mL), dried over $MgSO_4$, filtered, and the solvent was removed *in vacuo* to provide the crude product as a pink-purple solid. Chromatography on silica with CH_2Cl_2/EtOAc gradient (100:0 to 80:20) gave the product (14.6 g, 54.5 mmol, 84%) as a light-yellow solid. R_f (PE/EtOAc 70:30): 0.39 (UV). Mp 127.2–130.7 °C. ^{1}H NMR (600 MHz, CD_2Cl_2, δ): 9.90 (brs, 1H, 1-H), 7.26 (d, *J*=2.5, 1H, 3-H), 4.32 (q, *J*=7.1, 2H, 9-H), 3.66 (s, 3H, 18-H), 3.10 (t, *J*=6.7, 2H, 12-H), 2.65 (t, *J*=6.7, 2H, 13-H), 2.55 (s, 3H, 19-H), 1.36 (t, *J*=7.1, 3H, 10-H). ^{13}C NMR (151 MHz, CD_2Cl_2, δ): 194.7 (C-11), 173.9 (C-14), 161.4 (C-6), 139.8 (C-5), 122.0 (C-4), 121.0 (C-2), 116.6 (C-3), 61.2 (C-9), 51.9 (C-18), 35.1 (C-12), 28.2 (C-13), 14.5 (C-10), 14.1 (C-19). FT-IR (neat, cm^{-1}): 3301, 2994, 2961, 2940, 2912, 1710, 1661, 1569, 1500, 1445, 1411, 1375, 1362, 1341, 1306, 1267, 1225, 1190, 1178, 1125, 1090, 1035, 999, 987, 956, 908, 876, 847, 798, 766, 703, 671, 655, 615, 577, 502. HRMS (ESI) *m/z:* $[M + H]^+$ calcd for $C_{13}H_{18}NO_5$, 268.1179; found, 268.1180.

Ethyl 4-(4-hydroxybutyl)-5-methyl-1*H*-pyrrole-2-carboxylate (36)

In a 250 mL Schlenk flask under N_2 atmosphere, acylated pyrrole carboxylate (**35**, 8.00 g, 29.9 mmol, 1.00 eq.) was dissolved in dry THF (49.4 mL, 1.65 mL $mmol^{-1}$ carboxylate) to give a clear pale-yellow solution. $NaBH_4$ (2.50 g, 62.9 mmol, 2.10 eq.) was added at room temperature under gas development and a pale-yellow suspension remained in the reaction flask. Subsequently, boron trifluoride etherate (10.3 mL, 83.81 mmol, 2.80 eq.) was added with a syringe pump over 20 min at 0.517 mL min^{-1}, followed by 3 h stirring at 21 °C (a white solid precipitates). The reaction was carefully quenched at 0 °C by the addition of glacial acetic acid (4.5 mL) and water (55 mL) until the solution became clear yellow. The wet THF phase was extracted with CH_2Cl_2 (5 x 100 mL). Merged organic phases were washed with saturated $NaHCO_3$ (2 x 100 mL) and brine (2 x 200 mL) [the aqueous phase remains turbid while the organic phase is turbid yellow], dried over $MgSO_4$, filtered, and the solvent was removed *in vacuo*. The sticky yellow residue was almost pure product and crystallized upon thorough removal of THF at high vacuum to give an amorphous salmon pink solid (5.40 g, 24.0 mmol, 80%). If the product did not crystallize or the melting point did not match, boric acid impurities were likely to be present. R_f (PE/EtOAc 50:50): 0.34 (UV). Mp 79.3–81.9 °C. ^{1}H NMR (600 MHz, $CDCl_3$, δ): 9.39 (brs, 1H, 1-H), 6.69 (d, *J*=2.7, 1H, 3-H), 4.27 (q, *J*=7.2, 2H, 9-H), 3.65 (t, *J*=5.4, 2H, 14-H), 2.39 (t, *J*=6.7, 2H, 11-H), 2.20 (s, 3H, 16-H), 1.64–1.54 (m, 4H, 12-H, 13-H), 1.33 (t, *J*=7.1, 3H, 10-H). ^{13}C NMR (151 MHz, $CDCl_3$, δ): 161.6 (C-6), 130.8 (C-5), 122.3 (C-4), 119.8 (C-2), 115.8 (C-3), 62.9 (C-14), 60.1 (C-9), 32.4 (C-13), 27.0 (C-12), 25.5 (C-11), 14.6 (C-10), 11.4 (C-16). FT-IR (neat, cm^{-1}): 3302, 3270, 2988, 2937, 2932, 2864, 1656, 1581, 1504, 1499, 1485, 1460, 1417, 1375, 1340, 1323, 1264, 1221, 1203, 1127, 1059, 1023, 982, 940, 924, 910, 883, 838, 821, 776, 769, 685, 670, 645, 618, 608, 562, 551, 508, 480, 457. HRMS (ESI) *m/z:* $[M + H]^+$ calcd for $C_{12}H_{20}NO_3$, 226.1438; found, 226.1436.

4-(2-Methyl-1*H*-pyrrol-3-yl)butan-1-ol (32)

From the acylation route: Highly optimized procedure: To a 250 mL three-necked flask under N_2 atmosphere, equipped with a reflux condenser and thermometer, were added sodium hydroxide (2.73 g, 68.2 mmol, 3.20 eq.) and acylated pyrrole carboxylate (**36**, 4.80 g, 21.3 mmol, 1.00 eq.). The solids were then suspended in anhydrous ethylene glycol (53.3 mL, 2.5 mL $mmol^{-1}$ pyrrole) and the mixture degassed by two consecutive freeze-pump-thaw cycles. The reaction mixture was stirred at 60 °C until all solids had completely dissolved. The flask was then lowered into a pre-heated heating vessel at 130 °C and stirred for 15 min from the point where the internal temperature had reached 120 °C to convert the starting material to the corresponding acid under mild conditions (solution gets orange, no decarboxylation or polymerization). Then, the reaction was lowered immediately into a pre-heated vessel at 210 °C for decarboxylation and maintained at 180 °C for exactly 12 min (**!**) from the point where the internal temperature had reached 180 °C

(heating to 180 °C required approx. 2 min, turning brown). The solution was immediately cooled with an ice bath and diluted with water (400 mL). The aqueous phase was extracted with EtOAc (3 x 150 mL), the merged organic phases were washed with brine (2 x 300 mL), dried over $MgSO_4$, filtered, and the solvent was removed *in vacuo* to provide a viscous brown oil. Column chromatography on silica with CH_2Cl_2/EtOAc (80:20) yielded the desired pyrrole as a viscous yellow-orange oil (2.52 g, 16.4 mmol, 77%). R_f (CH_2Cl_2/EtOAc 60:40): 0.48 (UV and $KMnO_4$). ^{1}H NMR (600 MHz, $CDCl_3$, δ): 7.80 (brs, 1H, 1-H), 6.60 (t, *J*=2.7, 1H, 5-H), 6.02 (t, *J*=2.8, 1H, 4-H), 3.66 (t, *J*=6.1, 2H, 10-H), 2.44 (t, *J*=7.0, 2H, 7-H), 2.19 (s, 3H, 6-H), 1.62 (dq, *J*=7.1, 3.3, 4H, 8-H, 9-H). ^{13}C NMR (151 MHz, $CDCl_3$, δ): 123.4 (C-2), 119.3 (C-3), 115.1 (C-5), 108.8 (C-4), 63.1 (C-10), 32.7 (C-9), 27.4 (C-8), 25.7 (C-7), 11.1 (C-6). FT-IR (neat, cm^{-1}): 3365, 3094, 2929, 2857, 1722, 1586, 1444, 1379, 1338, 1275, 1256, 1246, 1208, 1173, 1137, 1102, 1058, 1032, 980, 959, 932, 901, 834, 715, 672, 665, 600, 563, 510, 504. HRMS (ESI) *m/z:* $[M + Na]^+$ calcd for $C_9H_{15}NNaO$, 176.1046; found, 176.1044.

3-(4-Azidobutyl)-2-methyl-1*H*-pyrrole (38)

In a 100 mL round-bottom flask under N_2 atmosphere, the hydroxy pyrrole (**32**, 1.38 g, 8.99 mmol, 1.00 eq.) and DMAP (1.65 g, 13.5 mmol, 1.50 eq.) were dissolved in dry CH_2Cl_2 (25.7 mL, 350 mM pyrrole) and stirred for 10 min at 20 °C. At 0 °C, a solution of *p*TsCl (25.2 mL, 2.40 g, 12.6 mmol, 1.40 eq., 500 mM in dry CH_2Cl_2) was added dropwise over 25 min and stirring continued at 0 °C for 4 h before slowly warming to 20 °C overnight by melting the ice (18 h in total, reaction solution is yellow). The reaction was quenched at 0 °C by slowly adding ice water (30 mL) and additional stirring for 15 min. More ice water (170 mL) was added, phases were separated, and the turbid aqueous phase was subsequently extracted with CH_2Cl_2 (3 x 25 mL). After drying over Na_2SO_4, filtration, and partial solvent evaporation (not evaporated to dryness**!** Otherwise, the product will degrade), the yellow residue was transferred to a 50 mL round-bottom flask and dissolved in dry DMF (12 mL). The excess CH_2Cl_2 was further removed under reduced pressure (>30 mbar) and sodium azide (2.05 g, 31.5 mmol, 3.50 eq.) was added under argon atmosphere to the clear orange solution of tosylate **37**. After adding five catalytic drops of dry DIPEA, the reaction was stirred overnight for 21 h at 20 °C (green brown with precipitate) and quenched at 0 °C by adding ice water (25 mL). After further dilution with ice water (100 mL), the residue was extracted with Et_2O (3 x 50 mL). Merged organic phases were washed with ice water (2 x 100 mL, getting yellow) and dried over $MgSO_4$. After filtration, the crude product was obtained as orange oil after solvent evaporation and chromatographed on silica with PE/CH_2Cl_2 (95:5) + 0.5% TEA to yield the azide as orange-yellow oil (1.14 g, 6.37 mmol, 71% over two steps). R_f (PE/CH_2Cl_2 20:80): 0.67 (UV and anisaldehyde). R_f (PE/EtOAc 60:40): 0.82 (UV and anisaldehyde). ^{1}H NMR (600 MHz, $CDCl_3$, δ): 7.75 (brs, 1H, 1-H), 6.61 (t, *J*=2.7, 1H, 5-H), 6.02 (t, *J*=2.8, 1H, 4-H), 3.29 (t, *J*=6.6, 2H, 10-H), 2.45 (t, *J*=7.1, 2H, 7-H), 2.20 (s, 3H, 6-H), 1.73–1.54 (m, 4H, 8-H, 9-H). ^{13}C NMR (151 MHz, $CDCl_3$, δ): 123.5 (C-2), 118.8 (C-3), 115.2 (C-5), 108.8 (C-4), 51.6 (C-10), 28.7 (C-8), 28.4 (C-9), 25.5 (C-7), 11.1 (C-6). FT-IR (neat, cm^{-1}): 3452, 3376, 2930, 2857, 2091, 1731, 1667, 1585, 1542, 1462, 1453, 1445, 1408, 1370, 1352, 1298, 1266, 1249, 1178, 1138, 1101, 1065, 1008, 955, 917, 901, 857, 835, 714, 674, 664, 641, 557, 491, 484, 477. HRMS (ESI) *m/z:* $[M + H]^+$ calcd for $C_9H_{15}N_4$, 179.1291; found, 179.1290.

4-(4-Azidobutyl)-5-methyl-1*H*-pyrrole-2-carbaldehyde (39)

To a 25 mL two-necked flask under N_2 atmosphere was added dry DMF (523 µL, 6.79 mmol, 1.20 eq.) and the solvent was cooled to 0 °C. Phosphoryl chloride (633 µL, 6.79 mmol, 1.20 eq.) was added dropwise at 0 °C to precipitate the Vilsmeier reagent as a white solid. At 0 °C, the Vilsmeier reagent was diluted with degassed dry 1,2-dichloroethane (1.70 mL, 0.3 mL $mmol^{-1}$ pyrrole) and stirred at 0 °C for 30 min. The azide pyrrole (**38**, 1.00 g, 5.66 mmol, 1.00 eq.) in 1,2-dichloroethane (3.40 mL, 0.6 mL $mmol^{-1}$ pyrrole) was added dropwise at 0 °C over 10 min, followed by stirring for 2 h at 0 °C and 2 h at 100 °C under reflux. A solution of sodium acetate (2.79 g, 34.0 mmol, 6.00 eq.) in water (8.50 mL, 4 mol L^{-1}) was added and refluxing was continued for 30 min. After cooling to ambient temperature, the deep brown mixture was diluted with water (50 mL) and extracted with CH_2Cl_2 (3 x 30 mL). Merged organic phases were washed with brine (100 mL), dried over $MgSO_4$, filtered, and the solvent was removed *in vacuo*. Purification of the crude product on silica with CH_2Cl_2/EtOAc (85:15) provided the product as orange oil that crystallized at ambient temperature to give a yellow to orange solid (1.09 g, 5.28 mmol, 93%). R_f (CH_2Cl_2/EtOAc 75:25): 0.50 (UV and $KMnO_4$). Mp 62.1–64.5 °C. ^{1}H NMR (600 MHz, $CDCl_3$, δ): 10.55 (s, 1H, 1-H), 9.28 (s, 1H, 6-H), 6.77 (d, *J*=2.8, 1H, 3-H), 3.29 (t, *J*=5.8, 2H, 11-H), 2.43 (t, *J*=6.5, 2H, 8-H), 2.30 (s, 3H, 13-H), 1.67–1.59 (m, 4H, 9-H, 10-H). ^{13}C NMR (151 MHz, $CDCl_3$, δ): 177.7 (C-6), 136.6 (C-5), 130.6 (C-2), 123.7 (C-4), 122.7 (C-3), 51.4 (C-11), 28.6 (C-9), 27.7 (C-10), 25.2 (C-8), 11.6 (C-13). FT-IR (neat, cm^{-1}): 3167, 3127, 3100, 3066, 3018, 2987, 2944, 2925, 2856, 2174, 2087, 1614, 1567, 1516, 1467, 1441, 1419, 1379, 1345, 1308, 1279, 1254, 1231, 1172, 1144, 1132, 1081, 1053, 1003, 984, 919, 901, 847, 816, 795, 781, 735, 678, 652, 636, 627, 559, 523, 456. HRMS (ESI) *m/z:* $[M + Na]^+$ calcd for $C_{10}H_{14}N_4NaO$, 229.1060; found, 229.1058.

(*Z*)-5-((4-(4-Azidobutyl)-5-methyl-1*H*-pyrrol-2-yl)methylene)-4-methoxy-1,5-dihydro-2*H*-pyrrol-2-one (40)

To a 100 mL Schlenk flask under N_2 atmosphere were added 4-(azidobutyl)-5-methyl-1*H*-pyrrole-2-carbaldehyde (**39**, 1.20 g, 5.83 mmol, 1.00 eq.) and 4-methoxy-3-pyrrolin-2-one (**8**, 1.32 g, 11.7 mmol, 2.00 eq.) and the solids were dissolved in degassed DMSO (20.4 mL, 3.5 mL $mmol^{-1}$ carbaldehyde). Degassed 2 M NaOH (*aq*.) (16.3 mL, 2.8 mL $mmol^{-1}$ carbaldehyde) was added and the reaction was stirred at 60 °C for 24 h. A yellow solid precipitated during the reaction. The product was partitioned between EtOAc (100 mL) and water (50 mL), the phases were separated, and the aqueous phase was extracted with EtOAc (5 x 75 mL). Merged organic phases were washed with water (100 mL) and brine (150 mL), dried over Na_2SO_4, filtered, and the solvent was removed *in vacuo*. The canary yellow crude product was suspended in *n*-heptane and filtered. The filter cake was then washed repeatedly with *n*-heptane, dropwise with *i*Pr_2O/acetone (1:1), and again with *n*-heptane to remove a brown impurity. After drying under high vacuum, the desired product (1.55 g, 5.15 mmol, 88%) was obtained as gold-yellow powder. Mp 126.8–130.6 °C. ^{1}H NMR (300 MHz, $CDCl_3$, δ): 10.77 (brs, 1H, 1-H), 10.26 (brs, 1H, 8-H), 6.27 (s, 1H, 6-H), 6.23 (d, *J*=2.6, 1H, 11-H), 5.10 (d, *J*=1.5, 1H, 3-H), 3.89 (s, 3H, 14-H), 3.28 (t, *J*=6.3, 2H, 19-H), 2.42 (t, *J*=6.7, 2H, 16-H), 2.34 (s, 3H, 15-H), 1.72 – 1.52 (m, *J*=6.6, 3.3, 4H, 17-H, 18-H). ^{13}C NMR (76 MHz, $CDCl_3$, δ): 173.2 (C-2), 168.1 (C-4), 132.2 (C-9), 124.5 (C-7), 122.8 (C-5), 121.7 (C-10), 117.6 (C-11), 102.7 (C-6), 90.2 (C-3), 58.3 (C-14), 51.6 (C-19), 28.6 (C-18), 28.1 (C-17), 25.2 (C-16), 11.5 (C-15). FT-IR (neat, cm^{-1}): 3338, 3167, 3145, 3027, 2937, 2910, 2855, 2083, 1667, 1634, 1569, 1481, 1466, 1437, 1386, 1346, 1311, 1276, 1261, 1221, 1174, 1148, 1119, 1063, 1042, 1006, 973, 870, 824, 808, 790, 780, 737, 716, 673, 641, 631, 556, 546, 510, 493, 473, 457. HRMS (ESI) *m/z:* $[M + H]^+$ calcd for $C_{15}H_{20}N_5O_2$, 302.1612; found, 302.1614.

(*Z*)-5-((4-(4-Azidobutyl)-5-methyl-2*H*-pyrrol-2-ylidene)methyl)-4-methoxy-1*H*-pyrrol-2-yl trifluoromethanesulfonate (41)

In a 250 mL Schlenk flask under N_2 atmosphere, trifluoromethanesulfonic anhydride (402 µL, 2.39 mmol, 1.20 eq., freshly opened**!**) was added dropwise to a solution of dipyrrinone (**40**, 600 mg, 1.99 mmol, 1.00 eq.) in CH_2Cl_2 (39 mL, 19.5 mL $mmol^{-1}$) at 0 °C. After stirring at this temperature for 1 h, the dark brown reaction mixture was poured into 2% $NaHCO_3$ (*aq*., 200 mL), the phases were separated and the aqueous phase was repeatedly extracted with MTBE (3 x 50 mL, solids from the reaction flask were solubilized with a minimal amount of EtOAc). The combined organic layers were washed with brine (200 mL), dried over Na_2SO_4, filtered, and the solvent evaporated *in vacuo*. Before complete removal of the solvent, *n*-pentane was repeatedly added to assist evaporation of MTBE and EtOAc. Thorough solvent removal provided the pure triflate (805 mg, 1.86 mmol, 93%) without the need for purification as a deep brown-yellow oil. ^{1}H NMR (300 MHz, $CDCl_3$, δ): 10.69 (brs, 1H, 1-H), 6.96 (s, 1H, 6-H), 6.50 (s, 1H, 11-H), 5.40 (s, 1H, 3-H), 3.85 (s, 3H, 17-H), 3.33 – 3.24 (m_c, *J*=6.2, 2H, 22-H), 2.46 – 2.36 (m_c, *J*=6.8, 2H, 19-H), 2.29 (s, 3H, 18-H), 1.74 – 1.48 (m, 4H, 20-H, 21-H). ^{13}C NMR (76 MHz, $CDCl_3$, δ): 167.8 (C-4), 160.8 (C-2), 137.9 (C-9), 132.0 (C-5), 127.5 (C-7), 124.6 (C-10), 122.7 (C-11), 121.8 (C-6), 118.8 (q, *J*=321.2, C-15), 87.0 (C-3), 58.8 (C-17), 51.4 (C-22), 28.5 (C-20), 27.7 (C-21), 25.2 (C-19), 12.2 (C-18). ^{19}F NMR (282 MHz, $CDCl_3$, δ): -72.7 (s, –CF_3). FT-IR (neat, cm^{-1}): 3335, 2937, 2861, 2094, 1623, 1552, 1478, 1418, 1371, 1333, 1265, 1234, 1210, 1174, 1130, 1086, 1011, 973, 886, 830, 793, 767, 747, 709, 653, 625, 604, 573, 520, 487. HRMS (ESI) *m/z:* $[M + H]^+$ calcd for $C_{16}H_{19}F_3N_5O_4S$, 434.1104; found, 434.1102.

(*Z*)-5'-((4-(4-Azidobutyl)-5-methyl-1*H*-pyrrol-2-yl)methylene)-4'-methoxy-5-pentyl-1*H*,5'*H*-[2,2'-bipyrrol]-1'-ium chloride (4·HCl)

A 50 mL Schlenk flask with magnetic stirring bar was sequentially charged with $Pd(OAc)_2$ (19.5 mg, 87.0 µmol, 0.05 eq.), SPhos (42.9 mg, 104 µmol, 0.06 eq.), triflate (**41**, 754 mg, 1.74 mmol, 1.00 eq.), alkyl boronic acid (**31**, 987 mg, 3.48 mmol, 2.00 eq.) and evacuated/N_2-refilled three times. Degassed *n*BuOH (9.59 mL) was added, and the mixture was stirred at 20 °C until homogeneous. A solution of K_3PO_4 (739 mg, 3.48 mmol, 2.00 eq., 1.45 M) in degassed H_2O (2.40 mL) was added in one portion to initiate the Suzuki reaction. The reaction vessel was lowered into a pre-heated heating mantle at 60 °C, and the orange solution was stirred under argon for 13 h. The red-orange solution was diluted with CH_2Cl_2 and brine (50 mL), the phases were separated, and the aqueous phase was repeatedly extracted with CH_2Cl_2 (25 mL portions) until the organic phase remained nearly colorless. Merged organic phases were dried over $MgSO_4$, filtered over degreased cotton wool (**!**), and the solvent was removed *in vacuo*. The deep red film was chromatographed on silica using CH_2Cl_2 + 0.7–3% NH_3 (7 N in MeOH) as eluent. Acidification of product fractions with 1 M HCl in Et_2O, immobilization on Celite, and chromatography on neutral aluminium oxide (Brockmann I) with *n*-pentane/EtOAc (0–100% EtOAc) provided the product as purple film. The film was dissolved in a minimal amount of CH_2Cl_2 and acidified again. Repeated treatment with *n*-pentane assisted precipitation of the HCl salt as an amorphous purple powder (367 mg, 803 µmol, 46%) upon solvent evaporation. Mp 112.4–113.2 °C. ^{1}H NMR (300 MHz, $CDCl_3$, δ): 12.60 (brs, 1H, 13-H), 12.51 (brs, 2H, 1-H, 7-H), 6.87 (dd, *J*=3.9, 2.5, 1H, 4-H), 6.85 (s, 1H, 11-H), 6.60 (d, *J*=2.6, 1H, 16-H), 6.10 (dd, *J*=3.9, 2.2, 1H, 3-H), 6.01 (d, *J*=1.8, 1H, 10-H), 3.98 (s, 3H, 23-H), 3.37 – 3.21 (m_c, 2H, 28-H), 2.76 (t, *J*=7.7, 2H, 17-H), 2.52 (s, 3H, 24-H), 2.48 – 2.35 (m_c, 2H, 25-H), 1.86 – 1.71 (m_c, *J*= 6.4, 2H, 18-H), 1.70 – 1.56 (m, 4H, 26-H, 27-H), 1.43 – 1.27 (m, 4H, 19-H, 20-H) 0.89 (m_c, *J*=6.9, 3H, 21-H). ^{13}C NMR (76 MHz, $CDCl_3$, δ): 165.9 (C-9),

148.1 (C-6), 145.3 (C-2), 144.5 (C-14), 126.8 (C-16), 126.5 (C-15), 125.0 (C-12), 121.6 (C-8), 121.0 (C-5), 119.1 (C-4), 114.4 (C-11), 110.8 (C-3), 92.7 (C-10), 58.8 (C-23), 51.4 (C-28), 31.6 (C-19), 28.9 (C-18), 28.6 (C-27), 28.3 (C-17), 27.4 (C-26), 25.0 (C-25), 22.5 (C-20), 14.2 (C-21), 12.3 (C-24). FT-IR (neat, cm^{-1}): 3172, 3146, 3108, 3075, 3013, 2924, 2856, 2095, 1634, 1607, 1579, 1538, 1519, 1495, 1447, 1406, 1348, 1257, 1209, 1168, 1155, 1128, 1046, 997, 972, 893, 882, 841, 813, 794, 732, 696, 651, 618, 601, 546, 504. HRMS (ESI) *m/z:* $[M - Cl]^+$ calcd for $C_{24}H_{33}N_6O$, 421.2710; found, 421.2717.

Bifunctional Alkyne-Maleimide Linker

Methyl 2,5-dioxo-2,5-dihydro-1*H*-pyrrole-1-carboxylate (S25)

A 500 mL Schlenk flask with stirring bar under N_2 atmosphere was charged with maleimide (**S24**, 3.00 g, 30.9 mmol, 1.00 eq.) and the solid dissolved in dry EtOAc (150 mL). Anhydrous *N*-methyl morpholine (NMM, 3.45 mL, 30.9 mmol, 1.00 eq.) was added. The resulting clear solution was cooled to 0 °C and stirred for 20 min. Methyl chloroformate (3.58 mL, 46.4 mmol, 1.50 eq.) was added dropwise at 0 °C, and the faint pink reaction was stirred for 30 min at 0 °C, followed by 2 h at 20 °C. Solids were collected by filtration over a Büchner funnel and washed with EtOAc (100 mL). The filtrate was washed with water (100 mL) and brine (100 mL), dried over $MgSO_4$, filtered, and the solvent removed under reduced pressure. The resulting faint pink crude product was chromatographed on silica using PE/EtOAc (70:30) to provide the product as a white solid (3.57 g, 23.0 mmol, 74%) after solvent evaporation. R_f (PE/EtOAc 50:50): 0.38. Mp 64.5–66.9 °C. ^1H NMR (600 MHz, $CDCl_3$, δ): 6.85 (d, *J*=0.8, 2H, 3-H, 4-H), 3.98 (s, 3H, 11-H). ^{13}C NMR (151 MHz, $CDCl_3$, δ): 165.7 (C-2, C-5), 148.2 (C-8), 135.4 (C-3, C-4), 54.4 (C-11). FT-IR (neat, cm^{-1}): 3190, 3111, 3102, 3091, 2971, 2905, 1801, 1756, 1709, 1596, 1453, 1439, 1391, 1332, 1280, 1256, 1187, 1137, 1103, 1059, 1034, 921, 840, 815, 769, 760, 693, 640, 592, 488. HRMS (ESI) *m/z:* $[M + H]^+$ calcd for $C_6H_6NO_4$, 156.0291; found, 156.0293.

tert-Butyl (2-(2,5-dioxo-2,5-dihydro-1*H*-pyrrol-1-yl)ethyl)carbamate (S27)

A 500 mL round-bottom flask was charged with *tert*-butyl 2-aminoethylcarbamate (**S26**, 3.41 g, 21.3 mmol, 1.10 eq.) and the oil was dissolved in saturated $NaHCO_3$ (*aq.*) (96.7 mL, 5 mL $mmol^{-1}$ maleimide) by stirring for 15 min at 23 °C, followed by stirring at 0 °C for 15 min. Finely ground methyl carbamate (**S25**, 3.00 g, 19.3 mmol, 1.00 eq.) was then added to the clear solution at 0 °C. Stirring at 0 °C was continued for 3 h (white solid precipitates), followed by 30 min at 20 °C. Water (150 mL) was added and the aqueous phase was extracted with EtOAc (3 x 150 mL). Merged organic phases were dried over $MgSO_4$, filtered, and the solvent was removed *in vacuo* to yield a weak pink solid. For chromatography, the solid was dissolved in a minimal amount of EtOAc and then diluted with an equal volume of PE. Chromatography on silica with PE/EtOAc (75:25) provided the product (3.52 g, 14.7 mmol, 76%) as a hygroscopic, fluffy white solid after removal of residual moisture by vacuum desiccation over anhydrous $CaCl_2$ for 10 days. R_f (PE/EtOAc 50:50): 0.42 (UV and $KMnO_4$). Mp 127.2–128.9 °C. ^1H NMR (600 MHz, $CDCl_3$, δ): 6.71 (s, 2H, 3-H, 4-H), 4.73 (s, 1H, 10-H), 3.66 (t, *J*=5.6, 2H, 8-H), 3.33 (q, *J*=5.8, 2H, 9-H), 1.40 (s, 9H, 15-H, 16-H, 17-H). ^{13}C NMR (151 MHz, $CDCl_3$, δ): 170.9 (C-2, C-5), 156.1 (C-11), 134.3 (C-3, C-4), 79.7 (C-14), 39.6 (C-9), 38.2 (C-8), 28.5 (C-15, C-16, C-17). FT-IR (neat, cm^{-1}): 3348, 3087, 2979, 2938, 1701, 1678, 1515, 1434, 1404, 1367, 1361, 1323, 1288, 1252, 1167, 1144, 1103, 1060, 1039, 1014, 943, 877, 844, 784, 761, 739, 691, 642, 612, 574, 536, 492, 464. HRMS (ESI) *m/z:* $[M + H]^+$ calcd for $C_{11}H_{17}N_2O_4$, 241.1183; found, 241.1185.

2-(2,5-Dioxo-2,5-dihydro-1*H*-pyrrol-1-yl)ethan-1-aminium 2,2,2-trifluoroacetate (S28)

The Boc-protected maleimide (**S27**, 3.00 g, 12.5 mmol, 1.00 eq.) was dissolved under N_2 atmosphere in CH_2Cl_2 (31.2 mL, 0.4 M) and cooled to 0 °C. TFA (6.73 mL, 87.4 mmol, 7.00 eq.) was added slowly to the maleimide and the solution was stirred overnight for 20 h at 20 °C. The solvent was removed and the pale-yellow oil taken up in approx. 7 mL CH_2Cl_2 and diluted with Et_2O to precipitate a solid. If clumped, brief sonication was used to give a fine suspended solid, which was then cooled at –20 °C for 3 h. The solid was filtered off, washed with Et_2O, *n*-pentane, and dried under high vacuum to provide the ammonium salt (3.11 g, 12.2 mmol, 98%) as a white hygroscopic solid. Mp 125.9–127.7 °C. ^1H NMR (600 MHz, D_2O, δ): 6.88 (s, 2H, 3-H, 4-H), 3.82 (t, *J*=5.8, 2H, 8-H), 3.21 (t, *J*=5.8, 2H, 9-H). ^{13}C NMR (151 MHz, D_2O, δ): 172.8 (C-2, C-5), 134.8 (C-3, C-4), 38.5 (C-9), 35.1 (C-8). FT-IR (neat, cm^{-1}): 3036, 2926, 2843, 2744, 2653, 2551, 2477, 2436, 1711, 1671, 1626, 1584, 1536, 1465, 1440, 1426, 1413, 1395, 1370, 1331, 1202, 1176, 1156, 1120, 1061, 1041, 1022, 948, 901, 850, 829, 799, 719, 698, 640, 609, 599, 520, 500. HRMS (ESI) *m/z:* $[M - CF_3CO_2^-]^+$ calcd for $C_6H_9N_2O_2$, 141.0659; found, 141.0660.

N-(2-(2,5-Dioxo-2,5-dihydro-1H-pyrrol-1-yl)ethyl)hex-5-ynamide (42)

To a 500 mL Schlenk flask under N_2 atmosphere were added PyBOP (7.47 g, 14.4 mmol, 1.40 eq., freshly opened**!**), anhydrous DIPEA (3.85 mL, 22.6 mmol, 2.20 eq.), and the maleimide trifluoroacetate (**S28**, 3.00 g, 11.79 mmol, 1.15 eq.). The reactants were dissolved in dry CH_2Cl_2 (103 mL, 10 mL mol^{-1} acid) until nearly dissolved. 5-Hexynoic acid (1.15 g, 10.3 mmol, 1.00 eq.) was added, and the bright yellow mixture was stirred for 18 h at 20 °C, after which the solution had become ivory colored. All volatile materials were evaporated under reduced pressure and the residue was dissolved in EtOAc (150 mL). The organic solution was washed with saturated $NaHCO_3$ (3 x 50 mL), dried over $MgSO_4$, filtered, and the solvent was removed *in vacuo*. The orange oil was applied to chromatography on silica with CH_2Cl_2/MeOH (97:3) to yield the product (2.10 g, 8.97 mmol, 87%) as a white solid. R_f (PE/EtOAc 20:80): 0.38 ($KMnO_4$), R_f (CH_2Cl_2/MeOH 90:10): 0.59 ($KMnO_4$). Mp 83.2–86.8 °C. ^1H NMR (300 MHz, $CDCl_3$, δ): 6.66 (s, 2H, 3-H, 4-H), 5.78 (s, 1H, 10-H), 3.69 – 3.55 (m, 2H, 8-H), 3.46 – 3.32 (m, 2H, 9-H), 2.26 – 2.17 (t, *J*=7.3, 2H, 13-H), 2.22 – 2.08 (dt, *J*=6.7, *J*=2.7, 2H, 15-H), 1.90 (t, *J*=2.6, 1H, 17-H), 1.75 (p, *J*=7.1, 2H, 14-H). ^{13}C NMR (76 MHz, $CDCl_3$, δ): 172.7 (C-11), 171.0 (C-2, C-5), 134.4 (C-3, C-4), 83.6 (C-16), 69.3 (C-17), 39.0 (C-9), 37.7 (C-8), 35.0 (C-13), 24.1 (C-14), 17.9 (C-15). FT-IR (neat, cm^{-1}): 3461, 3308, 3263, 3175, 3100, 2968, 2948, 2927, 2913, 2887, 1767, 1743, 1696, 1650, 1600, 1588, 1549, 1459, 1447, 1417, 1387, 1329, 1315, 1269, 1219, 1171, 1108, 1044, 1026, 980, 952, 908, 829, 785, 765, 722, 696, 678, 641, 625, 579, 571, 475. HRMS (ESI) *m/z:* [M + H]$^+$ calcd for $C_{12}H_{15}N_2O_3$, 235.1077; found, 235.1081.

Homo-Dimeric Zinc Complexes of Prodigiosin Azides

Prodigiosin A-Ring Azide Zinc Complex (43)

Under N_2 atmosphere, prodigiosin A-ring azide (**2·HCl**, 333 mg, 751 µmol, 1.00 eq.) and zinc(II) acetate dihydrate (990 mg, 4.51 mmol, 6.00 eq.) were dissolved in MeOH (75 mL). $CHCl_3$ (75 mL) and DIPEA (1.83 mL, 10.5 mmol, 14.0 eq.) were added sequentially and the reaction was stirred at 21 °C for 18 h. The solvent was evaporated and the crude product was dissolved in CH_2Cl_2. Purification over basic aluminium oxide with CH_2Cl_2 eluted the desired product. Starting material remained on the column and was eluted with EtOH. Evaporation of the solvent provided a red solid, which was repeatedly precipitated with *n*-pentane. The EtOH fraction was concentrated *in vacuo* and treated again with DIPEA and $Zn(OAc)_2$ dihydrate in $CHCl_3$/MeOH to allow further product isolation. The process was repeated four times in total. The desired prodigiosin A-ring azide zinc complex (241 mg, 275 µmol, 73%) was obtained as an amorphous red solid after repetitive precipitation with *n*-pentane. The product is soluble in CH_2Cl_2 and partially soluble in PE. R_f (PE/EtOAc 80:20): 0.49 (visible light). Mp 108.4–110.0 °C. ^1H NMR (300 MHz, $CDCl_3$, δ): 9.24 (t, *J*=2.5, 1H, 1-H), 7.09 (s, 1H, 11-H), 6.70 (s, 1H, 16-H), 6.48 (d, *J*=3.5, 2.4, 1H, 4-H), 6.07 (s, 1H, 10-H), 5.81 (dd, *J*=3.6, 2.3, 1H, 3-H), 3.95 (s, 3H, 24-H), 3.24 (t, *J*=7.0, 2H, 21-H), 2.30 (t, *J*=7.6, 2H, 26-H), 2.20 (t, *J*=7.1, 2H, 17-H), 1.88 (s, 3H, 25-H), 1.63 – 1.46 (m, 2H, 20-H), 1.45 (p, *J*=7.4, 2H, 27-H), 1.39 – 1.22 (m, 3H, 18-H, 19-H), 0.91 (t, *J*=7.3, 3H, 28-H). ^{13}C NMR (76 MHz, $CDCl_3$, δ): 165.3 (C-9), 154.2 (C-14), 151.8 (C-6), 137.5 (C-2), 135.0 (C-12), 130.4 (C-15), 129.1 (C-8), 126.6 (C-16), 125.9 (C-5), 119.7 (C-11), 112.8 (C-4), 107.6 (C-3), 94.9 (C-10), 58.2 (C-24), 51.5 (C-21), 29.6 (C-19), 28.7 (C-20), 28.4 (C-26), 27.9 (C-17), 26.5 (C-18), 23.5 (C-27), 14.2 (C-25), 14.1 (C-28). FT-IR (neat, cm^{-1}): 3316, 3100, 3007, 2954, 2927, 2869, 2856, 2092, 1587, 1544, 1533, 1510, 1451, 1393, 1366, 1306, 1263, 1228, 1200, 1180, 1143, 1125, 1094, 1078, 1044, 1016, 992, 979, 909, 846, 825, 803, 784, 767, 737, 714, 672, 655, 632, 594, 558, 524, 513, 498. HRMS (ESI) *m/z:* [M + H]$^+$ calcd for $C_{46}H_{59}N_{12}O_2Zn$, 875.4170; found, 875.4139.

Prodigiosin B-Ring Azide Zinc Complex (44)

Under N_2 atmosphere, prodigiosin B-ring azide (**3·HCl**, 434 mg, 895 µmol, 1.00 eq.) and zinc(II) acetate dihydrate (1.18 g, 5.37 mmol, 6.00 eq.) were dissolved in MeOH (90 mL). $CHCl_3$ (90 mL) and DIPEA (2.18 mL, 12.5 mmol, 14.0 eq.) were added sequentially and the reaction was stirred at 21 °C for 18 h. Celite was added and the solvent was evaporated. The immobilized product was applied to chromatography on silica with PE/CH_2Cl_2 (60:40). After product elution, the column was flushed with EtOAc to elute uncomplexed starting material. Evaporation of the solvent provided a red solid, which was repeatedly precipitated with PE to obtain the desired prodigiosin B-ring azide zinc complex as an amorphous, fluffy red solid. The EtOAc fraction was treated again with DIPEA and $Zn(OAc)_2$ dihydrate in $CHCl_3$/MeOH after solvent evaporation due to partial decomplexation of Zn complex during workup. The process was repeated three times in total. The desired prodigiosin B-ring azide zinc complex was obtained as a fluffy red solid (309 mg, 322 µmol, 72%). The product is soluble in CH_2Cl_2 and PE but rather insoluble in MeOH. R_f (PE/EtOAc 80:20): 0.54 (visible light). Mp 167.1–168.9 °C. ^1H NMR (300 MHz, $CDCl_3$, δ): 9.18 (t, *J*=2.5, 1H, 1-H), 7.06 (s, 1H, 11-H), 6.69 (s, 1H, 16-H), 6.44 (dd, *J*=3.6, 2.4, 1H, 4-H), 6.00 (s, 1H, 10-H), 5.78 (dd, *J*=3.5, 2.3, 1H, 3-H), 4.10 (td, *J*=6.0, 3.4, 2H, 23-H), 3.42 (t, *J*=6.5, 2H, 26-H), 2.28 (t, *J*=7.5, 2H, 29-H), 2.20 – 2.12 (m, 2H, 17-H), 2.02 – 1.91 (m, 2H, 24-H), 1.90 – 1.79 (m, 2H, 25-H), 1.86 (s, 3H, 28-H), 1.59 – 1.43 (m, *J*=7.5, 2H, 30-H), 1.37 – 1.12 (m, *J*=6.8, 6H, 18-H, 19-H, 20-H), 0.89 (t, *J*=7.5, 6H, 21-H, 31-H). ^{13}C NMR (76 MHz, $CDCl_3$, δ): 164.2 (C-9), 154.0 (C-14), 152.0 (C-6), 138.2 (C-2), 135.0 (C-12), 130.3 (C-15), 129.3 (C-8), 126.6 (C-16), 125.8 (C-5), 119.6 (C-11), 112.8 (C-4), 107.4 (C-

3), 95.3 (C-10), 70.3 (C-23), 51.3 (C-26), 31.8 (C-19), 29.8 (C-18), 28.5 (C-29), 28.1 (C-17), 26.6 (C-24), 25.9 (C-25), 23.5 (C-30), 22.6 (C-20), 14.3 (C-28), 14.3 (C-21), 14.1 (C-31). FT-IR (neat, cm^{-1}): 3312, 3168, 3105, 2954, 2927, 2869, 2857, 2091, 1587, 1540, 1528, 1509, 1452, 1435, 1389, 1358, 1305, 1257, 1227, 1192, 1144, 1126, 1076, 1040, 1018, 990, 978, 901, 855, 848, 827, 804, 767, 738, 692, 664, 632, 564, 511, 486, 474. HRMS (ESI) *m/z:* $[M + H]^+$ calcd for $C_{52}H_{70}N_{12}O_2Zn$, 958.5031; found, 958.5013.

Prodigiosin C-Ring Azide Zinc Complex (45)

Under N_2 atmosphere, prodigiosin C-ring azide (**4·HCl**, 227 mg, 497 µmol, 1.00 eq.) and zinc(II) acetate dihydrate (654 mg, 2.98 mmol, 6.00 eq.) were dissolved in MeOH (50 mL). $CHCl_3$ (50 mL) and DIPEA (1.21 mL, 6.95 mmol, 14.0 eq.) were added sequentially and the reaction was stirred at 21 °C for 18 h. Celite was added and the solvent was evaporated. The immobilized product was applied to chromatography on silica with PE/CH_2Cl_2 (60:40). After product elution, the column was flushed with EtOAc to elute uncomplexed starting material. Evaporation of the solvent provided a red solid, which was repeatedly precipitated with PE. The EtOAc fraction was treated again with DIPEA and $Zn(OAc)_2$ dihydrate in $CHCl_3$/MeOH after solvent evaporation due to partial decomplexation of Zn complex during workup. The process was repeated three times in total. The desired prodigiosin C-ring azide zinc complex was obtained as a fine powdered red solid (163 mg, 180 µmol, 72%) that is soluble in CH_2Cl_2, less soluble in MeOH, and rather insoluble in PE. R_f (PE/EtOAc 80:20): 0.54 (visible light). Mp 127.8–129.5 °C. ^1H NMR (300 MHz, $CDCl_3$, δ): 9.19 (t, *J*=2.3, 1H, 1-H), 7.05 (s, 1H, 11-H), 6.65 (s, 1H, 16-H), 6.47 (dd, *J*=3.6, 2.6, 1H, 4-H), 6.04 (s, 1H, 10-H), 5.78 (dd, *J*=3.5, 2.4, 1H, 3-H), 3.93 (s, 3H, 23-H), 3.28 – 3.17 (m, 2H, 28-H), 2.38 – 2.28 (m, 2H, 25-H), 2.20 – 2.11 (m, 2H, 17-H), 1.84 (s, 3H, 24-H), 1.64 – 1.50 (m_c, 4H, 26-H, 27-H), 1.34 – 1.12 (m, 6H, 18-H, 19-H, 20-H), 0.88 (t, *J*=6.9, 3H, 21-H). ^{13}C NMR (76 MHz, $CDCl_3$, δ): 165.6 (C-9), 153.2 (C-14), 152.4 (C-6), 138.5 (C-2), 134.9 (C-12), 129.5 (C-8), 129.1 (C-15), 126.2 (C-16), 125.7 (C-5), 119.7 (C-11), 113.2 (C-4), 107.5 (C-3), 95.0 (C-10), 58.2 (C-23), 51.5 (C-28), 31.8 (C-19), 29.8 (C-18), 28.7 (C-27), 28.1 (C-17), 27.4 (C-26), 25.9 (C-25), 22.6 (C-20), 14.2 (C-21), 14.2 (C-24). FT-IR (neat, cm^{-1}): 3320, 2957, 2929, 2870, 2855, 2089, 1590, 1549, 1510, 1454, 1423, 1393, 1366, 1303, 1267, 1232, 1200, 1180, 1147, 1129, 1117, 1086, 1075, 1063, 1044, 1024, 992, 976, 909, 848, 831, 815, 798, 786, 769, 735, 715, 671, 633, 595, 567, 558, 517, 495, 468, 459. HRMS (ESI) *m/z:* $[M + H]^+$ calcd for $C_{48}H_{62}N_{12}O_2Zn$, 902.4405; found, 902.4388.

(*Z*)-5-(5-(4-(4-((2-(2,5-Dioxo-2,5-dihydro-1*H*-pyrrol-1-yl)ethyl)amino)-4-oxobutyl)-1*H*-1,2,3-triazol-1-yl)pentyl)-4'-methoxy-5'-((5-methyl-4-propyl-1*H*-pyrrol-2-yl)methylene)-1*H*,5'*H*-[2,2'-bipyrrol]-1'-ium chloride (46)

To a 100 mL round-bottom flask under N_2 atmosphere, prodigiosin A-ring azide zinc complex (**43**, 205 mg, 234 µmol, 1.00 eq., final 5 mM) and alkyne-maleimide linker (**42**, 274 mg, 1.17 mmol, 5.00 eq.) were added and dissolved in CH_2Cl_2 (15.6 mL). EtOH (15.2 mL) was added to dissolve the linker. Solutions of TDETA (4.29 mL, 122 mg, 258 µmol, 1.10 eq., 60 mM in EtOH) and sodium ascorbate (703 µL, 69.6 mg, 351 µmol, 1.50 eq., 500 mM in water) were added. The reaction was started by the addition of a freshly prepared solution of $Cu(OAc)_2$ (11.7 mL, 42.5 mg, 234 µmol, 1.00 eq., 20 mM in EtOH) and was stirred for 60 min at 21 °C (final ratio of CH_2Cl_2/EtOH 1:2). The reaction was diluted with CH_2Cl_2 (100 mL) and washed with 1 M aqueous HCl (100 mL) to disrupt the zinc complex. Phases were separated and the aqueous phase was extracted with CH_2Cl_2 (15 mL portions) until the organic phase was weakly pink. Merged organic phases were then washed with brine (2 x 100 mL), dried over anhydrous Na_2SO_4, and filtered over degreased cotton wool (**!**). After solvent evaporation, the purple residue was purified by column chromatography on silica with a gradient of CH_2Cl_2/MeOH (0–3% MeOH with 1% iterations, then 6% and 10%). The red product fractions eluted at 3–6% MeOH. The solvent was removed *in vacuo* and the residue purified on silica with isocratic elution in CH_2Cl_2/MeOH (97:3). After solvent evaporation, the residue was dissolved in CH_2Cl_2 and repeatedly precipitated with *n*-pentane. After drying under high vacuum, the desired product was yielded as a deep purple solid (254 mg, 93.6 µmol, 80%). R_f (CH_2Cl_2/MeOH 90:10): 0.44 (visible light). Mp 58.5–62.3 °C. ^1H NMR (300 MHz, $CDCl_3$, δ): 12.50 (brs, 1H, 13-H), 12.46 (brs, 1H, 1-H), 12.40 (brs, 1H, 7-H), 7.47 (s, 1H, 26-H), 6.88 (s, 1H, 11-H), 6.85 (dd, *J*=3.9, 2.1, 1H, 4-H), 6.64 (s, 2H, 37-H, 38-H), 6.63 (d, *J*=2.8, 1H, 16-H), 6.55 (t, *J*=5.9, 1H, 32-H), 6.07 (dd, *J*=4.0, 1.9, 1H, 3-H), 6.04 (d, *J*=1.3, 1H, 10-H), 4.33 (t, *J*=7.0, 2H, 21-H), 3.99 (s, 3H, 43-H), 3.70 – 3.61 (m, 2H, 34-H), 3.48 – 3.38 (m, 2H, 33-H), 2.75 (t, *J*=7.5, 2H, 17-H), 2.67 (t, *J*=7.0, 2H, 27-H), 2.50 (s, 3H, 44-H), 2.36 (t, *J*=7.5, 2H, 45-H), 2.15 (t, *J*=7.2, 2H, 29-H), 2.02 – 1.76 (m, *J*=7.5, 7.4, 6H, 18-H, 19-H, 28-H), 1.55 (h, *J*=7.4, 2H, 46-H), 1.45 – 1.26 (m, 2H, 20-H), 0.92 (t, *J*=7.3, 3H, 47-H). ^{13}C NMR (76 MHz, $CDCl_3$, δ): 173.4 (C-30), 171.0 (C-36, C-39), 165.9 (C-9), 147.7 (C-6), 147.1 (C-25), 145.5 (C-14), 143.7 (C-2), 134.3 (C-37, C-38), 127.8 (C-15), 127.7 (C-16), 125.0 (C-12), 121.6 (C-26), 121.2 (C-5, C-8), 118.7 (C-4), 114.9 (C-11), 110.9 (C-3), 92.7 (C-10), 58.8 (C-43), 49.9 (C-21), 38.5 (C-33), 37.8 (C-34), 35.3 (C-29), 29.9 (C-19), 28.4 (C-18), 27.9 (C-17), 27.6 (C-45), 25.8 (C-20), 25.4 (C-28), 24.4 (C-27), 23.5 (C-46), 13.9 (C-47), 12.4 (C-44). FT-IR (neat, cm^{-1}): 3173, 3112, 3072, 2931, 2864, 1705, 1631, 1602, 1577, 1537, 1495, 1436, 1404, 1355, 1253, 1208, 1152, 1131, 1097, 1080, 1043, 994, 969, 895, 884, 823, 805, 782, 734, 695, 648, 623. HRMS (ESI) *m/z:* $[M - Cl]^+$ calcd for $C_{35}H_{45}N_8O_4$, 641.3558; found, 641.3570.

(*Z*)-4'-(4-(4-(4-((2-(2,5-Dioxo-2,5-dihydro-1*H*-pyrrol-1-yl)ethyl)amino)-4-oxobutyl)-1*H*-1,2,3-triazol-1-yl)butoxy)-5'-((5-methyl-4-propyl-1*H*-pyrrol-2-yl)methylene)-5-pentyl-1*H*,5'*H*-[2,2'-bipyrrol]-1'-ium chloride (47)

To a 100 mL round-bottom flask under N_2 atmosphere, prodigiosin B-ring azide zinc complex (**44**, 130 mg, 135 µmol, 1.00 eq., final 5 mM) and alkyne-maleimide linker (**42**, 159 mg, 677 µmol, 5.00 eq.) were added and dissolved in CH_2Cl_2 (9.02 mL). EtOH (8.79 mL) was added to dissolve the linker. Solutions of TDETA (2.48 mL, 70.5 mg, 149 µmol, 1.10 eq., 60 mM in EtOH) and sodium ascorbate (406 µL, 40.2 mg, 203 µmol, 1.50 eq., 500 mM in water) were added. The reaction was started by the addition of a freshly prepared solution of $Cu(OAc)_2$ (6.77 mL, 24.9 mg, 135 µmol, 1.00 eq., 20 mM in EtOH) and was stirred for 60 min at 21 °C (final ratio of CH_2Cl_2/EtOH 1:2). The reaction was diluted with CH_2Cl_2 (100 mL) and washed with 1 M aqueous HCl (100 mL) to disrupt the zinc complex. Phases were separated and the aqueous phase was extracted with CH_2Cl_2 (15 mL portions) until the organic phase was weakly pink. Merged organic phases were then washed with brine (2 x 100 mL), dried over anhydrous Na_2SO_4, and filtered over degreased cotton wool (**!**). After solvent evaporation, the purple residue was purified by column chromatography on silica with a gradient of CH_2Cl_2/MeOH (0–4% MeOH, 1% iterations). The red product fractions eluted at 3% MeOH. The solvent was removed *in vacuo* and the residue purified on silica with isocratic elution in CH_2Cl_2/MeOH (96:4). After solvent evaporation, the residue was dissolved in CH_2Cl_2 and repeatedly precipitated with *n*-pentane. After drying under high vacuum, the desired product was yielded as a deep purple solid (145 mg, 50.3 µmol, 74%). R_f (CH_2Cl_2/MeOH 90:10): 0.54 (visible light). Mp 49.3–52.3 °C. ^{1}H NMR (300 MHz, $CDCl_3$, δ): 12.59 (brs, 1H, 13-H), 12.49 (brs, 2H, 1-H, 7-H), 7.36 (s, 1H, 31-H), 6.87 – 6.82 (m, 2H, 4-H, 11-H), 6.69 (s, 2H, 42-H, 43-H), 6.66 (s, 1H, 16-H), 6.21 (t, *J*=5.2, 1H, 37-H), 6.10 (d, *J*=3.8, 1H, 3-H), 5.97 (s, 1H, 10-H), 4.42 (t, *J*=6.9, 2H, 26-H), 4.14 (t, *J*=6.1, 2H, 23-H), 3.70 – 3.63 (m, 2H, 39-H), 3.48 – 3.40 (m, 2H, 38-H), 2.76 (t, *J*=7.7, 2H, 17-H), 2.72 (t, *J*=7.4, 2H, 32-H), 2.52 (s, 3H, 47-H), 2.37 (t, *J*=7.5, 2H, 48-H), 2.18 (t, *J*=7.2, 2H, 34-H), 2.18 – 2.05 (m, 2H, 25-H), 1.93 (p, *J*=7.2, 2H, 33-H), 1.92 – 1.82 (m, 2H, 24-H), 1.77 (p, *J*=7.4, 2H, 18-H), 1.56 (h, *J*=7.4, 2H, 49-H), 1.42 – 1.27 (m, 4H, 19-H, 20-H), 0.93 (t, *J*=7.3, 3H, 50-H), 0.92 – 0.84 (m_c, *J*=6.8, 3H, 21-H). ^{13}C NMR (76 MHz, $CDCl_3$, δ): 173.1 (C-35), 171.0 (C-41, C-44), 164.4 (C-9), 147.74 (C-30), 147.70 (C-6), 145.5 (C-14), 145.1 (C-2), 134.3 (C-42, C-43), 127.7 (C-15), 127.5 (C-16), 125.0 (C-12), 121.3 (C-8), 121.1 (C-31), 120.9 (C-5), 118.9 (C-4), 114.5 (C-11), 110.7 (C-3), 93.1 (C-10), 70.8 (C-23), 49.7 (C-26), 38.8 (C-38), 37.8 (C-39), 35.5 (C-34), 31.6 (C-19), 28.9 (C-18), 28.3 (C-17), 27.6 (C-48), 26.9 (C-25), 26.0 (C-24), 25.4 (C-33), 24.7 (C-32), 23.5 (C-49), 22.5 (C-20), 14.2 (C-21), 13.9 (C-50), 12.4 (C-47). FT-IR (neat, cm^{-1}): 3167, 3111, 3064, 2953, 2927, 2869, 2859, 1706, 1631, 1602, 1576, 1538, 1495, 1435, 1405, 1389, 1353, 1338, 1250, 1202, 1151, 1131, 1099, 1079, 1043, 1000, 968, 888, 849, 822, 806, 784, 734, 695, 661, 623, 600, 568, 550, 502, 474, 467, 457. HRMS (ESI) *m/z:* $[M - Cl]^+$ calcd for $C_{38}H_{51}N_8O_4$, 683.4028; found, 683.4028.

(*Z*)-5'-((4-(4-(4-(4-((2-(2,5-Dioxo-2,5-dihydro-1*H*-pyrrol-1-yl)ethyl)amino)-4-oxobutyl)-1*H*-1,2,3-triazol-1-yl)butyl)-5-methyl-1*H*-pyrrol-2-yl)methylene)-4'-methoxy-5-pentyl-1*H*,5'*H*-[2,2'-bipyrrol]-1'-ium chloride (48)

To a 100 mL round-bottom flask under N_2 atmosphere, prodigiosin C-ring azide zinc complex (**45**, 100 mg, 111 µmol, 1.00 eq., final 5 mM) and alkyne-maleimide linker (**42**, 129 mg, 553 µmol, 5.00 eq.) were added and were dissolved in CH_2Cl_2 (5.50 mL). EtOH (9.00 mL) was added to dissolve the linker. A solution of TDETA (2.03 mL, 57.6 mg, 122 µmol, 1.10 eq., 60 mM in EtOH) and sodium ascorbate (332 µL, 32.9 mg, 166 µmol, 1.50 eq., 500 mM in water) were added. The reaction was started by the addition of a freshly prepared solution of $Cu(OAc)_2$ (5.53 mL, 20.1 mg, 111 µmol, 1.00 eq., 20 mM in EtOH) and was stirred for 60 min at 21 °C (final ratio of CH_2Cl_2/EtOH 1:3). The reaction was diluted with CH_2Cl_2 (100 mL) and washed with 1 M aqueous HCl (100 mL) to disrupt the zinc complex. Phases were separated and the aqueous phase was extracted with CH_2Cl_2 (15 mL portions) until the organic phase was weakly pink. Merged organic phases were then washed with brine (2 x 100 mL), dried over anhydrous Na_2SO_4, and filtered over degreased cotton wool (**!**). After solvent evaporation, the purple residue was purified by column chromatography on silica with a gradient of CH_2Cl_2/MeOH (0–3% MeOH with 1% iterations). The red product fractions eluted at 3% MeOH. The solvent was removed *in vacuo* and the residue purified on silica with isocratic elution in CH_2Cl_2/MeOH (97:3). After solvent evaporation, the residue was dissolved in CH_2Cl_2 and repeatedly precipitated with *n*-pentane. After drying under high vacuum, the desired product was yielded as a deep purple solid (123 mg, 44.3 µmol, 80%). R_f (CH_2Cl_2/MeOH 90:10): 0.52 (visible light). Mp 55.3–64.5 °C (broad transition). ^{1}H NMR (300 MHz, $CDCl_3$, δ): 12.57 (brs, 1H, 13-H), 12.50 (brs, 2H, 1-H, 7-H), 7.29 (s, 1H, 33-H), 6.88 (dd, *J*=3.9, 2.3, 1H, 4-H), 6.84 (s, 1H, 11-H), 6.68 (s, 2H, 44-H, 45-H), 6.56 (d, *J*=2.5, 1H, 16-H), 6.30 (t, *J*=6.0, 1H, 39-H), 6.11 (dd, *J*=3.9, 2.1, 1H, 3-H), 6.02 (d, *J*=1.7, 1H, 10-H), 4.32 (t, *J*=7.1, 2H, 28-H), 3.99 (s, 3H, 23-H), 3.71 – 3.63 (m, 2H, 41-H), 3.50 – 3.40 (m, 2H, 40-H), 2.76 (t, *J*=7.7, 2H, 17-H), 2.72 (t, *J*=7.1, 2H, 34-H), 2.49 (s, 3H, 24-H), 2.44 (t, *J*=7.4, 2H, 25-H), 2.18 (t, *J*=7.2, 2H, 36-H), 1.94 (p, *J*=7.2, 2H, 35-H), 1.96 – 1.83 (m, 2H, 27-H), 1.78 (pd, *J*=7.3, 2H, 18-H), 1.55 (p, *J*=7.5, 2H, 26-H), 1.41 – 1.27 (m, 4H, 19-H, 20-H), 0.99 – 0.77 (m_c, *J*=6.8, 3H, 21-H). ^{13}C NMR (76 MHz, $CDCl_3$, δ): 173.3 (C-37), 171.0 (C-43, C-46), 165.9 (C-9), 148.3 (C-6), 147.4 (C-32), 145.5 (C-2), 144.3 (C-14), 134.3 (C-44, C-45), 126.7 (C-16), 126.0 (C-15), 124.9 (C-12), 121.8 (C-8), 121.0 (C-5), 120.9 (C-33), 119.3 (C-4), 114.4 (C-11), 110.9 (C-3), 92.8 (C-10), 58.8 (C-23), 50.1 (C-28), 38.7 (C-40), 37.8 (C-41), 35.4 (C-36), 31.6 (C-19), 29.9 (C-27), 28.9 (C-18), 28.3 (C-17), 27.2 (C-26), 25.5 (C-35), 24.8 (C-25), 24.6 (C-34), 22.5 (C-20), 14.2 (C-21), 12.3 (C-24). FT-IR (neat, cm^{-1}): 3169, 3112, 3067, 2929, 2859, 1706,

1631, 1603, 1578, 1538, 1495, 1436, 1404, 1357, 1253, 1206, 1162, 1132, 1118, 1044, 994, 969, 895, 884, 839, 822, 797, 787, 734, 695, 650, 624, 503. HRMS (ESI) *m/z:* [M – Cl]+ calcd for $C_{36}H_{47}N_8O_4$, 655.3715; found, 655.3723.

(*Z*)-5'-((5-Methyl-4-propyl-1*H*-pyrrol-2-yl)methylene)-4'-(4-(4-(15-oxo-19-((3a*S*,4*S*,6a*R*)-2-oxohexahydro-1*H*-thieno[3,4-*d*]imidazol-4-yl)-2,5,8,11-tetraoxa-14-azanonadecyl)-1*H*-1,2,3-triazol-1-yl)butoxy)-5-pentyl-1*H*,5'*H*-[2,2'-bipyrrol]-1'-ium chloride (50)

To a 25 mL round-bottom flask, prodigiosin B-ring azide zinc complex (**44**, 48.5 mg, 50.5 µmol, 1.00 eq.), biotin-PEG$_4$-acetylene (53.5 mg, 111 µmol, 2.20 eq., 95%), and TDETA (25.1 mg, 53.0 µmol, 1.05 eq.) were added, and the solids were dissolved in $CHCl_3$ (2.00 mL). Subsequently, EtOH (1.48 mL) and solutions of $Cu(OAc)_2$ (2.52 mL, 9.17 mg, 50.5 µmol, 1.00 eq., 20 mM in EtOH) and sodium ascorbate (151 µL, 15.0 mg, 75.7 µmol, 1.50 eq., 500 mM in water) were added. The reaction was stirred at 20 °C for 110 min in a final ratio of $CHCl_3$/EtOH (1:2). The purple reaction solution was diluted with $CHCl_3$ (30 mL) and washed with 1 M HCl (30 mL) to disrupt the zinc complex. The aqueous phase was extracted with $CHCl_3$ (3 x 3 mL), and the merged organic phases were dried over $MgSO_4$ and filtered. After solvent evaporation, the purple crude product was chromatographed on silica with a gradient of CH_2Cl_2/MeOH (0–30% MeOH, with 5%, 10%, 15%, 20% and 30% steps), followed by a second chromatographic separation on silica with CH_2Cl_2/MeOH (0% MeOH, then 20% MeOH). After solvent removal, the biotinylated B-ring prodiginine was obtained as a purple film (82.9 mg, 22.0 µmol, 87%). R_f (CH_2Cl_2/MeOH 70:30): 0.86 (visible light and $KMnO_4$). ^{1}H NMR (600 MHz, $CDCl_3$, δ): 12.56 (brs, 1H, 13-H), 12.45 (brs, 2H, 1-H, 7-H), 7.64 (s, 1H, 31-H), 6.88 (t, *J*=5.4, 1H, 45-H), 6.84 (dd, *J*=4.0, 2.1, 1H, 4-H), 6.82 (s, 1H, 11-H), 6.70 (brs, 1H, 56-H), 6.66 – 6.63 (m, 1H, 16-H), 6.07 (dd, *J*=3.9, 2.2, 1H, 3-H), 5.99 (d, *J*=1.9, 1H, 10-H), 5.78 (brs, 1H, 59-H), 4.66 (s, 2H, 32-H), 4.46 (dd, *J*=7.5, 4.9, 1H, 55-H), 4.44 (t, *J*=7.0, 2H, 26-H), 4.26 (dd, *J*=7.8, 4.6, 1H, 60-H), 4.13 (t, *J*=6.2, 2H, 23-H), 3.70 – 3.65 (m, 2H), 3.65 – 3.62 (m, 2H), 3.61 (s, 4H), 3.60 – 3.56 (m, 4H), 3.52 (t, *J*=5.1, 2H, 43-H), 3.43 – 3.31 (m, 2H, 44-H), 3.08 (td, *J*=7.4, 4.5, 1H, 52-H), 2.85 (dd, *J*=12.8, 4.9, 1H, 54a-H), 2.73 (t, *J*=7.8, 2H, 17-H), 2.70 (d, *J*=12.7, 1H, 54b-H), 2.49 (s, 3H, 61-H), 2.34 (t, *J*=7.5, 2H, 62-H), 2.18 (t, *J*=7.5, 2H, 48-H), 2.10 (p, *J*=7.2, 2H, 25-H), 1.89 – 1.82 (m, 2H, 24-H), 1.79 – 1.72 (m_c, 2H, 18-H), 1.73 – 1.65 (m, 1H, 51a-H), 1.66 – 1.57 (m, *J*=7.4, 3H, 49-H, 51b-H), 1.53 (h, *J*=7.3, 2H, 63-H), 1.43 – 1.34 (m, 2H, 50-H), 1.36 – 1.29 (m, 4H, 19-H, 20-H), 0.91 (t, *J*=7.3, 3H, 64-H), 0.89 – 0.84 (m_c, *J*=6.7, 3H, 21-H). ^{13}C NMR (151 MHz, $CDCl_3$, δ): 173.5 (C-46), 164.4 (C-9), 164.1 (C-57), 147.7 (C-6), 145.27 (C-30), 145.25 (C-14), 144.9 (C-2), 127.6 (C-15), 127.4 (C-16), 124.9 (C-12), 122.8 (C-31), 121.2 (C-8), 120.9 (C-5), 118.9 (C-4), 114.4 (C-11), 110.6 (C-3), 93.1 (C-10), 70.8 (C-23), 70.58, 70.57, 70.48, 70.42, 70.13, 69.93, 69.86, 64.7 (C-32), 61.8 (C-60), 60.3 (C-55), 55.7 (C-52), 49.8 (C-26), 40.6 (C-54), 39.2 (C-44), 35.9 (C-48), 31.5 (C-19), 28.8 (C-18), 28.23 (C-17), 28.22 (C-50), 28.1 (C-51), 27.5 (C-62), 26.9 (C-25), 25.9 (C-24), 25.7 (C-49), 23.45 (C-63), 22.43 (C-20), 14.1 (C-21), 13.9 (C-64), 12.3 (C-61). FT-IR (neat, cm^{-1}): 3221, 3172, 3134, 3112, 3072, 2925, 2862, 1699, 1664, 1631, 1603, 1577, 1539, 1404, 1354, 1338, 1309, 1253, 1203, 1130, 1083, 1044, 1001, 970, 887, 845, 806, 783, 735, 683, 661, 624, 598, 566, 550, 545, 503, 486, 478, 469, 463. HRMS (ESI) *m/z:* [M – Cl]+ calcd for $C_{47}H_{72}N_9O_7S$, 906.5270; found, 906.5265.

1-(1*H*-Pyrrol-2-yl)pent-4-en-1-one (S14)

Under N_2 atmosphere, a solution of 4-pentenoic acid (**S12**, 7.07 g, 70.6 mmol, 1.00 eq.), 2,2'-dipyridyl disulfide (DPS, 20.2 g, 91.8 mmol, 1.30 eq.), and triphenylphosphine (24.1 g, 91.8 mmol, 1.30 eq.) in dry toluene (90 mL) was stirred at 25 °C for 4 h for activation of the carboxylic acid. The reaction mixture was then cooled to –78 °C and a solution of pyrrolylmagnesium chloride [formed by deprotonation of 1*H*-pyrrole (19.6 mL, 283 mmol, 4.00 eq.) with methylmagnesium chloride (3 M in THF, 70.6 mL, 212 mmol, 3.00 eq.) in dry toluene (400 mL) at –40 °C, stirred for at least 20 min] was introduced with a transfer cannula. After stirring for 1 h at –78 °C, the reaction was quenched with saturated NH_4Cl at –78 °C, and the aqueous layer was repeatedly extracted with MTBE (3 x 200 mL). The organic phase was successively washed with 5% K_2CO_3 (250 mL), water (400 mL), and brine (400 mL). After drying over Na_2SO_4 and concentration under reduced pressure, chromatography over silica (*n*-pentane/EtOAc 98:2) afforded the product (8.49 g, 56.9 mmol, 81%) as a colorless syrup that crystallized at –20 °C. R_f (PE/EtOAc 80:20): 0.37 (UV and $KMnO_4$). Mp 33.3–36.4 °C. ^{1}H NMR (600 MHz, $CDCl_3$, δ): 9.41 (brs, 1H, 1-H), 7.03 (td, *J*=2.8, 1.4, 1H, 5-H), 6.92 (ddd, *J*=4.0, 2.6, 1.4, 1H, 3-H), 6.28 (dt, *J*=3.8, 2.5, 1H, 4-H), 5.88 (ddt, *J*=16.8, 10.2, 6.5, 1H, 10-H), 5.08 (dq, *J*=17.1, 1.6, 1H, 11a-H), 5.00 (dq, *J*=10.2, 1.3, 1H, 11b-H), 2.87 (dd, *J*=7.4, 6.8, 2H, 8-H), 2.48 (qt, *J*=7.3, 1.3, 2H, 9-H). ^{13}C NMR (151 MHz, $CDCl_3$, δ): 190.0 (C-6), 137.5 (C-10), 132.1 (C-2), 124.5 (C-5), 116.1 (C-3), 115.4 (C-11), 110.8 (C-4), 37.2 (C-8), 29.0 (C-9). FT-IR (neat, cm^{-1}): 3280, 3079, 2978, 2911, 1737, 1632, 1545, 1426, 1402, 1318, 1297, 1244, 1136, 1113, 1099, 1043, 999, 836, 748, 606, 514, 505, 462. HRMS (ESI) *m/z:* [M + H]+ calcd for $C_9H_{12}NO$, 150.0913; found, 150.0916.

2-(Pent-4-en-1-yl)-1*H*-pyrrole (S2)

Under N_2 atmosphere, $NaBH_4$ (4.97 g, 131 mmol, 2.80 eq.) was suspended in dry *i*PrOH (150 mL). The acylated pyrrole (**S14**, 7.00 g, 46.9 mmol, 1.00 eq.) was dissolved in dry *i*PrOH (200 mL) and slowly added to the $NaBH_4$ suspension at 0 °C. Afterwards, the solution was refluxed for 22 h at 98 °C. The reaction mixture was cooled to 0 °C, quenched with saturated NH_4Cl, and extracted with EtOAc (3 x 150 mL). The merged organic phases were washed with water (2 x 150 mL) and dried over Na_2SO_4. After solvent evaporation, the crude product was purified over silica using PE/Et_2O (98:2) + 0.5% TEA and obtained as an instable volatile colorless syrup (4.97 g, 36.7 mmol, 78%) that darkened over time at −20 °C under argon. R_f (PE/EtOAc 80:20): 0.64 (UV and $KMnO_4$). ^{1}H NMR (600 MHz, $CDCl_3$, δ): 7.91 (brs, 1H, 1-H), 6.69 (q, *J*=2.5, 1.6, 1H, 5-H), 6.18 (q, *J*=3.2, 2.6, 1H, 4-H), 5.97 (t, *J*=3.2, 1H, 3-H), 5.87 (ddt, *J*=16.9, 10.0, 6.7, 1H, 9-H), 5.08 (dq, *J*=17.1, 1.7, 1H, 10a-H), 5.03 (dt, *J*=10.3, 1.6, 1H, 10b-H), 2.65 (t, *J*=7.8, 2H, 6-H), 2.16 (q, *J*=7.5, 6.6, 2H, 8-H), 1.76 (p, *J*=7.5, 2H, 7-H). ^{13}C NMR (151 MHz, $CDCl_3$, δ): 138.5 (C-9), 132.5 (C-2), 116.2 (C-5), 115.0 (C-10), 108.4 (C-4), 105.1 (C-3), 33.4 (C-8), 28.9 (C-7), 27.1 (C-6). FT-IR (neat, cm^{-1}): 3380, 3096, 3076, 2995, 2976, 2931, 2856, 1640, 1567, 1472, 1456, 1439, 1428, 1403, 1347, 1335, 1304, 1292, 1117, 1094, 1025, 992, 958, 911, 884, 787, 713, 644, 639, 575, 564, 557, 547, 537, 526, 514, 509, 502. HRMS (ESI) *m/z:* $[M + H]^+$ calcd for $C_9H_{14}N$, 136.1121; found, 136.1127.

5-Chloro-1-(1*H*-pyrrol-2-yl)pentan-1-one (S3)

Under N_2 atmosphere, 1*H*-pyrrole (8.95 mL, 129 mmol, 2.00 eq.) was dissolved in dry toluene (260 mL). Methylmagnesium chloride (22.8 mL, 67.7 mmol, 1.05 eq., 2.97 M in THF) was introduced at 21 °C and the mixture was stirred for 60 min at this temperature before the pyrrolylmagnesium chloride was cooled to −40 °C for 30 min. 5-Chloropentanoyl chloride (**S15**, 8.29 mL, 64.5 mmol, 1.00 eq.) in dry toluene (65 mL) was cooled to −78 °C and the pyrrolylmagnesium chloride was added to the acid chloride with a transfer cannula. After stirring for 3 h at −78 °C, the green-brown solution was allowed to warm to 21 °C and stirred for a further 24 h. The reaction was quenched at 0 °C with saturated NH_4Cl, causing a color shift to dark green. After phase separation, the aqueous layer was repeatedly extracted with MTBE (3 x 150 mL). The brown organic phase was successively washed with 5% K_2CO_3, water, and brine (each 250 mL), and dried over $MgSO_4$. Concentration under reduced pressure provided a viscous brown liquid that slowly crystallized at −20 °C. Purification was achieved on silica with *n*-pentane/CH_2Cl_2/MTBE (85:8:7) and a long column of 50 cm to give the product (6.19 g, 33.3 mmol, 52%) as a weak brown solid. Separation of two product isomers (probably 2- and 3-acylated pyrrole) is **only** possible in the presence of 5–10% CH_2Cl_2. All two-component solvent mixtures failed in separation. R_f (*n*-pentane/CH_2Cl_2/MTBE 60:30:10): 0.32 (UV and anisaldehyde). Mp 58.9–60.4 °C. ^{1}H NMR (600 MHz, $CDCl_3$, δ): 9.36 (brs, 1H, 1-H), 7.03 (td, *J*=2.7, 1.3, 1H, 5-H), 6.91 (ddd, *J*=3.8, 2.5, 1.3, 1H, 3-H), 6.28 (dt, *J*=3.8, 2.6, 1H, 4-H), 3.57 (t, *J*=6.1, 2H, 11-H), 2.81 (t, *J*=6.8, 2H, 8-H), 1.93 – 1.82 (m, 4H, 9-H, 10-H). ^{13}C NMR (151 MHz, $CDCl_3$, δ): 190.2 (C-6), 132.0 (C-2), 124.6 (C-5), 116.1 (C-3), 110.9 (C-4), 44.8 (C-11), 37.0 (C-8), 32.3 (C-9), 22.4 (C-10). FT-IR (neat, cm^{-1}): 3285, 3127, 2988, 2952, 2897, 1638, 1545, 1430, 1405, 1372, 1313, 1271, 1139, 1112, 1053, 1037, 1002, 925, 834, 756, 735, 718, 606, 525. HRMS (ESI) *m/z:* $[M + H]^+$ calcd for $C_9H_{13}ClNO$, 186.0680; found, 189.0679.

tert-Butyl 2-oxo-4-(tosyloxy)-2,5-dihydro-1*H*-pyrrole-1-carboxylate (S7)

Under N_2 atmosphere, *N*-Boc-protected tetramic acid (**18**, 2.50 g, 12.6 mmol, 1.00 eq.) was dissolved in dry CH_2Cl_2 (125 mL) and *p*TsCl (2.51 g, 13.2 mmol, 1.05 eq.) was added at 0 °C to give a yellow slurry. DIPEA (4.29 mL, 25.1 mmol, 2.00 eq.) was added dropwise at 0 °C to this solution over 30 min (143 µL min^{-1} with a syringe pump) to yield a clear yellow solution turning dark brown to black over time. The resulting mixture was stirred for 6 h at 21 °C. Then, the reaction mixture was washed with 5% HCl (15 mL), 5% $NaHCO_3$ (25 mL), and brine (25 mL), dried over $MgSO_4$, and filtered. The solvent was removed *in vacuo*, and the residue was chromatographed on silica using PE/EtOAc (80:20) to give the *O*-tosylated Boc-protected tetramic acid (3.75 g, 10.6 mmol, 84%) as a white solid. R_f (PE/EtOAc 80:20): 0.12 (UV and $KMnO_4$). R_f (PE/EtOAc 60:40): 0.41 (UV and $KMnO_4$). Mp 104.8–106.5 °C. ^{1}H NMR (600 MHz, $CDCl_3$, δ): 7.84 (d, *J*=8.0, 2H, 19-H, 23-H), 7.41 (d, *J*=7.9, 2H, 20-H, 22-H), 5.74 (s, 1H, 3-H), 4.21 (s, 2H, 5-H), 2.48 (s, 3H, 24-H), 1.51 (s, 9H, 12-H, 13-H, 14-H). ^{13}C NMR (151 MHz, $CDCl_3$, δ): 167.0 (C-2), 162.9 (C-4), 148.9 (C-8), 147.3 (C-21), 131.1 (C-18), 130.5 (C-20, C-22), 128.7 (C-19, C-23), 107.8 (C-3), 83.6 (C-11), 49.6 (C-5), 28.2 (C-12, C-13, C-14), 22.0 (C-24). FT-IR (neat, cm^{-1}): 3136, 3007, 2995, 2967, 2956, 2932, 2883, 1737, 1709, 1625, 1595, 1491, 1480, 1461, 1448, 1396, 1372, 1361, 1346, 1304, 1259, 1206, 1190, 1179, 1159, 1121, 1082, 1038, 1015, 1008, 993, 935, 858, 842, 810, 800, 783, 745, 725, 701, 676, 661, 599, 561, 549, 502, 493, 480, 468. HRMS (ESI) *m/z:* $[M + H]^+$ calcd for $C_{16}H_{20}NO_6S$, 354.1006; found, 189.0679.

(*Z*)-4'-Methoxy-5'-((5-methyl-4-propyl-1*H*-pyrrol-2-yl)methylene)-1*H*,5'*H*-[2,2'-bipyrrol]-1'-ium chloride (S17)

Propylprodigiosin **S17** was synthesized in a palladium-catalyzed Suzuki-Miyaura cross-coupling reaction between unsubstituted Boc-pyrrole boronic acid **S6** and triflate **10** to evaluate different Pd ligands and precatalysts [$Pd(PPh_3)_4$, dppf, SPhos, XPhos, and RuPhos] to catalyze the reaction at mild temperatures below 60 °C.

A 25 mL Schlenk flask was charged with triflate **10** (50.0 mg, 132 µmol, 1.00 eq.), *N*-Boc-pyrrole-2-boronic acid (**S6**, 55.8 mg, 264 µmol, 2.00 eq.), ligand (see Table S3, 7.93 µmol, 0.06 eq.), and $Pd(OAc)_2$ (1.48 mg, 6.61 µmol, 0.05 eq.), and the reaction vessel was three times vacuum/nitrogen backfilled. For the $Pd(PPh_3)_4$ precatalyst instead, no additional ligand or $Pd(OAc)_2$ was added and the precatalyst was used at a final concentration of 5 mol% (7.64 mg, 6.61 µmol, 0.05 eq.). The solids were dissolved in degassed *n*BuOH (728 µL) and supplemented by the addition of a solution of K_3PO_4 (56.1 mg, 264 µmol, 2.00 eq.) in degassed water (182 µL). The reactions were stirred under N_2 atmosphere at 40 °C or 60 °C for 18 h (cf. Table S3). Finally, the reaction solution was diluted with CH_2Cl_2 (5 mL) and a saturated aqueous solution of $NaHCO_3$ (5 mL) was added. The aqueous phase was repeatedly extracted with CH_2Cl_2 (3 x 5 mL). After drying the merged organic phases over $MgSO_4$, an orange extract was obtained. Purification by column chromatography on silica with CH_2Cl_2 + 7 N NH_3 in MeOH (gradient, 0–1%), followed by a second column on silica using CH_2Cl_2 with 7 N NH_3 in MeOH (isocratic, 1%), and acidification with 1 M HCl In Et_2O provided the product as a red solid in yields between 18-84% (for isolated yields see Table S3). ^{1}H NMR (300 MHz, $CDCl_3$, δ): 12.66 (brs, 2H, 7-H, 13-H), 12.53 (brs, 1H, 1-H), 7.20 (td, *J*=2.6, 1.2, 1H, 2-H), 6.92 (s, 1H, 11-H), 6.89 (td, *J*=1.8, 1H, 4-H), 6.66 (d, *J*=2.0, 1H, 16-H), 6.33 (dt, *J*=4.3, 1.8, 1H, 3-H), 6.06 (d, *J*=1.6, 1H, 10-H), 3.98 (s, 3H, 18-H), 2.53 (s, 3H, 19-H), 2.36 (t, *J*=7.5, 2H, 20-H), 1.55 (h, *J*=7.4, 2H, 21-H), 0.93 (t, *J*=7.3, 3H, 22-H). ^{13}C NMR (76 MHz, $CDCl_3$, δ): 165.9 (C-9), 147.8 (C-6), 147.0 (C-14), 128.5 (C-15), 128.3 (C-16), 127.0 (C-2), 125.2 (C-12), 122.3 (C-5), 120.8 (C-8), 117.2 (C-4), 116.1 (C-11), 111.8 (C-3), 93.0 (C-10), 58.9 (C-18), 27.5 (C-20), 23.4 (C-21), 13.9 (C-22), 12.5 (C-19). FT-IR (neat, cm^{-1}): 3153, 3097, 3069, 3010, 2958, 2926, 2870, 1629, 1601, 1574, 1542, 1510, 1450, 1352, 1250, 1130, 1066, 1042, 987, 957, 880, 836, 808, 780, 744, 720, 698, 649, 621, 594. HRMS (ESI) *m/z*: $[M - Cl]^+$ calcd for $C_{18}H_{22}N_3O$, 296.1757; found, 296.1757.

N-Methoxy-*N*-methylhex-5-enamide (S18)

A 1 L Schlenk flask under N_2 atmosphere was charged with 5-hexenoic acid (**S10**, 9.96 g, 87.3 mmol, 1.00 eq.) and the acid was dissolved in dry CH_2Cl_2 (300 mL). *N*,*O*-Dimethylhydroxylamine hydrochloride (10.2 g, 105 mmol, 1.20 eq.), *N*-(3-dimethylaminopropyl)-*N*-ethylcarbodiimide hydrochloride (20.1 g, 105 mmol, 1.20 eq.), and DMAP (16.0 g, 131 mmol, 1.50 eq.) were added sequentially. The reaction was stirred at 21 °C for 23 h, quenched with saturated NH_4Cl, and the phases were separated. The aqueous phase was extracted with CH_2Cl_2 (3 x 150 mL) and the merged organic phases were dried over $MgSO_4$. The solution was filtered and the desired product (13.6 g, 86.3 mmol, 99%) was obtained as a volatile colorless liquid after solvent evaporation. The product was used without further purification. R_f (PE/EtOAc 40:60): 0.54 ($KMnO_4$). ^{1}H NMR (600 MHz, $CDCl_3$, δ): 5.78 (dtd, *J*=16.7, 9.6, 1H, 5-H), 5.02 (dt, *J*=17.1, 1.8, 1H, 6a-H), 4.96 (d, *J*=10.0, 1H, 6b-H), 3.66 (s, 3H, 11-H), 3.16 (s, 3H, 9-H), 2.42 (t, *J*=7.7, 2H, 2-H), 2.10 (q, *J*=7.3, 2H, 4-H), 1.73 (p, *J*=7.3, 7.8, 2H, 3-H). ^{13}C NMR (151 MHz, $CDCl_3$, δ): 174.6 (C-1), 138.3 (C-5), 115.2 (C-6), 61.3 (C-11), 33.4 (C-4), 32.3 (C-9), 31.2 (C-2), 23.8 (C-3). FT-IR (neat, cm^{-1}): 3077, 2995, 2973, 2938, 2911, 2870, 2821, 1663, 1460, 1441, 1415, 1384, 1349, 1318, 1177, 1151, 1117, 1092, 996, 912, 859, 799, 782, 762, 745, 733, 722, 635, 610, 564, 496. HRMS (ESI) *m/z:* $[M + H]^+$ calcd for $C_8H_{16}NO_2$, 158.1176; found, 158.1176.

Hept-6-en-2-one (S19)

Under N_2 atmosphere, the Weinreb amide (**S18**, 23.1 g, 147 mmol, 1.00 eq.) was dissolved in dry THF (500 mL) and cooled to 0 °C. Methylmagnesium chloride (98.1 mL, 294 mmol, 2.00 eq., 3.0 M in THF) was added dropwise over 30 min and the reaction stirred for a further 3 h at 0 °C. At 0 °C, saturated NH_4Cl was carefully added for quenching and the reaction was further stirred for 15 min. The phases were separated and the aqueous phase was diluted with water until the solid had nearly dissolved. Then the aqueous phase was extracted with *n*-pentane (3 x 150 mL). Merged organic phases were dried over $MgSO_4$, filtered, and the solvent was carefully removed *in vacuo* to yield the product (15.2 g, 136 mmol, 92%) as a volatile, light-yellow liquid. The product was used without further purification. R_f (PE/EtOAc 80:20): 0.57 ($KMnO_4$). ^{1}H NMR (600 MHz, $CDCl_3$, δ): 5.76 (ddt, *J*=17.0, 10.2, 6.7, 1H, 6-H), 5.01 (dq, *J*=17.0, 1.7, 1H, 7a-H), 4.97 (ddt, *J*=10.2, 2.5, 1.2, 1H, 7b-H), 2.43 (t, *J*=7.4, 2H, 3-H), 2.13 (s, 3H), 2.05 (qd, *J*=7.0, 1.4, 2H, 5-H), 1.67 (p, *J*=7.4, 2H, 4-H). ^{13}C NMR (151 MHz, $CDCl_3$, δ): 208.9 (C-2), 138.1 (C-6), 115.4 (C-7), 43.0 (C-3), 33.2 (C-5), 30.1 (C-1), 23.0 (C-4). FT-IR (neat, cm^{-1}): 3078, 2998, 2976, 2935, 2867, 1715, 1641, 1439, 1414, 1361, 1305, 1224, 1164, 1112, 1067, 1048, 1037, 995, 913, 734, 640, 585, 556, 532. HRMS (ESI) *m/z:* $[M + H]^+$ calcd for $C_7H_{13}O$, 113.0961; found, 113.0959.

(*E*/*Z*)-Hept-6-en-2-one oxime (S20)

A 500 mL round-bottom flask was charged with sodium acetate (17.6 g, 214 mmol, 2.00 eq.) and hydroxylamine hydrochloride (11.2 g, 161 mmol, 1.50 eq.), and the reactants were dissolved in 357 mL EtOH/water (4:1). 6-Hepten-2-one (**S19**, 12.0 g, 107 mmol, 1.00 eq., 0.3 M) was added to the suspension and the reaction mixture was heated to reflux for 4 h. The reaction was cooled to ambient temperature and excess EtOH was removed under reduced pressure. Water (100 mL) was added to the crude mixture, and the aqueous phase was subsequently extracted with EtOAc (3 × 100 mL). Combined organic phases were then washed with saturated $NaHCO_3$

(2 x 100 mL) and water (2 x 100 mL), dried over $MgSO_4$, filtered, and concentrated *in vacuo*. The oxime product (12.8 g, 100 mmol, 94%) was obtained as a yellow liquid with a fruity smell and used without further purification. (*E*/*Z*)-ratio: 72:28 (NMR). *E*-Isomer: ^1H NMR (600 MHz, $CDCl_3$, δ): 8.62 (brs, 1H, 9-OH), 5.81 (tdt, *J*=17.2, 10.4, 6.7, 1H, 6-H), 5.02 (dq, *J*=17.1, 1.7, 1H, 7a-H), 4.97 (ddd, *J*=10.2, 1.2, 1H, 7b-H), 2.20 (t, *J*=7.6, 2H, 3-H), 2.07 (q, *J*=7.2, 2H, 5-H), 1.88 (s, 3H, 1-H), 1.610 (p, *J*=7.6, 2H, 4-H). ^{13}C NMR (151 MHz, $CDCl_3$, δ): 158.6 (C-2), 138.2 (C-6), 115.2 (C-7), 35.4 (C-3), 33.3 (C-5), 25.6 (C-4), 13.5 (C-1). *Z*-Isomer: ^1H NMR (600 MHz, $CDCl_3$, δ): 8.51 (brs, 1H, 9'-OH), 5.81 (tdt, *J*=17.2, 10.4, 6.7, 1H, 6'-H), 5.04 (dq, *J*=17.1, 1.8, 1H, 7'a-H), 5.00–4.95 (m, *J*=10.2, 1.2, 1H, 7'b-H), 2.40–2.35 (m, *J*=8.1, 2H, 3'-H), 2.10 (q, *J*=7.7, 2H, 5'-H), 1.87 (s, 3H, 1'-H), 1.608 (q, *J*=7.7, 2H, 4'-H). ^{13}C NMR (151 MHz, $CDCl_3$, δ): 159.0 (C-2'), 138.3 (C-6'), 115.1 (C-7'), 33.8 (C-5'), 28.2 (C-3'), 24.9 (C-4'), 20.0 (C-1'). FT-IR (neat, cm^{-1}): 3226, 3119, 3078, 2977, 2927, 2862, 1664, 1641, 1456, 1440, 1417, 1368, 1303, 1278, 1256, 1233, 1207, 1188, 1166, 1113, 1024, 996, 954, 912, 815, 760, 741, 632, 577, 557, 535, 523. HRMS (ESI) *m/z:* $[M + H]^+$ calcd for $C_7H_{14}NO$, 128.1070; found, 128.1071.

3-(But-3-en-1-yl)-2-methyl-1*H*-pyrrole (S9)

(*E*/*Z*)-Hept-6-en-2-one oxime (**S20**, 14.3 g, 112 mmol, 1.00 eq.), pestled KOH (31.5 g, 562 mmol, 5.00 eq.), and water (1.54 mL, 13.7 µL $mmol^{-1}$ oxime) were sequentially added to a 500 mL three-necked flask under N_2 atmosphere. Degassed and dried DMSO (216 mL, 1.92 mL $mmol^{-1}$ oxime) was added. The reaction was heated at 100 °C and a solution of 1,2-dichloroethane (31.1 mL, 394 mmol, 3.50 eq.) in degassed DMSO (23.6 mL, 0.21 mL $mmol^{-1}$ oxime) was added with a syringe pump over 2 h. A second batch of KOH (31.5 g, 562 mmol, 5.00 eq.) was added under ice cooling after the first hour of 1,2-dichloroethane addition (dichloroethane-feeding is paused during KOH addition) before the dichloroethane addition was continued for a further 1 h. Once the addition of dichloroethane was completed, the reaction was heated at 100 °C for 2 h. The reaction was cooled to 0 °C and ice water (400 mL) was added for quenching until potassium salts were completely dissolved. Extraction was performed with Et_2O (3 x 200 mL). Combined organic phases were dried over $MgSO_4$, filtered over degreased cotton wool, and the solvent was removed *in vacuo*. Kugelrohr distillation at 120–140 °C at 3 x 10^{-2} mbar provided the impure product as an orange liquid. Further chromatographic purification on silica with *n*-pentane/Et_2O (95:5) + 1% TEA provided the monopyrrole (9.00 g, 66.6 mmol, 59%) as a yellow-orange liquid. R_f (*n*-pentane/Et_2O 95:5): 0.24 ($KMnO_4$). R_f (PE/EtOAc 80:20): 0.58 ($KMnO_4$). ^1H NMR (600 MHz, $CDCl_3$, δ): 7.73 (brs, 1H, 1-H), 6.61 (t, *J*=2.7, 1H, 5-H), 6.05 (t, *J*=2.7, 1H, 4-H), 5.92 (ddt, *J*=17.1, 10.1, 6.5, 1H, 9-H), 5.07 (dq, *J*=17.2, 1.7, 1H, 10a-H), 5.02 – 4.92 (dq, *J*=10.1, 1.3, 1H, 10b-H), 2.51 (dt, *J*=8.6, 7.4, 2H, 7-H), 2.31 (m, *J*=7.2, 2H, 8-H), 2.20 (s, 3H, 6-H). ^{13}C NMR (151 MHz, $CDCl_3$, δ): 139.2 (C-9), 123.5 (C-2), 119.0 (C-3), 115.1 (C-5), 114.4 (C-10), 108.9 (C-4), 35.5 (C-8), 25.7 (C-7), 11.2 (C-6). FT-IR (neat, cm^{-1}): 3378, 3075, 2975, 2917, 2851, 1639, 1585, 1464, 1439, 1415, 1378, 1338, 1274, 1247, 1201, 1192, 1107, 1090, 1064, 995, 955, 909, 834, 786, 714, 669, 651, 601, 554, 547, 538, 531, 522, 510. HRMS (ESI) *m/z:* $[M + H]^+$ calcd for $C_9H_{14}N$, 136.1121; found, 136.1122.

4-(2-Methyl-1*H*-pyrrol-3-yl)butan-1-ol (32)

From the hydroboration-oxidation route: To a 500 mL three-necked flask under N_2 atmosphere was added alkene pyrrole (**S9**, 2.93 g, 21.7 mmol, 1.00 eq.). The pyrrole was dissolved in dry and degassed THF (179 mL, 8.24 mL $mmol^{-1}$ pyrrole, 0.25 M pyrrole) and stirred for 15 min at 0 °C. A solution of 9-BBN (69.3 mL, 34.7 mmol, 1.60 eq., 0.5 M in THF) was added over 15 min with a syringe pump while keeping the reaction at 0 °C (pale yellow). Stirring was continued at 0 °C for 1 h, followed by reflux at 75 °C for 3 h (pale orange). At 0 °C, 36.2 mL 3 M NaOH (*aq.*) (turning yellow) and then 34.4 mL 30% H_2O_2 (first pinkish, then yellow, then turbid colorless) were added dropwise. After 1 h at 0 °C (boric acid precipitated as a white solid), the reaction was allowed to warm to 20 °C and stirred for a further 17 h. The reaction was diluted with ice water and repeatedly extracted with CH_2Cl_2 (3 x 75 mL). Merged organic phases (pale-yellow) were dried over $MgSO_4$, filtered, and the solvent was removed *in vacuo*. The orange crude product was chromatographed on silica with PE/EtOAc (70:30) + 1% TEA to yield the desired product (2.89 g, 18.9 mmol, 87%) as pale-yellow viscous oil. R_f (PE/EtOAc 20:80): 0.62 ($KMnO_4$). ^1H NMR (600 MHz, $CDCl_3$, δ): 7.83 (brs, 1H, 1-H), 6.59 (t, *J*=2.7, 1H, 5-H), 6.02 (t, *J*=2.8, 1H, 4-H), 3.66 (t, *J*=6.1, 2H, 10-H), 2.43 (t, *J*=6.9, 2H, 7-H), 2.19 (s, 3H, 6-H), 1.67–1.56 (m, *J*=6.9, 4H, 8-H, 9-H). ^{13}C NMR (151 MHz, $CDCl_3$, δ): 123.4 (C-2), 119.2 (C-3), 115.1 (C-5), 108.8 (C-4), 63.1 (C-10), 32.7 (C-9), 27.4 (C-8), 25.7 (C-7), 11.1 (C-6). FT-IR (neat, cm^{-1}): 3367, 3094, 2929, 2857, 2736, 1668, 1585, 1544, 1445, 1378, 1276, 1261, 1246, 1206, 1176, 1136, 1102, 1058, 1028, 979, 957, 932, 900, 835, 715, 667, 596, 562. HRMS (ESI) *m/z:* $[M + H]^+$ calcd for $C_9H_{16}NO$, 154.1226; found, 154.1228.

Analytical Procedures

Coupled Gas Chromatography-Mass Spectrometry (GC-MS)

Coupled GC-MS measurements were performed to evaluate the progress of specific reactions and the relative portion of volatile compounds in complex mixtures. For this purpose, a Trace 1310 gas chromatograph (Thermo Scientific) with a coupled ISQTM QD Single Quadrupole mass spectrometer was used. All samples were dissolved in MTBE, and 1 µL per sample was injected into the split injector (heated to 250 °C). At a carrier gas flow rate of 1 mL/min, samples were separated on an Optima 5MS column (30 m x 0.25 mm, 0.25 µm particle size, Macherey-Nagel) with helium carrier gas according to the following temperature profile: hold 60 °C for 1 min; heating at 15 °C/min increment up to 185 °C; heating at 120 °C/min increment up to 280 °C; hold 280 °C for 5 min. The transfer line to the MS instrument was heated at 280 °C, and the samples were ionized by electron impact ionization at 230 °C and 70 eV. Spectra were recorded in an *m/z* range from 50−1000 with a scan time of 0.2 s.

Physical Procedures

Experimental Determination of Molar Extinction Coefficients

The prodigiosin maleimides, namely prodigiosin A-ring maleimide **46**, prodigiosin B-ring maleimide **47**, and the prodigiosin C-ring maleimide **48**, were weighed into a 1.5 mL reagent tube and dissolved in acidified EtOH (EtOH p.a. + 4% 1 M HCl) to a concentration of 10 mM. For each compound, three individual samples were prepared.

<u>For measurements in acidified EtOH:</u> Via consecutive 1:10 dilutions in acidified EtOH were 1 mM, 100 µM, and 10 µM solutions prepared. Dilutions (1 µM, 3 µM, and 5 µM) were then prepared for every individual sample from the 10 µM dilution. Absorption values were determined at 535 nm and at the respective wavelength of maximum absorption.

<u>For measurements in aqueous buffer with EtOH as co-solvent:</u> The 10 mM stock solution in acidified EtOH was diluted to 1 mM in EtOH (p.a.). The 1 mM dilution is then used for 1:10 dilutions in dialysis buffer + 20% EtOH up to a final concentration of 10 µM. Therefore, 100 µL of the 1 mM dilution in EtOH (p.a.) was sequentially mixed with 100 µL EtOH, and 800 µL dialysis buffer was finally added. 100 µL of this dilution was mixed with 180 µL EtOH, and 720 µL dialysis buffer was added to maintain the final concentration of 20% EtOH. From this 10 µM solution, 3 µM, 5 µM, and 7 µM dilutions were prepared as before. Absorption values were determined at 280 nm and at the respective absorption maximum.

All measurements were performed on a Shimadzu UV-1800 UV Spectrophotometer with a tempered measuring cell at 20 °C. A quartz glass cuvette (Hellma Analytics Quartz Glass SUPRASIL QS, 10 mm light path) was used throughout all experiments. Absorption spectra were collected in a range from 200–800 nm with 1.0 nm increment (mode: fast) from 10 µM prodigiosin solutions. Absorption measurements at static wavelengths were performed at the indicated wavelengths. All values were corrected by subtracting the background signals from acidified EtOH or Buffer + 20% EtOH. Resulting from the Beer-Lambert law ($E = \varepsilon \cdot c \cdot d$), the molar extinction E of every individual sample was plotted against the molar concentration c. Molar extinction coefficients ε were then deduced from the slope of a linear regression curve (calculated with Origin 2021). The light path length d was 10 mm throughout all experiments.

Biochemical and Microbiological Procedures

General Information

Liquid cultures of bacteria were cultivated in lysogeny broth (LB) medium (10 g L^{-1} tryptone, 5 g L^{-1} yeast extract, 10 g L^{-1} NaCl, adjusted to pH 7.2 with NaOH) and terrific broth (TB) medium (Carl Roth, Terrific-Broth-Medium, 4 mL L^{-1} glycerol added). Plating on solid media was performed on LB agar, containing 1.4% agar-agar. Stock solutions of ampicillin (100 mg mL^{-1}) and IPTG (0.5 M) in water were filter-sterilized with 0.2 µm syringe filters. Strains of *E. coli* were transformed with plasmid DNA in accordance with the heat shock protocol from New England Biolabs (NEB) and plated on antibiotic-supplemented LB agar for selection.

Bacterial Strains and Plasmids

Table S7. Bacterial strains used in this work.

Strain	Genotype or relevant characteristic	Reference
E. coli BL21 (DE3)	*E. coli* strain B, F⁻ *ompT gal dcm lon* hsdSB($r_B^-m_B^-$) *λ*(DE3) [*lacI lacUV5-T7p07 ind1 sam7 nin5*]) [*malB*$^+$]$_{K-12}$($λ^S$)	[46]

Table S8. Plasmids used in this work.

Plasmid	Genotype	Encoded Proteins
pTWE10	pET21a(+), *pyoS2*, *imS2-His*$_6$	PyoS2, ImS2-His$_6$
pTWE16	pET21a(+), *pyoS2-Q570C*, *imS2-His*$_6$	PyoS2 Q570C, ImS2-His$_6$

Heterologous Protein Synthesis

Proteins were heterologously produced in *Escherichia coli* BL21 (DE3) by expression from pET21a(+) vectors, harboring a tandem of the coding genes for pyocin S2 (*pyoS2*) and its C-terminally His-tagged immunity protein (*imS2-His*$_6$). Precultures in LB media (100 µg mL^{-1} ampicillin) were inoculated with a single colony of *E. coli* BL21 (DE3) pET21a(+)_*pyoS2 mutant_ImS2-His*$_6$ and incubated overnight at 37 °C (130 RPM). 2 L of TB media (100 µg mL^{-1} ampicillin) in a 5 L Erlenmeyer flask were inoculated to an optical density (OD_{600}) of 0.05 and cultures grown at 37 °C (130 RPM) until an OD_{600} of 0.6–0.8 was reached. Cultures were allowed to chill at room temperature for 30 min prior to induction with 500 µm isopropyl-β-D-thiogalactopyranoside (IPTG). Protein production was then carried out overnight at 25 °C (130 RPM). Expression cultures were harvested by centrifugation (4,500 RPM, 15 min, 4 °C), the supernatant was discarded, the cell pellets were resuspended in an appropriate amount of binding buffer (20 mM Tris-HCl, 500 mM NaCl, 5 mM imidazole, pH 7.5), and stored at −20 °C until needed.

Purification of PyoS2 Derivatives

For purification, the resuspended cells were supplemented with 1 mM PMSF (100 mM in *i*PrOH) and disrupted by sonication (3 x 10 min, 40% amplitude, 5 min rest on ice). Debris was removed by centrifugation (15,000 x g, 2 x 30 min, 4 °C) and the supernatant was separated from the pellet. The cleared lysate was then transferred onto a 20 mL (4 x 5 mL) Qiagen Ni-NTA Superflow column[1], equilibrated in binding buffer, and afterwards thoroughly washed with binding buffer (min. 35–40 CV, column volumes) to remove unbound PyoS2/ImS2 complex. All pyocins were purified as previously described for colicins by disrupting the column-bound PyoS2/ImS2 complex by washing with 3 CV of complex disruption buffer (20 mM Tris-HCl, 500 mM NaCl, 6 M guanidine hydrochloride, pH 7.5), leaving the His-tagged ImS2 bound to the NTA resin.[47] After washing the column with 2 CV of binding buffer, the immunity protein was separately eluted with elution buffer (20 mM Tris-HCl, 500 mM NaCl, 250 mM imidazole, pH 7.5). Eluted proteins from Ni-NTA purification were dialyzed overnight at 4 °C against 5 L of dialysis buffer (20 mM Tris-HCl, 500 mM NaCl, 250 mM imidazole, pH 7.5) with Spectra/Por 12–14 kDa tubing and subsequently purified by size exclusion chromatography on a Cytiva HiLoad 26/600 Superdex 200 prep grade column with dialysis buffer as eluent. Protein solutions were finally reduced in volume by centrifugal concentration (Sartorius Vivaspin 20, 10 kDa MWCO) to the desired concentration and stored at −20 °C until needed.

Maleimide Labeling of PyoS2 Cysteine Mutants

Purified cysteine mutants of PyoS2 were thawed on ice and dithiothreitol (DTT) was added to a final concentration of 10 mM to reduce putative disulfide bridges. Incubation was carried out for 60 min on ice. Excess of reducing agent was removed by desalting into labeling buffer (25 mM Tris-HCl, 150 mM NaCl, pH 7.5) on a Cytiva HiLoad desalting column (5 mL). PyoS2-containing fractions were merged and the protein concentration was determined. The desired prodigiosin maleimide (prodigiosin A-ring maleimide **46**, prodigiosin B-ring maleimide **47**, or prodigiosin C-ring maleimide **48**, 10 mM in DMSO) was added to a 3-fold excess and incubated at room temperature

[1] Ni-NTA columns were stripped with ETDA (20 mM Na_3PO_4, 500 mM NaCl, 50 mM Na_2-EDTA, pH 7.4) and cleaned with 1 M NaOH before each run, to avoid cross contamination of DNase active and inactive PyoS2 variants.

under gentle agitation in the dark for 1.5 h. DTT was added to a final concentration of 5 mM for quenching and the labeled proteins were separated from unreacted prodigiosin maleimide by desalting into dialysis buffer (25 mM Tris-HCl, 150 mM NaCl, pH 8.0). After reduction of the volume in centrifugal concentrators (Sartorius Vivaspin 500, 10 kDa MWCO) to the desired protein concentration, labeled proteins were stored at −20 °C until needed.

Protein Quantification

Purified and unlabeled proteins were quantified by measuring the absorption at 280 nm according to the Beer-Lambert Law. Molar extinction coefficients (Table S9) and molecular weights were predicted and calculated with the ExPASy ProtParam tool. Prodigiosin-labeled proteins were quantified using the Pierce 660 nm assay, calibrated and validated with bovine serum albumin (determined by weight) and wild-type pyocin S2 (determined by UV quantification). Briefly, proteins of unknown concentration were diluted 1:10 in dialysis buffer (25 mM Tris-HCl, 150 mM NaCl, pH 8.0) and 10 µL of this dilution was placed in a 96-well plate (four replicates per protein). For BSA, 10 µL of distinct concentrations in a range from 50−2000 µg mL^{-1} (diluted in dialysis buffer) were placed in a 96-well plate. 150 µL of the Pierce 660 nm assay reagent (Thermo Scientific, Product No. 22660) was added with a multi-channel pipette and briefly mixed by pipetting. Plates were incubated for 5 min at ambient temperature, followed by quick shaking and measurement of the absorption at 660 nm. Data sets were fitted by linear regression and protein concentrations were determined on the basis of this fit.

Table S9. Purified proteins and their physical parameters for UV_{280} quantification.

Protein	Amino Acids[a]	Molecular Weight [Da]	Extinction Coefficient [M^{-1} cm^{-1}][b]
Pyocin S2 (wild-type)	688	73722.65	65780
Pyocin S2 Q570C	688	73697.65	65780

[a] The N-terminal translation initiator methionine is post-transcriptionally processed and removed, as proven by mass spectrometry. Therefore, molecular weights and molar extinction coefficients were calculated without the initial methionine for all proteins; [b] Calculated with the ExPASy.ProtParam tool.

Sodium Dodecyl Sulfate Polyacrylamide Gel Electrophoresis (SDS-PAGE)

Purified proteins were diluted in dialysis buffer (25 mM Tris-HCl, 150 mM NaCl, pH 8.0) to a concentration of 0.33 µg $µL^{-1}$ and mixed with 4x SDS sample buffer (200 mM Tris-HCl, pH 6.8, 8% w/v SDS, 0.4% w/v bromophenol blue, 40% w/v glycerol, 400 mM β-mercaptoethanol) to give a final concentration of 0.25 µg $µL^{-1}$. Samples were heated at 98 °C for 10 min and used without centrifugation. 5 µL marker (ThermoScientific PageRuler Unstained Protein Ladder, Ref 26614) and 8 µL protein sample (equals 2 µg protein per well) were applied to a commercial Invitrogen NuPAGE 4−12% Bis-Tris gel (1.0 mm x 15 well, Ref NP0323BOX). Gels were run at 180 V for 46 min in 1x NuPAGE MOPS SDS running buffer (50 mM MOPS, 50 mM Tris Base, 0.1% SDS, 1 mM EDTA, pH 7.7, Ref NP0001). After three-times washing with water and incubation with colloidal Coomassie overnight, stained gels were incubated in water with a cellulose tissue to destain the background. Gels were documented by photography.

Electrospray Ionization Mass Spectrometry (ESI-MS)

Purified and labeled proteins were analyzed and verified by LC-coupled ESI-MS measurements at the Central Institute of Engineering, Electronics and Analytics (ZEA-3) at the Jülich Research Center. Concentrated samples were diluted 1:10 with water and separated on an Accucore 150-C4 HPLC column (100 mm x 4.6 mm, 2.6 µm particle size), using a 5−95% gradient of acetonitrile. The eluents water and acetonitrile were supplemented with 0.025% heptafluorobutyric acid (HFBA) for ion-pairing and 1% formic acid (FA). Eluent A (water + 0.025% HFBA + 1% FA), eluent B (MeCN + 0.025% HFBA + 1% FA).

References

[1] B. A. Trofimov, A. I. Mikhaleva, A. N. Vasil'ev, S. E. Korostova, S. G. Shevchenko, "Pyrroles from Ketoximes and Acetylene. 29. Synthesis of Alkylpyrroles from Dialkylketoximes and Dichloroethane by Reaction with KOH-DMSO" *Chem. Heterocycl. Compd.* **1985**, *21*, 46-49; 10.1007/BF00505898.

[2] T. M. Weber, A. Leyens, L. Berning, B. Stork, J. Pietruszka, "New prodigiosin derivatives – chemoenzymatic synthesis and physiological evaluation against cisplatin-resistant cancer cells" *Catal. Sci. Technol.* **2023**, *13*(21), 6165-6184; 10.1039/D3CY00913K.

[3] X. Mo, T. D. R. Morgan, H. T. Ang, D. G. Hall, "Scope and Mechanism of a True Organocatalytic Beckmann Rearrangement with a Boronic Acid/Perfluoropinacol System under Ambient Conditions" *J. Am. Chem. Soc.* **2018**, *140*(15), 5264-5271; 10.1021/jacs.8b01618.

[4] A. Fürstner, J. Grabowski, C. W. Lehmann, "Total Synthesis and Structural Refinement of the Cyclic Tripyrrole Pigment Nonylprodigiosin" *J. Org. Chem.* **1999**, *64*(22), 8275-8280; 10.1021/jo991021i.

[5] R. Greenhouse, C. Ramirez, J. M. Muchowski, "Synthesis of Alkylpyrroles by the Sodium Borohydride Reduction of Acylpyrroles" *J. Org. Chem.* **1985**, *50*(16), 2961-2965; 10.1021/jo00216a030.

[6] T. Ono, D. Koga, Y. Hisaeda, "Facile Synthesis of 9,10,19,20-Tetraalkylporphycenes" *Chem. Lett.* **2017**, *46*(2), 260-262; 10.1246/cl.161019.

[7] E. F. Knights, H. C. Brown, "9-Borabicyclo[3.3.1]nonane as a Convenient Selective Hydroborating Agent" *J. Am. Chem. Soc.* **1968**, *90*(19), 5281-5283; 10.1021/ja01021a047.

[8] L. Aldrich, PhD Thesis, "Progress Toward the Total Synthesis of Marineosins A & B; Total Synthesis of Tambjamine K and Unnatural Analogs with Improved Anticancer Activity, and Discovery of Selective M_1 Antagonists", Vanderbilt University Tennessee (USA), **2012**.

[9] M. Fieser, L. F. Fieser, T.-L. Ho, R. Danheiser, W. Roush, "Lithium aluminium hydride" in *Fieser and Fieser's Reagents for Organic Synthesis*, (Eds.: M. Fieser, L. F. Fieser, T.-L. Ho), John Wiley & Sons, Inc., Hoboken, NJ, USA, **2017**; 10.1002/9780471264194.fos06257.pub5.

[10] S. Martina, V. Enkelmann, G. Wegner, A.-D. Schlüter, "*N*-Protected Pyrrole Derivatives Substituted for Metal-Catalyzed Cross-Coupling Reactions" *Synthesis* **1991**, *1991*(8), 613-615; 10.1055/s-1991-26526.

[11] M. D. Clift, R. J. Thomson, "Development of a Merged Conjugate Addition/Oxidative Coupling Sequence. Application to the Enantioselective Total Synthesis of Metacycloprodigiosin and Prodigiosin R1" *J. Am. Chem. Soc.* **2009**, *131*(40), 14579-14583; 10.1021/ja906122g.

[12] A. Fürstner, K. Radkowski, H. Peters, "Chasing a Phantom by Total Synthesis: The Butylcycloheptylprodigiosin Case" *Angew. Chem. Int. Ed.* **2005**, *44*(18), 2777-2781; 10.1002/anie.200462215.

[13] A. Fürstner, K. Radkowski, H. Peters, "Chasing a Phantom by Total Synthesis: The Butylcycloheptylprodigiosin Case" *Angew. Chem.* **2005**, *117*(18), 2837-2841; 10.1002/ange.200462215.

[14] S. Mo, P. K. Sydor, C. Corre, M. M. Alhamadsheh, A. E. Stanley, S. W. Haynes, L. Song, K. A. Reynolds, G. L. Challis, "Elucidation of the *Streptomyces coelicolor* Pathway to 2-Undecylpyrrole, a Key Intermediate in Undecylprodiginine and Streptorubin B Biosynthesis" *Chem. Biol.* **2008**, *15*(2), 137–148; 10.1016/j.chembiol.2007.11.015.

[15] P. Kancharla, J. X. Kelly, K. A. Reynolds, "Synthesis and Structure–Activity Relationships of Tambjamines and B-Ring Functionalized Prodiginines as Potent Antimalarials" *J. Med. Chem.* **2015**, *58*(18), 7286-7309; 10.1021/acs.jmedchem.5b00560.

[16] N. Miyaura, A. Suzuki, "Stereoselective Synthesis of Arylated (*E*)-Alkenes by the Reaction of Alk-1-enylboranes with Aryl Halides in the Presence of Palladium Catalyst" *J. Chem. Soc., Chem. Commun.* **1979**, *19*, 866-867; 10.1039/C39790000866.

[17] N. Miyaura, K. Yamada, A. Suzuki, "A New Stereospecific Cross-Coupling by the Palladium-Catalyzed Reaction of 1-Alkenylboranes with 1-Alkenyl or 1-Alkynyl Halides" *Tetrahedron Lett.* **1979**, *20*(36), 3437-3440; 10.1016/S0040-4039(01)95429-2.

[18] N. Miyaura, T. Yanagi, A. Suzuki, "The Palladium-Catalyzed Cross-Coupling Reaction of Phenylboronic Acid with Haloarenes in the Presence of Bases" *Synth. Commun.* **1981**, *11*(7), 513-519; 10.1080/00397918108063618.

[19] C. N. Johnson, G. Stemp, N. Anand, S. C. Stephen, T. Gallagher, "Palladium (0)-Catalysed Arylations using Pyrrole and Indole 2-Boronic Acids" *Synlett* **1998**, *1998*(9), 1025-1027; 10.1055/s-1998-1834.

[20] M. S. Melvin, J. T. Tomlinson, G. Park, C. S. Day, G. R. Saluta, G. L. Kucera, R. A. Manderville, "Influence of the A-Ring on the Proton Affinity and Anticancer Properties of the Prodigiosins" *Chem. Res. Toxicol.* **2002**, *15*(5), 734-741; 10.1021/tx025507x.

[21] R. D'Alessio, A. Bargiotti, O. Carlini, F. Colotta, M. Ferrari, P. Gnocchi, A. Isetta, N. Mongelli, P. Motta, A. Rossi, M. Rossi, M. Tibolla, E. Vanotti, "Synthesis and Immunosuppressive Activity of Novel Prodigiosin Derivatives" *J. Med. Chem.* **2000**, *43*(13), 2557-2565; 10.1021/jm001003p.

[22] E. Marchal, C. Figliola, A. Thompson, "Prodigiosenes conjugated to tamoxifen and estradiol" *Org. Biomol. Chem.* **2017**, *15*(25), 5410-5427; 10.1039/c7ob00943g.

[23] P. Kancharla, M. Smilkstein, J. X. Kelly, Shweta, S. M. Salem, M. Alhamadsheh, S. W. Haynes, G. L. Challis, K. A. Reynolds, "Antimalarial Activity of Natural and Synthetic Prodiginines" *J. Med. Chem.* **2011**, *54*(15), 5296-5306; 10.1021/jm200543y.

[24] X.-C. Cai, B. B. Snider, "Synthesis of the Spiroiminal Moiety and Approaches to the Synthesis of Marineosins A and B" *J. Org. Chem.* **2013**, *78*(23), 12161-12175; 10.1021/jo402178r.

[25] N. Miyaura, A. Suzuki, "Palladium-Catalyzed Cross-Coupling Reactions of Organoboron Compounds" *Chem. Rev.* **1995**, *95*(7), 2457-2483; 10.1021/cr00039a007.

[26] C. Amatore, M. Azzabi, A. Jutand, "Role and Effects of Halide Ions on the Rates and Mechanisms of Oxidative Addition of Iodobenzene to Low-Ligated Zerovalent Palladium Complexes $Pd^0(PPh_3)_2$" *J. Am. Chem. Soc.* **1991**, *113*(22), 8375-8384; 10.1021/ja00022a026.

[27] P. Fitton, E. A. Rick, "The Addition of Aryl Halides to Tetrakis(triphenylphosphine)palladium(0)" *J. Organomet. Chem.* **1971**, *28*(2), 287-291; 10.1016/S0022-328X(00)84578-7.

[28] D. M. Knapp, E. P. Gillis, M. D. Burke, "A General Solution for Unstable Boronic Acids: Slow-Release Cross-Coupling from Air-Stable MIDA Boronates" *J. Am. Chem. Soc.* **2009**, *131*(20), 6961-6963; 10.1021/ja901416p.

[29] S. Bräse, C. Gil, K. Knepper, V. Zimmermann, "Organic Azides: An Exploding Diversity of a Unique Class of Compounds" *Angew. Chem. Int. Ed.* **2005**, *44*(33), 5188-5240; 10.1002/anie.200400657.

[30] S. Bräse, C. Gil, K. Knepper, V. Zimmermann, "Organische Azide – explodierende Vielfalt bei einer einzigartigen Substanzklasse" *Angew. Chem.* **2005**, *117*(33), 5320-5374; 10.1002/ange.200400657.

[31] E. Tyrrell, P. Brookes, "The Synthesis and Applications of Heterocyclic Boronic Acids" *Synthesis* **2003**, *2003*(4), 0469-0483; 10.1055/s-2003-37721.

[32] S. Ge, J. F. Hartwig, "Highly Reactive, Single-Component Nickel Catalyst Precursor for Suzuki–Miyuara Cross-Coupling of Heteroaryl Boronic Acids with Heteroaryl Halides" *Angew. Chem. Int. Ed.* **2012**, *51*(51), 12837-12841; 10.1002/anie.201207428.

[33] S. Ge, J. F. Hartwig, "Highly Reactive, Single-Component Nickel Catalyst Precursor for Suzuki–Miyuara Cross-Coupling of Heteroaryl Boronic Acids with Heteroaryl Halides" *Angew. Chem.* **2012**, *124*(51), 13009-13013; 10.1002/ange.201207428.

[34] P.-J. Chen, A. M. Kelly, D. J. Blair, M. D. Burke, "Preparation of MIDA Anhydride and Reaction with Boronic Acids" *Org. Synth.* **2022**, *99*, 92-112; 10.15227/orgsyn.099.0092.

[35] M. A. Düfert, K. L. Billingsley, S. L. Buchwald, "Suzuki-Miyaura Cross-Coupling of Unprotected, Nitrogen-Rich Heterocycles: Substrate Scope and Mechanistic Investigation" *J. Am. Chem. Soc.* **2013**, *135*(34), 12877-12885; 10.1021/ja4064469.

[36] K. L. Billingsley, K. W. Anderson, S. L. Buchwald, "A Highly Active Catalyst for Suzuki-Miyaura Cross-Coupling Reactions of Heteroaryl Compounds" *Angew. Chem. Int. Ed.* **2006**, *45*(21), 3484-3488; 10.1002/anie.200600493.

[37] K. L. Billingsley, K. W. Anderson, S. L. Buchwald, "A Highly Active Catalyst for Suzuki-Miyaura Cross-Coupling Reactions of Heteroaryl Compounds" *Angew. Chem.* **2006**, *118*(21), 3564-3568; 10.1002/ange.200600493.

[38] G. R. Fulmer, A. J. M. Miller, N. H. Sherden, H. E. Gottlieb, A. Nudelman, B. M. Stoltz, J. E. Bercaw, K. I. Goldberg, "NMR Chemical Shifts of Trace Impurities: Common Laboratory Solvents, Organics, and Gases in Deuterated Solvents Relevant to the Organometallic Chemist" *Organometallics* **2010**, *29*(9), 2176-2179; 10.1021/om100106e.

[39] S. M. Greening, PhD Thesis, "Synthesis of 2-Aryl Pyrroles, Prodigiosene *F*-BODIPYs and B-Ring Modified Prodigiosenes", Dalhousie University Halifax (Canada), **2019**.

[40] A. S. Klein, A. Domröse, P. Bongen, H. U. C. Brass, T. Classen, A. Loeschcke, T. Drepper, L. Laraia, S. Sievers, K.-E. Jaeger, J. Pietruszka, "New Prodigiosin Derivatives Obtained by Mutasynthesis in *Pseudomonas putida*" *ACS Synth. Biol.* **2017**, *6*(9), 1757-1765; 10.1021/acssynbio.7b00099.

[41] J. Agramunt, R. Ginesi, E. Pedroso, A. Grandas, "Inverse Electron-Demand Diels–Alder Bioconjugation Reactions Using 7-Oxanorbornenes as Dienophiles" *J. Org. Chem.* **2020**, *85*(10), 6593-6604; 10.1021/acs.joc.0c00583.

[42] T. L. Foley, A. Yasgar, C. J. Garcia, A. Jadhav, A. Simeonov, M. D. Burkart, "Preparation of FRET Reporters to Support Chemical Probe Development" *Org. Biomol. Chem.* **2010**, *8*(20), 4601-4606; 10.1039/C0OB00322K.

[43] T. Uehara, T. Rokugawa, M. Kinoshita, S. Nemoto, G. G. Fransisco Lazaro, H. Hanaoka, Y. Arano, "$^{67/68}$Ga-Labeling Agent that Liberates $^{67/68}$Ga-NOTA-Methionine by Lysosomal Proteolysis of Parental Low Molecular Weight Polypeptides to Reduce Renal Radioactivity Levels" *Bioconjugate Chem.* **2014**, *25*(11), 2038-2045; 10.1021/bc5004058.

[44] L. Buschbeck, J. Christoffers, "Orthogonally Protected Diaminoterephthalate Scaffolds: Installation of Two Functional Units at the Chromophore" *J. Org. Chem.* **2018**, *83*(7), 4002-4014; 10.1021/acs.joc.8b00347.

[45] T. M. Weber, J. Pietruszka, "Synthesis of a Water-Soluble Tridentate (Dimethylamino)ethyl Cu(I)/Cu(II)-Ligand" *Synthesis* **2023**, *55*(14), 2128-2133; 10.1055/a-2034-9427.

[46] F. W. Studier, B. A. Moffatt, "Use of bacteriophage T7 RNA polymerase to direct selective high-level expression of cloned genes" *J. Mol. Biol.* **1986**, *189*(1), 113-130; 10.1016/0022-2836(86)90385-2.

[47] R. Wallis, A. Reilly, A. Rowe, G. R. Moore, R. James, C. Kleanthous, "*In vivo* and *in vitro* characterization of overproduced colicin E9 immunity protein" *Eur. J. Biochem.* **1992**, *207*(2), 687-695; 10.1111/j.1432-1033.1992.tb17096.x.

NMR Spectra

(*E*/*Z*)-Hexan-2-one oxime (S1)

^{1}H NMR (600 MHz, $CDCl_3$)

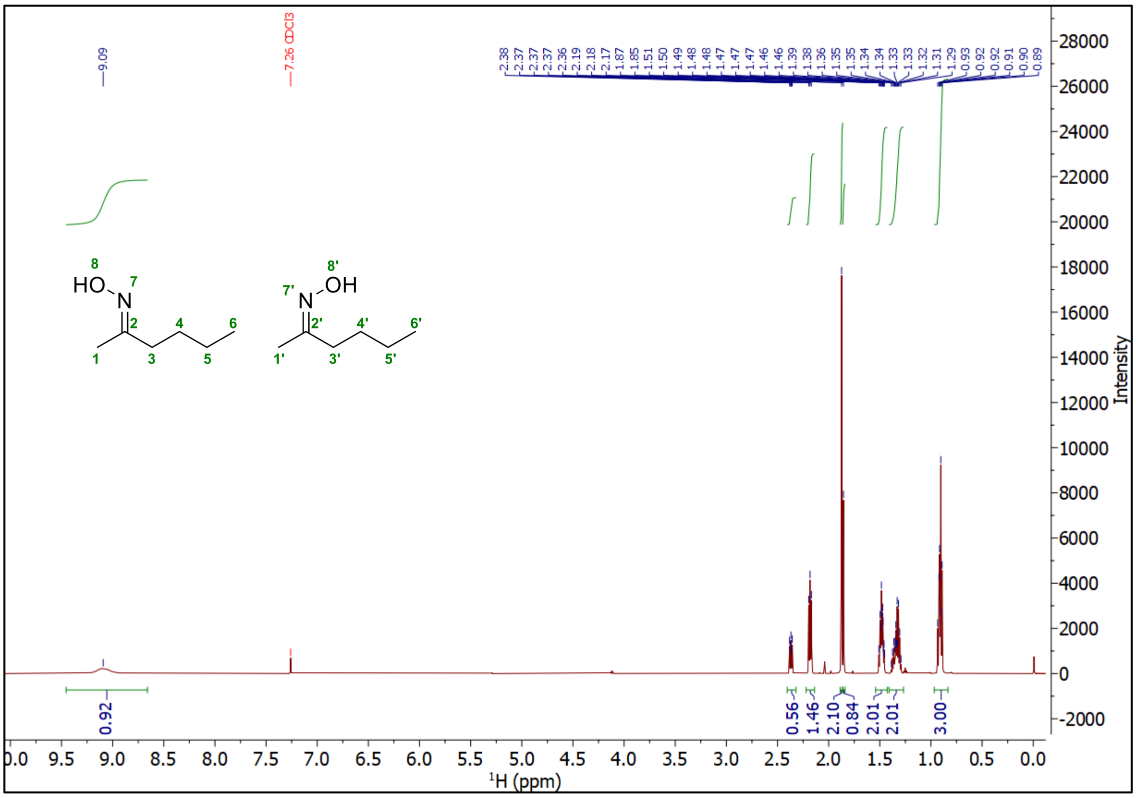

^{13}C NMR (151 MHz, $CDCl_3$)

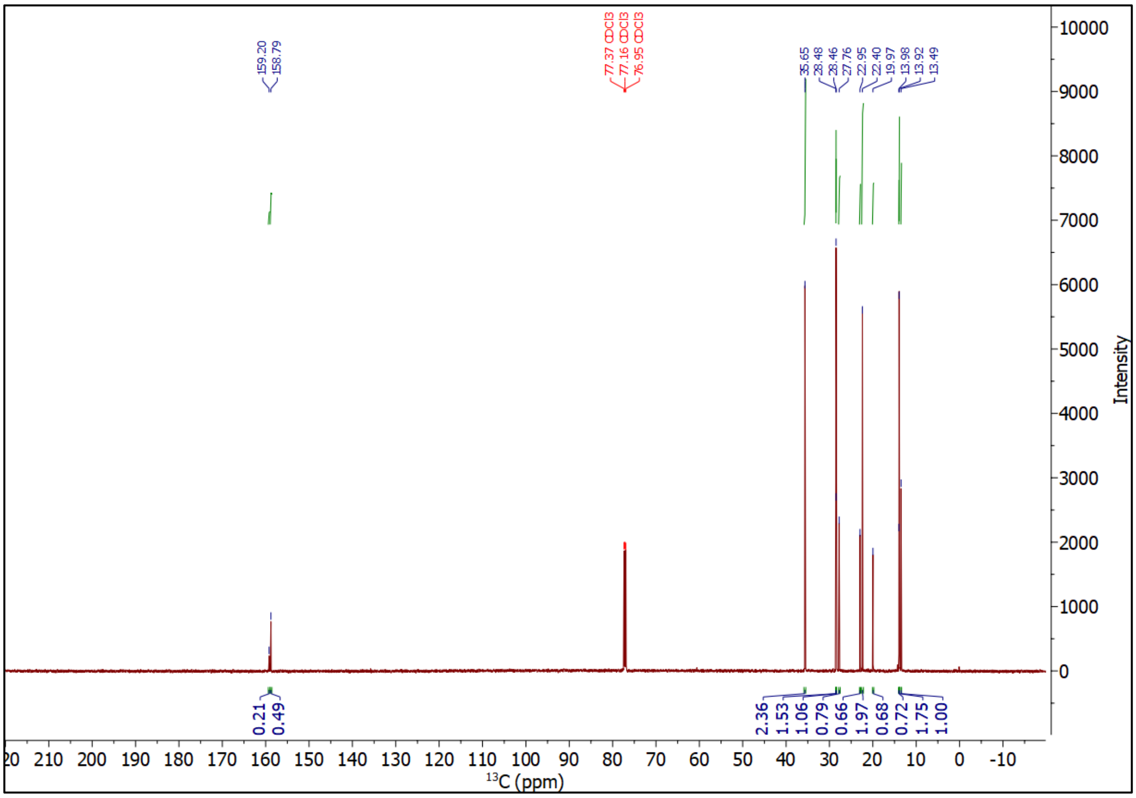

2-Methyl-3-propyl-1*H*-pyrrole (5)

^{1}H NMR (600 MHz, $CDCl_3$)

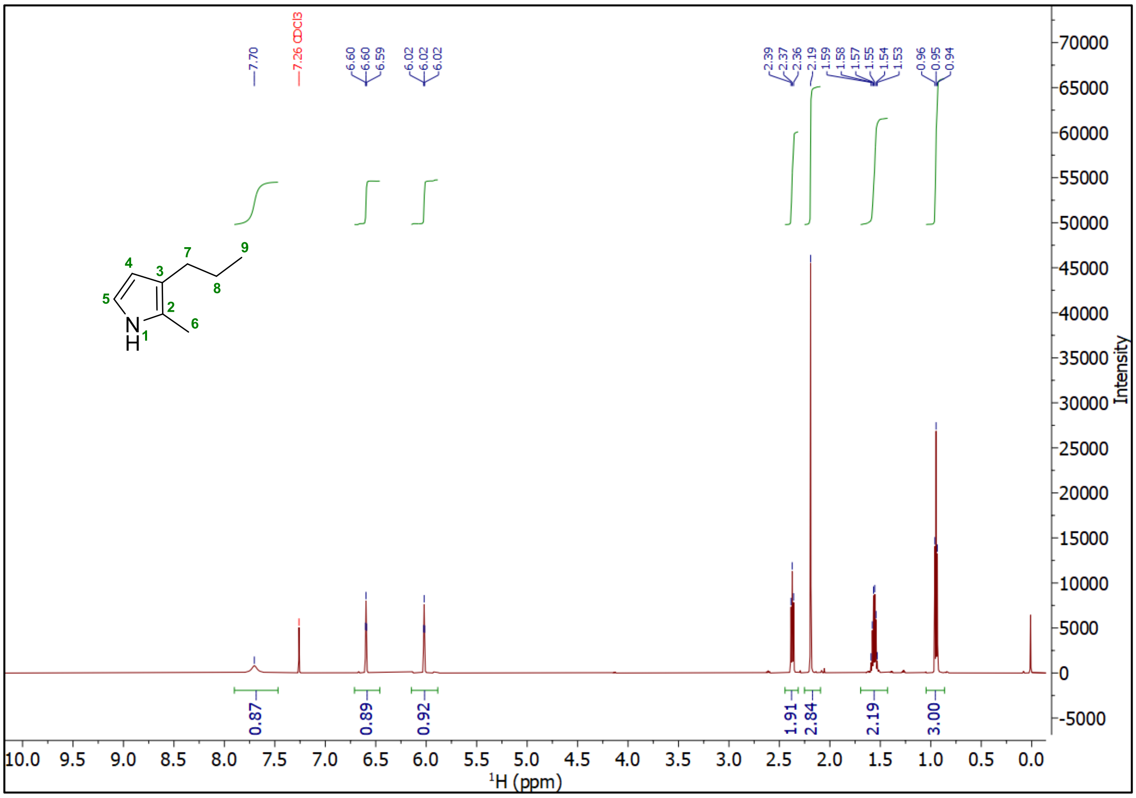

^{13}C NMR (151 MHz, $CDCl_3$)

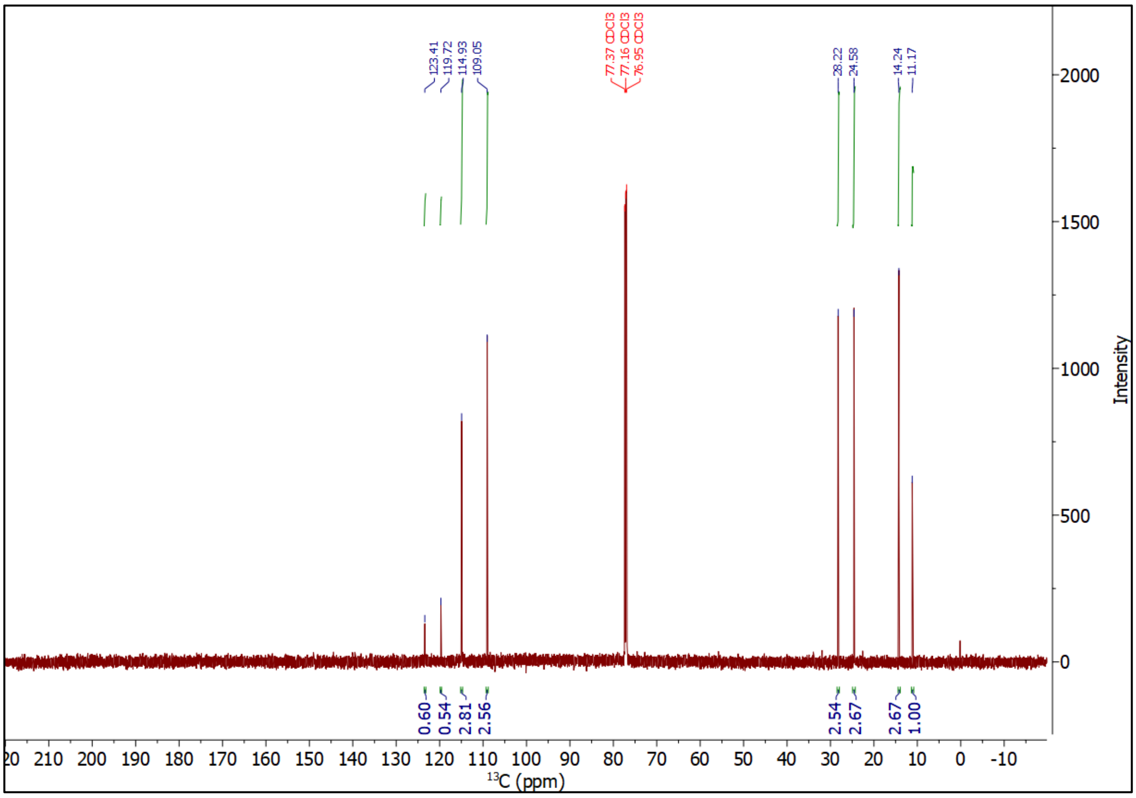

5-Methyl-4-propyl-1*H*-pyrrole-2-carbaldehyde (7)

^{1}H NMR (600 MHz, $CDCl_3$)

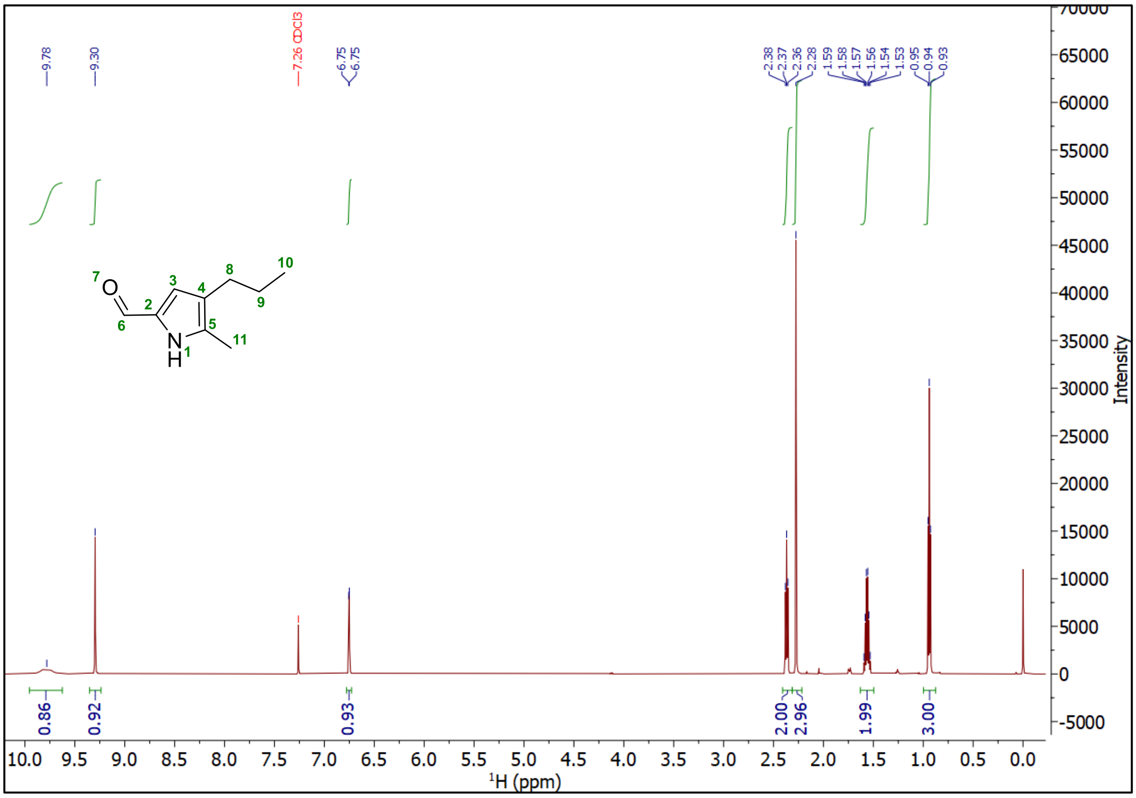

^{13}C NMR (151 MHz, $CDCl_3$)

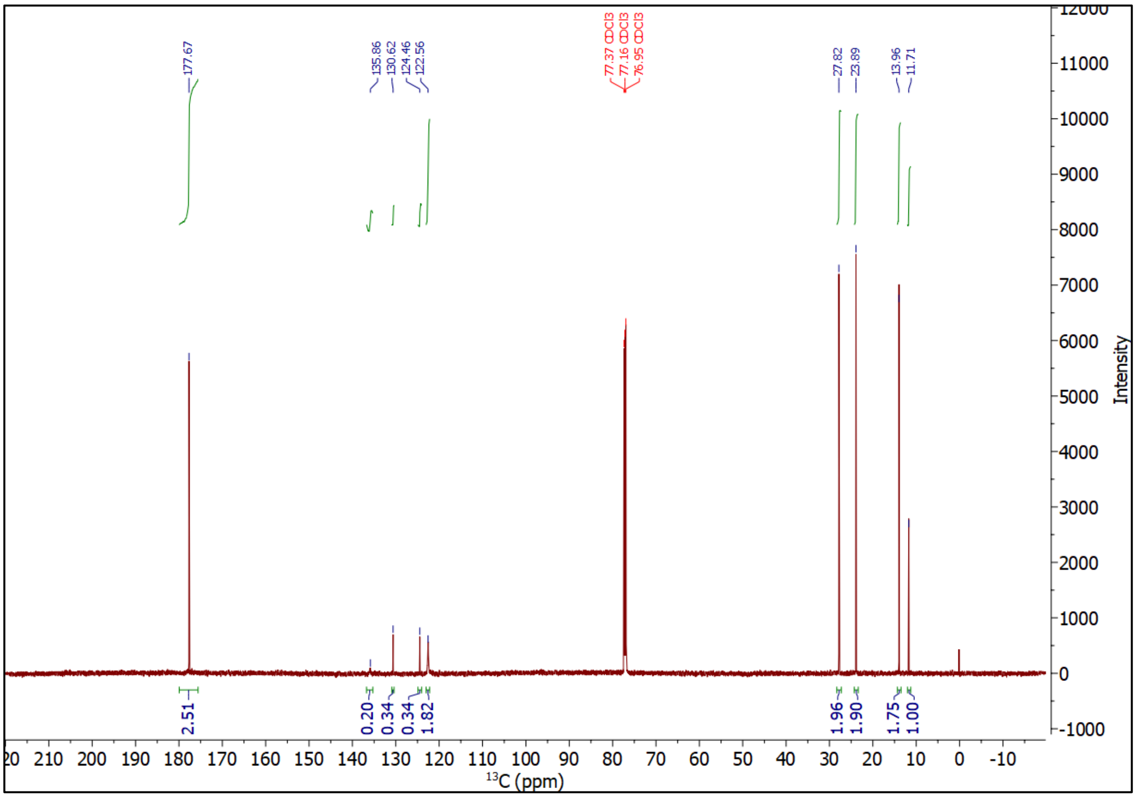

(*Z*)-4-Methoxy-5-((5-methyl-4-propyl-1*H*-pyrrol-2-yl)methylene)-1,5-dihydro-2*H*-pyrrol-2-one (9)

^{1}H NMR (600 MHz, $CDCl_3$)

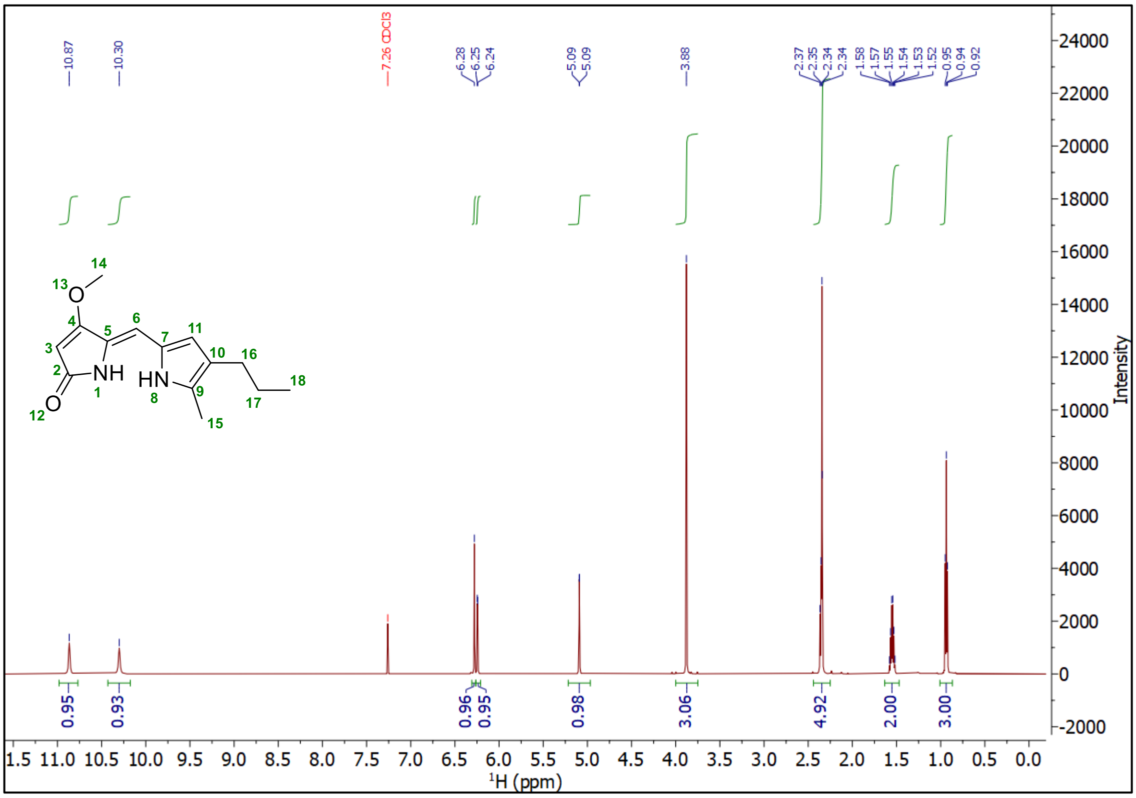

^{13}C NMR (151 MHz, $CDCl_3$)

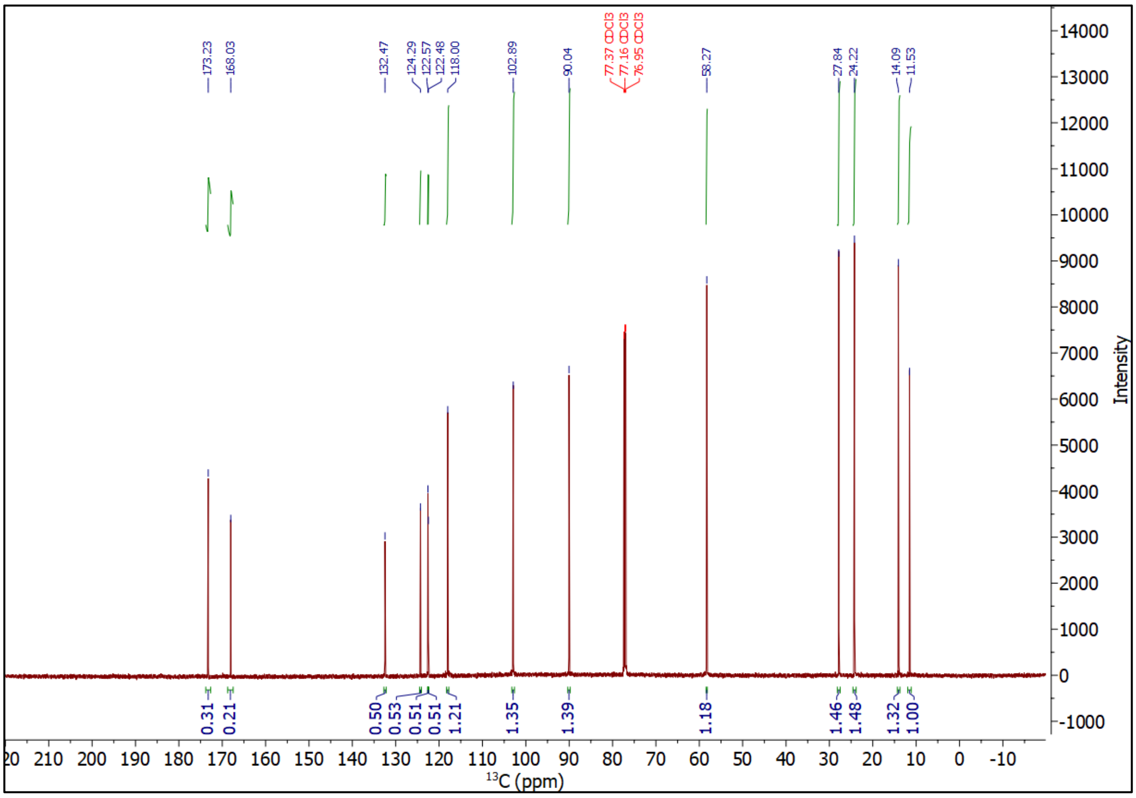

(*Z*)-4-Methoxy-5-((5-methyl-4-propyl-2*H*-pyrrol-2-ylidene)methyl)-1*H*-pyrrol-2-yl trifluoromethanesulfonate (10)

^{1}H NMR (300 MHz, $CDCl_3$)

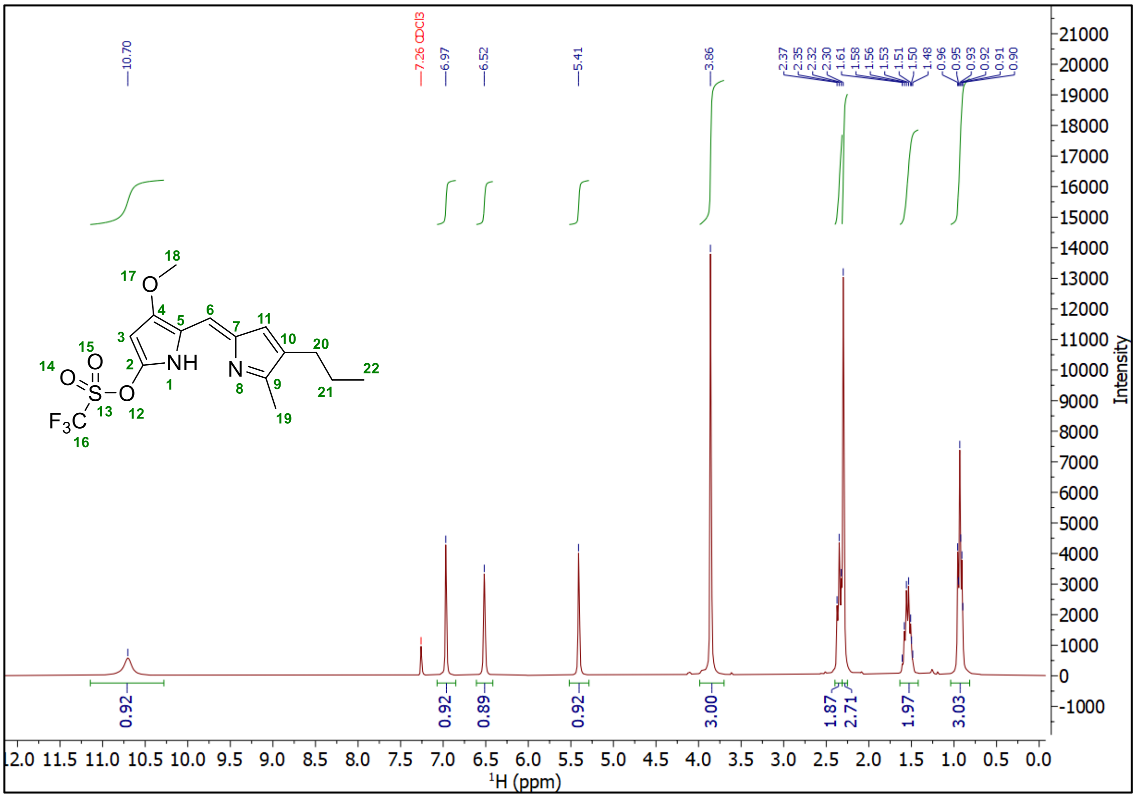

^{13}C NMR (76 MHz, $CDCl_3$)

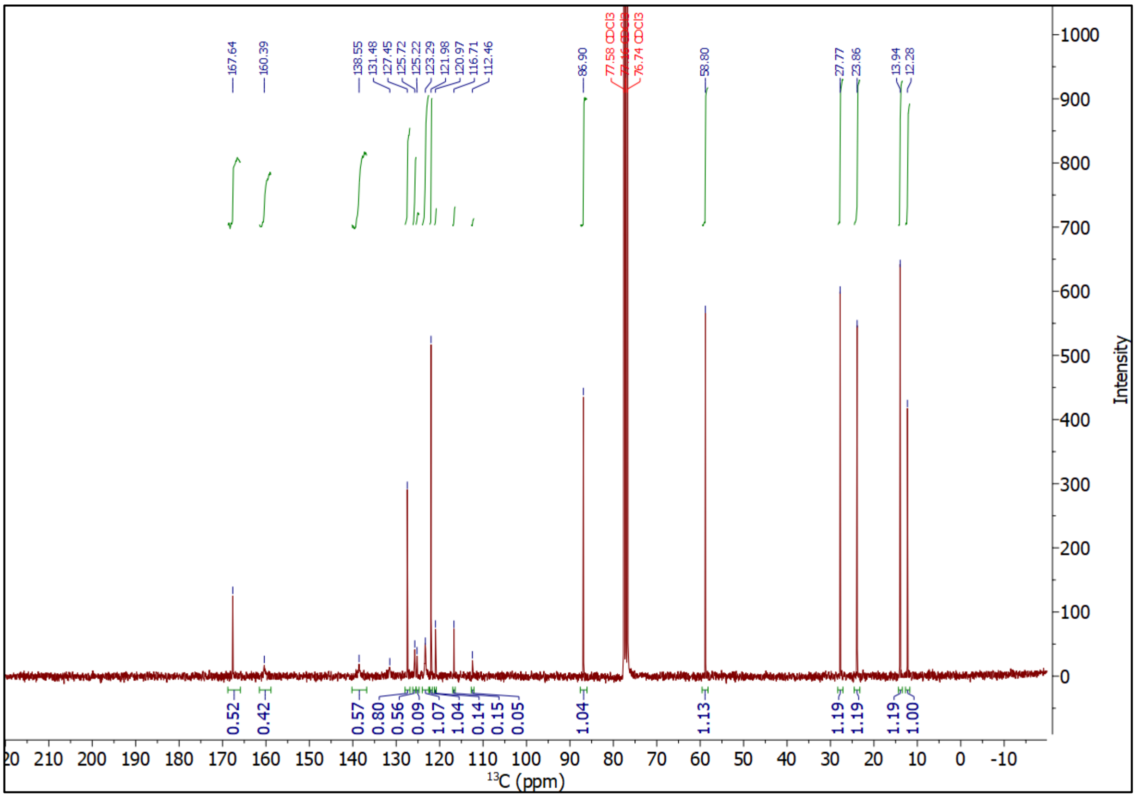

Ethyl 5-oxo-5-(1*H*-pyrrol-2-yl)pentanoate (12)

^{1}H NMR (300 MHz, $CDCl_3$)

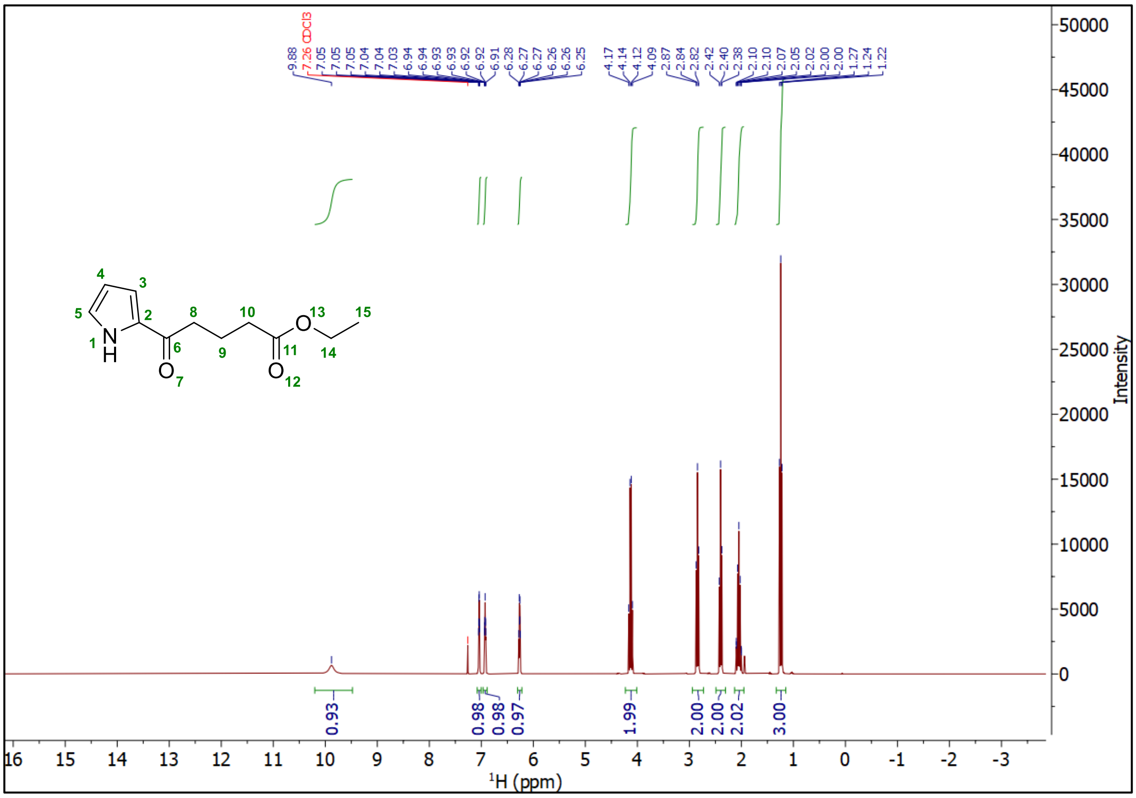

^{13}C NMR (76 MHz, $CDCl_3$)

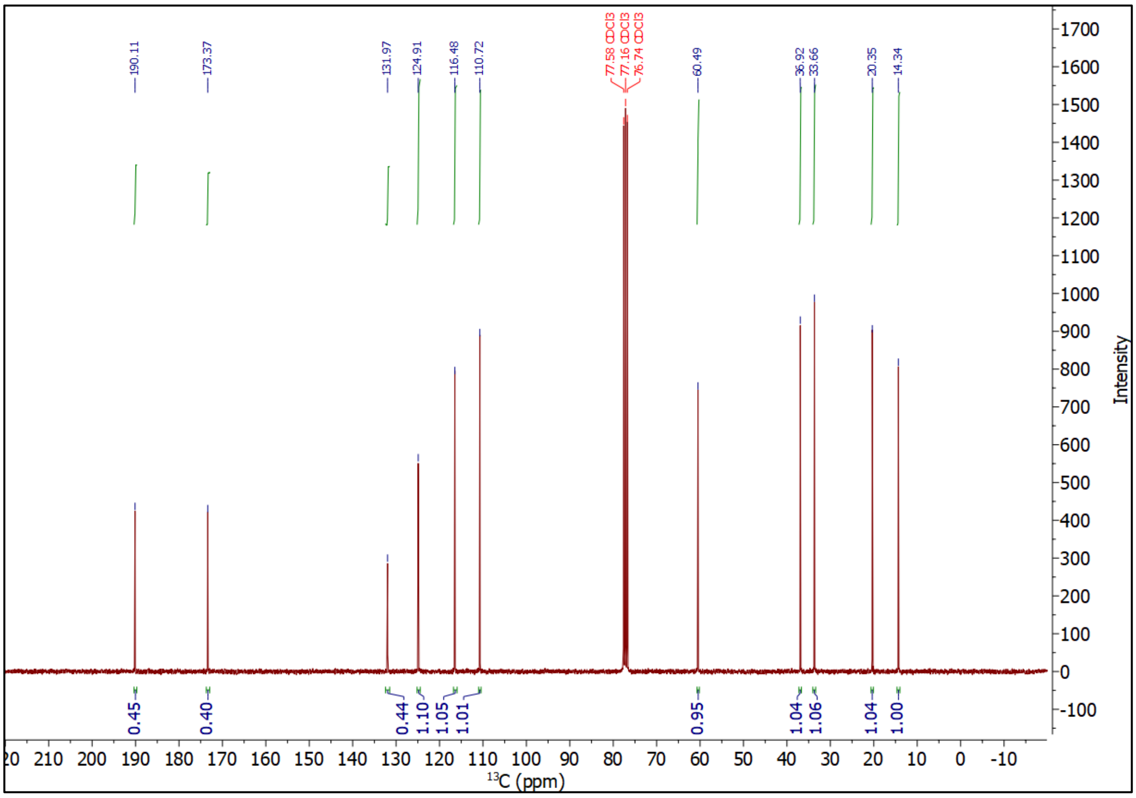

5-(1*H*-Pyrrol-2-yl)pentan-1-ol (13)

^{1}H NMR (300 MHz, $CDCl_3$)

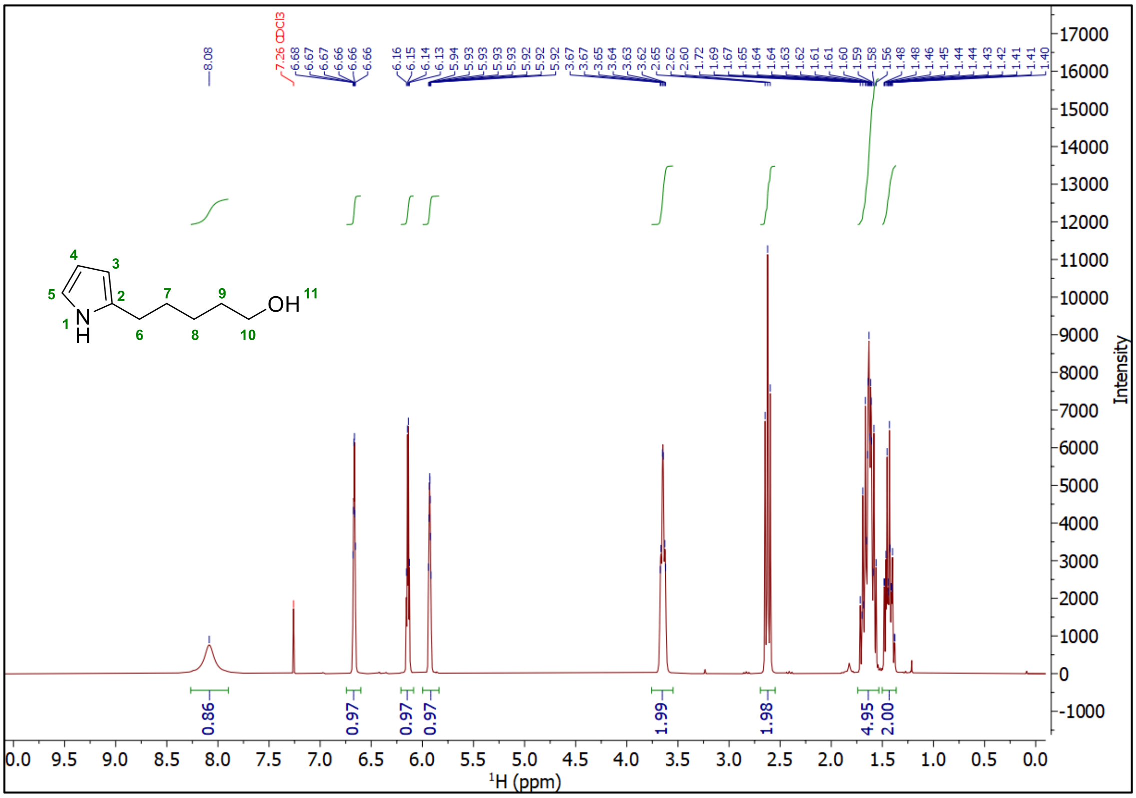

^{13}C NMR (76 MHz, $CDCl_3$)

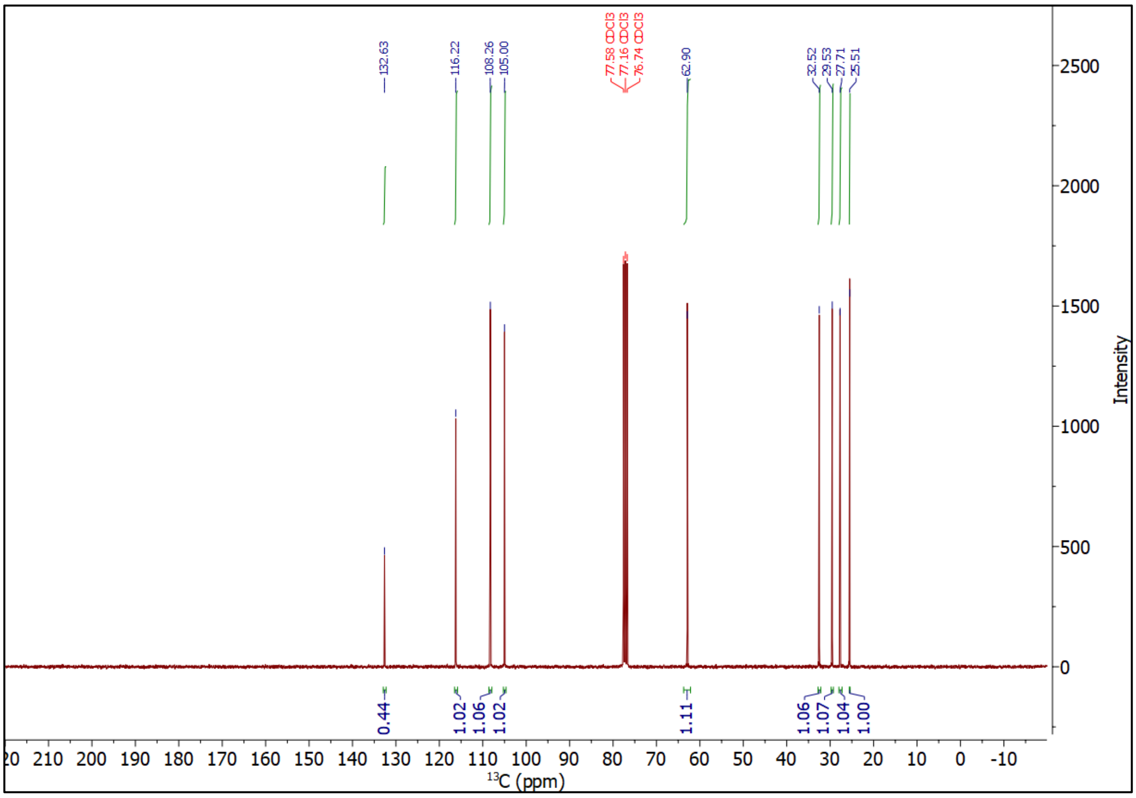

5-(1*H*-Pyrrol-2-yl)pentyl 4-methylbenzenesulfonate (14)

^{1}H NMR (600 MHz, $CDCl_3$)

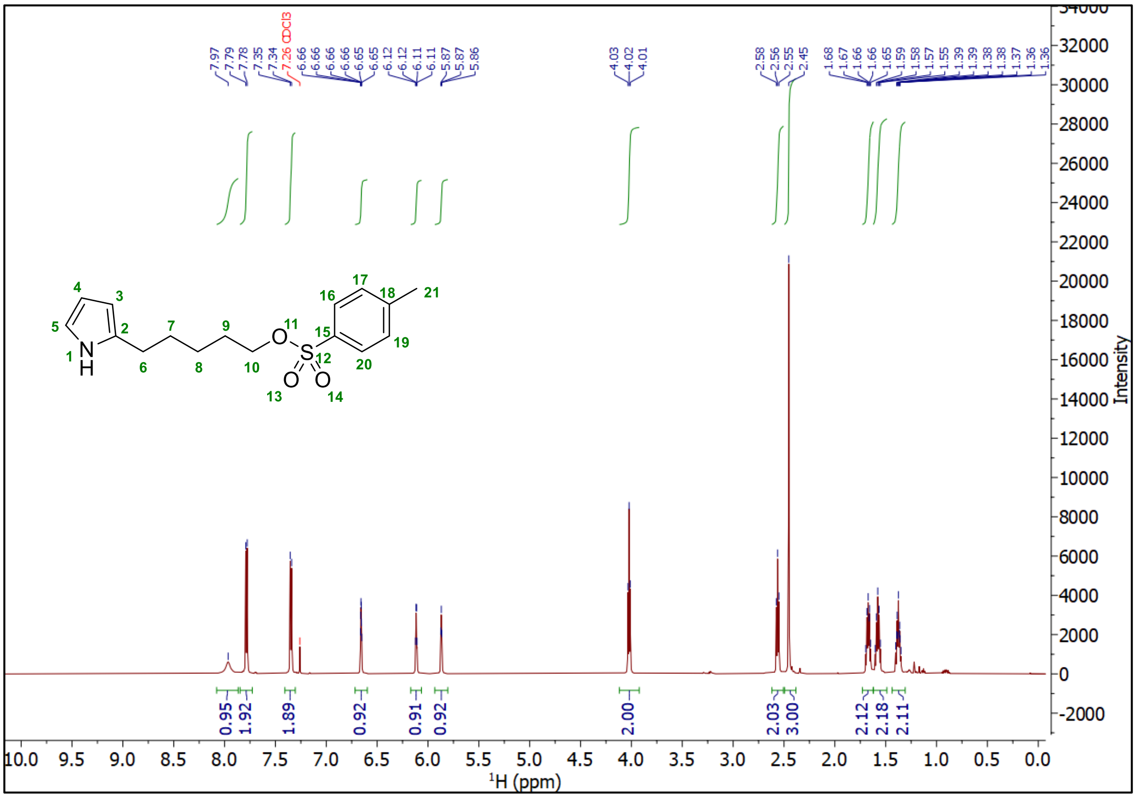

^{13}C NMR (151 MHz, $CDCl_3$)

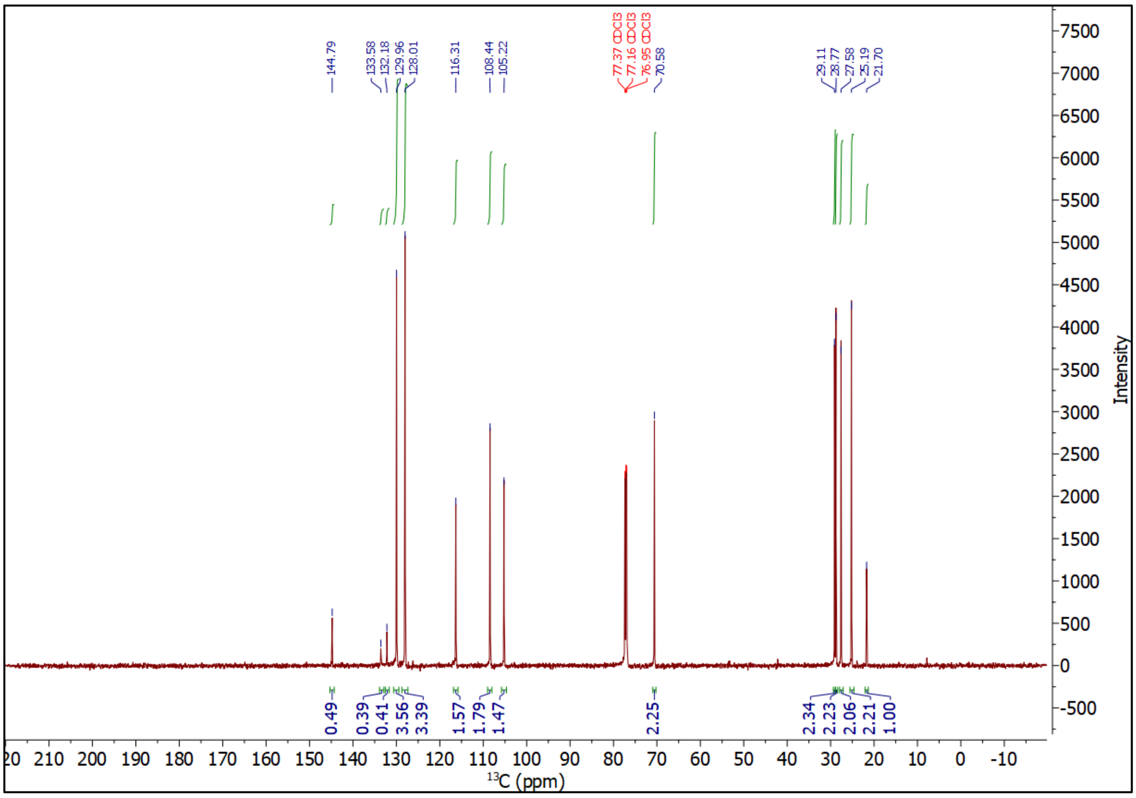

***tert*-Butyl 2-(5-(tosyloxy)pentyl)-1*H*-pyrrole-1-carboxylate (15)**

^{1}H NMR (300 MHz, $CDCl_3$)

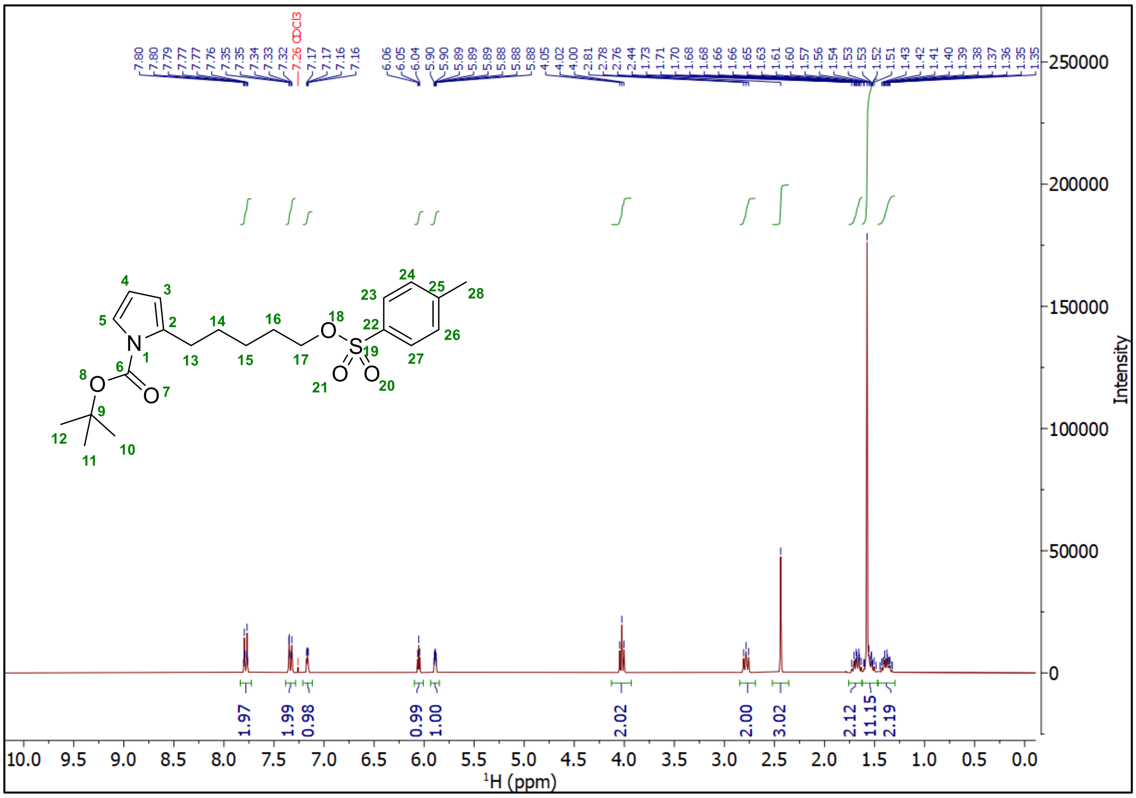

^{13}C NMR (76 MHz, $CDCl_3$)

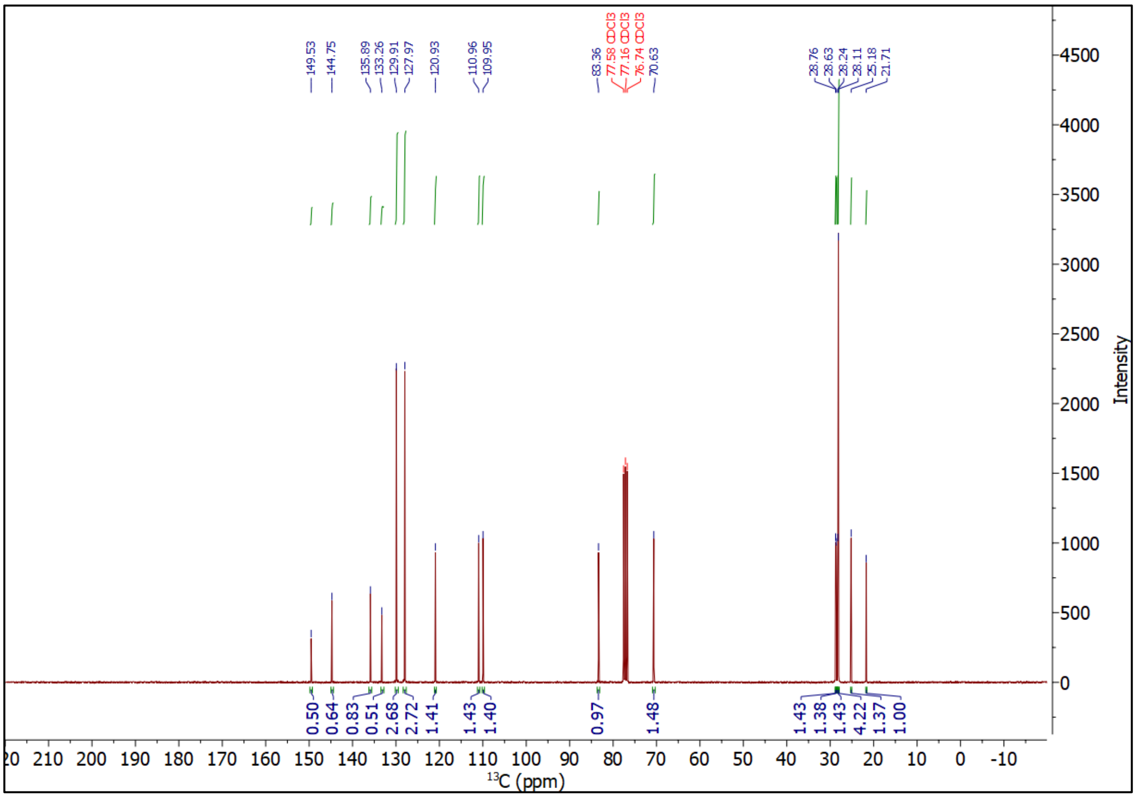

***tert*-Butyl 2-(5-azidopentyl)-1*H*-pyrrole-1-carboxylate (16)**

^{1}H NMR (600 MHz, $CDCl_3$)

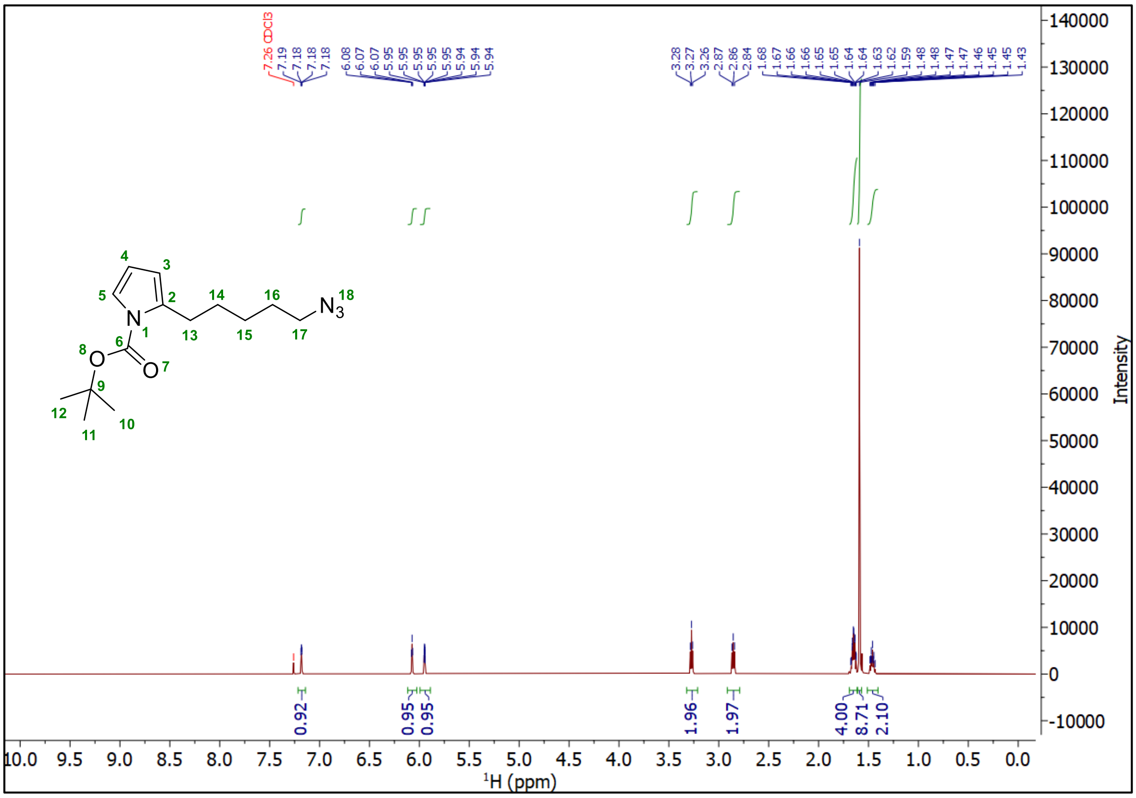

^{13}C NMR (151 MHz, $CDCl_3$)

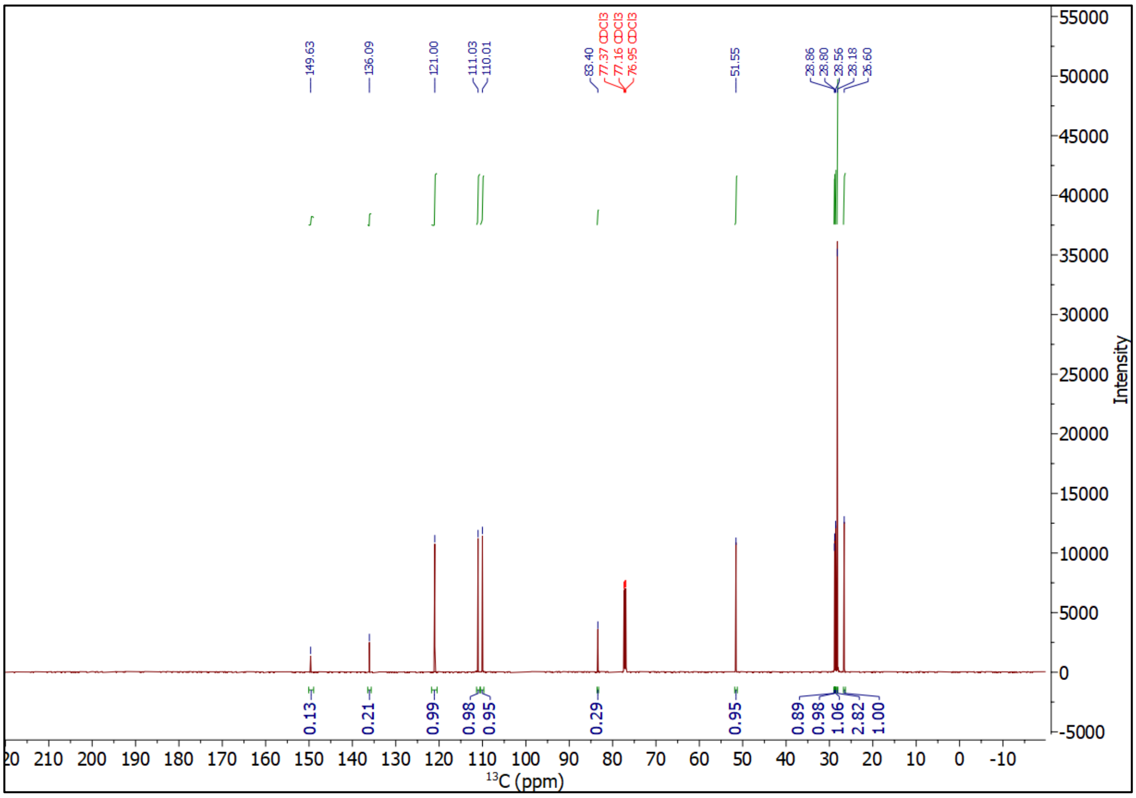

(5-(5-Azidopentyl)-1-(*tert*-butoxycarbonyl)-1*H*-pyrrol-2-yl)boronic acid (17)

^{1}H NMR (300 MHz, $CDCl_3$)

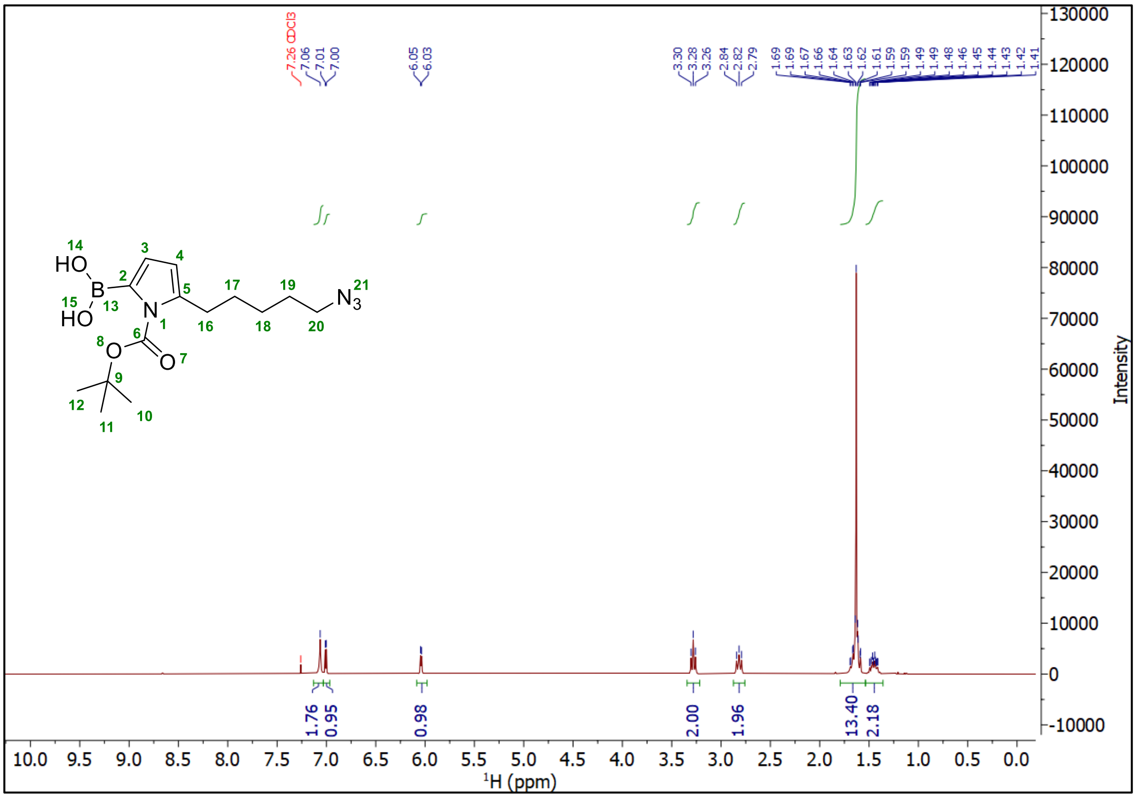

^{13}C NMR (76 MHz, $CDCl_3$)

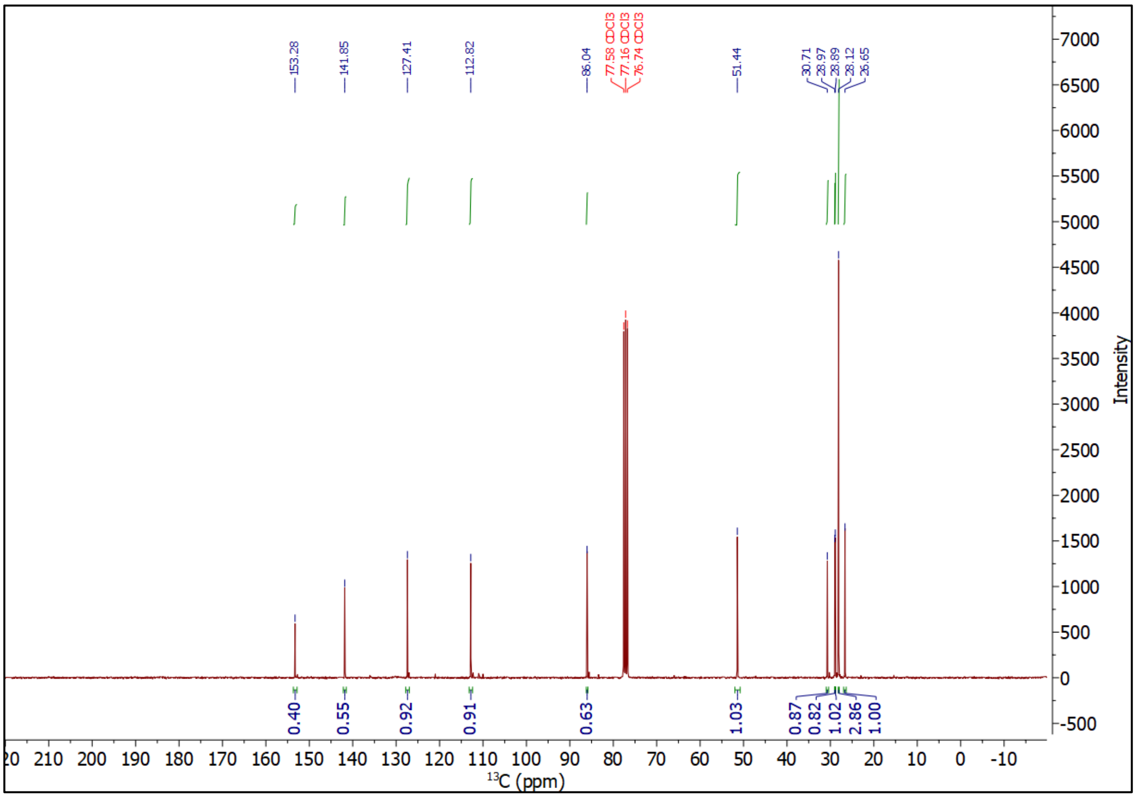

(*Z*)-5-(5-Azidopentyl)-4'-methoxy-5'-((5-methyl-4-propyl-1*H*-pyrrol-2-yl)methylene)-1*H*,5'*H*-[2,2'-bipyrrol]-1'-ium chloride (2·HCl)

^{1}H NMR (300 MHz, $CDCl_3$)

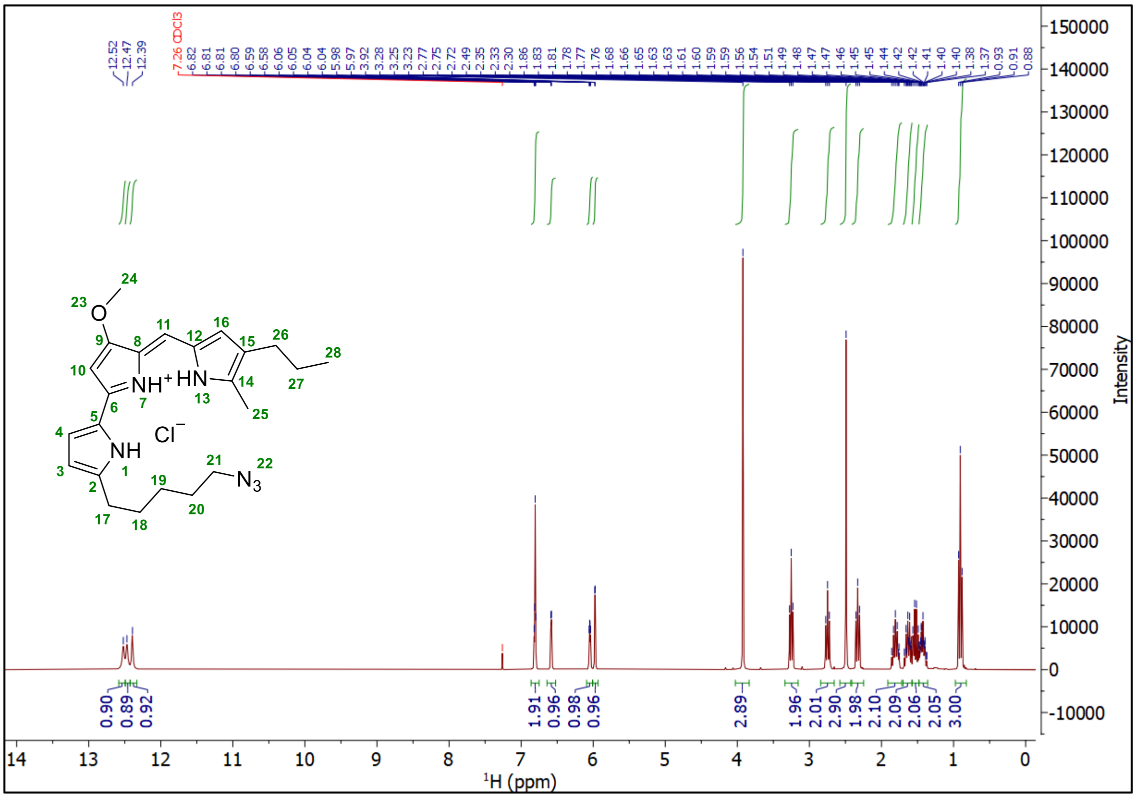

^{13}C NMR (76 MHz, $CDCl_3$)

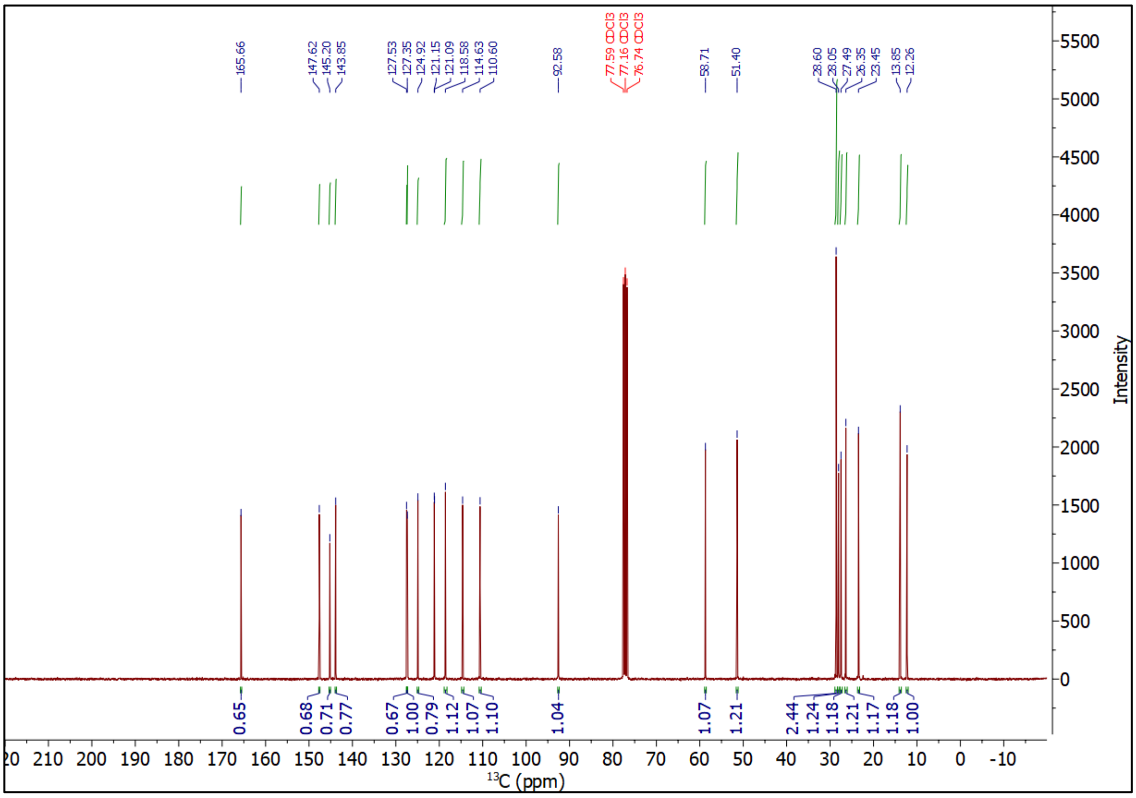

WILEY-VCH

3-(5-(5-Azidopentyl)-1*H*-pyrrol-2-yl)-5,5-difluoro-1-methoxy-7-methyl-8-propyl-5*H*-5λ4,6λ4-dipyrrolo[1,2-*c*:2',1'-*f*][1,3,2]diazaborinine (49)

^{1}H NMR (600 MHz, $CDCl_3$)

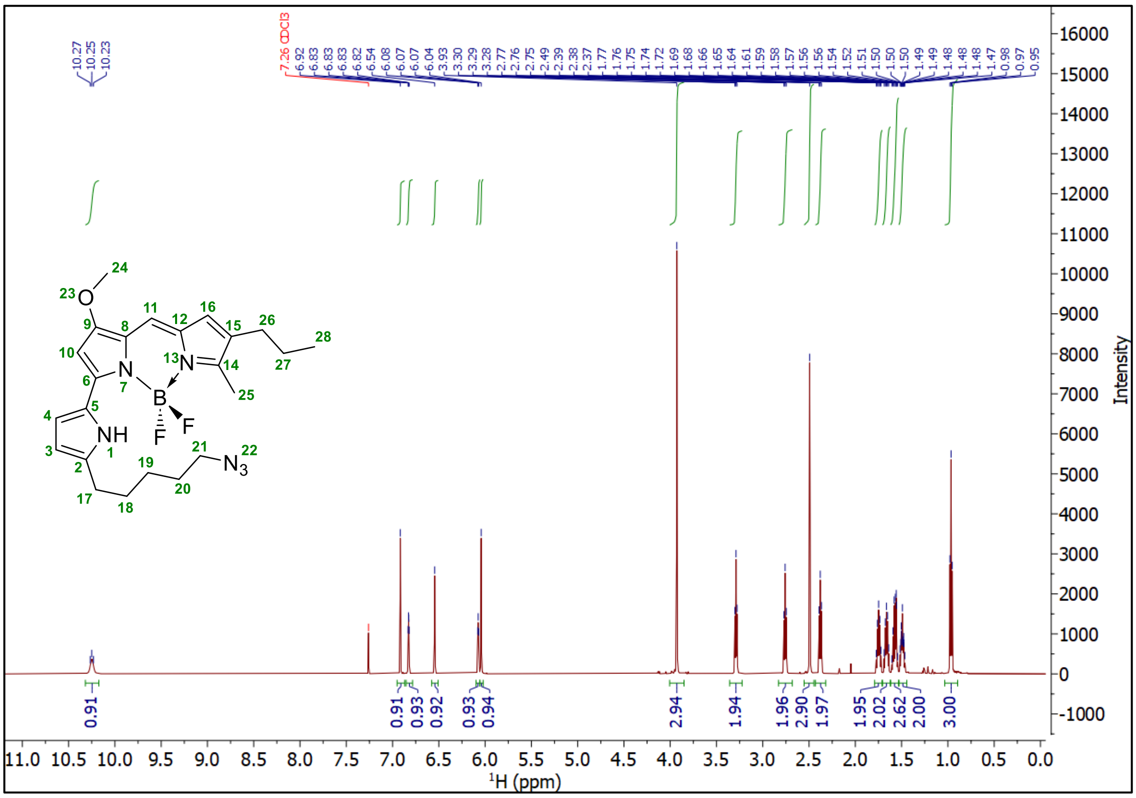

^{13}C NMR (151 MHz, $CDCl_3$)

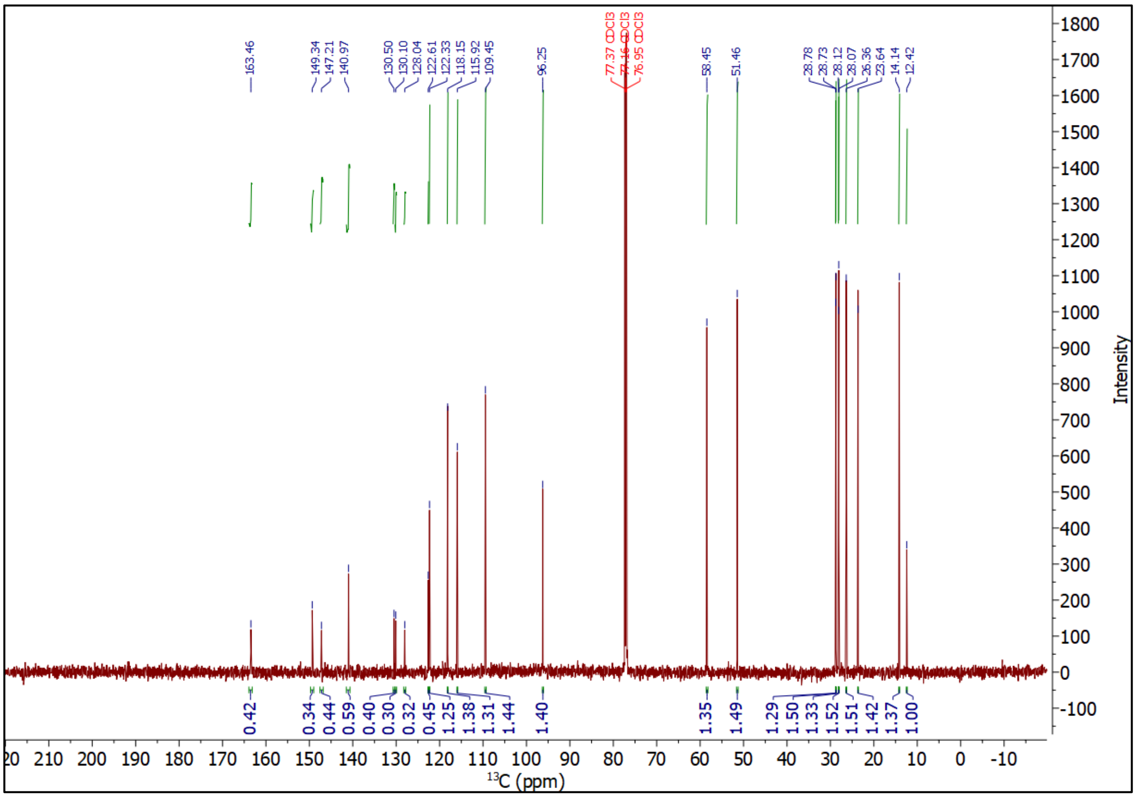

tert-Butyl 4-hydroxy-2-oxo-2,5-dihydro-1_H_-pyrrole-1-carboxylate (18)

^{1}H NMR (600 MHz, DMSO-d_6)

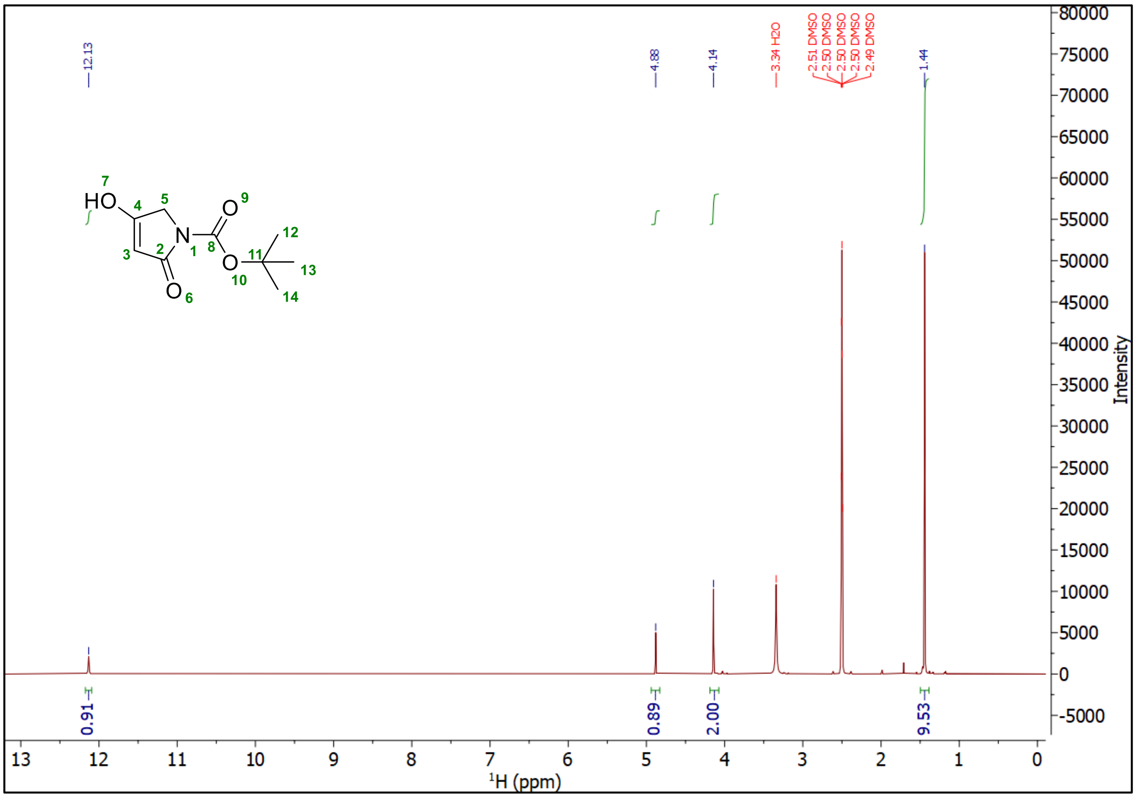

^{13}C NMR (151 MHz, DMSO-d_6)

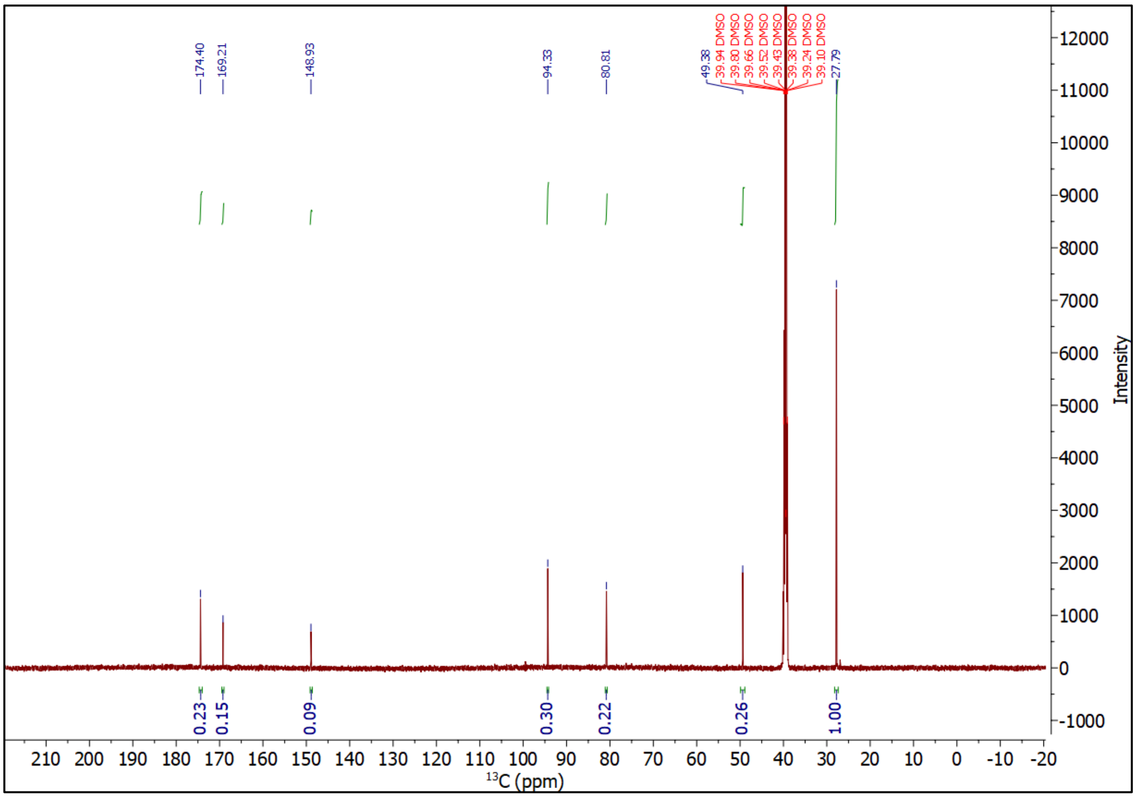

4-Azidobutan-1-ol (21)

^{1}H NMR (600 MHz, $CDCl_3$)

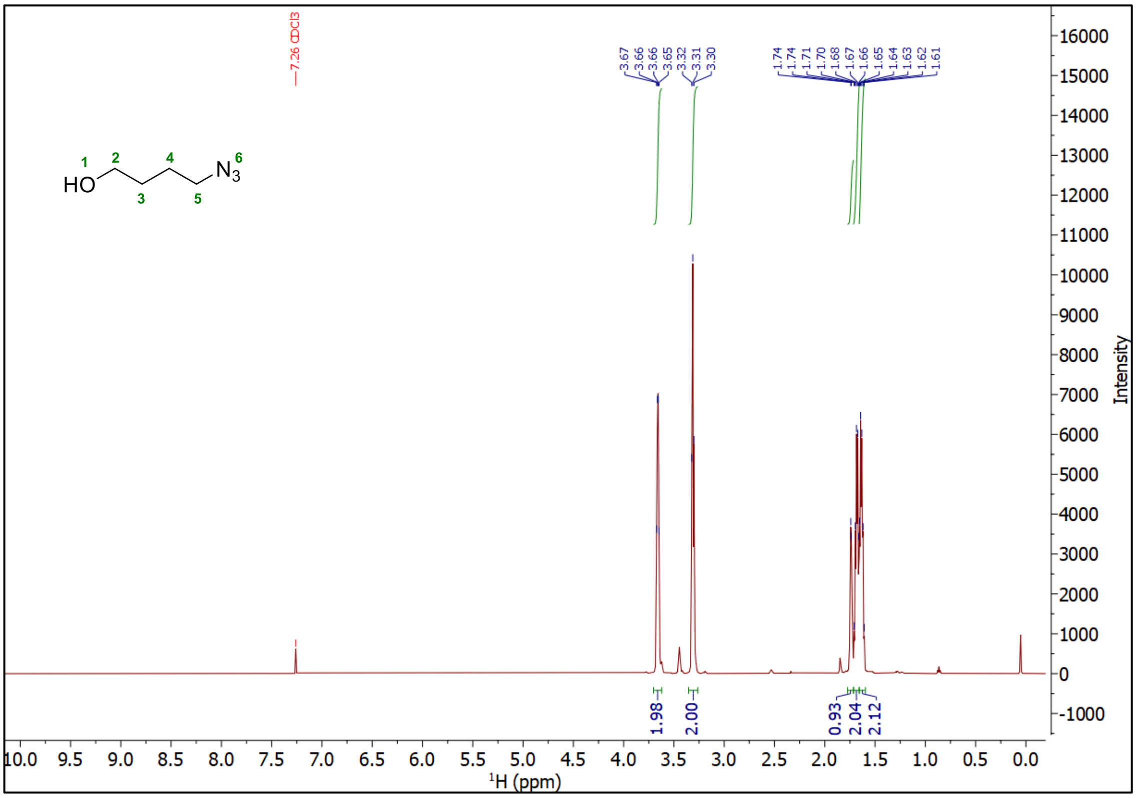

^{13}C NMR (151 MHz, $CDCl_3$)

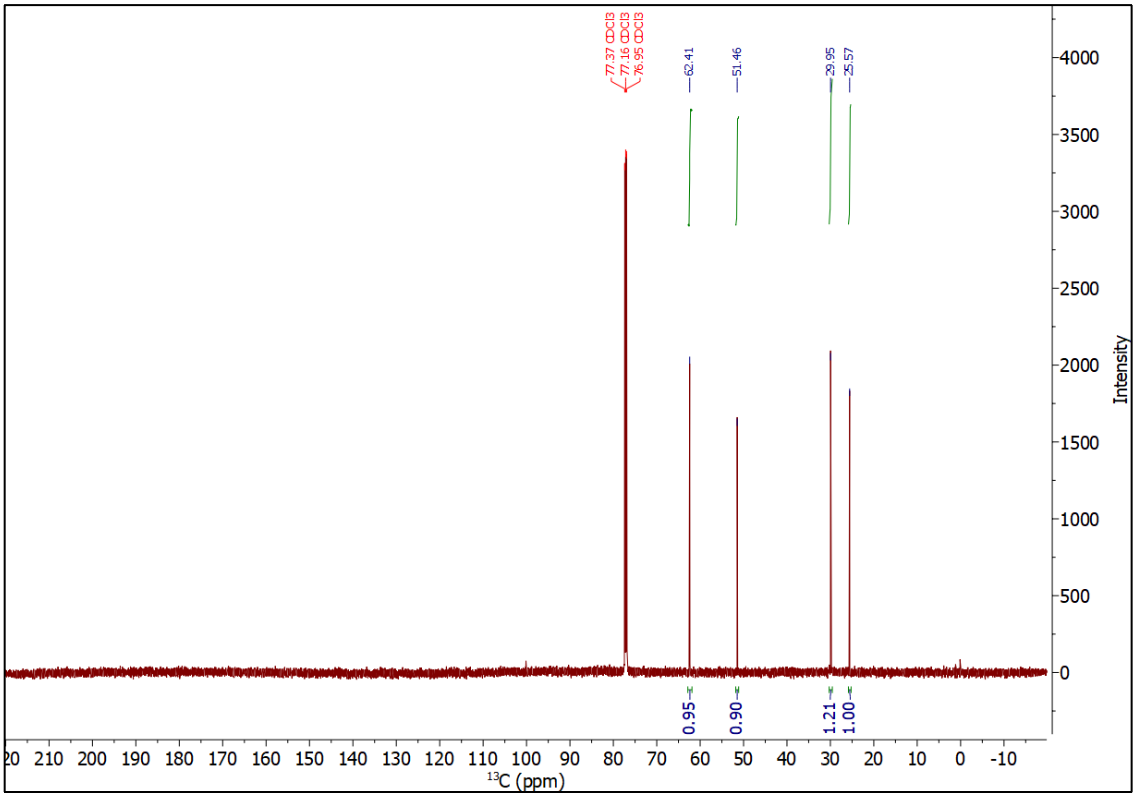

***tert*-Butyl 4-(4-azidobutoxy)-2-oxo-2,5-dihydro-1*H*-pyrrole-1-carboxylate (23)**

^{1}H NMR (600 MHz, $CDCl_3$)

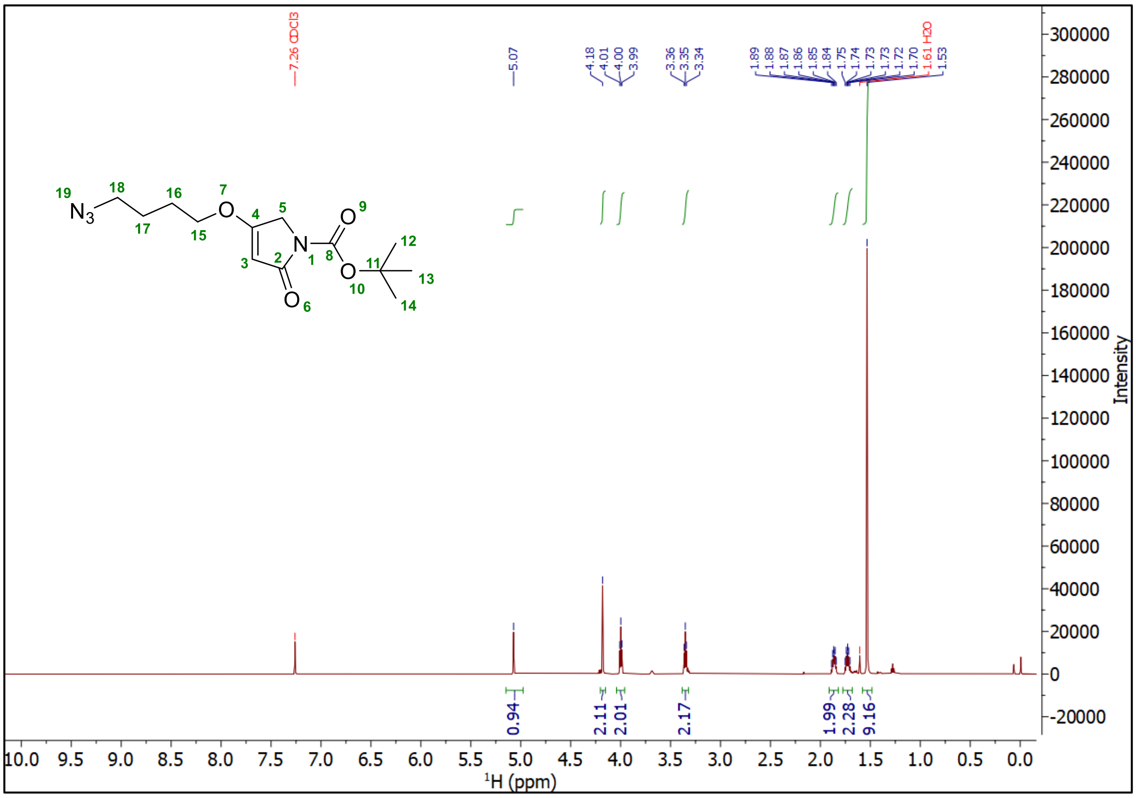

^{13}C NMR (151 MHz, $CDCl_3$)

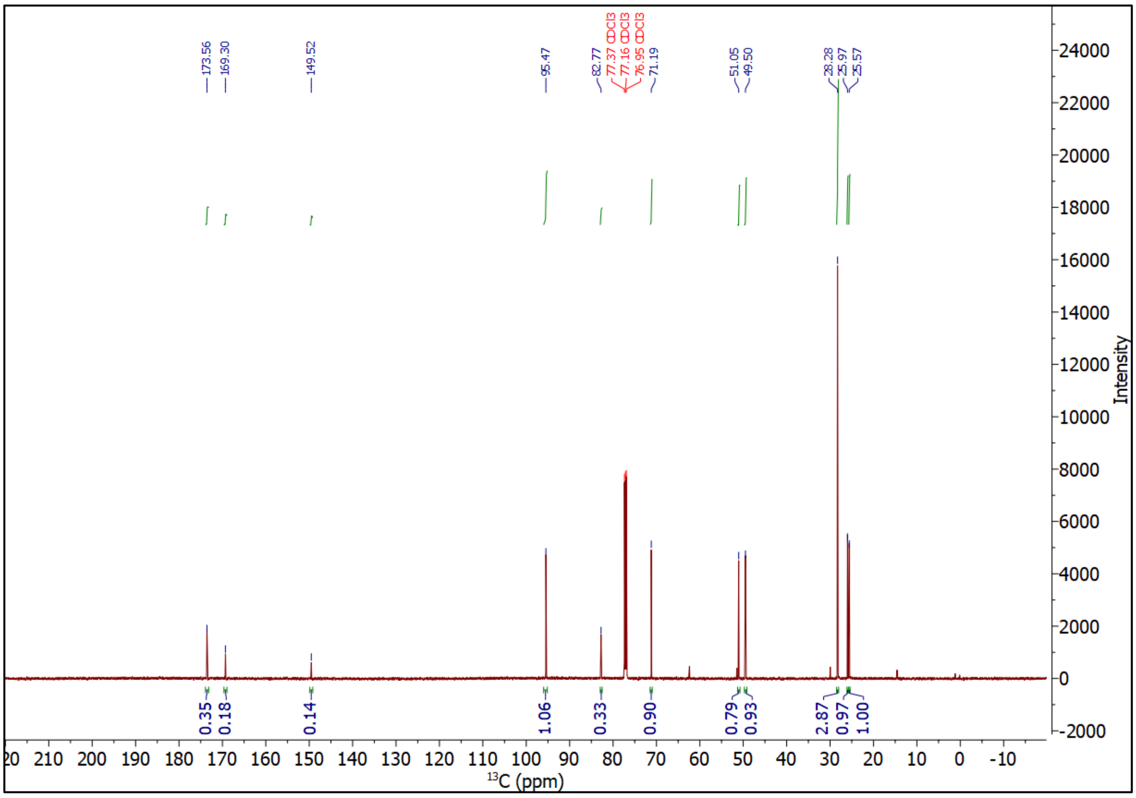

4-(4-Azidobutoxy)-1,5-dihydro-2*H*-pyrrol-2-one (24)

^{1}H NMR (600 MHz, $CDCl_3$)

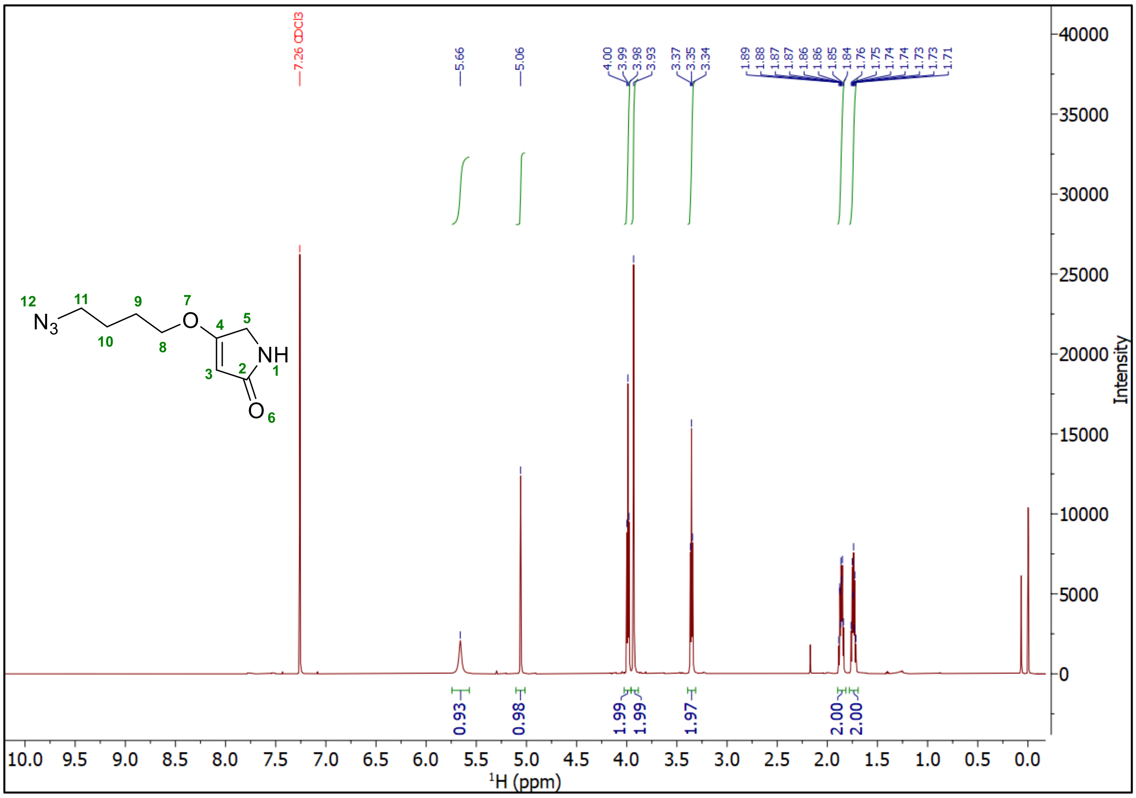

^{13}C NMR (151 MHz, $CDCl_3$)

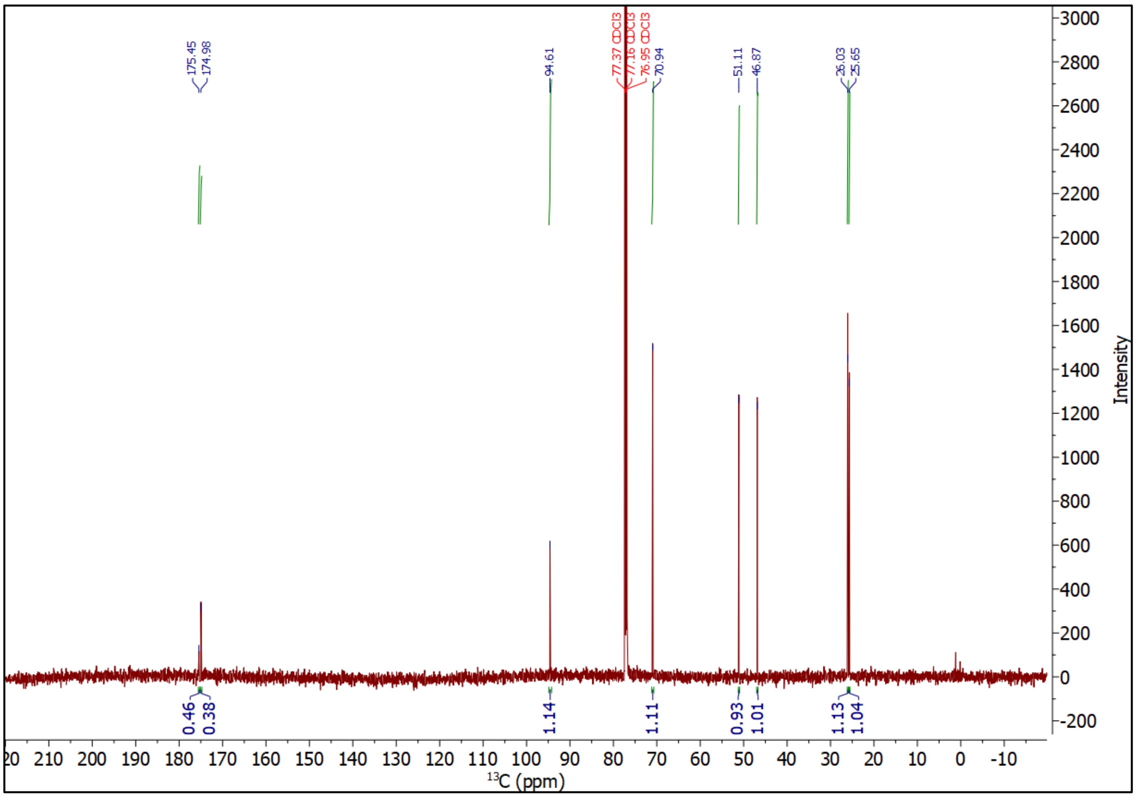

(*Z*)-4-(4-Azidobutoxy)-5-((5-methyl-4-propyl-1*H*-pyrrol-2-yl)methylene)-1,5-dihydro-2*H*-pyrrol-2-one (25)

^{1}H NMR (600 MHz, $CDCl_3$)

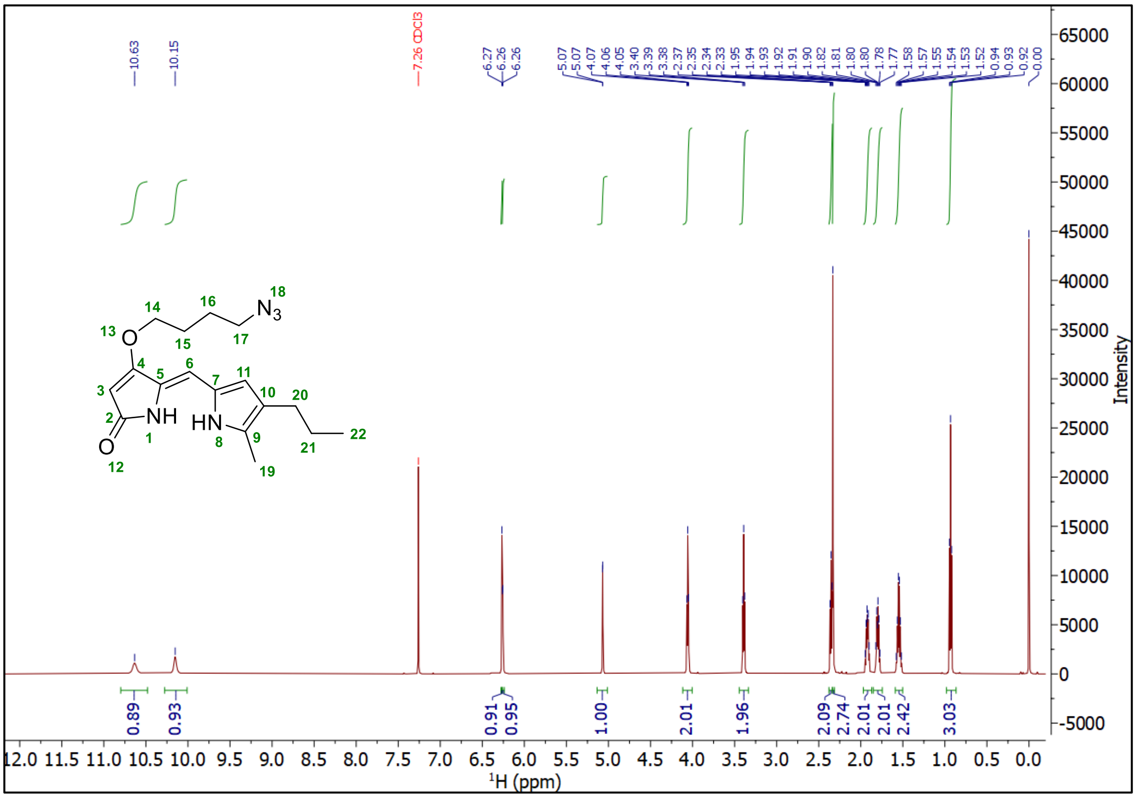

^{13}C NMR (151 MHz, $CDCl_3$)

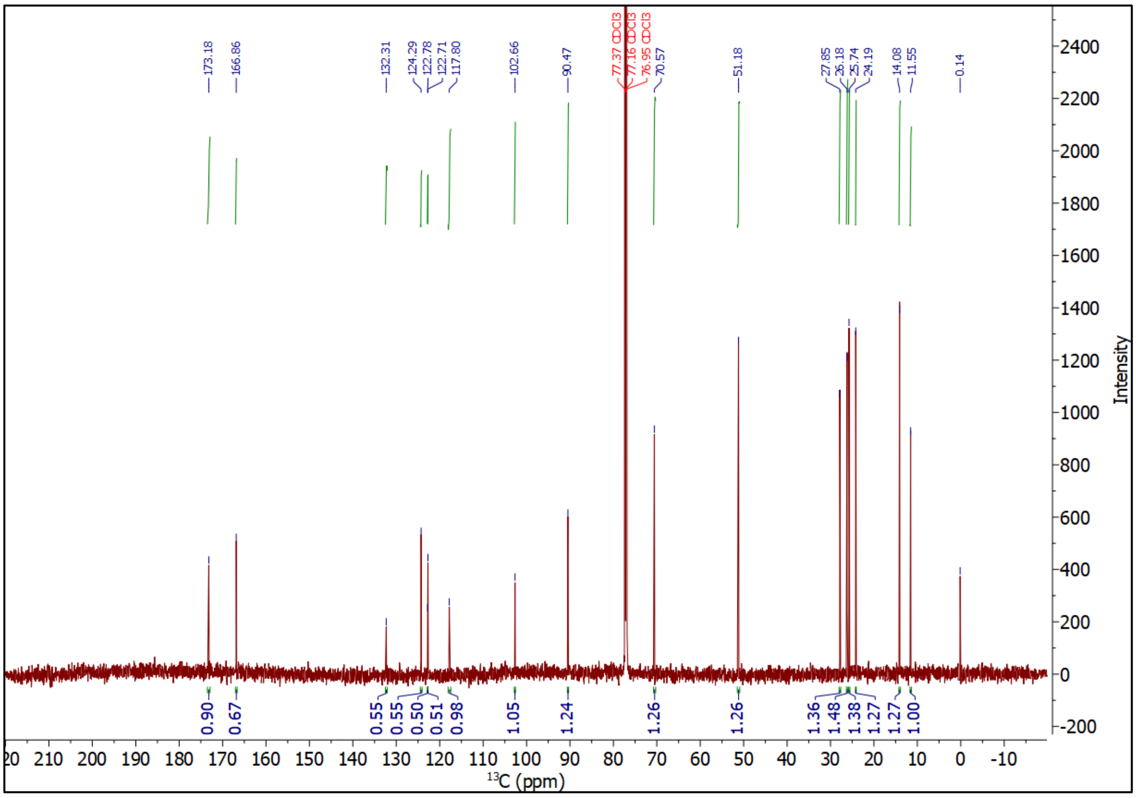

(*Z*)-4-(4-Azidobutoxy)-5-((5-methyl-4-propyl-2*H*-pyrrol-2-ylidene)methyl)-1*H*-pyrrol-2-yl trifluoromethanesulfonate (26)

^{1}H NMR (300 MHz, $CDCl_3$)

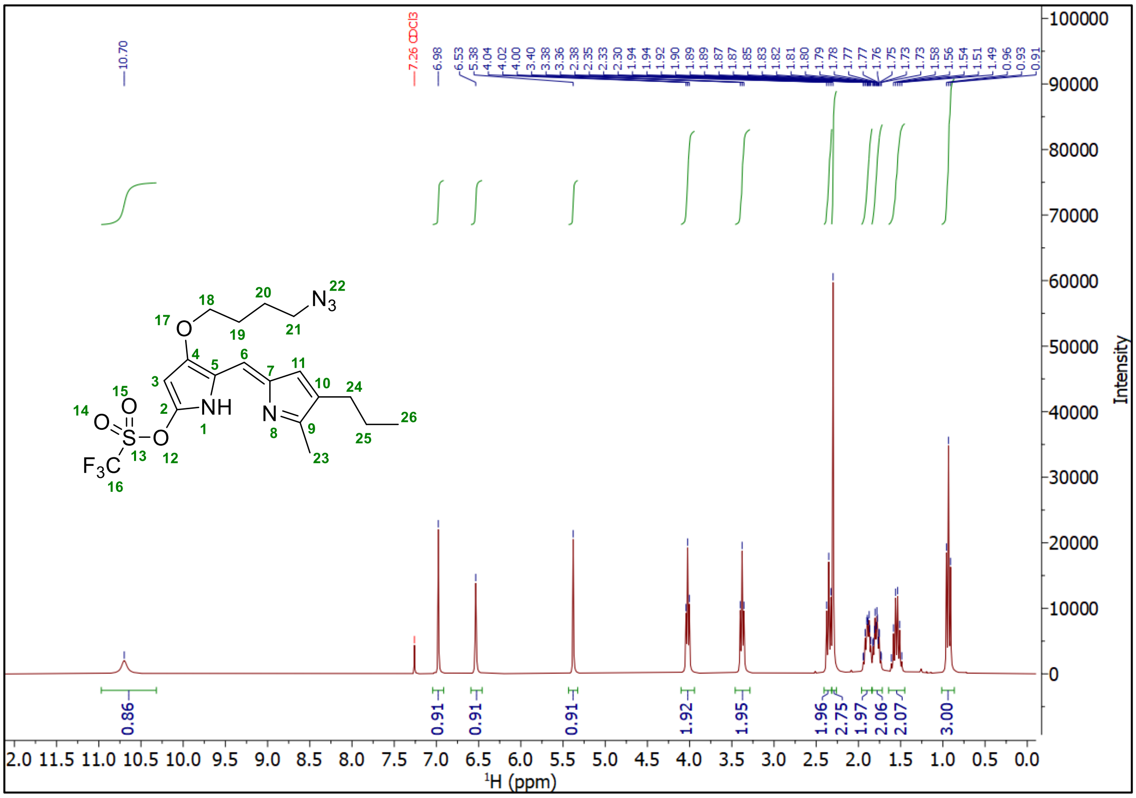

^{13}C NMR (76 MHz, $CDCl_3$)

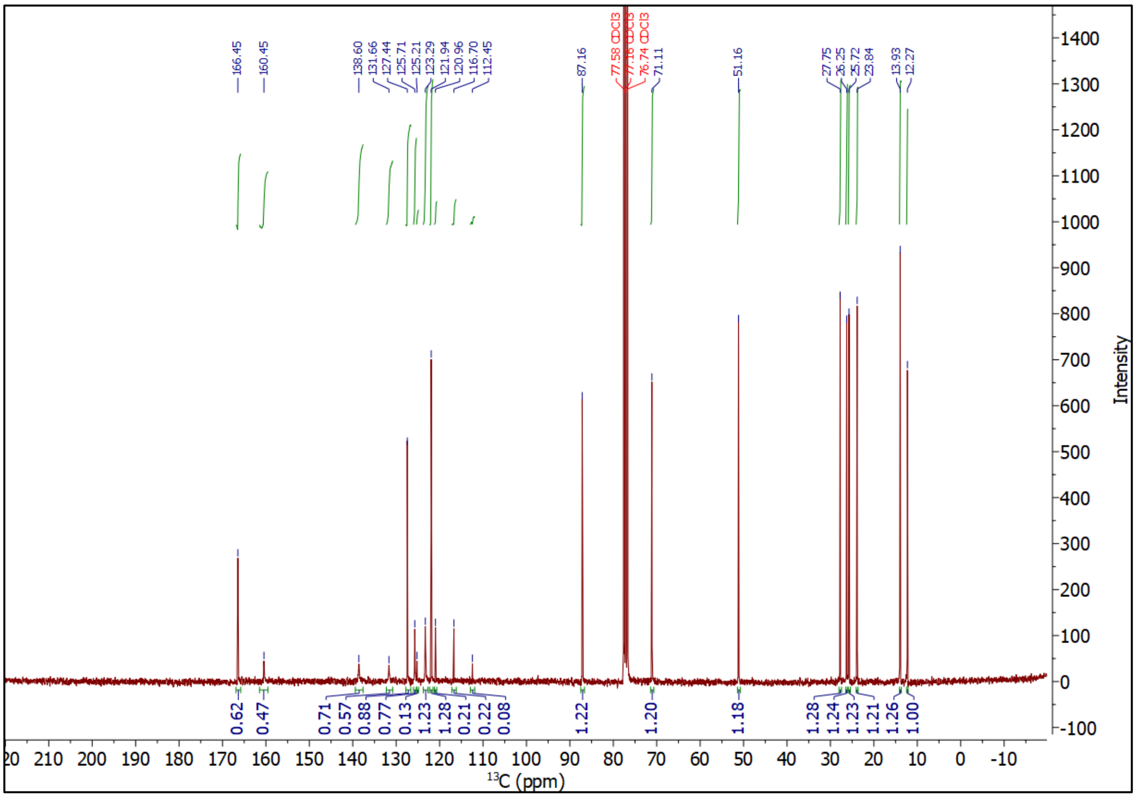

(1*H*-Pyrrol-2-yl)pentan-1-one (28)

^{1}H NMR (600 MHz, $CDCl_3$)

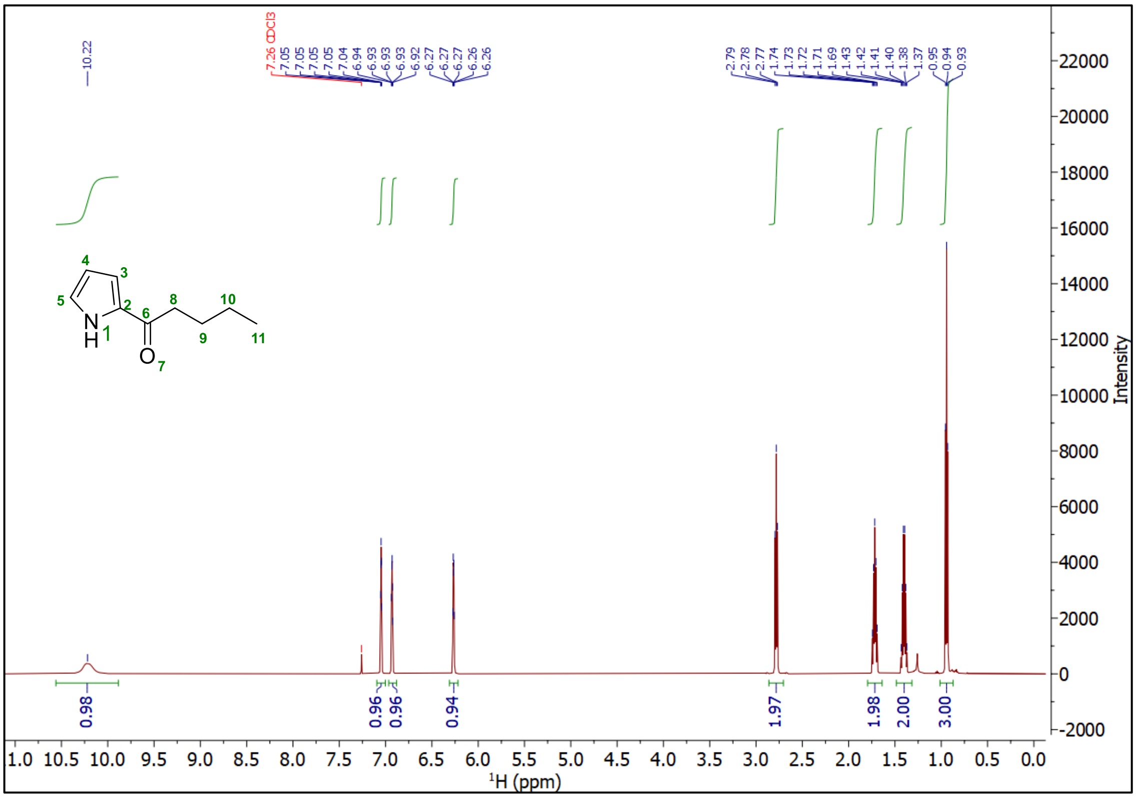

^{13}C NMR (151 MHz, $CDCl_3$)

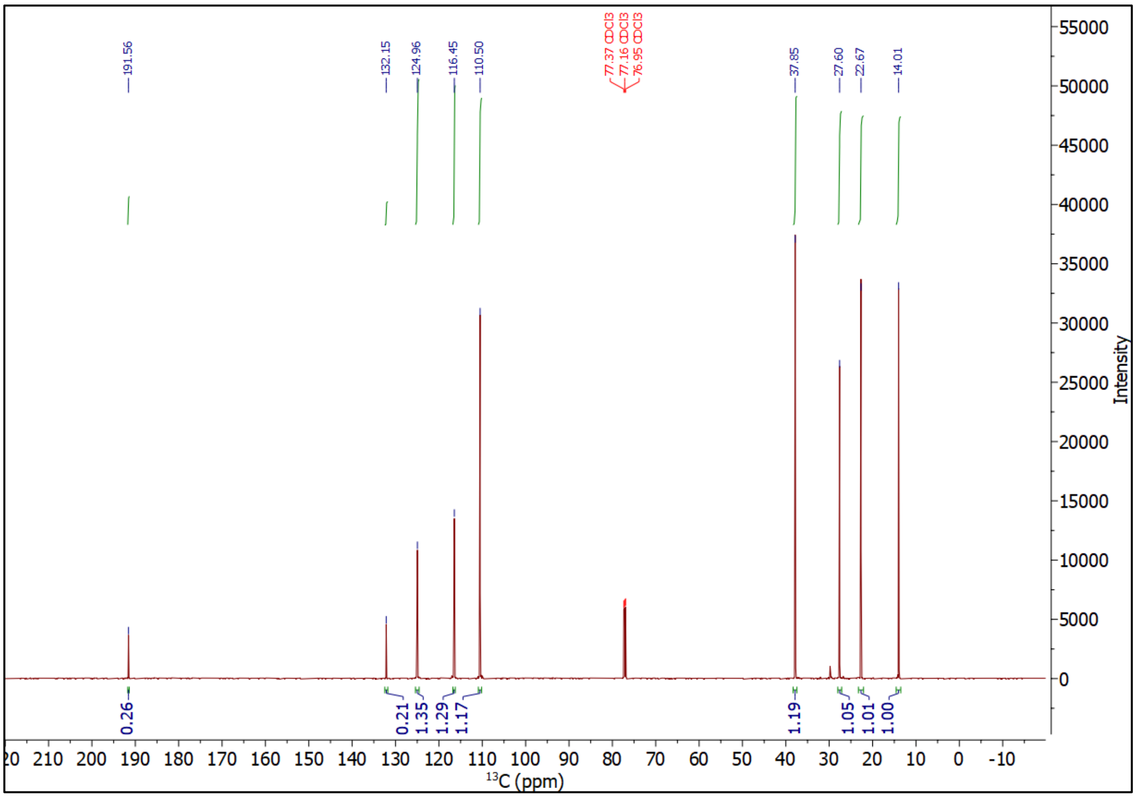

2-Pentyl-1*H*-pyrrole (29)

^{1}H NMR (600 MHz, $CDCl_3$)

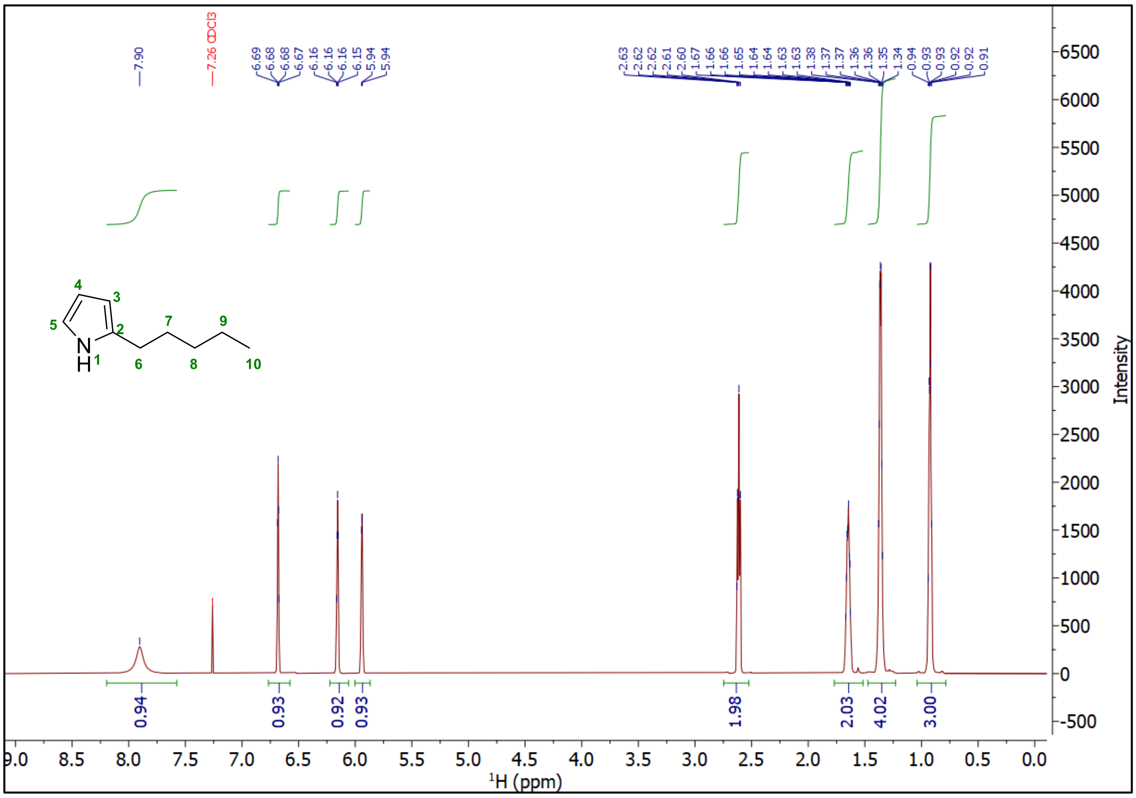

^{13}C NMR (151 MHz, $CDCl_3$)

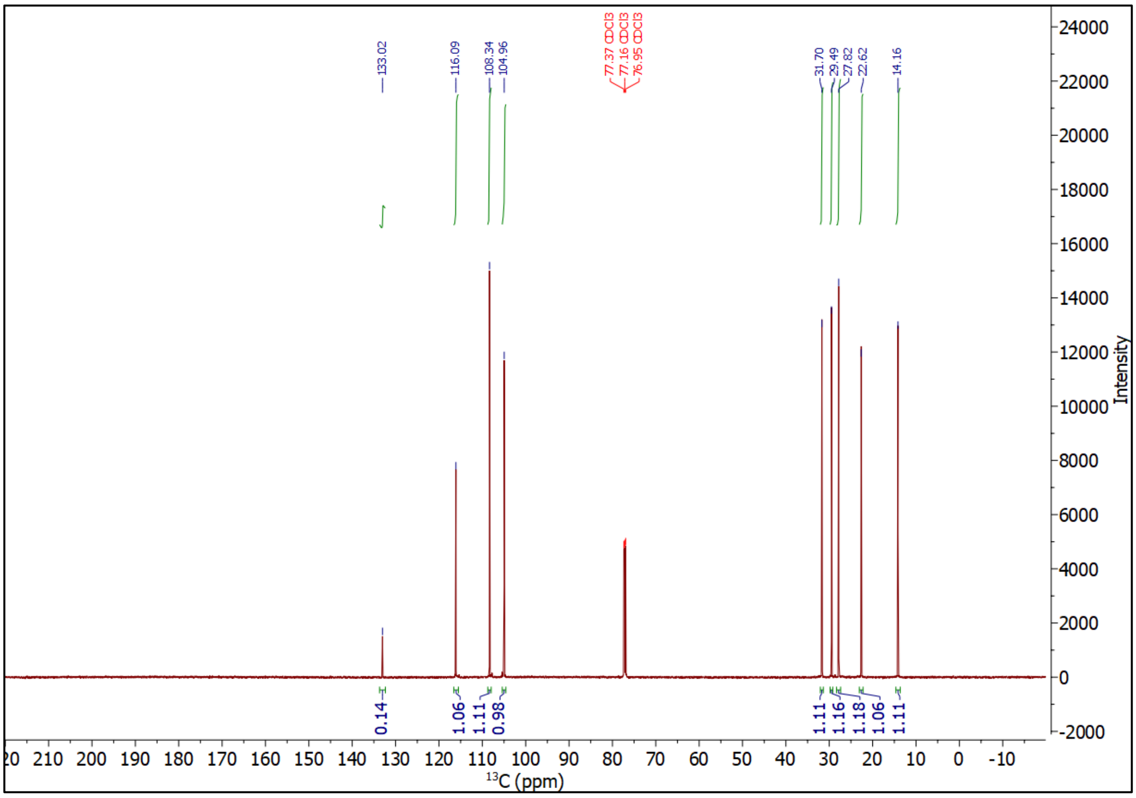

WILEY-VCH

***tert*-Butyl 2-pentyl-1*H*-pyrrole-1-carboxylate (30)**

^{1}H NMR (600 MHz, $CDCl_3$)

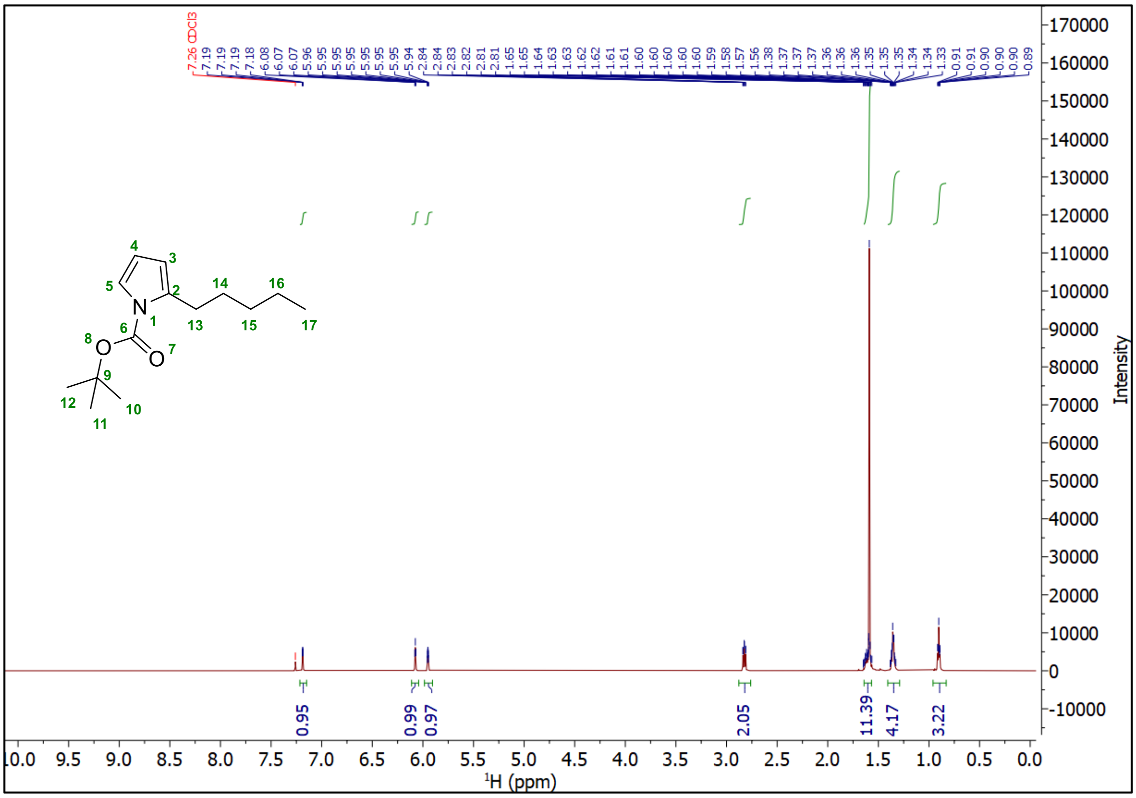

^{13}C NMR (151 MHz, $CDCl_3$)

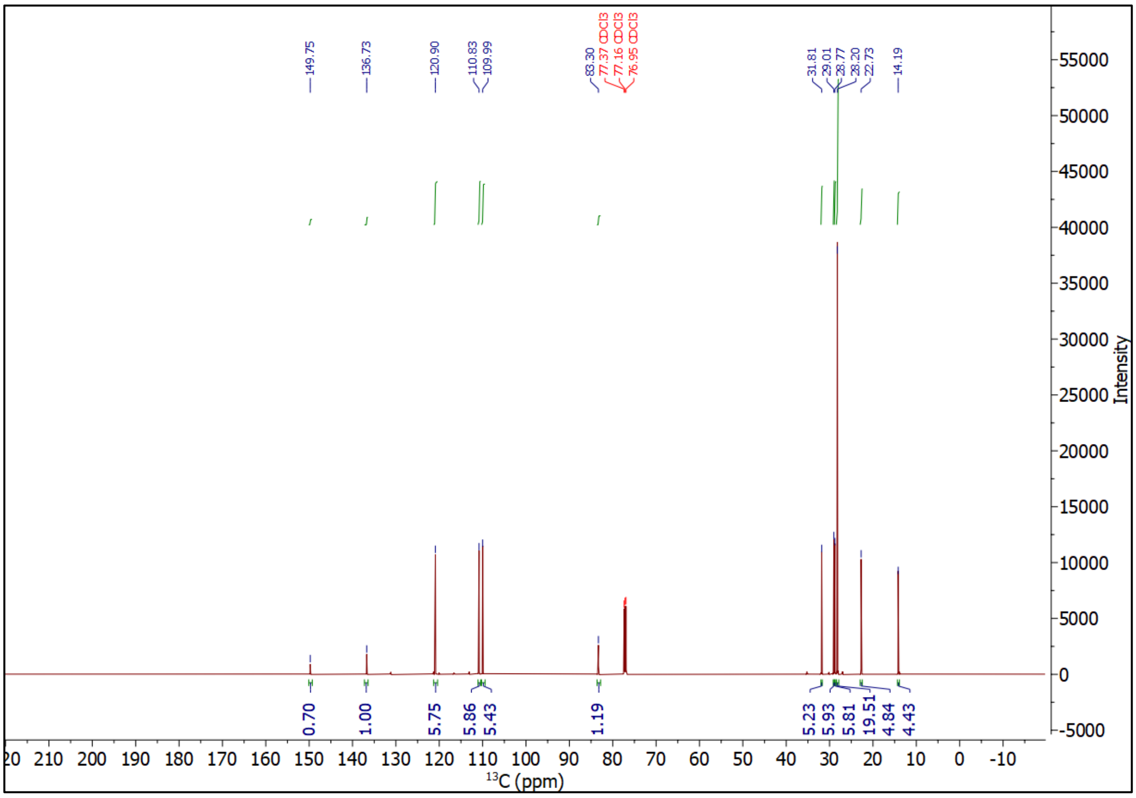

(1-(*tert*-Butoxycarbonyl)-5-pentyl-1*H*-pyrrol-2-yl)boronic acid (31)

^{1}H NMR (300 MHz, $CDCl_3$)

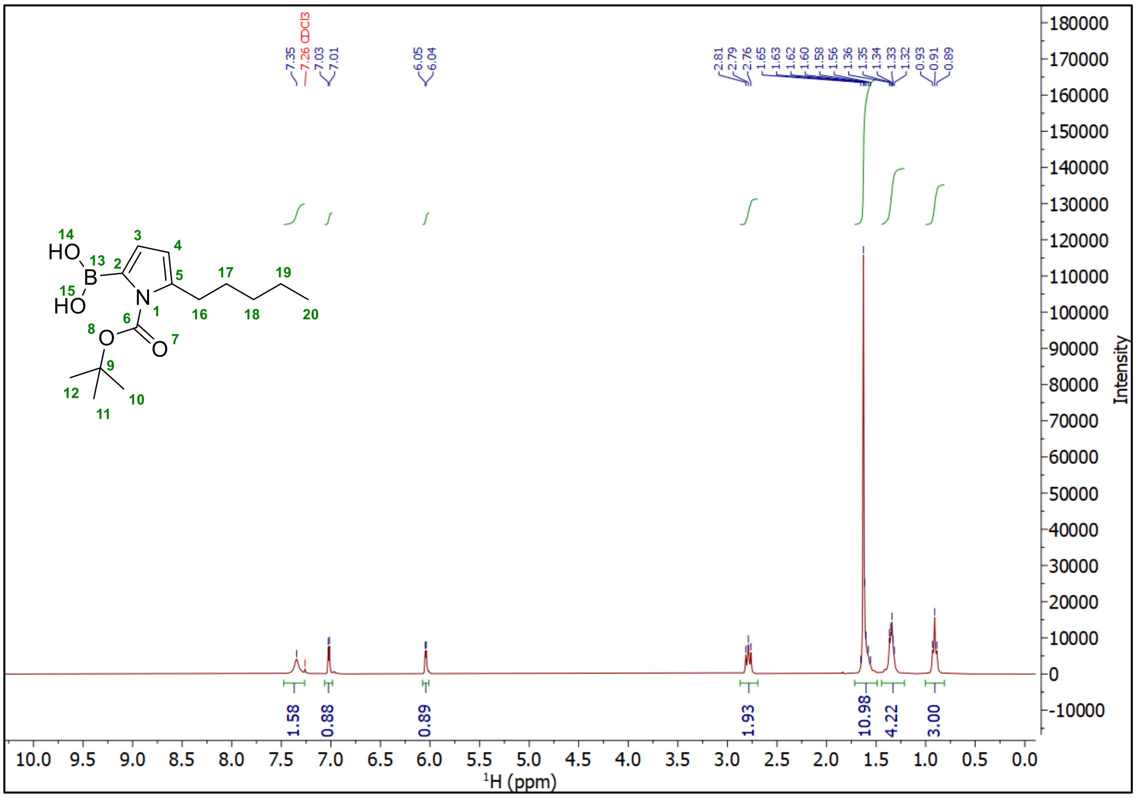

^{13}C NMR (76 MHz, $CDCl_3$)

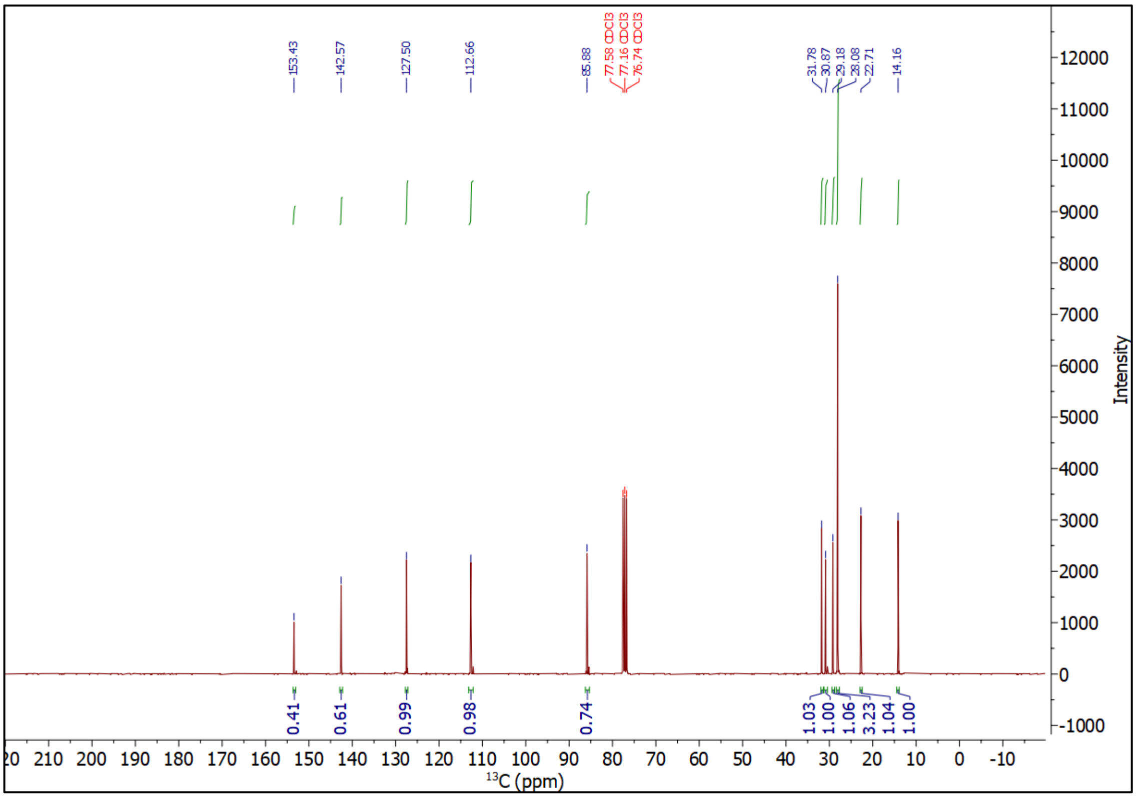

(*Z*)-4'-(4-Azidobutoxy)-5'-((5-methyl-4-propyl-1*H*-pyrrol-2-yl)methylene)-5-pentyl-1*H*,5'*H*-[2,2'-bipyrrol]-1'-ium chloride (3·HCl)

^{1}H NMR (300 MHz, $CDCl_3$)

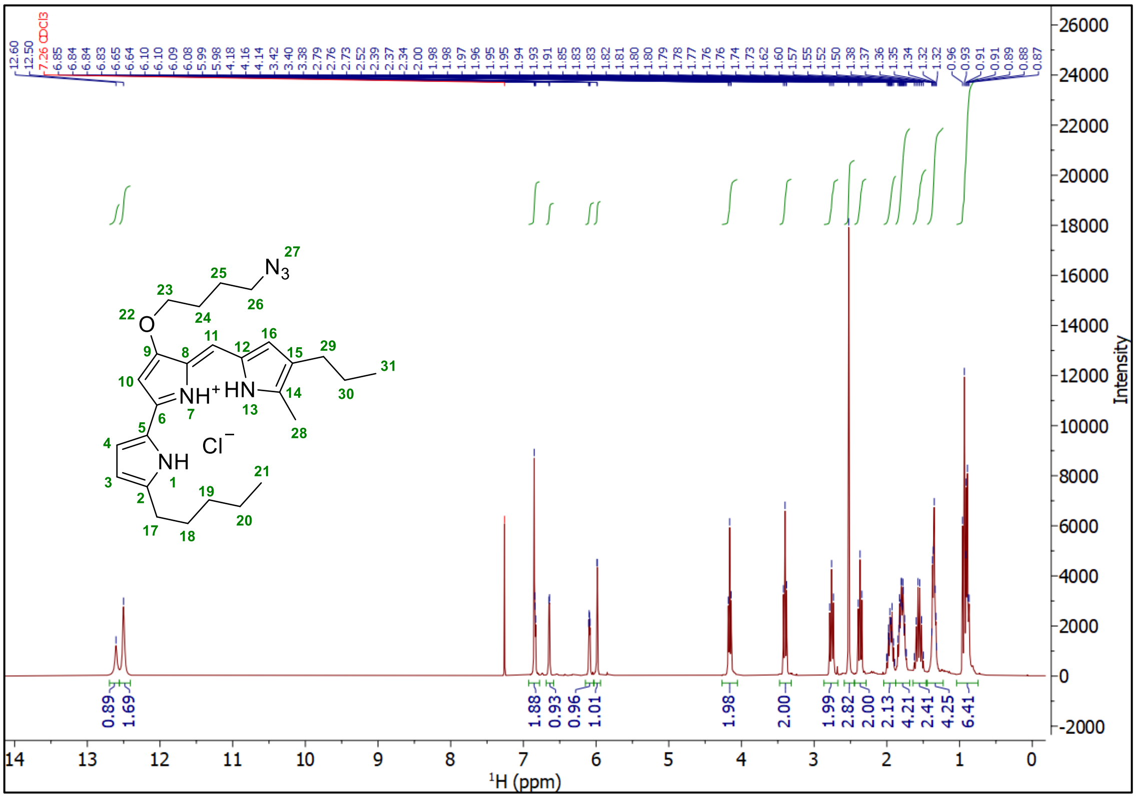

^{13}C NMR (76 MHz, $CDCl_3$)

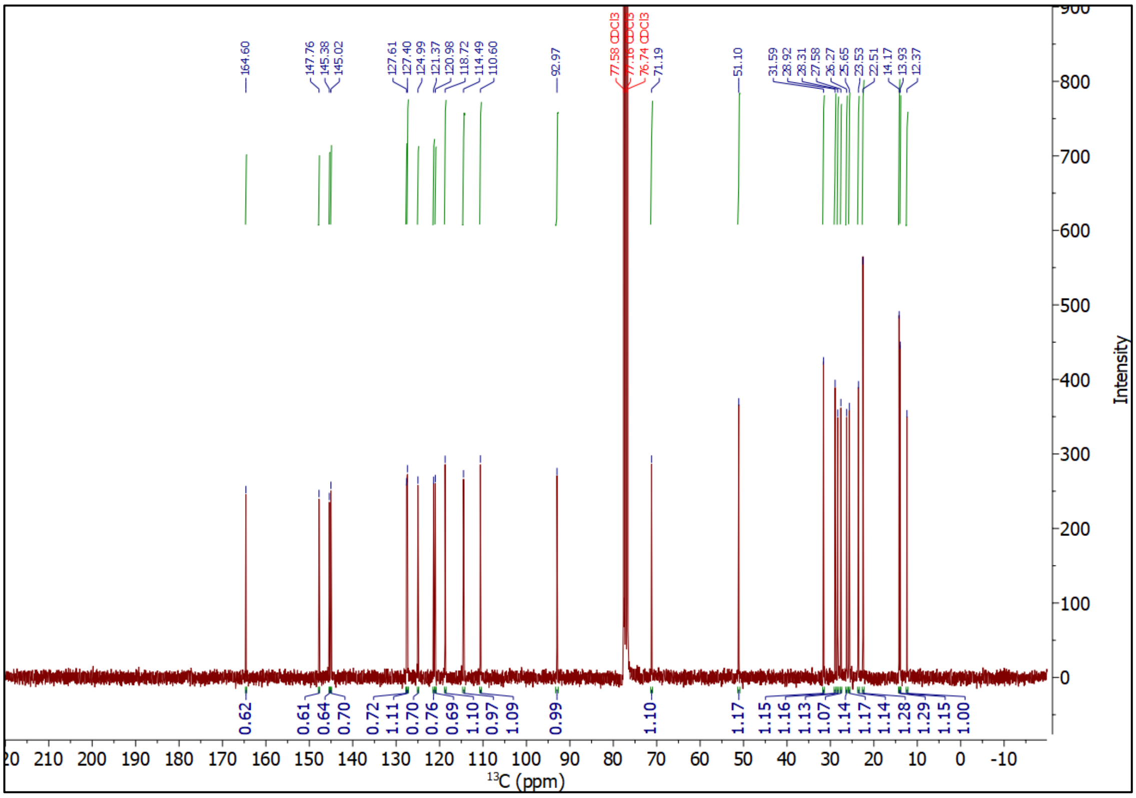

4-Methoxy-4-oxobutanoic acid (S23)

^{1}H NMR (600 MHz, $CDCl_3$)

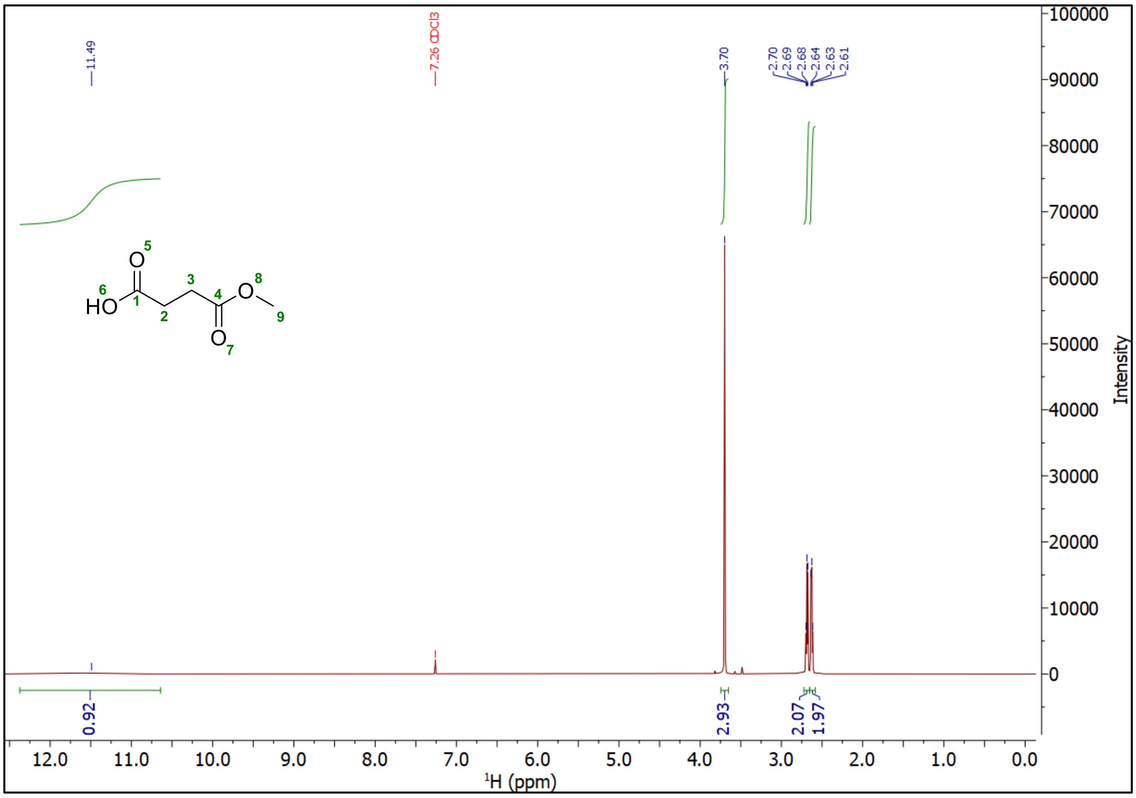

^{13}C NMR (151 MHz, $CDCl_3$)

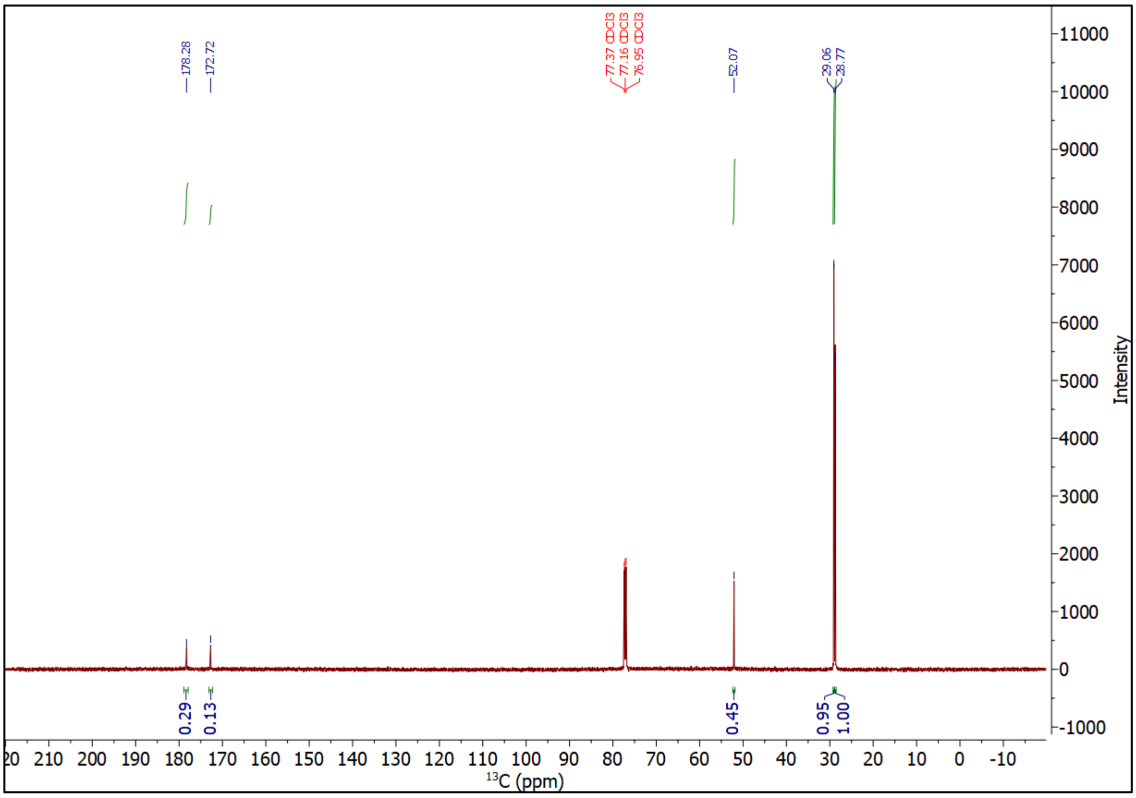

Methyl 4-chloro-4-oxobutanoate (34)

^{1}H NMR (600 MHz, $CDCl_3$)

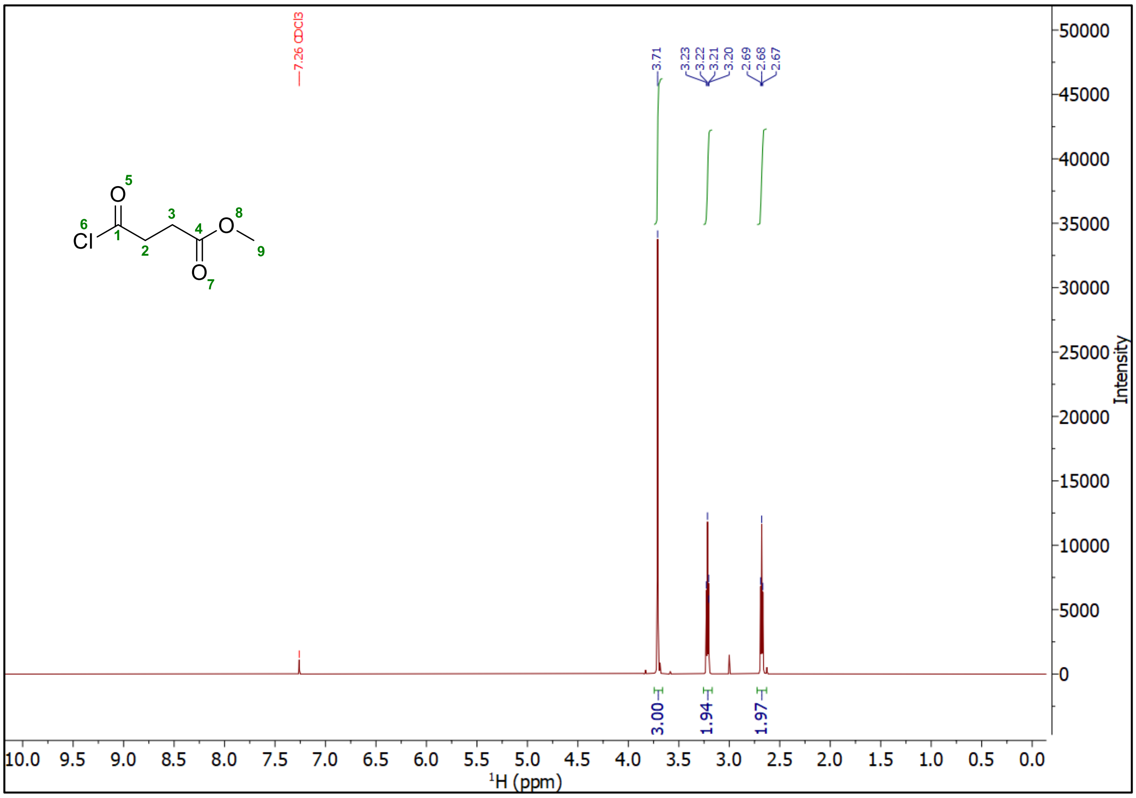

^{13}C NMR (151 MHz, $CDCl_3$)

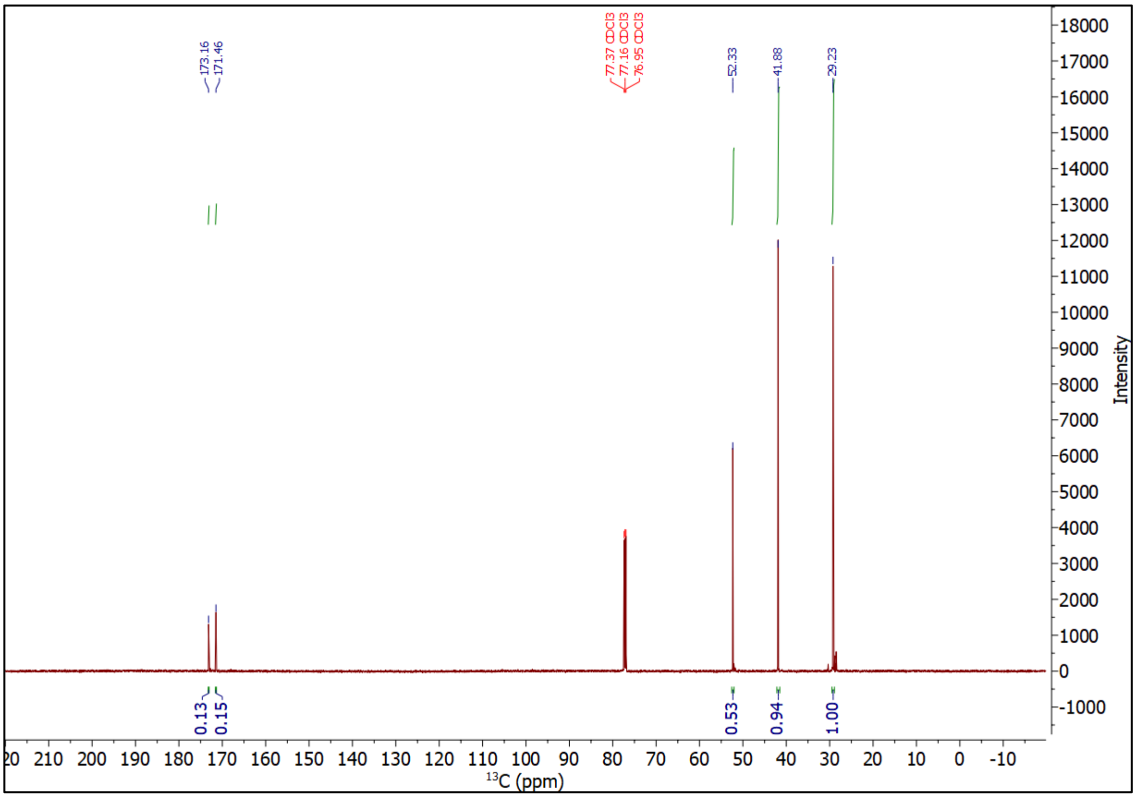

Ethyl 4-(4-methoxy-4-oxobutanoyl)-5-methyl-1*H*-pyrrole-2-carboxylate (35)

^{1}H NMR (600 MHz, CD_2Cl_2)

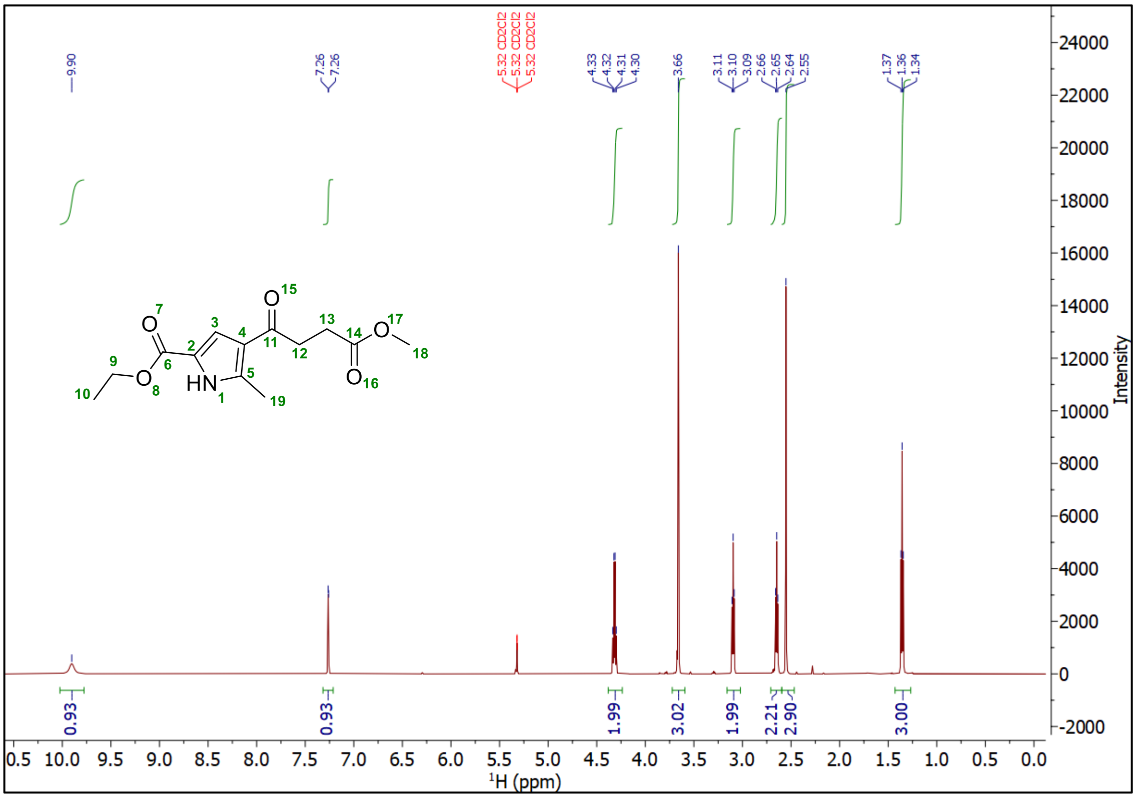

^{13}C NMR (151 MHz, CD_2Cl_2)

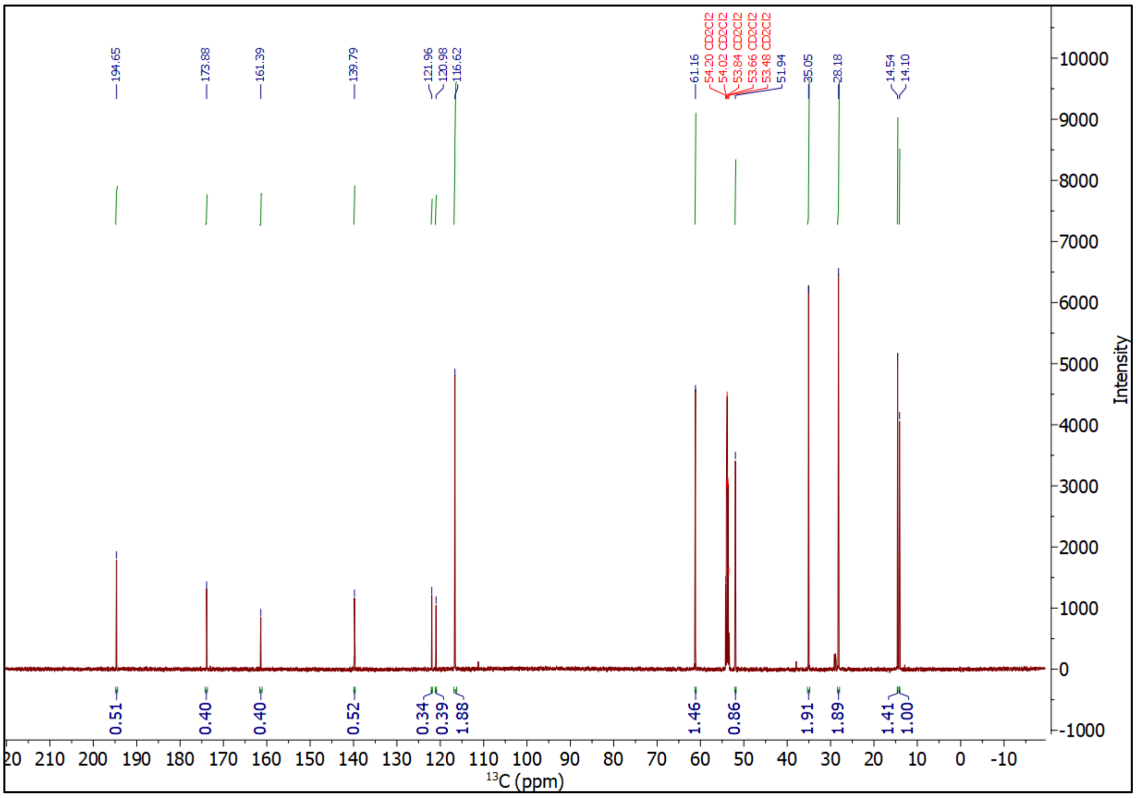

WILEY-VCH

Ethyl 4-(4-hydroxybutyl)-5-methyl-1*H*-pyrrole-2-carboxylate (36)

^{1}H NMR (600 MHz, $CDCl_3$)

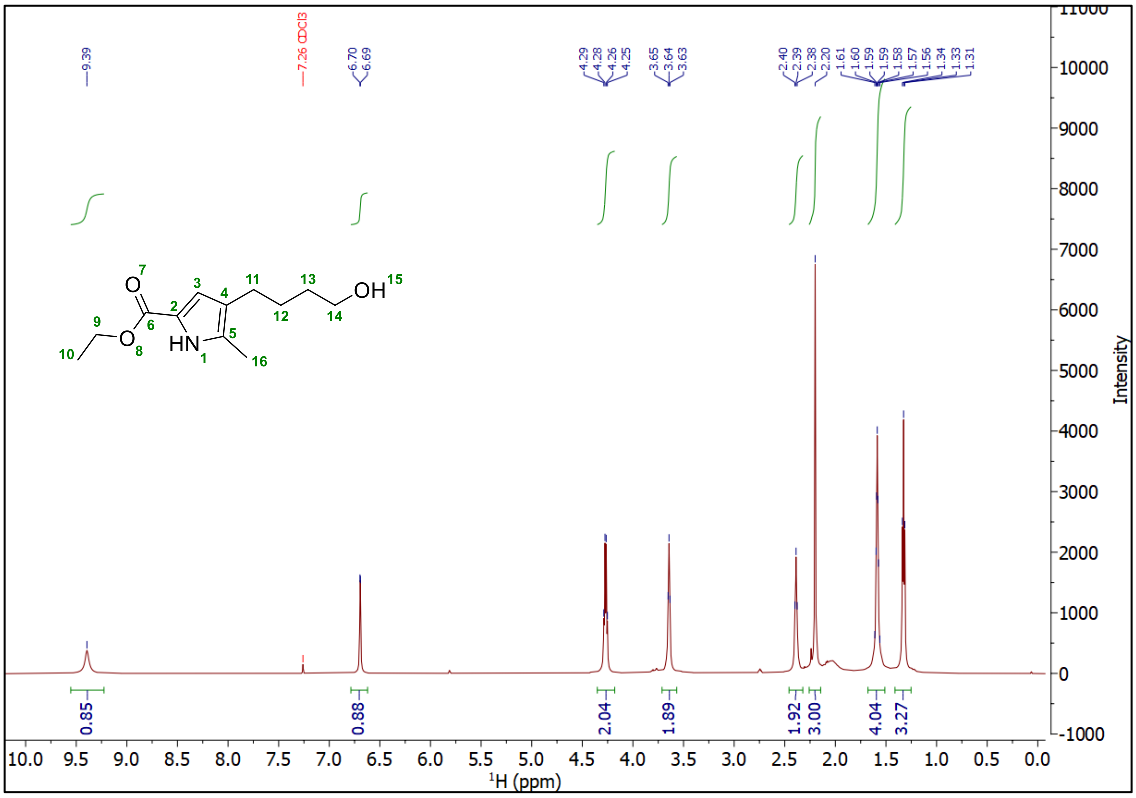

^{13}C NMR (151 MHz, $CDCl_3$)

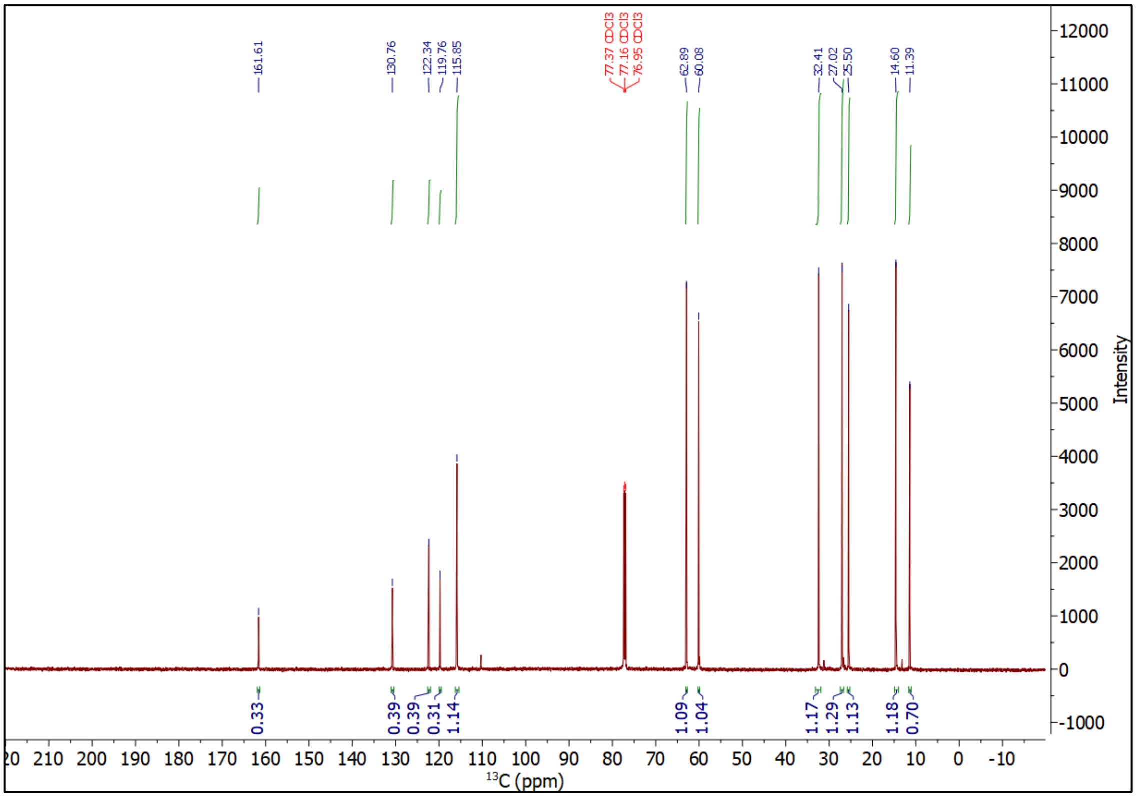

4-(2-Methyl-1*H*-pyrrol-3-yl)butan-1-ol (32) (from decarboxylation of pyrrole **36**)

^{1}H NMR (600 MHz, $CDCl_3$)

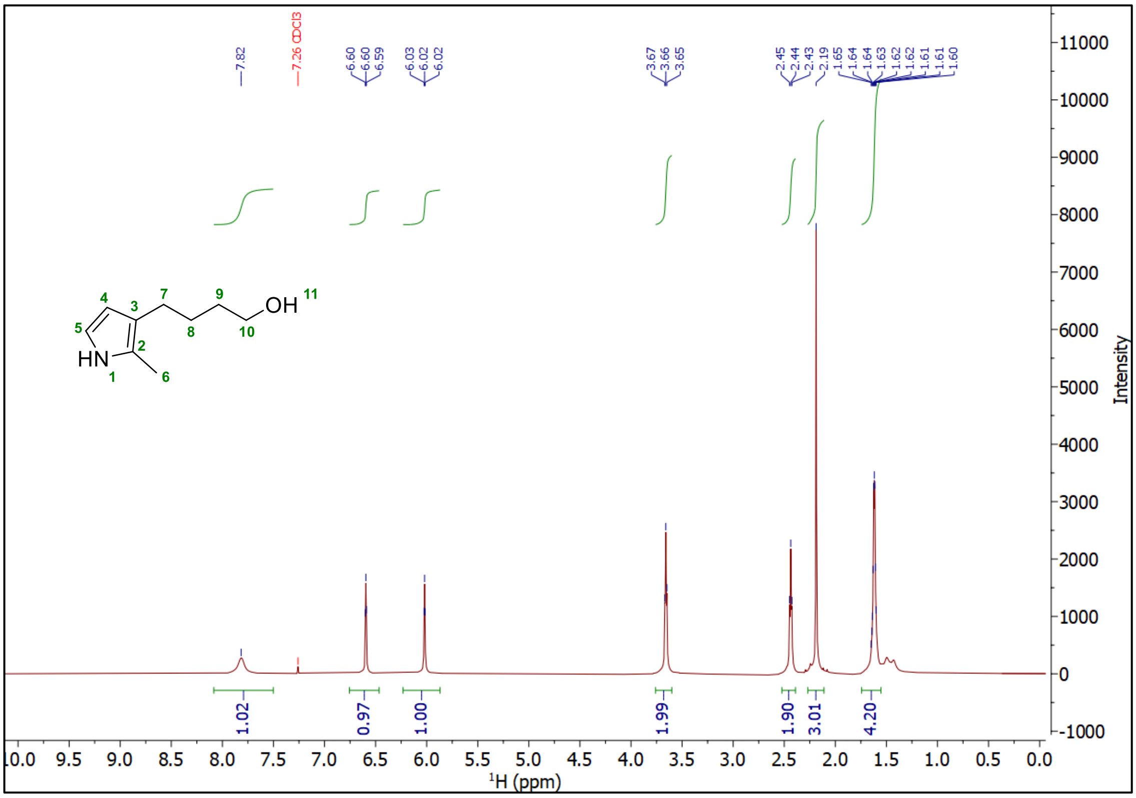

^{13}C NMR (151 MHz, $CDCl_3$)

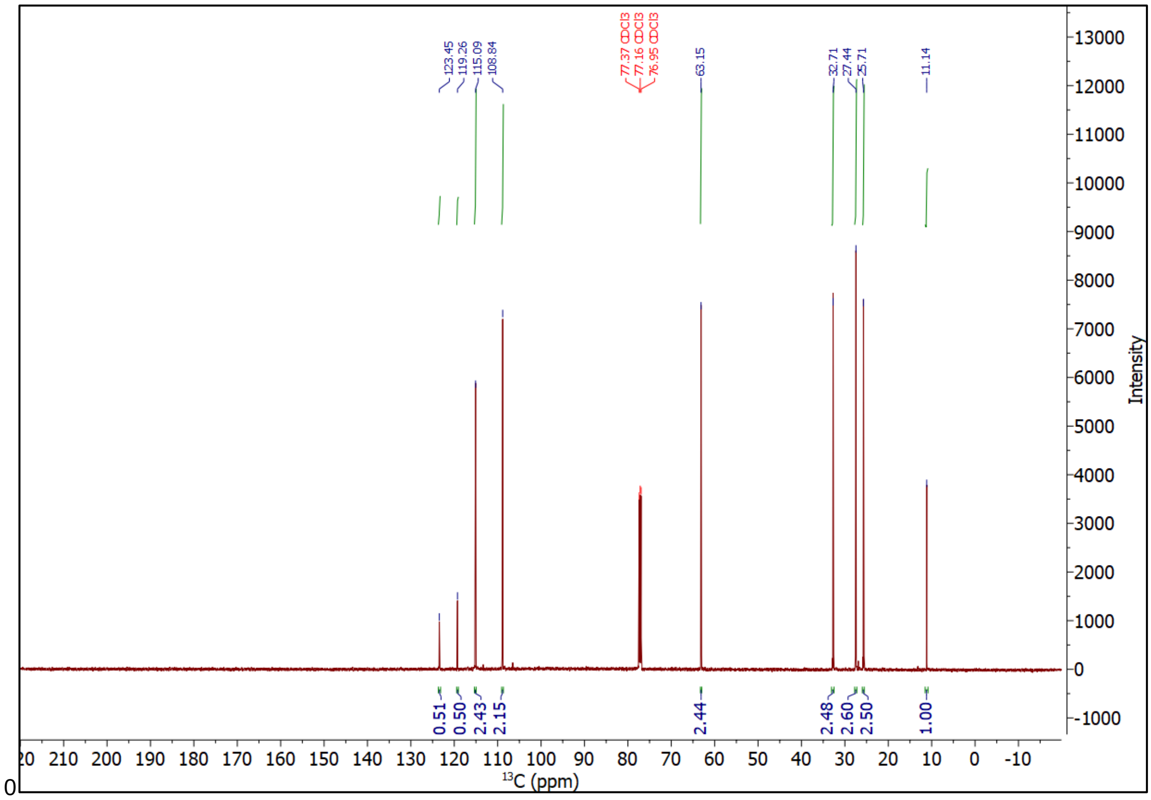

3-(4-Azidobutyl)-2-methyl-1*H*-pyrrole (38)

^{1}H NMR (600 MHz, $CDCl_3$)

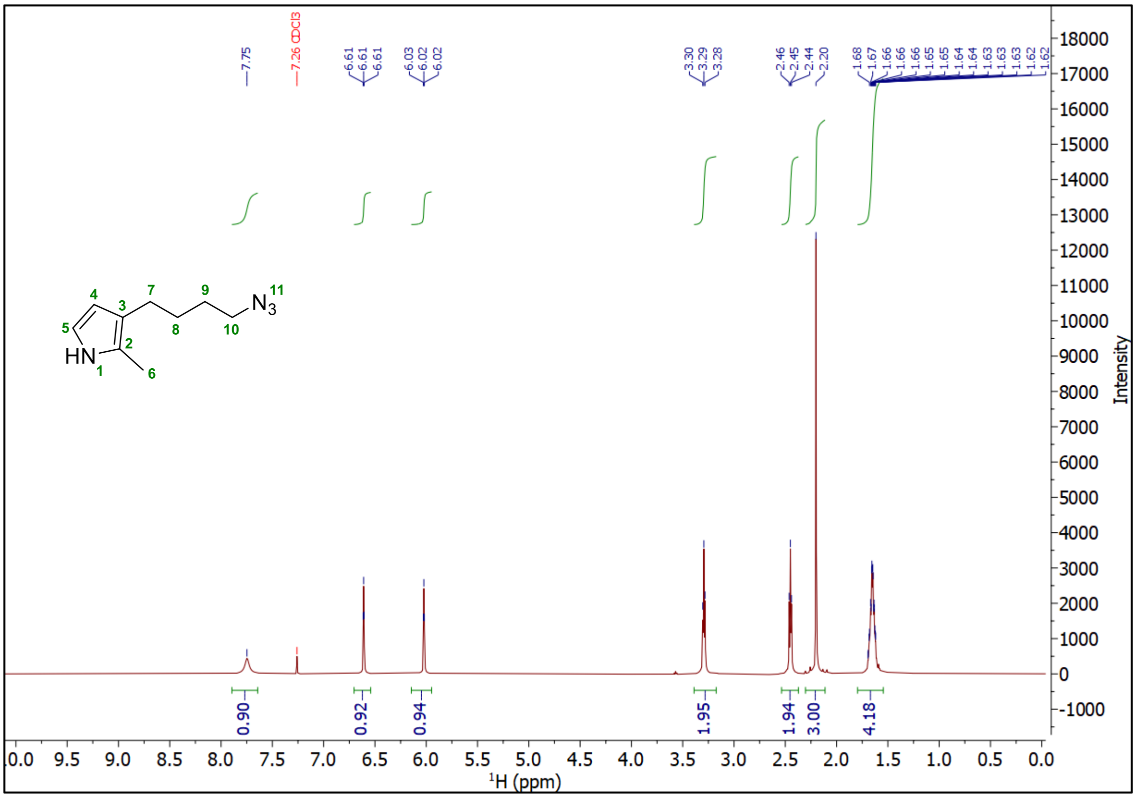

^{13}C NMR (151 MHz, $CDCl_3$)

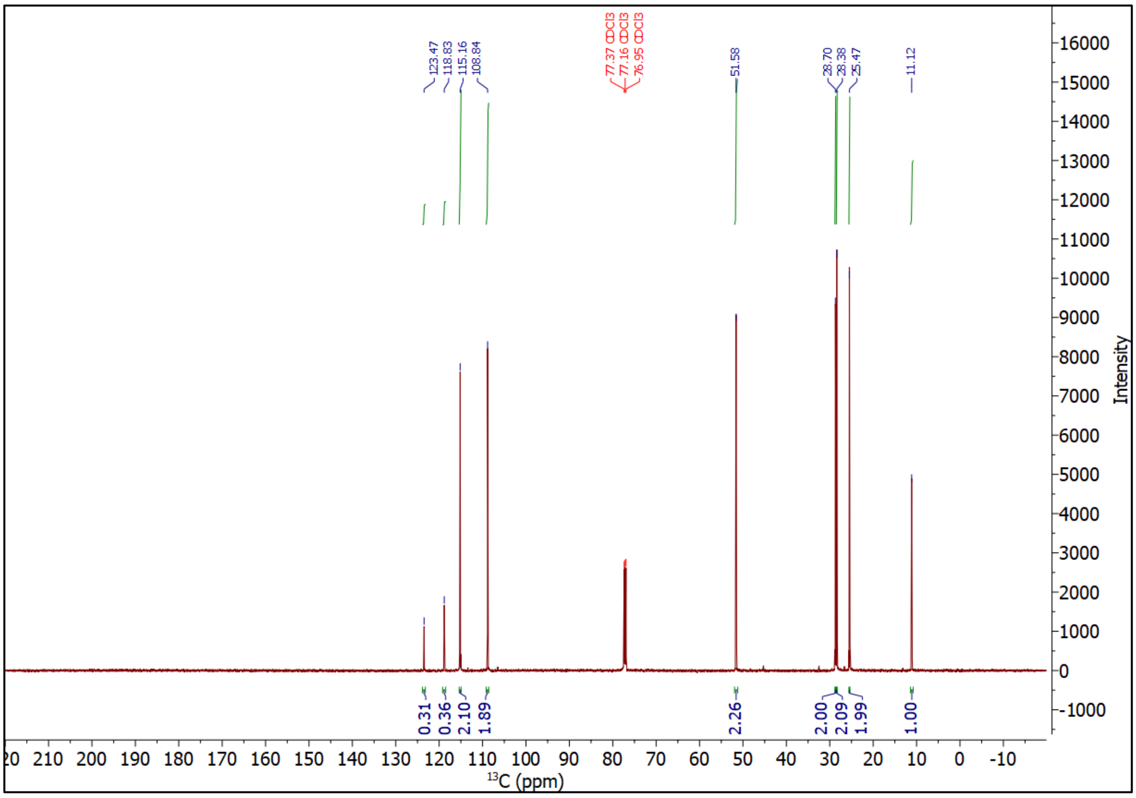

4-(4-Azidobutyl)-5-methyl-1*H*-pyrrole-2-carbaldehyde (39)

^{1}H NMR (600 MHz, $CDCl_3$)

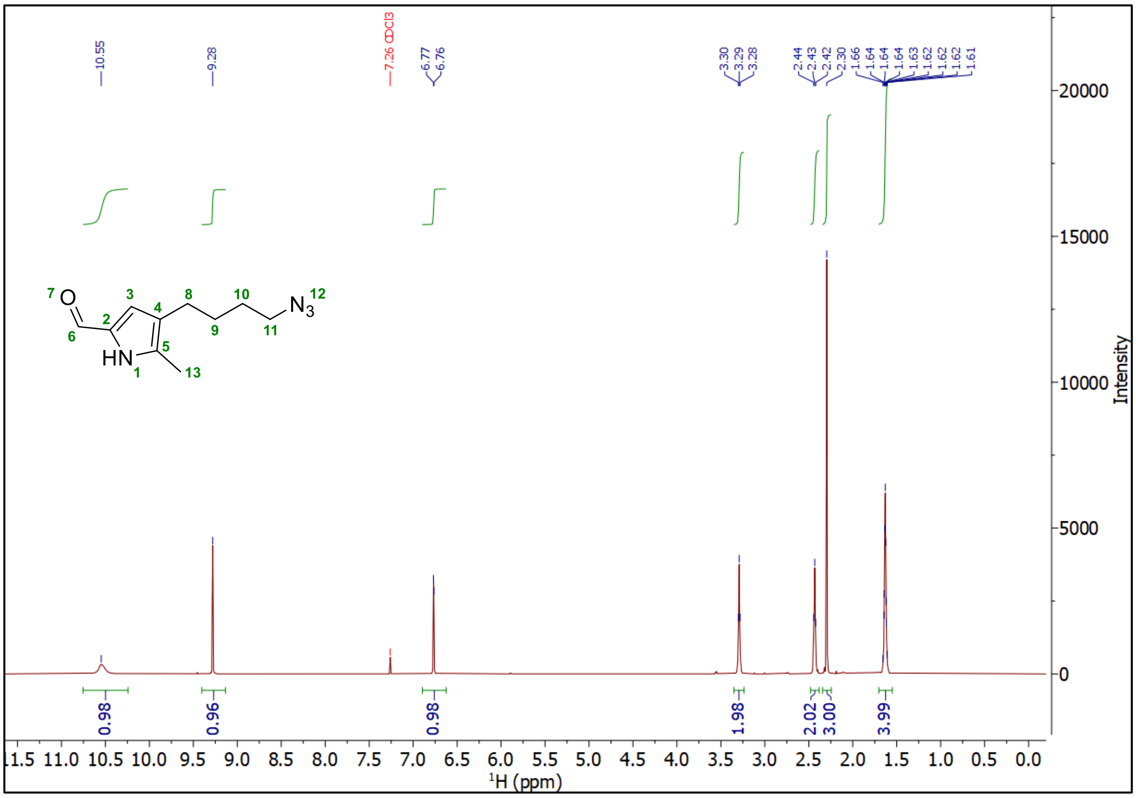

^{13}C NMR (151 MHz, $CDCl_3$)

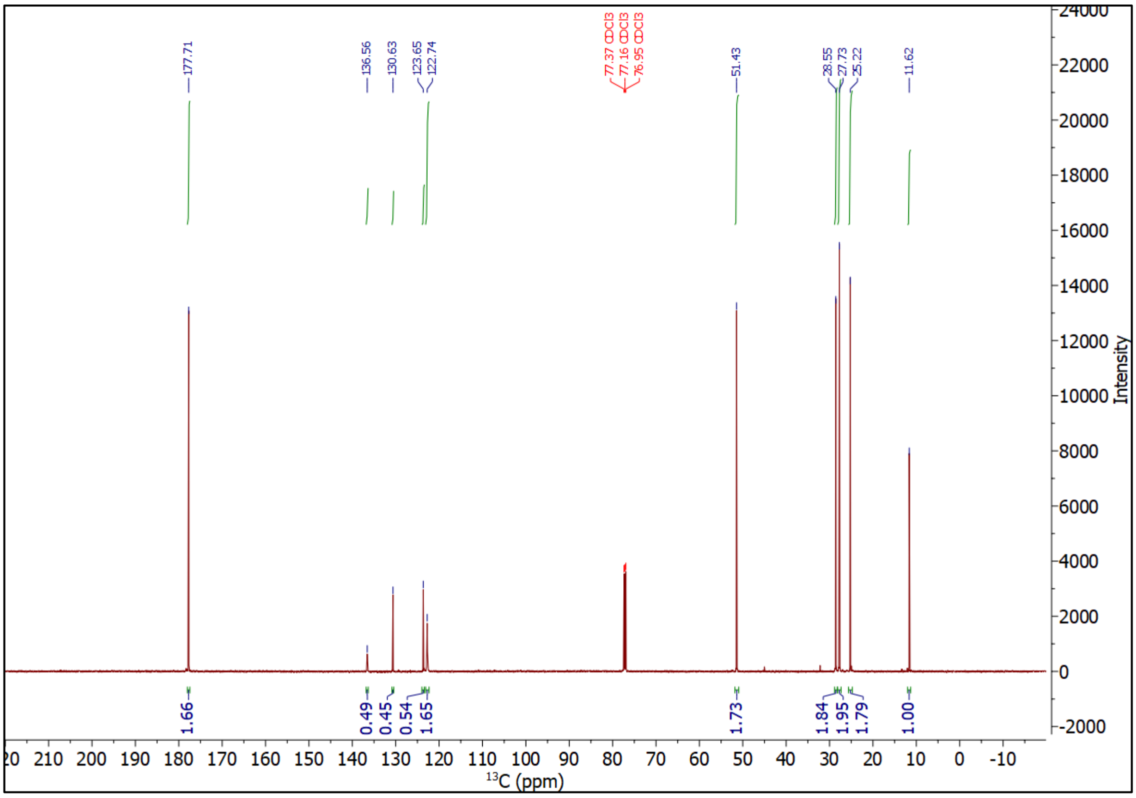

(*Z*)-5-((4-(4-Azidobutyl)-5-methyl-1*H*-pyrrol-2-yl)methylene)-4-methoxy-1,5-dihydro-2*H*-pyrrol-2-one (40)

^{1}H NMR (300 MHz, $CDCl_3$)

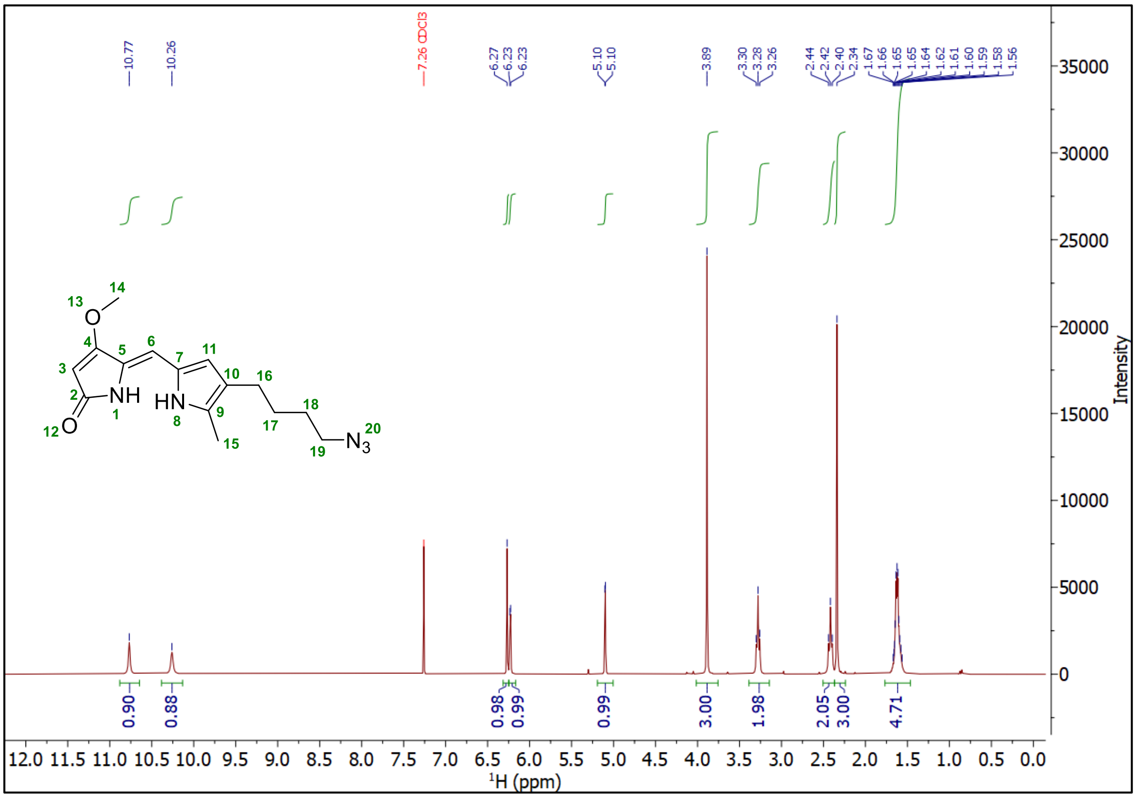

^{13}C NMR (76 MHz, $CDCl_3$)

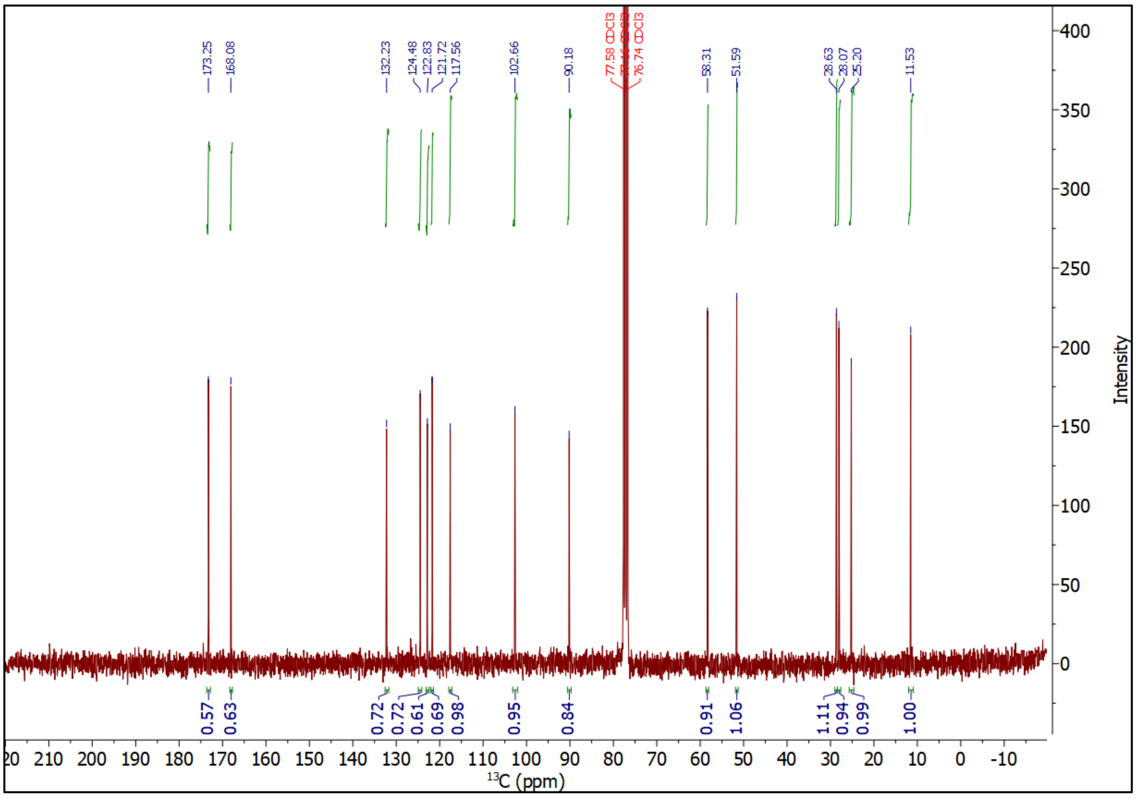

(*Z*)-5-((4-(4-Azidobutyl)-5-methyl-2*H*-pyrrol-2-ylidene)methyl)-4-methoxy-1*H*-pyrrol-2-yl trifluoromethanesulfonate (41)

^{1}H NMR (300 MHz, $CDCl_3$)

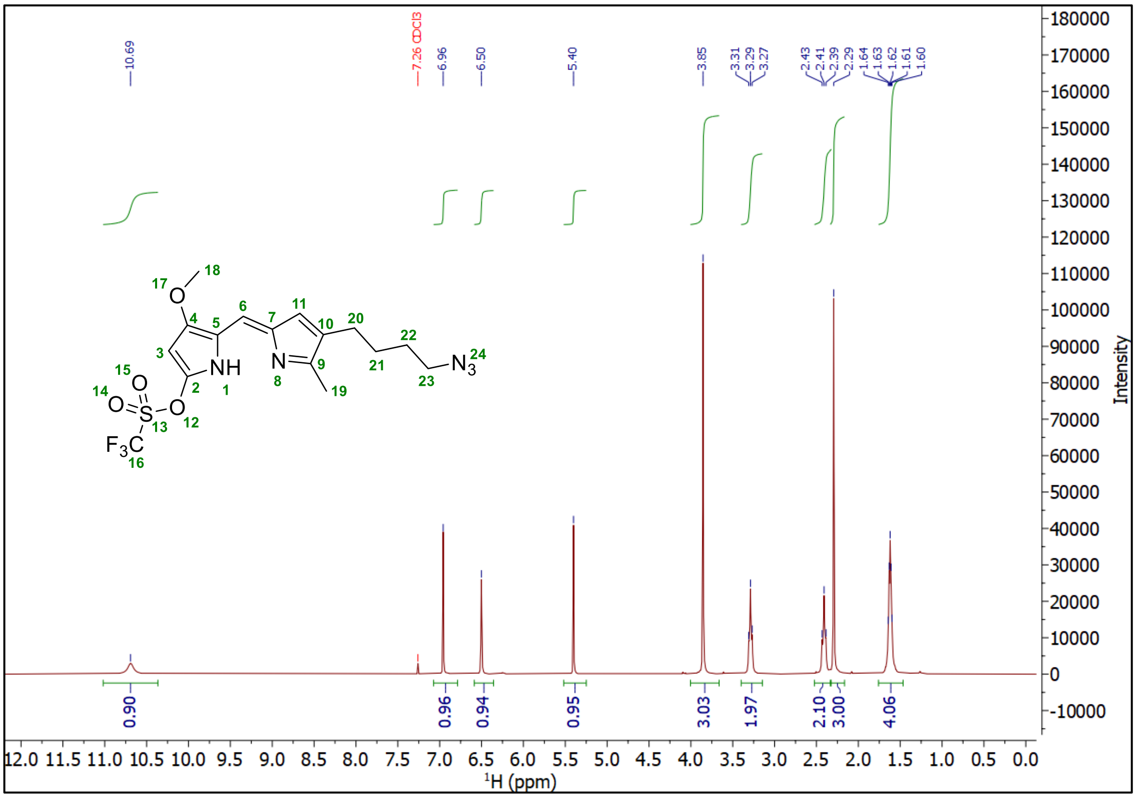

^{13}C NMR (76 MHz, $CDCl_3$)

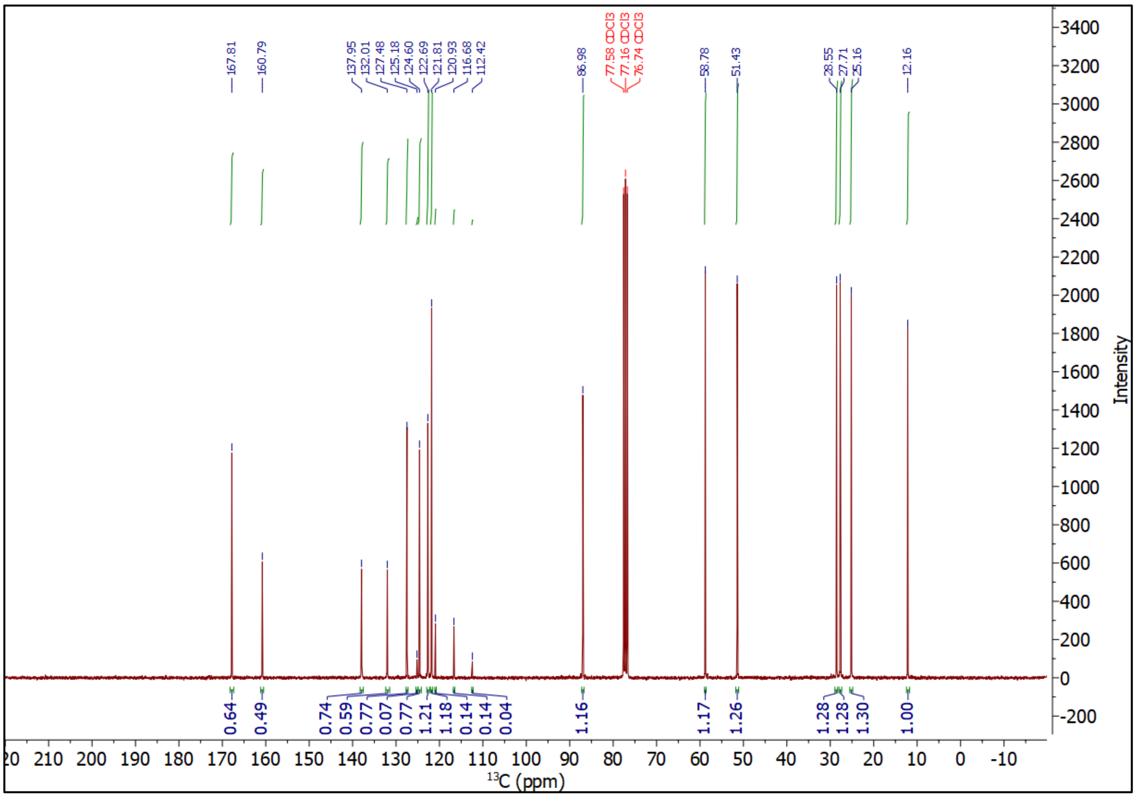

(*Z*)-5'-((4-(4-Azidobutyl)-5-methyl-1*H*-pyrrol-2-yl)methylene)-4'-methoxy-5-pentyl-1*H*,5'*H*-[2,2'-bipyrrol]-1'-ium chloride (4·HCl)

^{1}H NMR (300 MHz, $CDCl_3$)

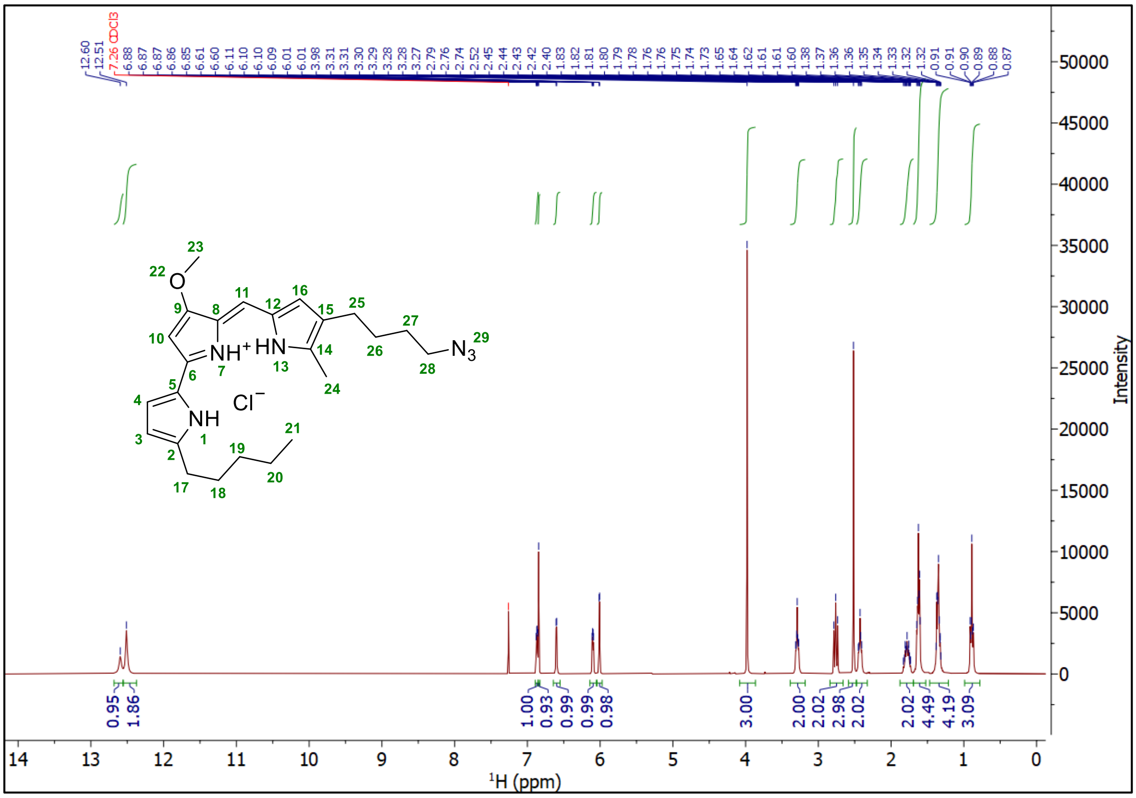

^{13}C NMR (76 MHz, $CDCl_3$)

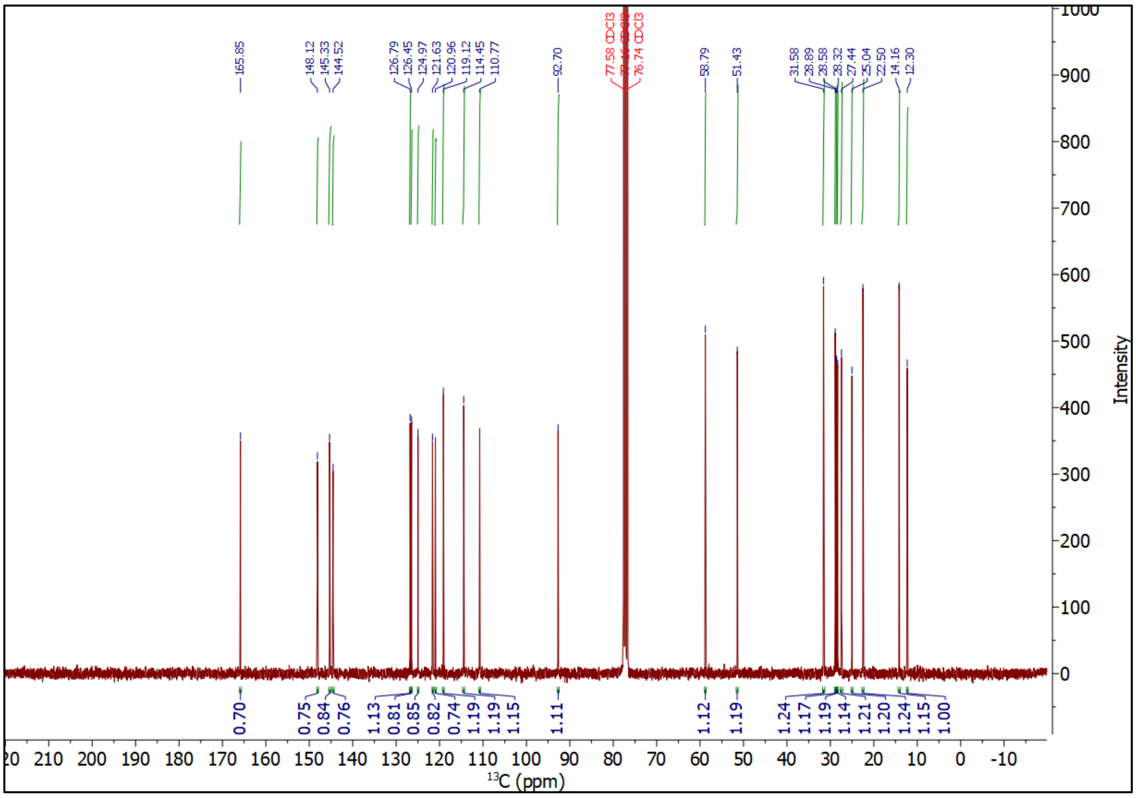

Methyl 2,5-dioxo-2,5-dihydro-1*H*-pyrrole-1-carboxylate (S25)

^{1}H NMR (600 MHz, $CDCl_3$)

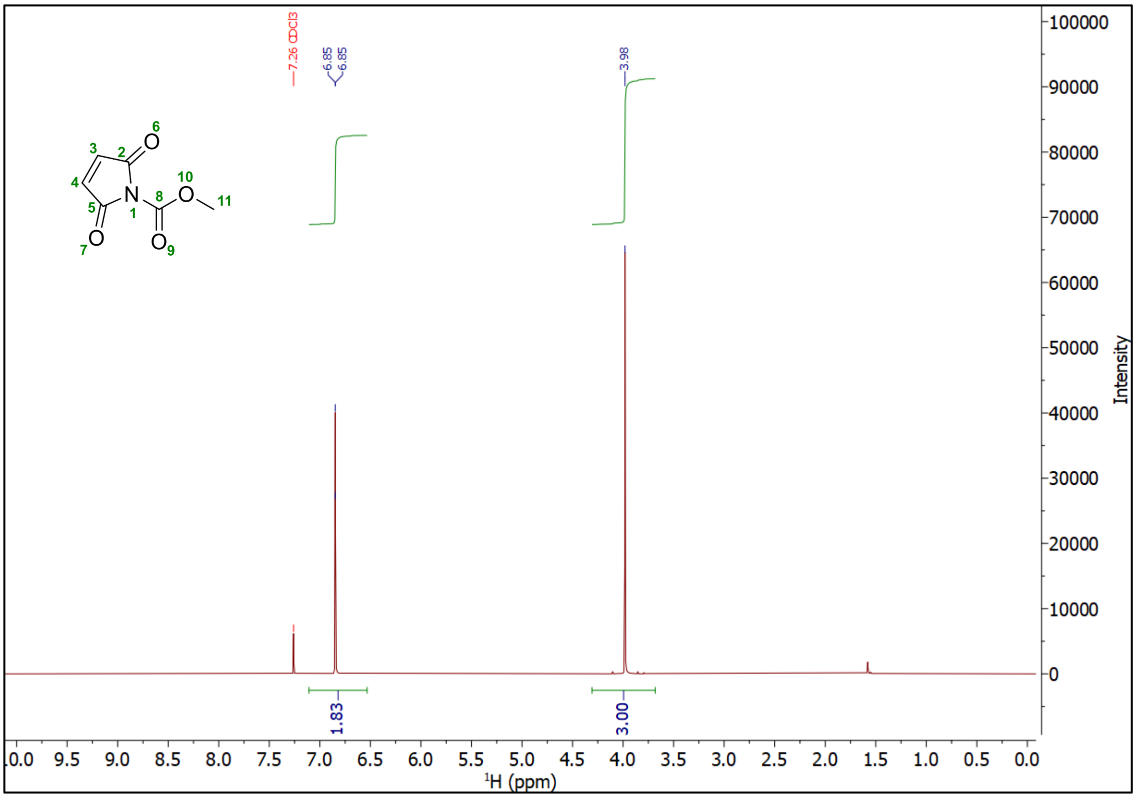

^{13}C NMR (151 MHz, $CDCl_3$)

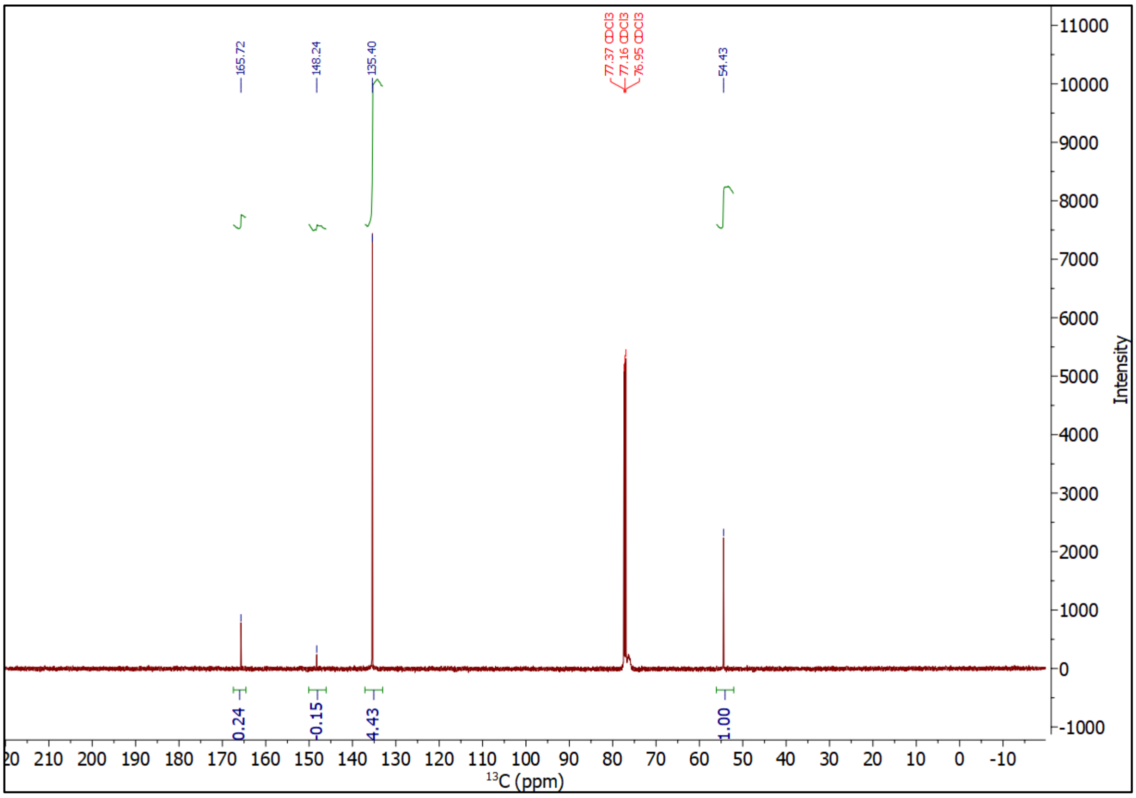

***tert*-Butyl (2-(2,5-dioxo-2,5-dihydro-1*H*-pyrrol-1-yl)ethyl)carbamate (S27)**

^{1}H NMR (600 MHz, $CDCl_3$)

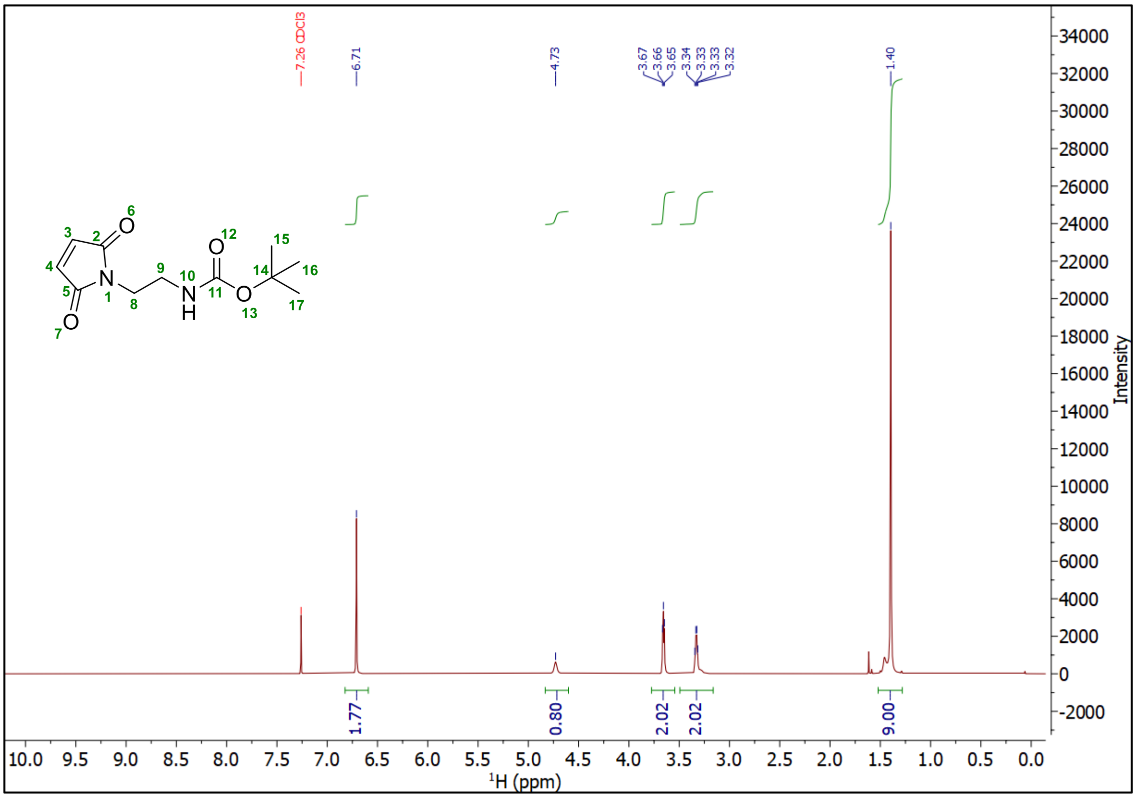

^{13}C NMR (151 MHz, $CDCl_3$)

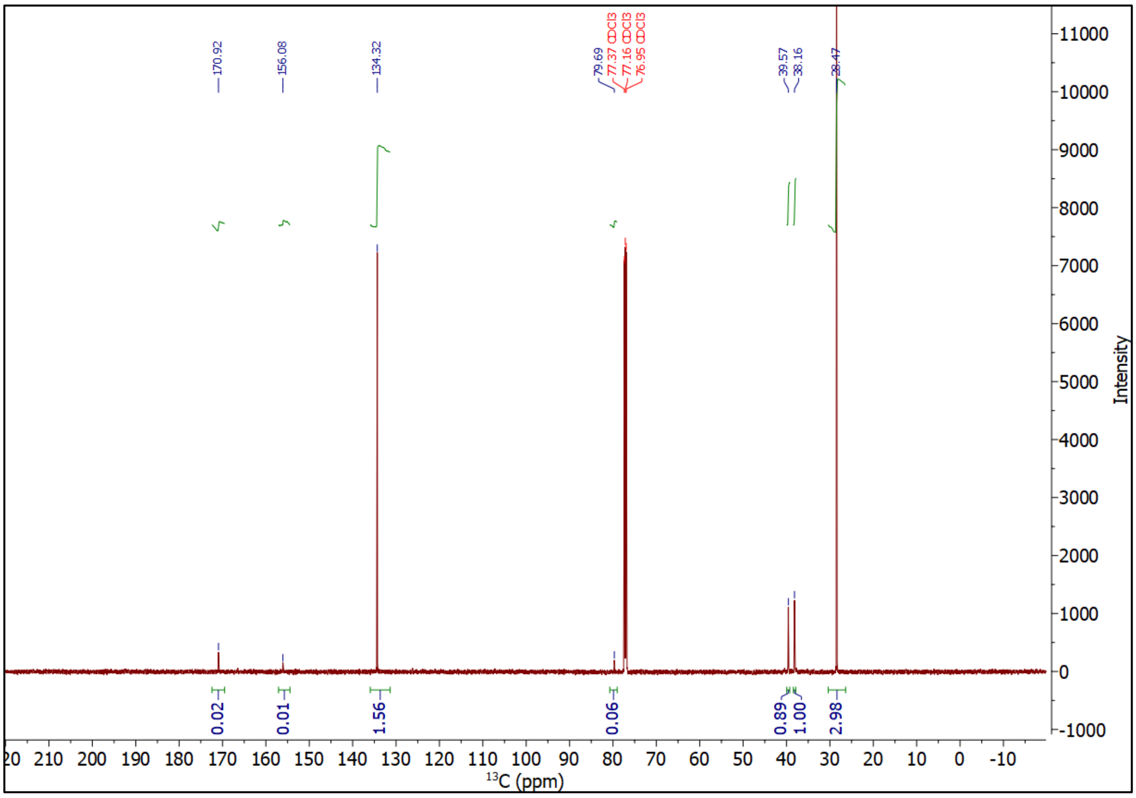

2-(2,5-Dioxo-2,5-dihydro-1*H*-pyrrol-1-yl)ethan-1-aminium 2,2,2-trifluoroacetate (S28)

^{1}H NMR (600 MHz, D_2O)

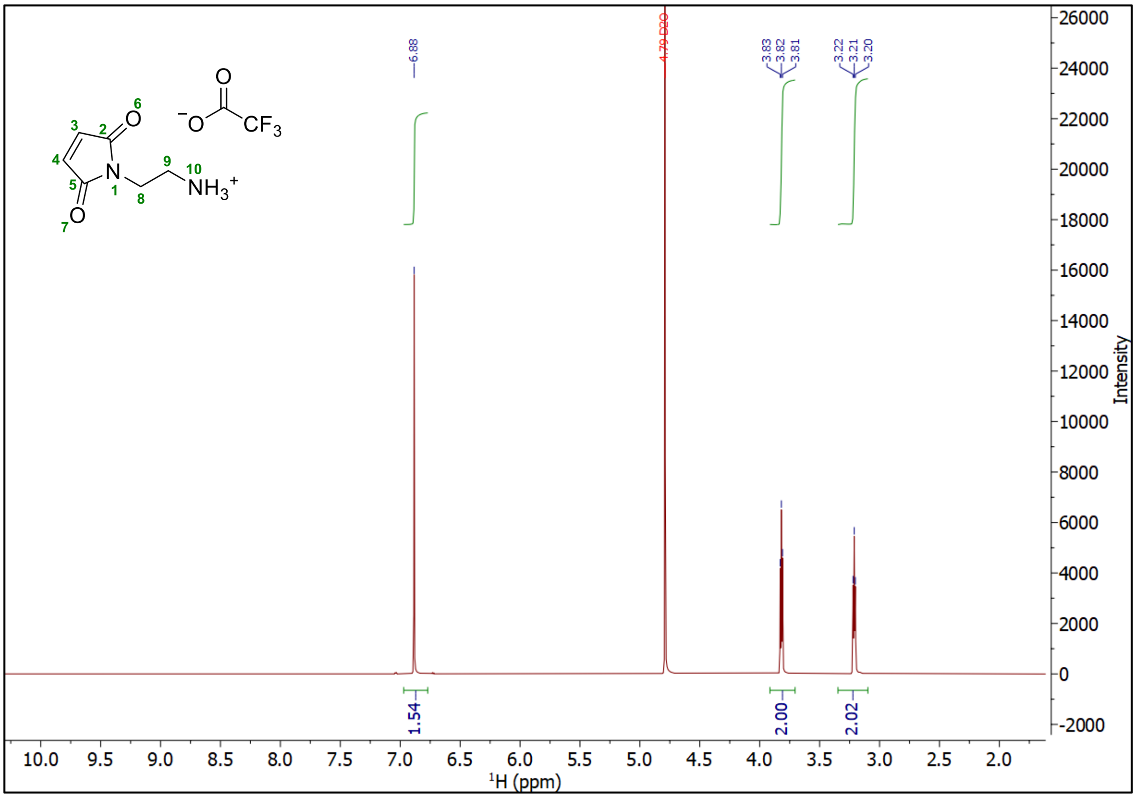

^{13}C NMR (151 MHz, D_2O)

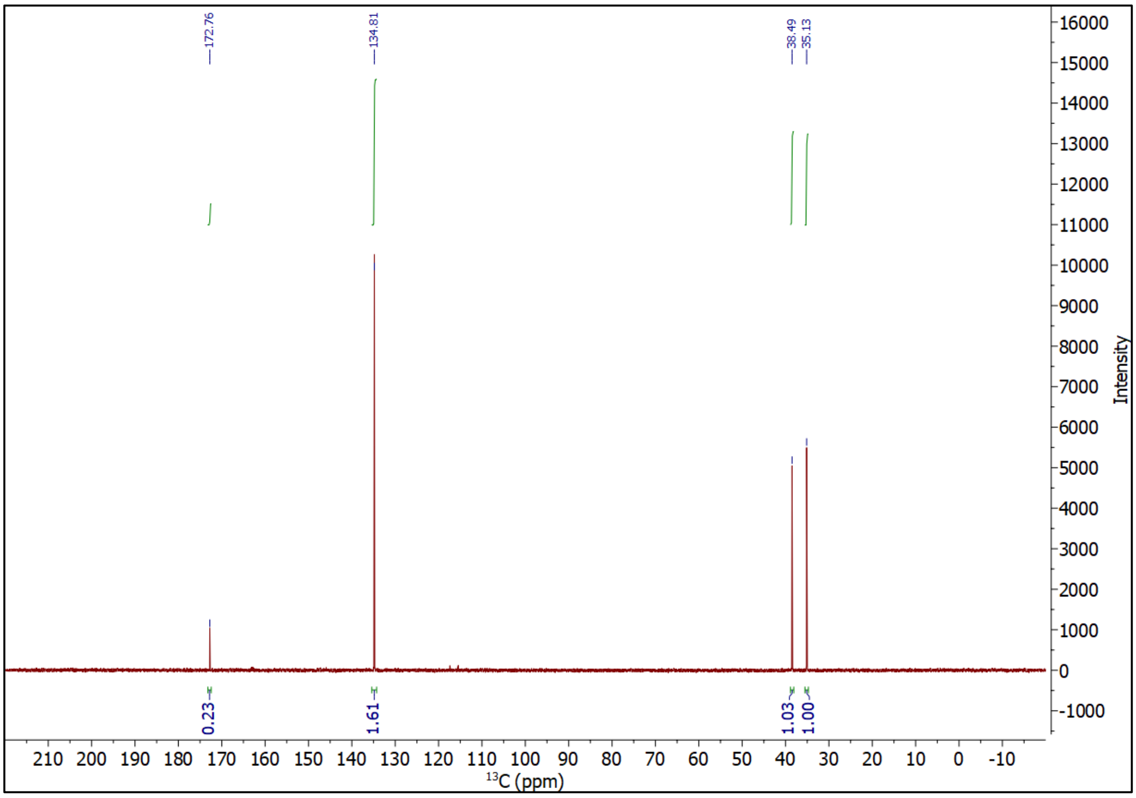

_N_-(2-(2,5-Dioxo-2,5-dihydro-1_H_-pyrrol-1-yl)ethyl)hex-5-ynamide (42)

^{1}H NMR (600 MHz, $CDCl_3$)

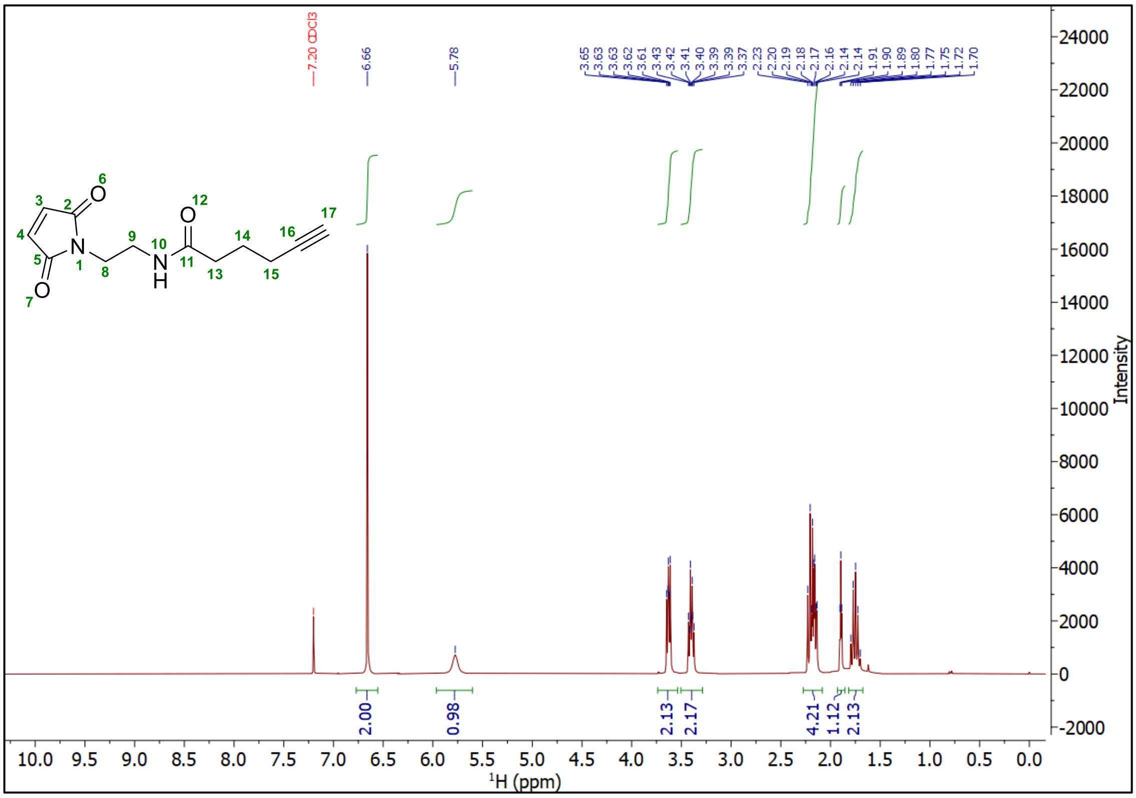

^{13}C NMR (151 MHz, $CDCl_3$)

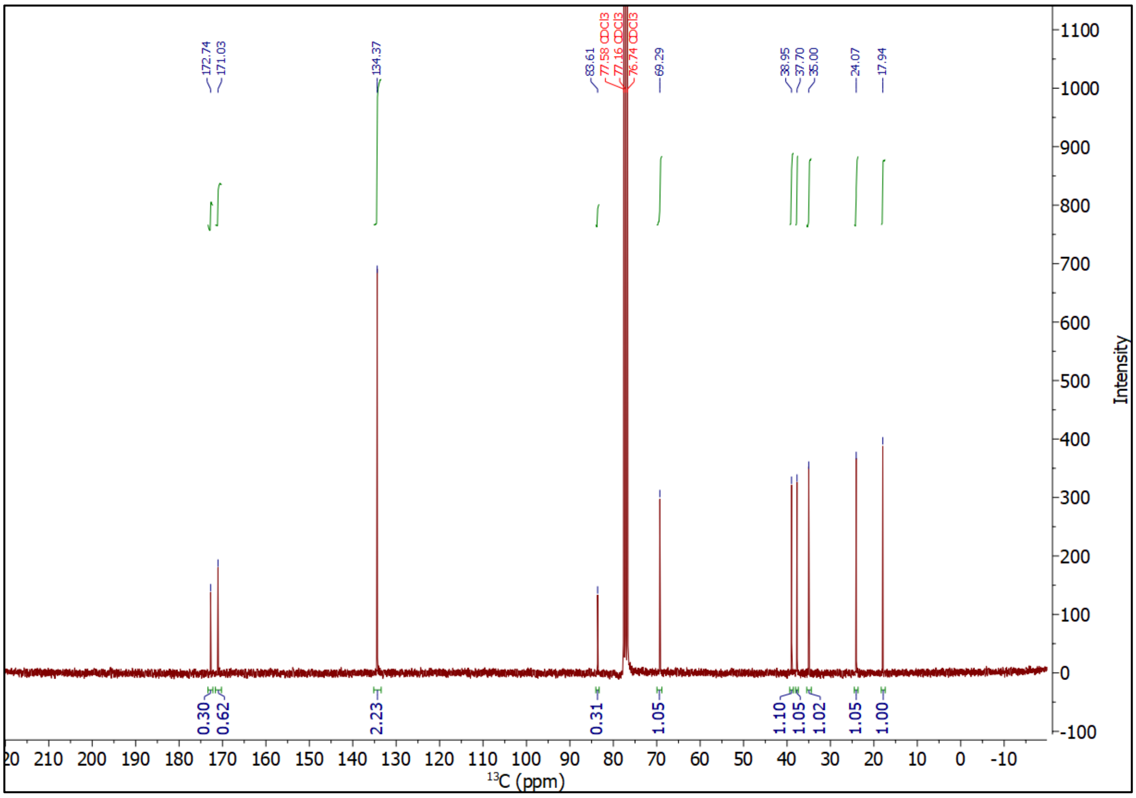

Prodigiosin A-Ring Azide Zinc-Complex (43)

^{1}H NMR (300 MHz, $CDCl_3$)

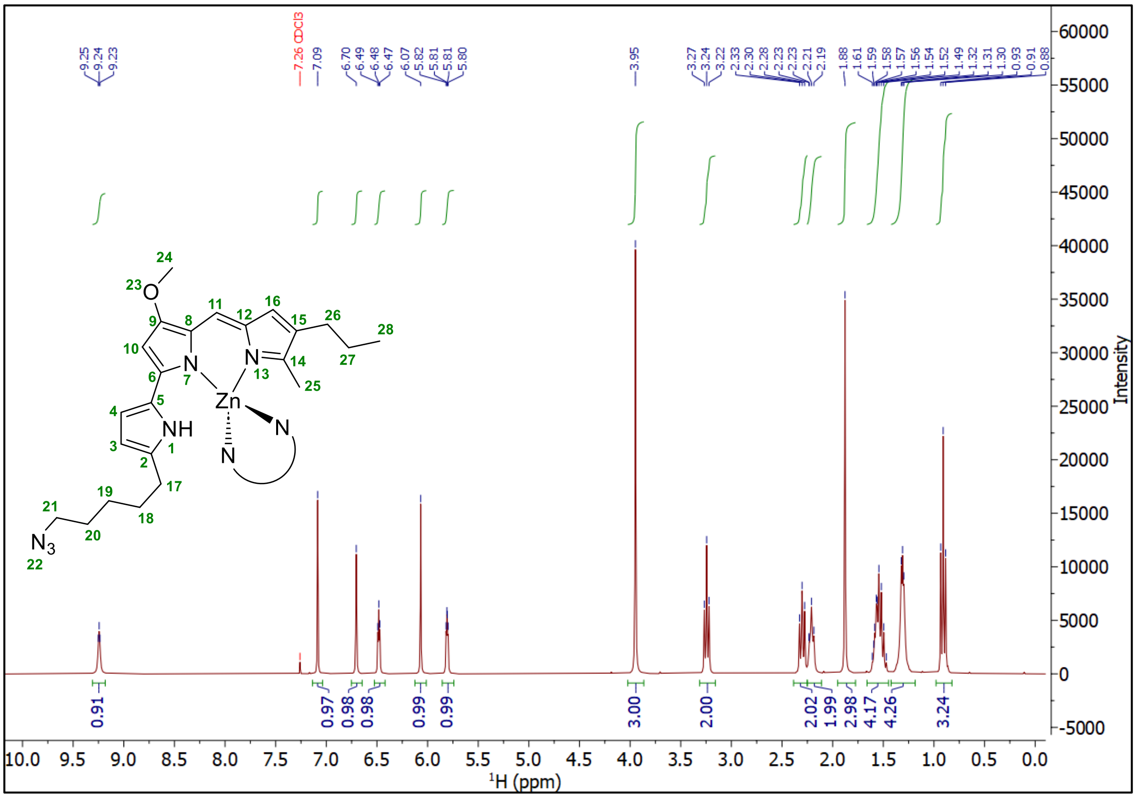

^{13}C NMR (76 MHz, $CDCl_3$)

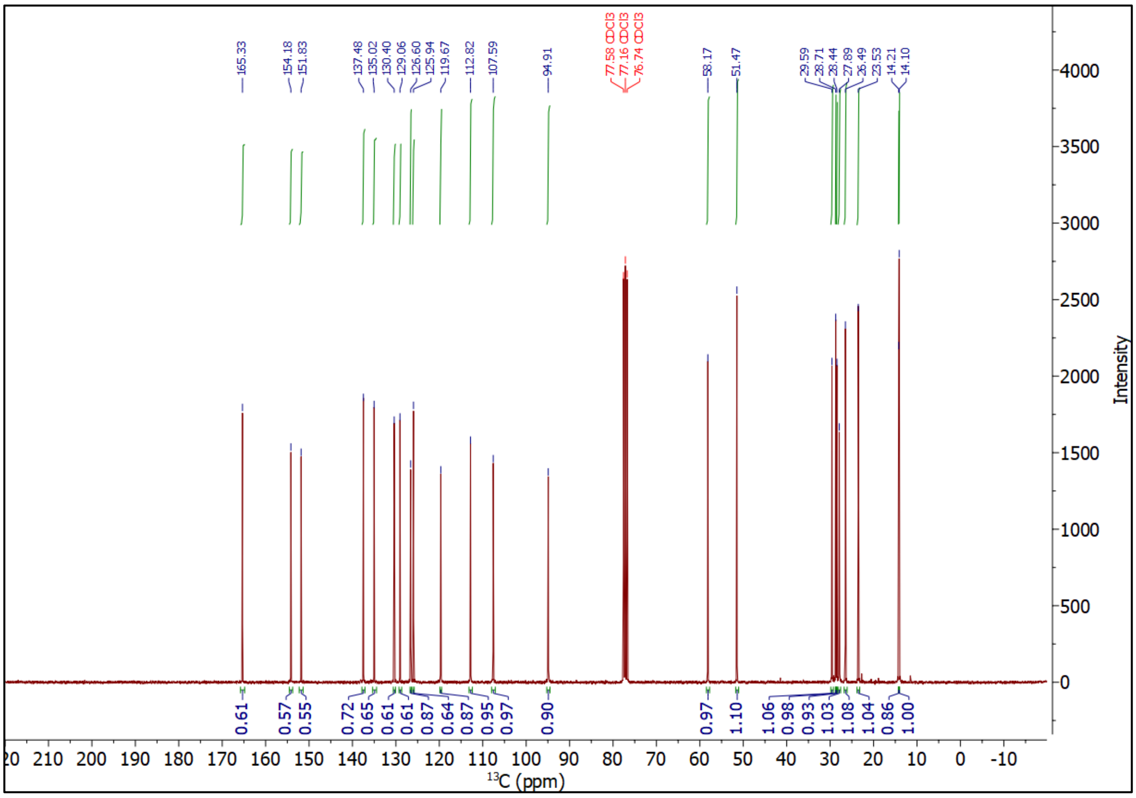

Prodigiosin B-Ring Azide Zinc-Complex (44)

^{1}H NMR (300 MHz, $CDCl_3$)

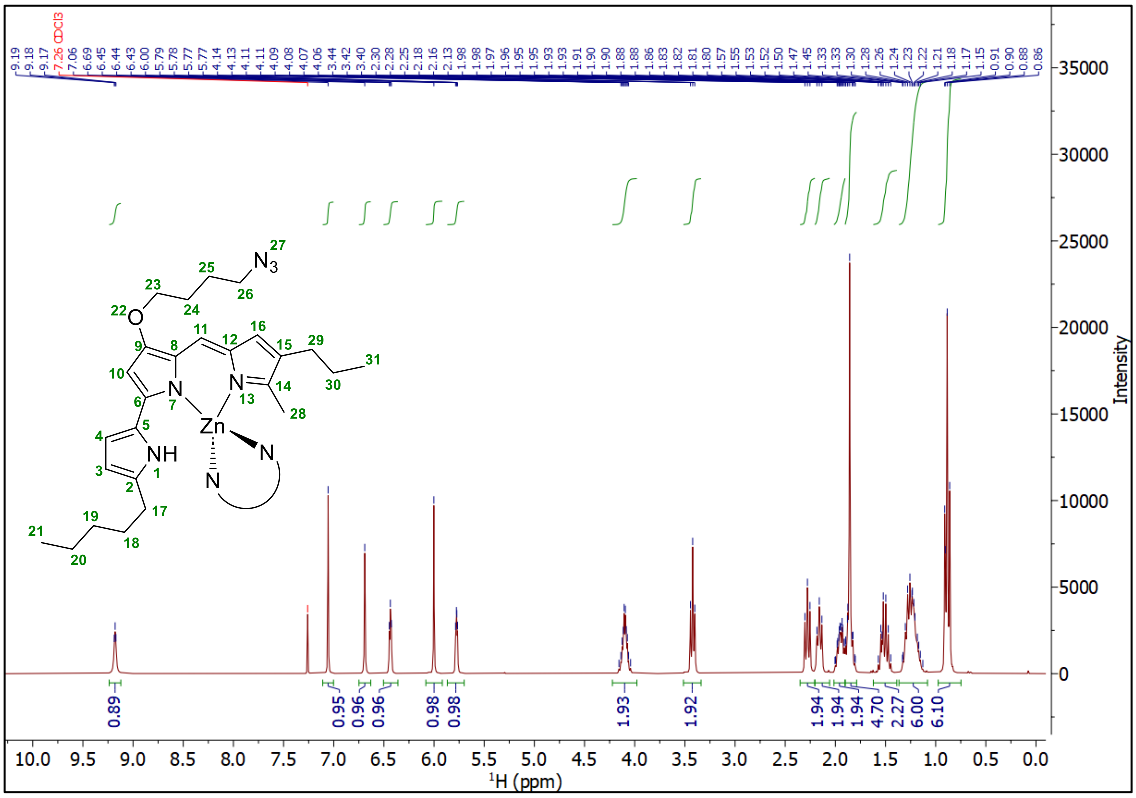

^{13}C NMR (76 MHz, $CDCl_3$)

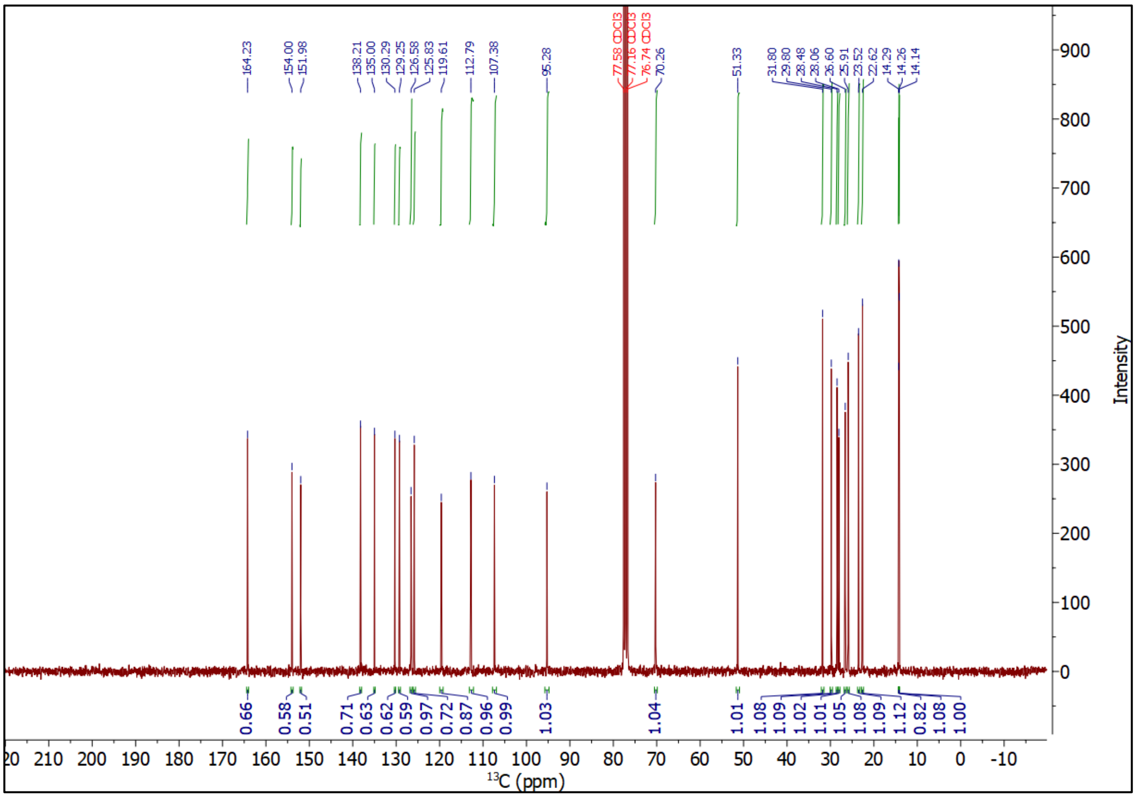

Prodigiosin C-Ring Azide Zinc-Complex (45)

^{1}H NMR (300 MHz, $CDCl_3$)

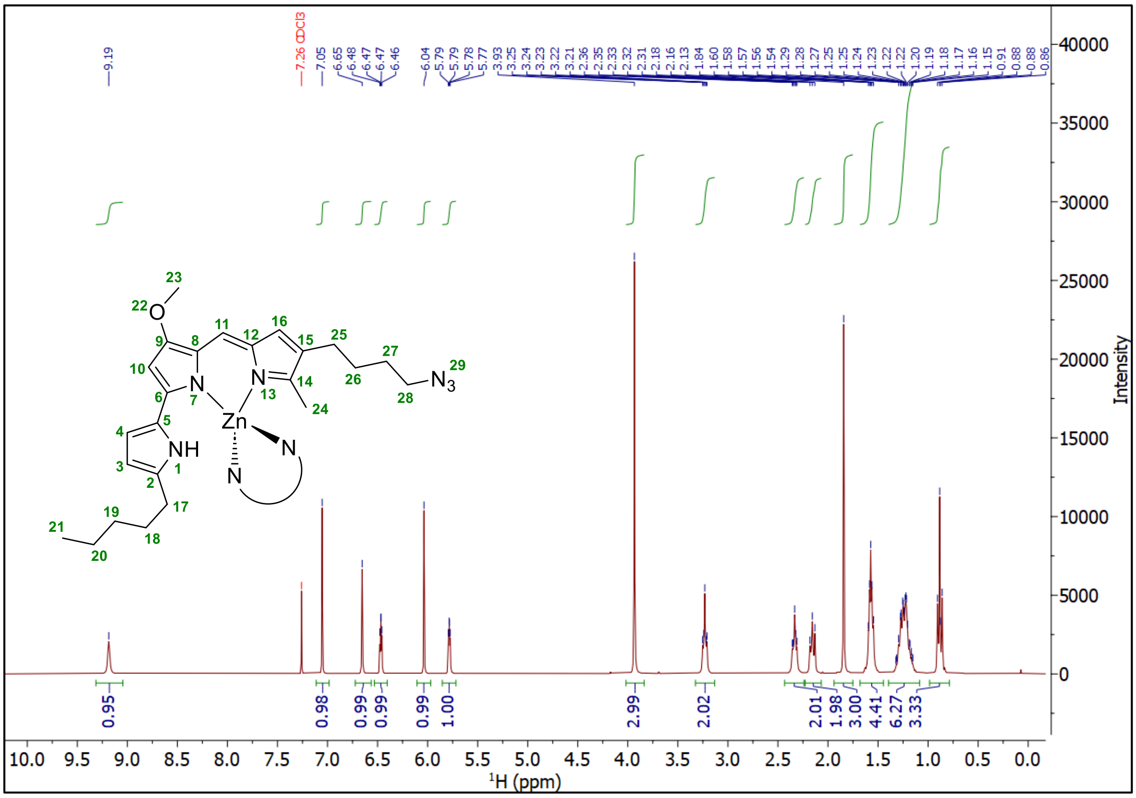

^{13}C NMR (76 MHz, $CDCl_3$)

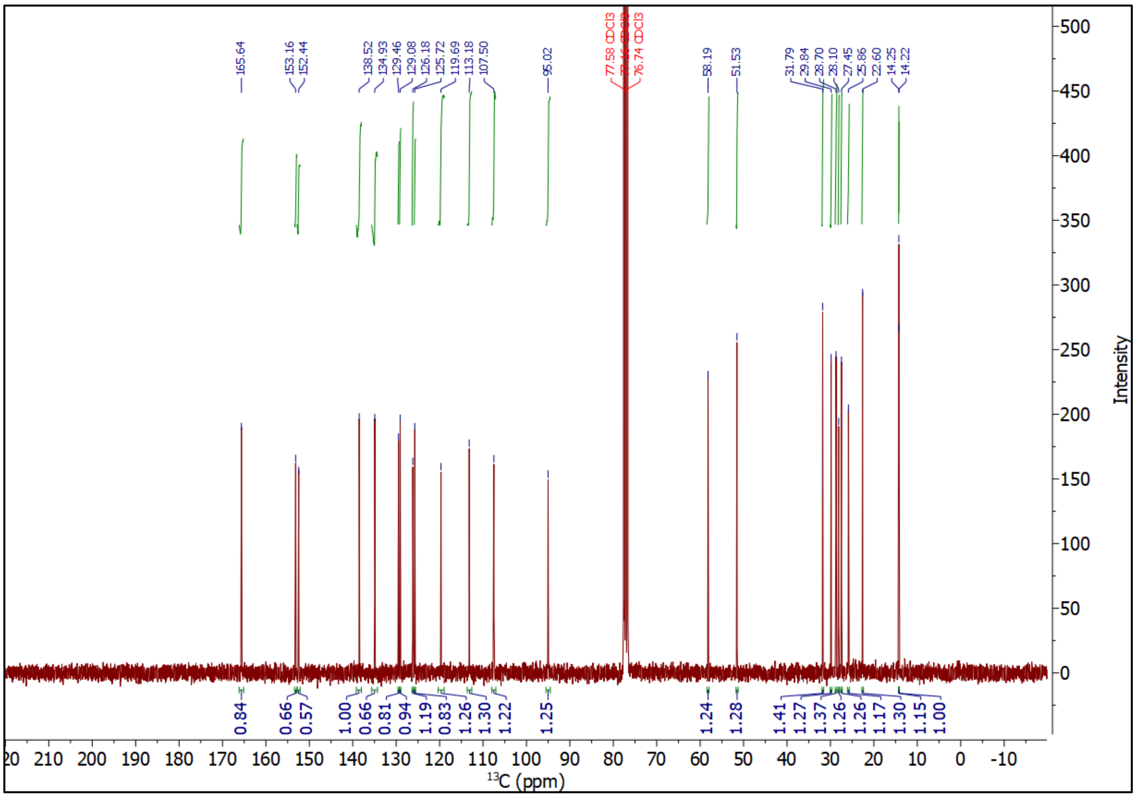

(*Z*)-5-(5-(4-(4-((2-(2,5-Dioxo-2,5-dihydro-1*H*-pyrrol-1-yl)ethyl)amino)-4-oxobutyl)-1*H*-1,2,3-triazol-1-yl)pentyl)-4'-methoxy-5'-((5-methyl-4-propyl-1*H*-pyrrol-2-yl)methylene)-1*H*,5'*H*-[2,2'-bipyrrol]-1'-ium chloride (46)

^{1}H NMR (300 MHz, CDCl$_3$)

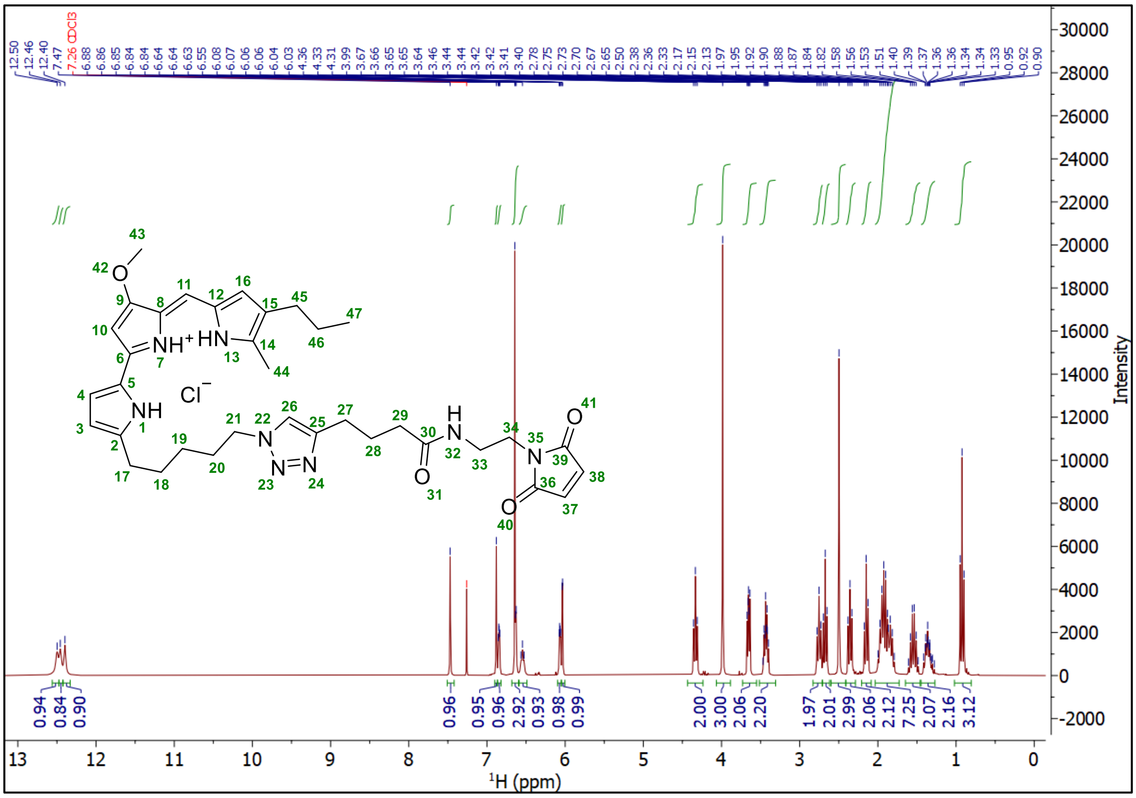

^{13}C NMR (76 MHz, CDCl$_3$)

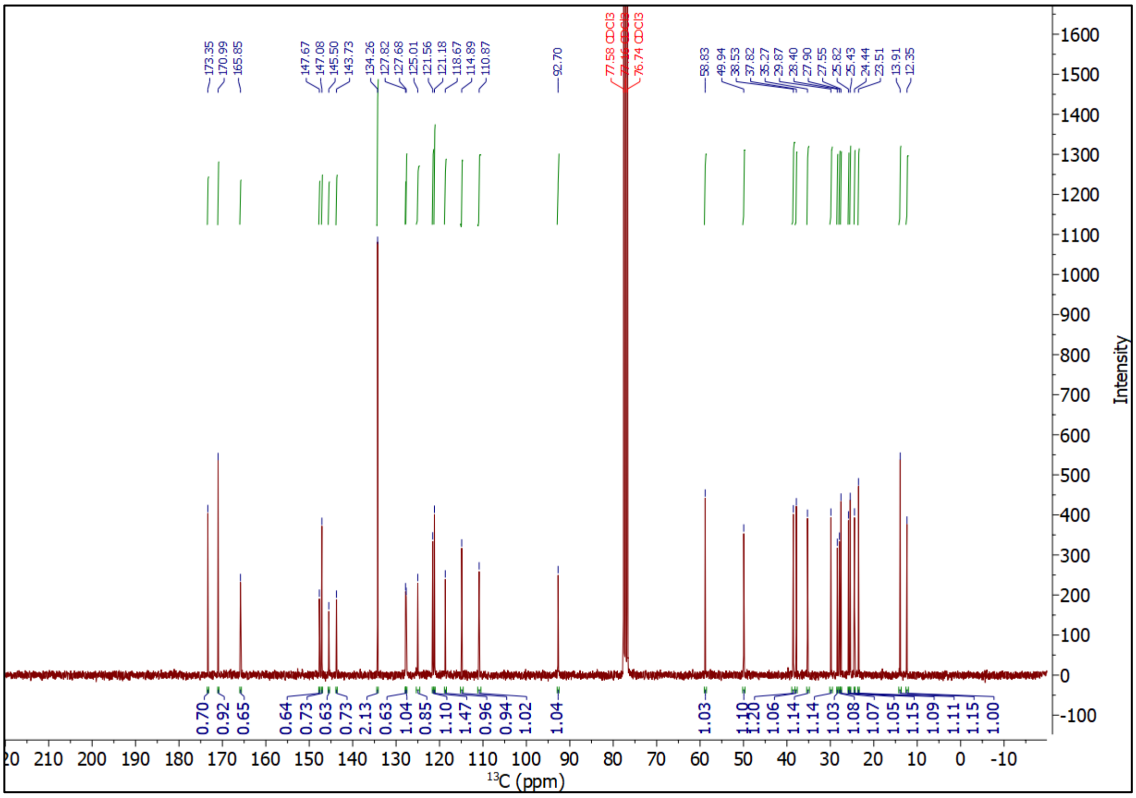

(*Z*)-4'-(4-(4-(4-((2-(2,5-Dioxo-2,5-dihydro-1*H*-pyrrol-1-yl)ethyl)amino)-4-oxobutyl)-1*H*-1,2,3-triazol-1-yl)butoxy)-5'-((5-methyl-4-propyl-1*H*-pyrrol-2-yl)methylene)-5-pentyl-1*H*,5'*H*-[2,2'-bipyrrol]-1'-ium chloride (47)

^{1}H NMR (300 MHz, $CDCl_3$)

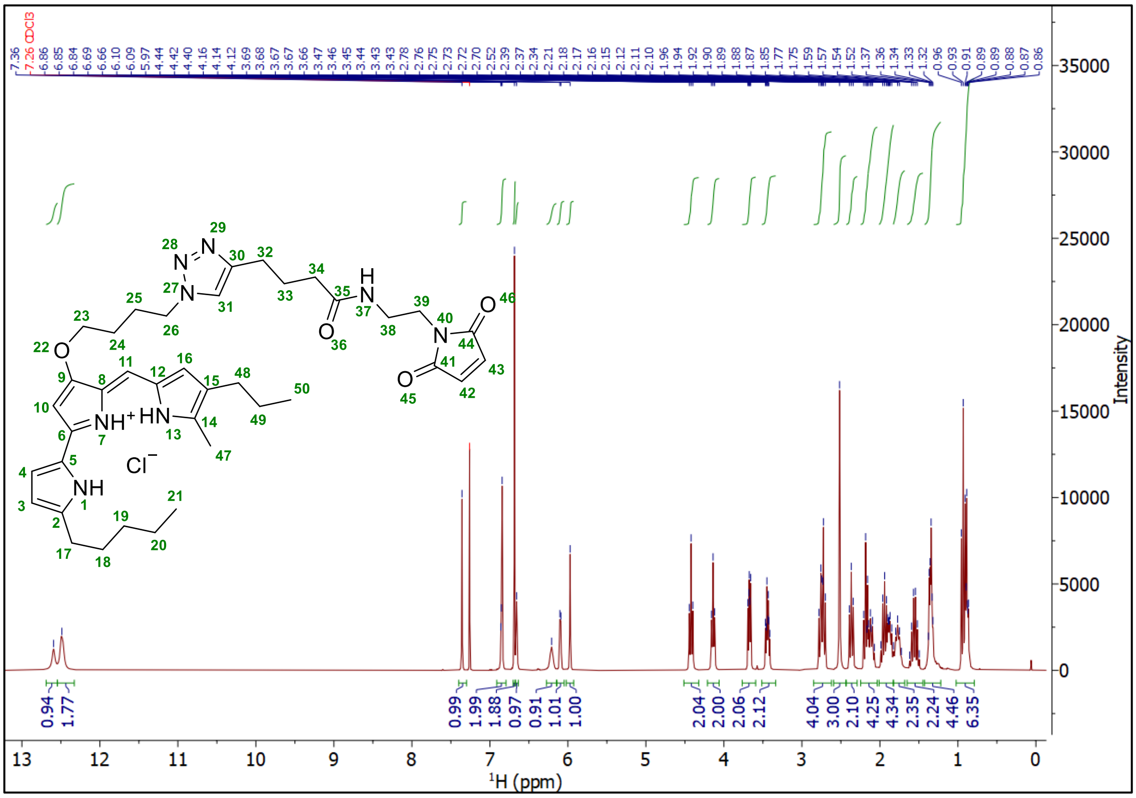

^{13}C NMR (76 MHz, $CDCl_3$)

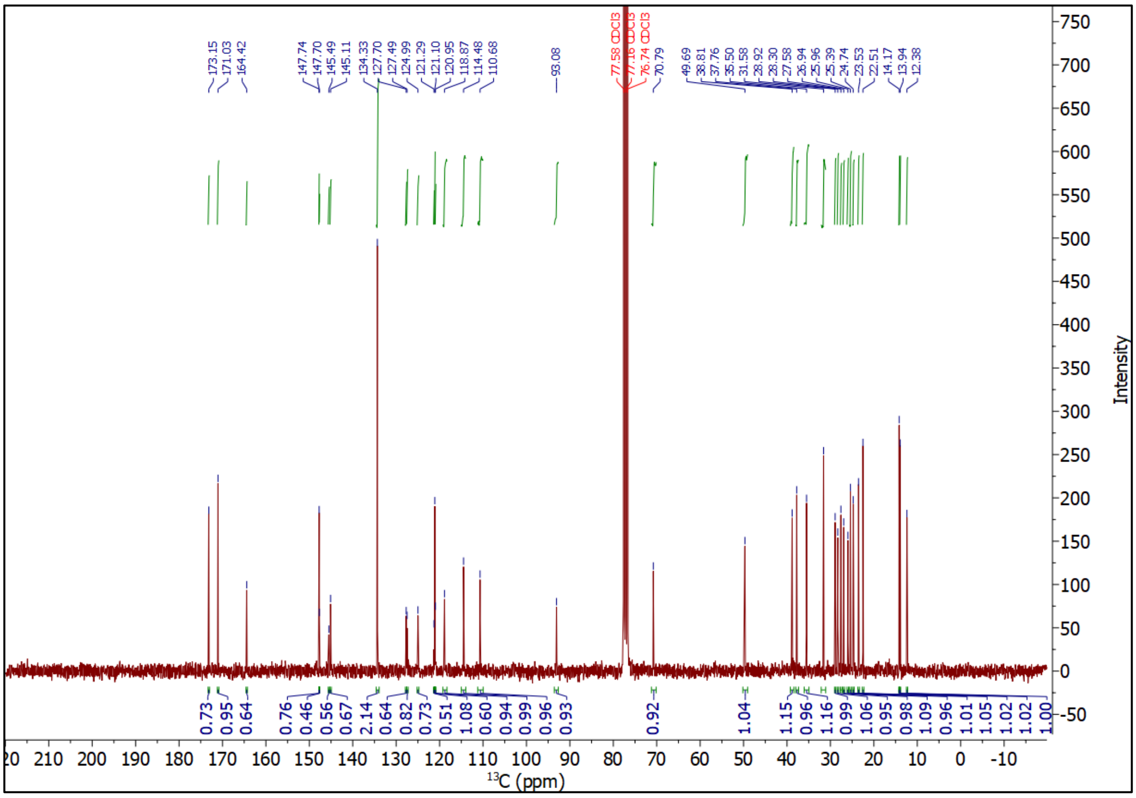

(*Z*)-5'-((4-(4-(4-(4-((2-(2,5-Dioxo-2,5-dihydro-1*H*-pyrrol-1-yl)ethyl)amino)-4-oxobutyl)-1*H*-1,2,3-triazol-1-yl)butyl)-5-methyl-1*H*-pyrrol-2-yl)methylene)-4'-methoxy-5-pentyl-1*H*,5'*H*-[2,2'-bipyrrol]-1'-ium chloride (48)

^{1}H NMR (300 MHz, $CDCl_3$)

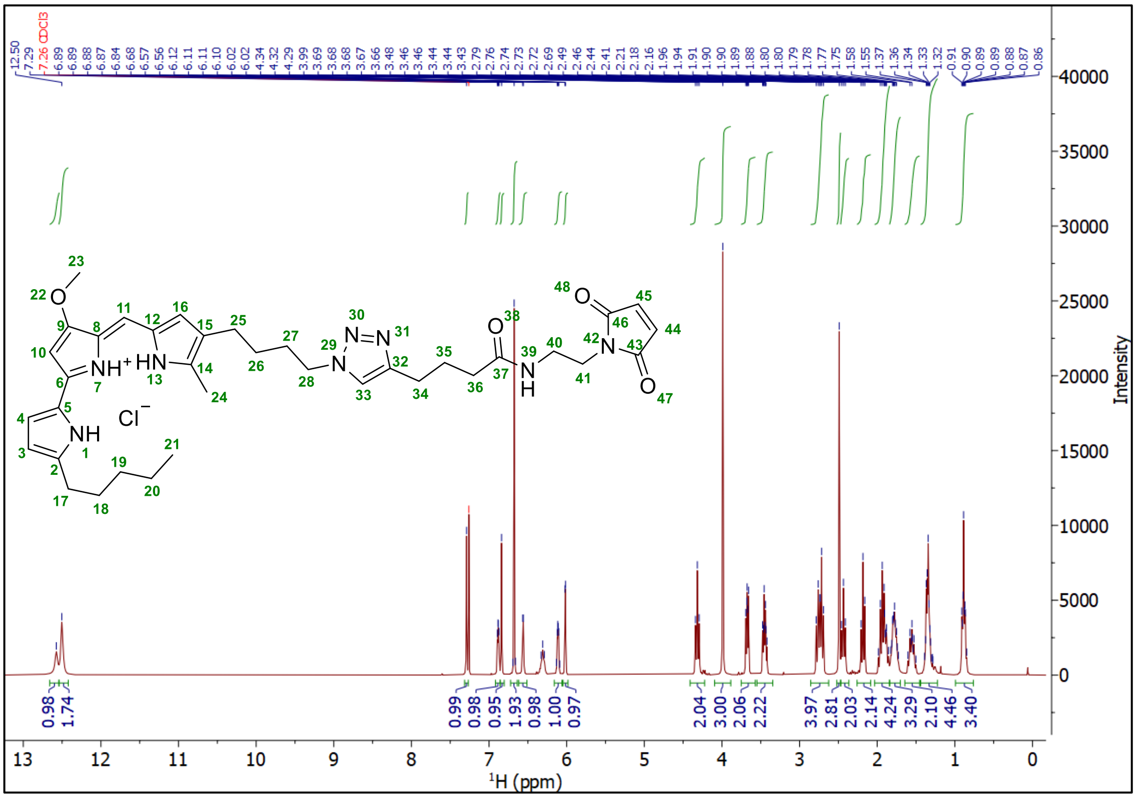

^{13}C NMR (76 MHz, $CDCl_3$)

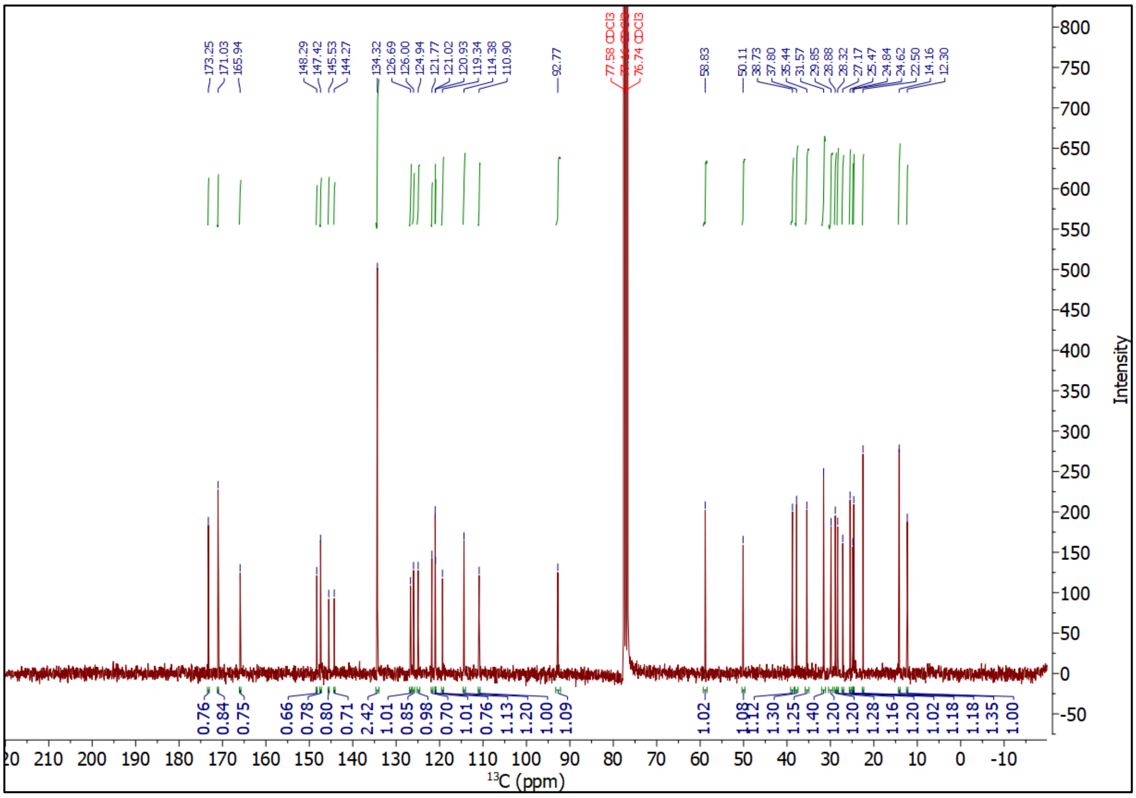

(*Z*)-5'-((5-Methyl-4-propyl-1*H*-pyrrol-2-yl)methylene)-4'-(4-(4-(15-oxo-19-((3a*S*,4*S*,6a*R*)-2-oxohexahydro-1*H*-thieno[3,4-*d*]imidazol-4-yl)-2,5,8,11-tetraoxa-14-azanonadecyl)-1*H*-1,2,3-triazol-1-yl)butoxy)-5-pentyl-1*H*,5'*H*-[2,2'-bipyrrol]-1'-ium chloride (50)

^{1}H NMR (600 MHz, $CDCl_3$)

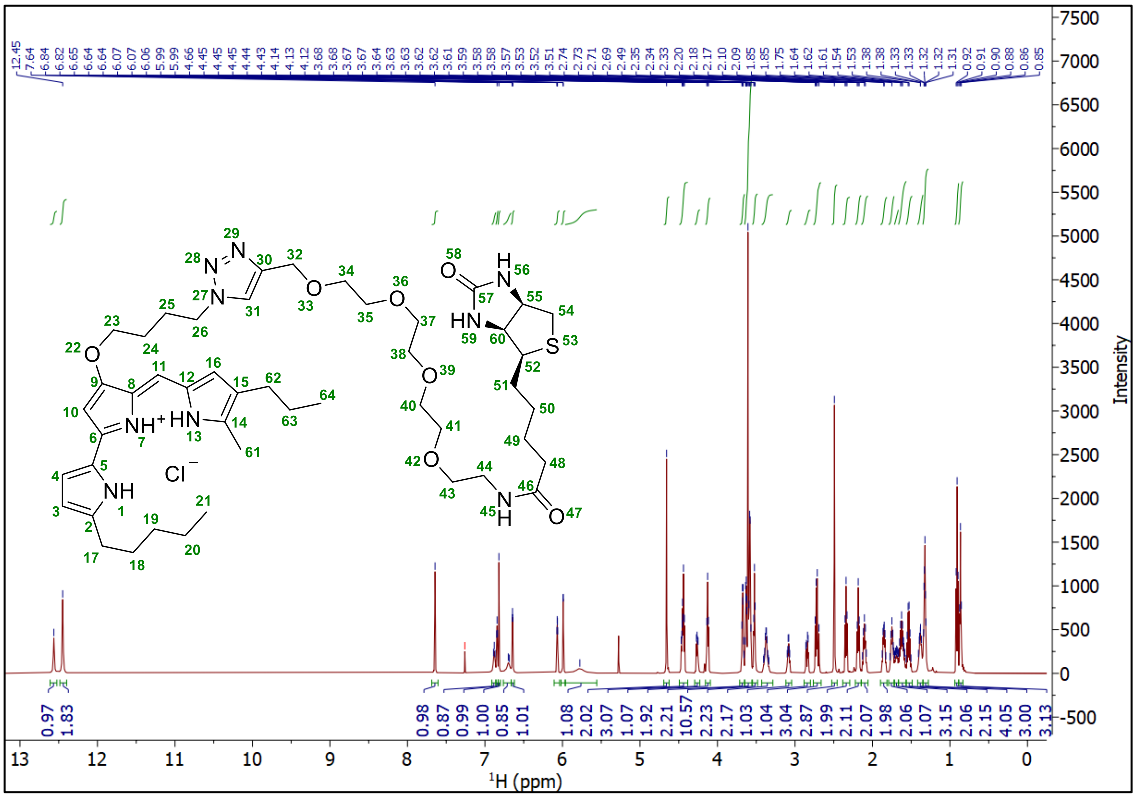

^{13}C NMR (151 MHz, $CDCl_3$)

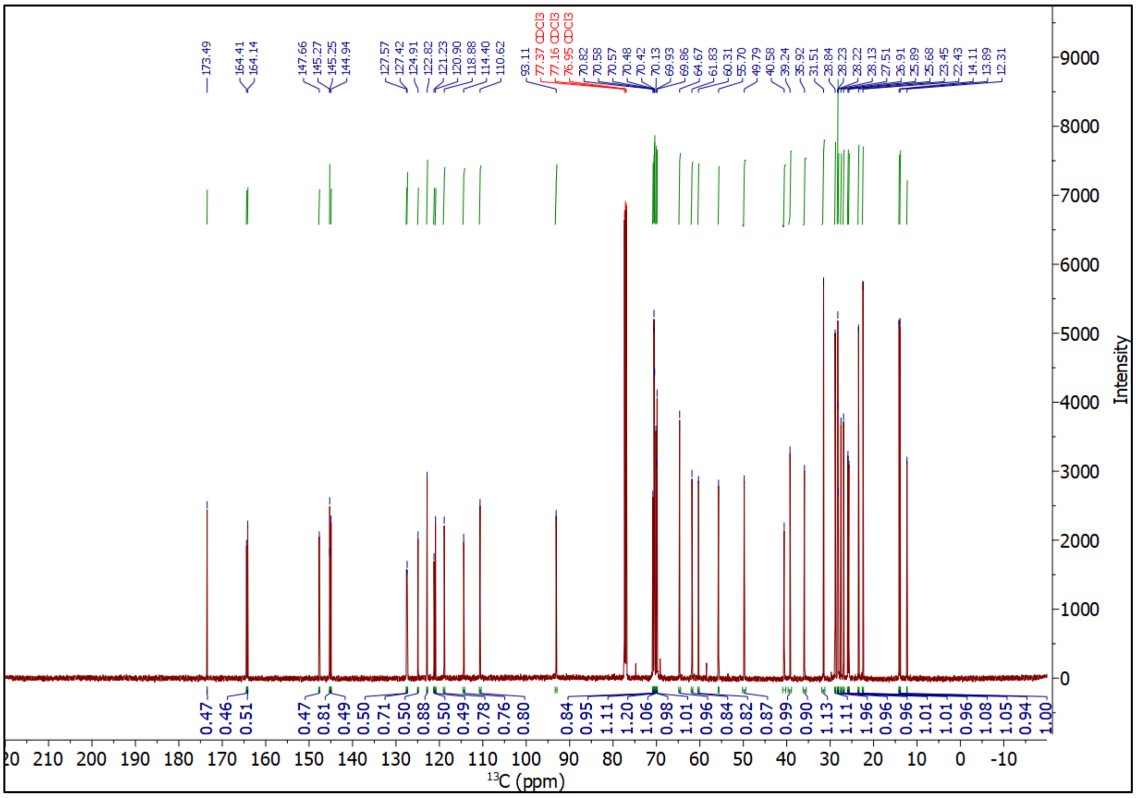

2-(Pent-4-en-1-yl)-1*H*-pyrrole (S2)

^{1}H NMR (600 MHz, $CDCl_3$)

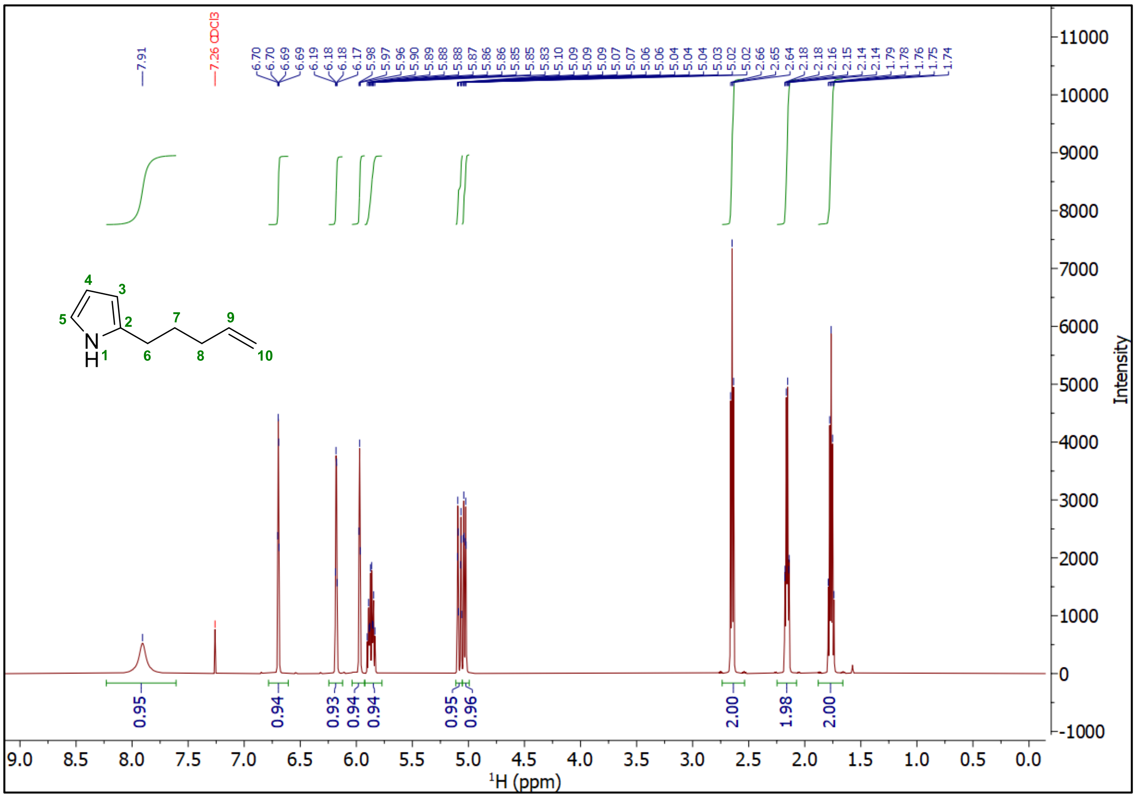

^{13}C NMR (151 MHz, $CDCl_3$)

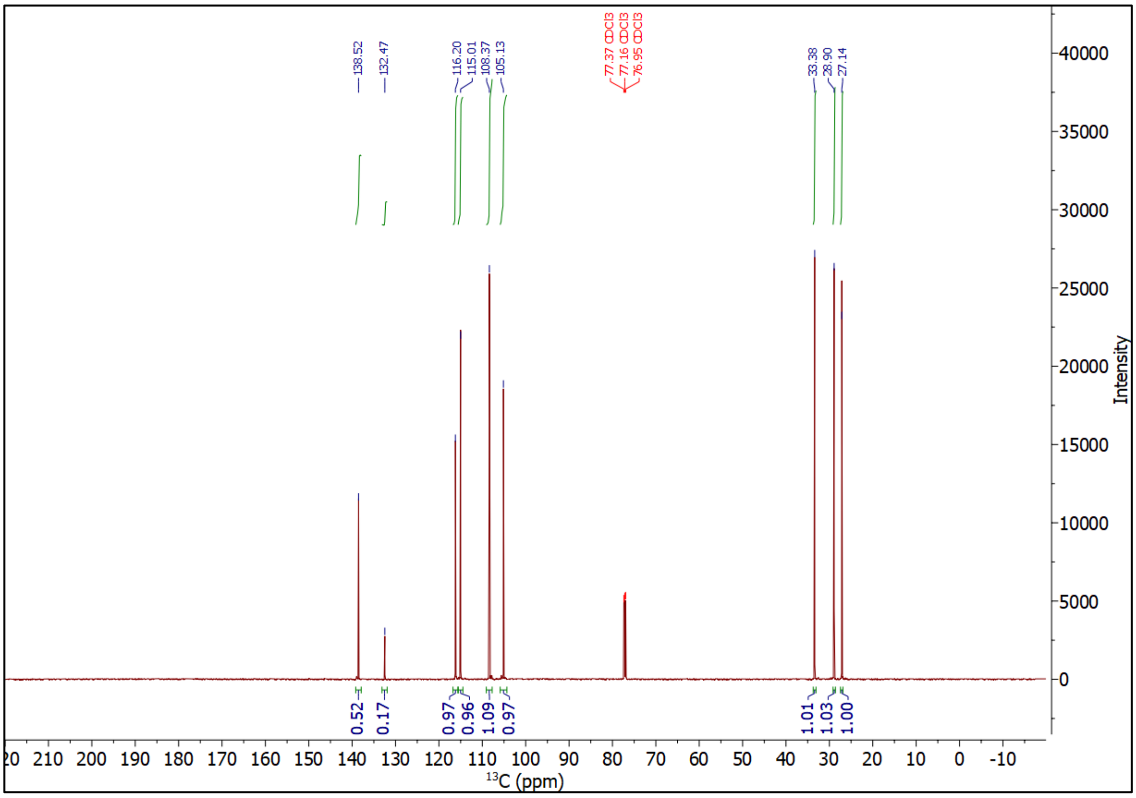

5-Chloro-1-(1*H*-pyrrol-2-yl)pentan-1-one (S3)

^{1}H NMR (600 MHz, $CDCl_3$)

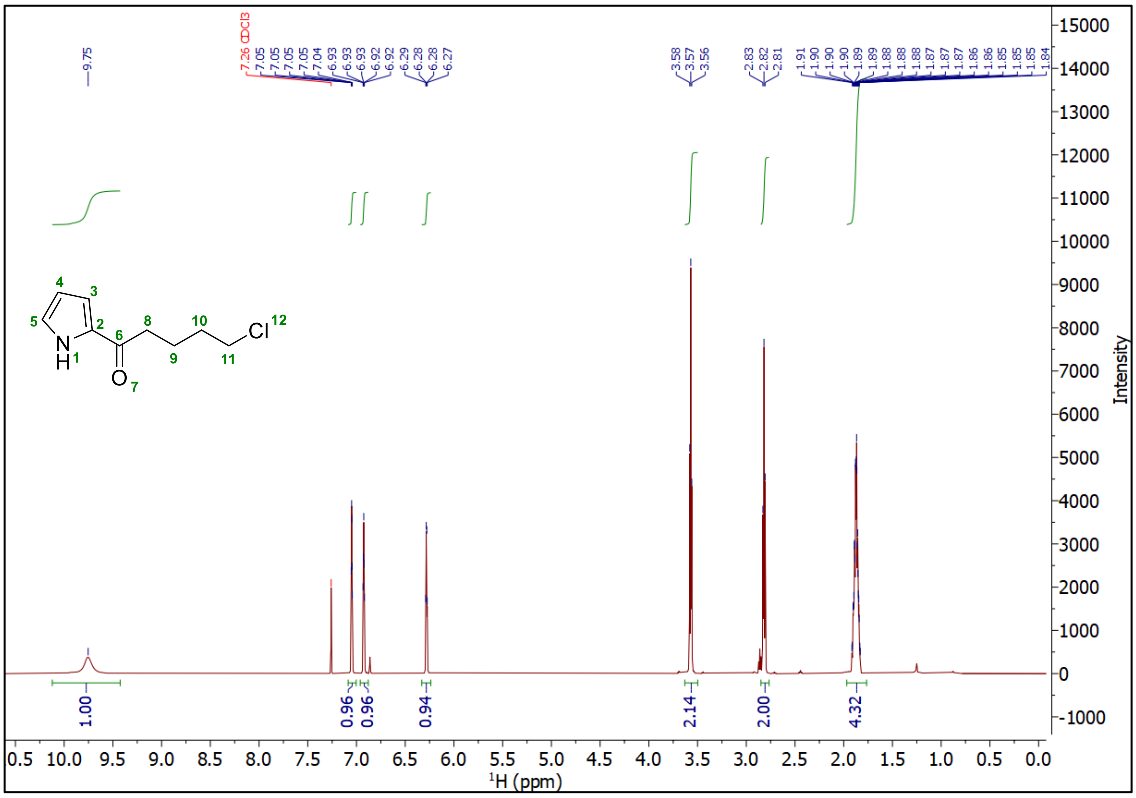

^{13}C NMR (151 MHz, $CDCl_3$)

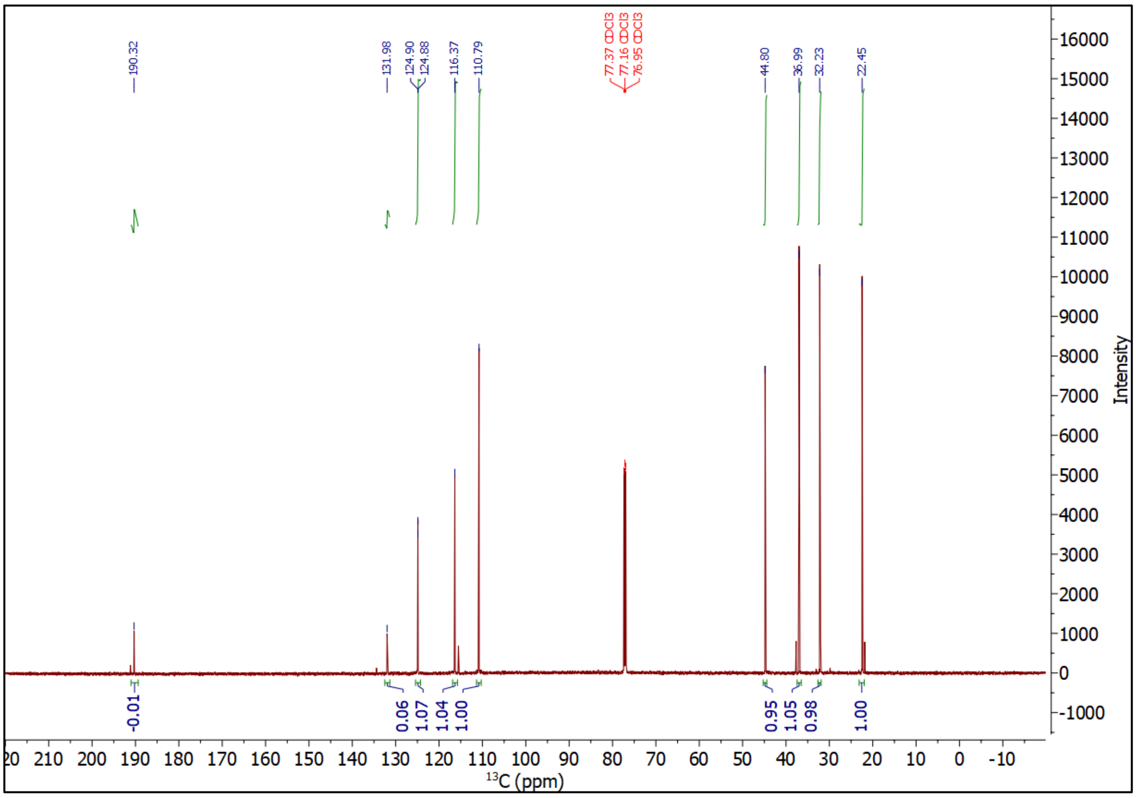

***tert*-Butyl 2-oxo-4-(tosyloxy)-2,5-dihydro-1*H*-pyrrole-1-carboxylate (S7)**

^{1}H NMR (600 MHz, CDCl$_3$)

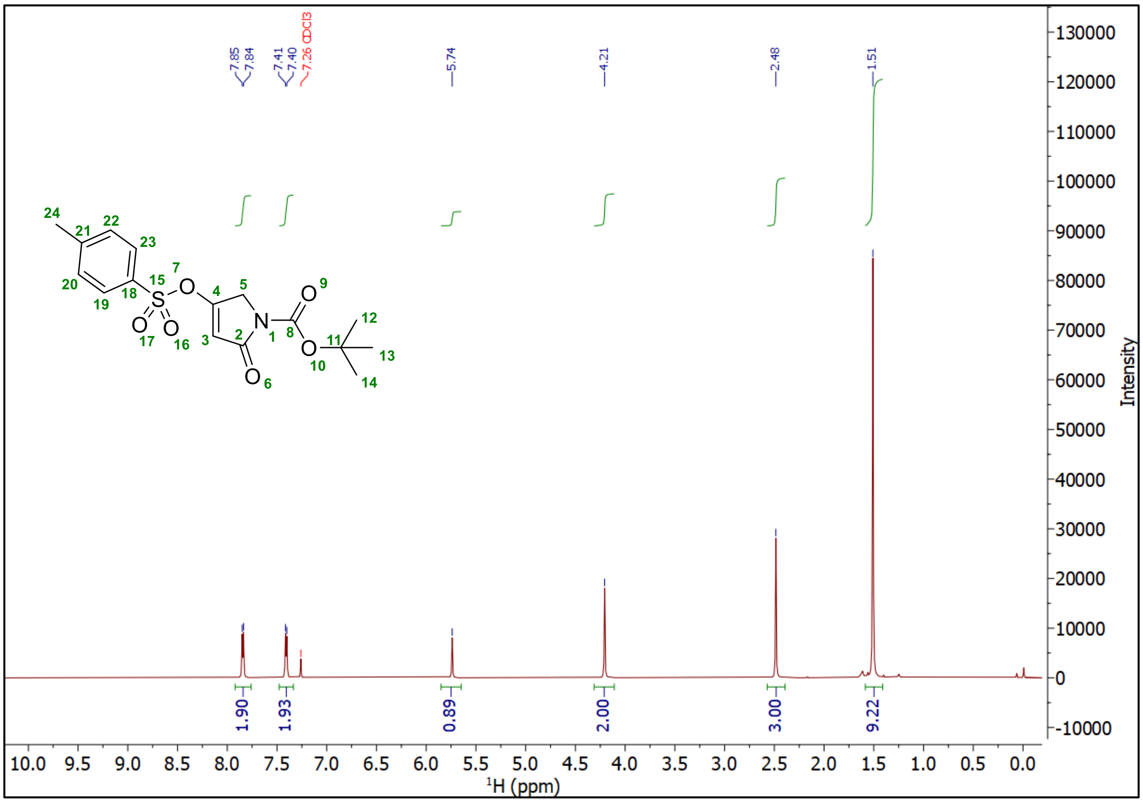

^{13}C NMR (151 MHz, CDCl$_3$)

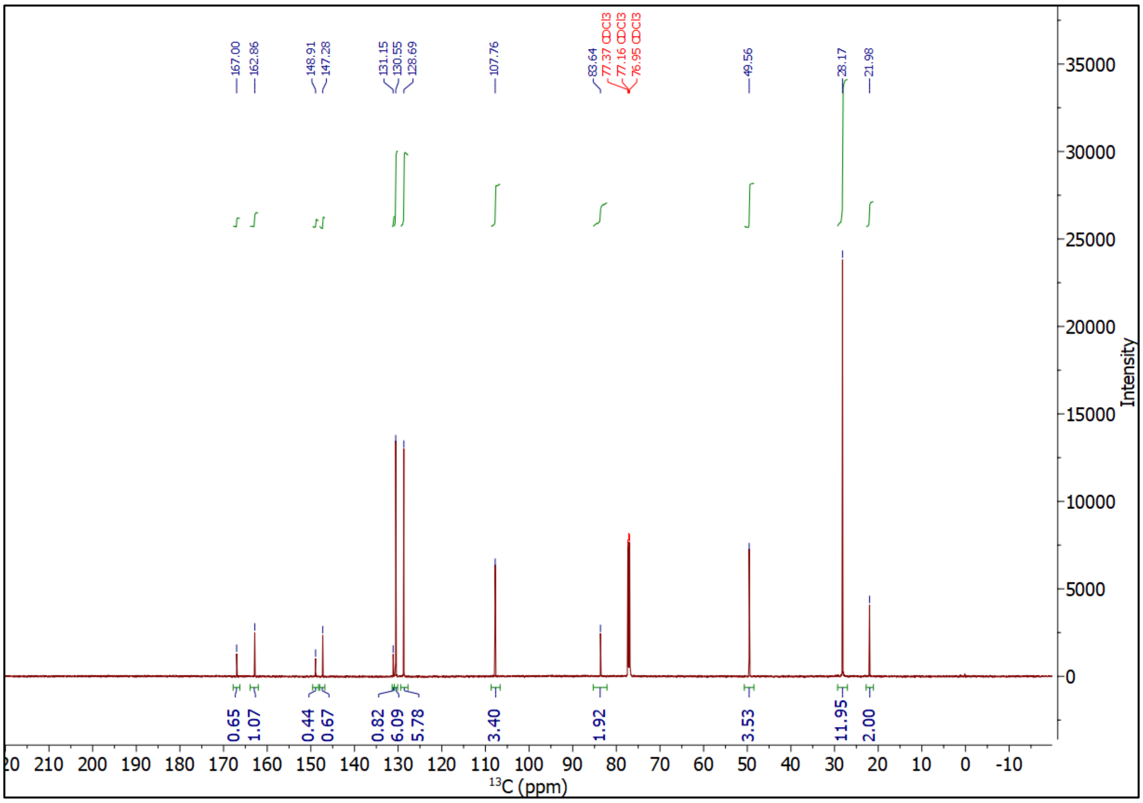

3-(But-3-en-1-yl)-2-methyl-1*H*-pyrrole (S9)

^{1}H NMR (600 MHz, $CDCl_3$)

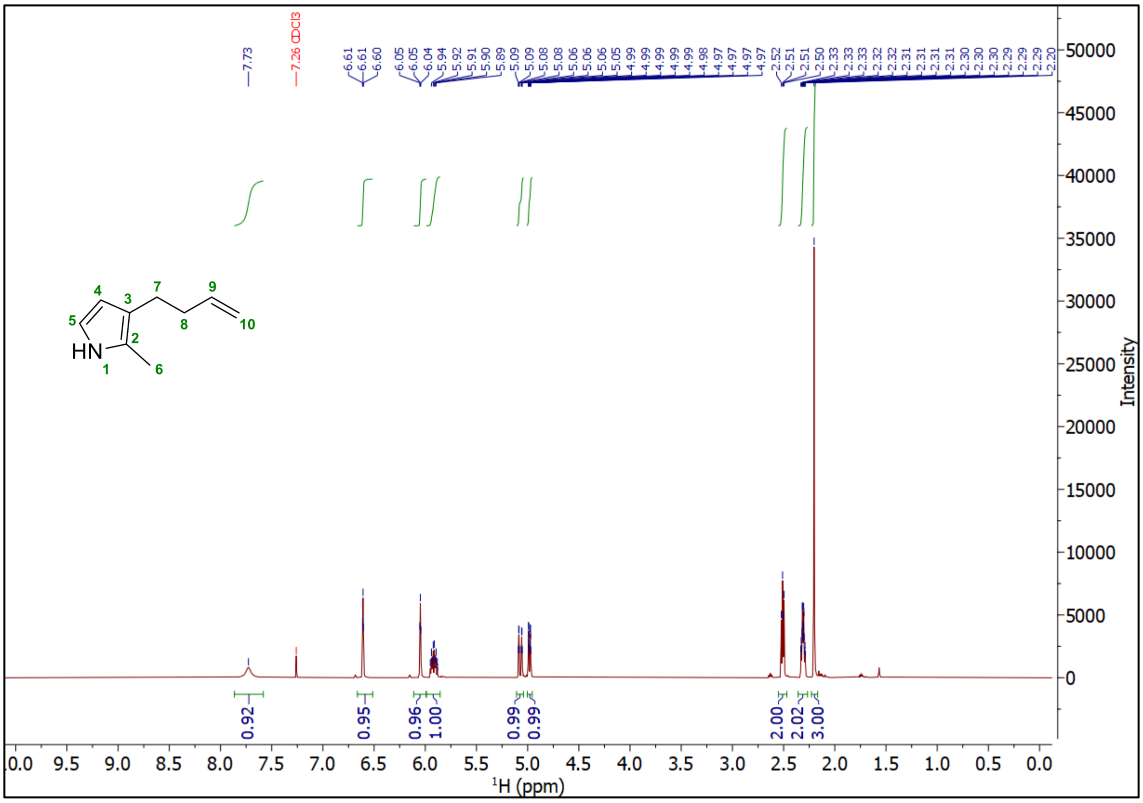

^{13}C NMR (151 MHz, $CDCl_3$)

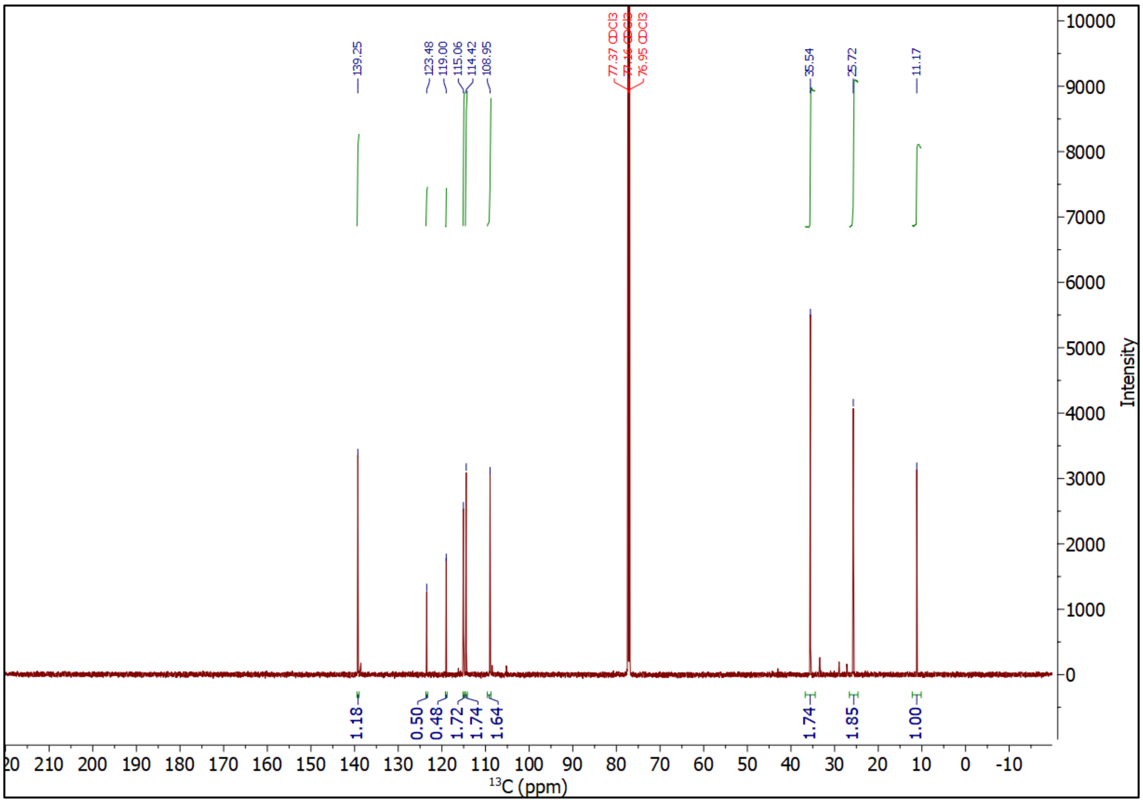

1-(1*H*-Pyrrol-2-yl)pent-4-en-1-one (S14)

^{1}H NMR (600 MHz, $CDCl_3$)

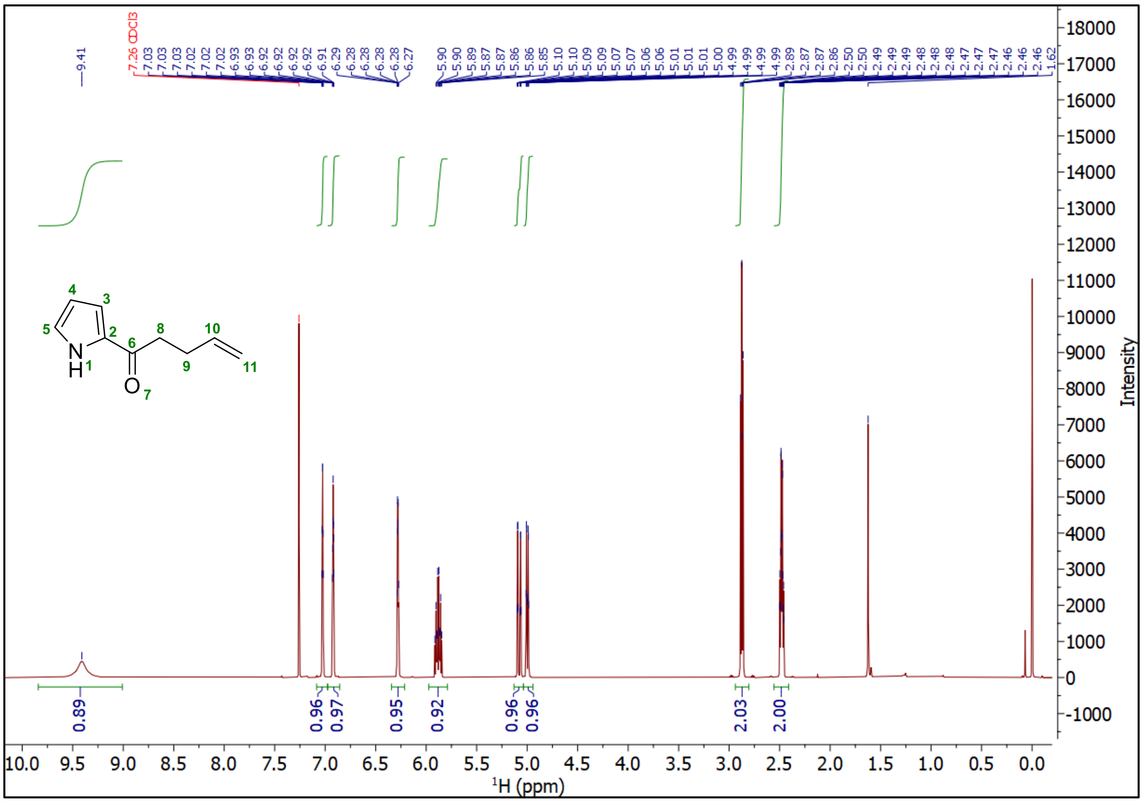

^{13}C NMR (151 MHz, $CDCl_3$)

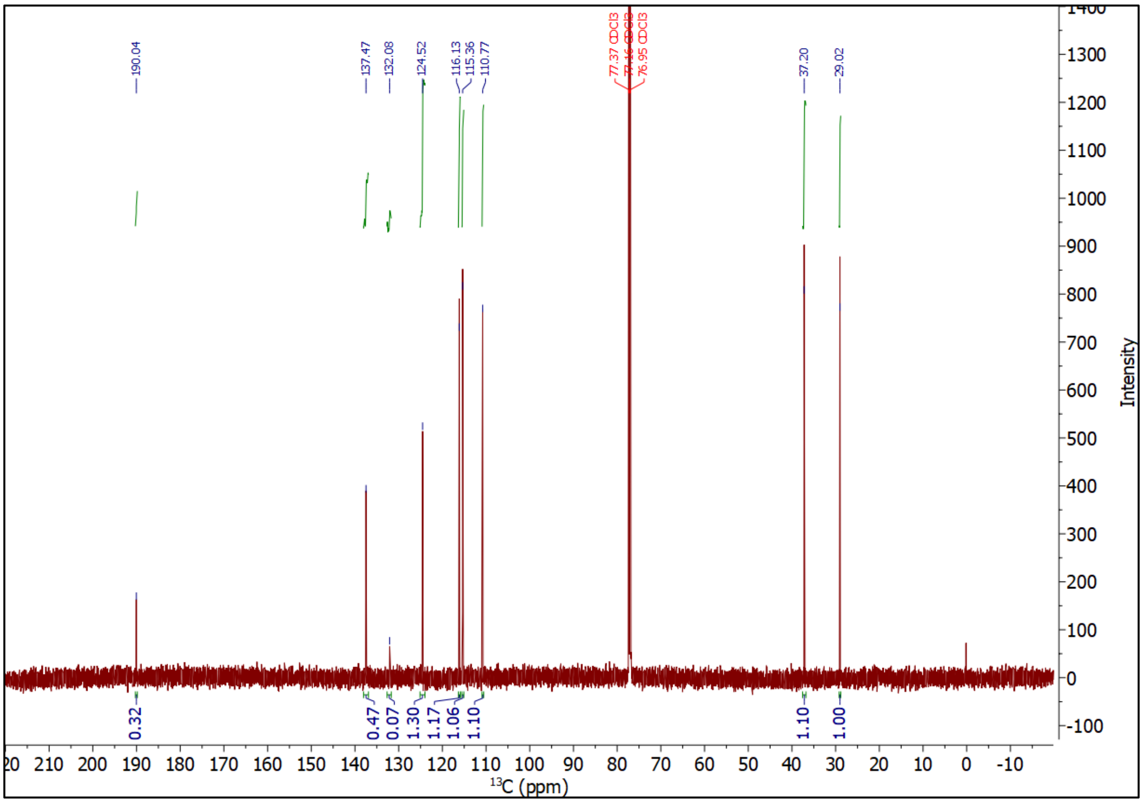

(*Z*)-4'-Methoxy-5'-((5-methyl-4-propyl-1*H*-pyrrol-2-yl)methylene)-1*H*,5'*H*-[2,2'-bipyrrol]-1'-ium chloride (S17)

^{1}H NMR (300 MHz, $CDCl_3$)

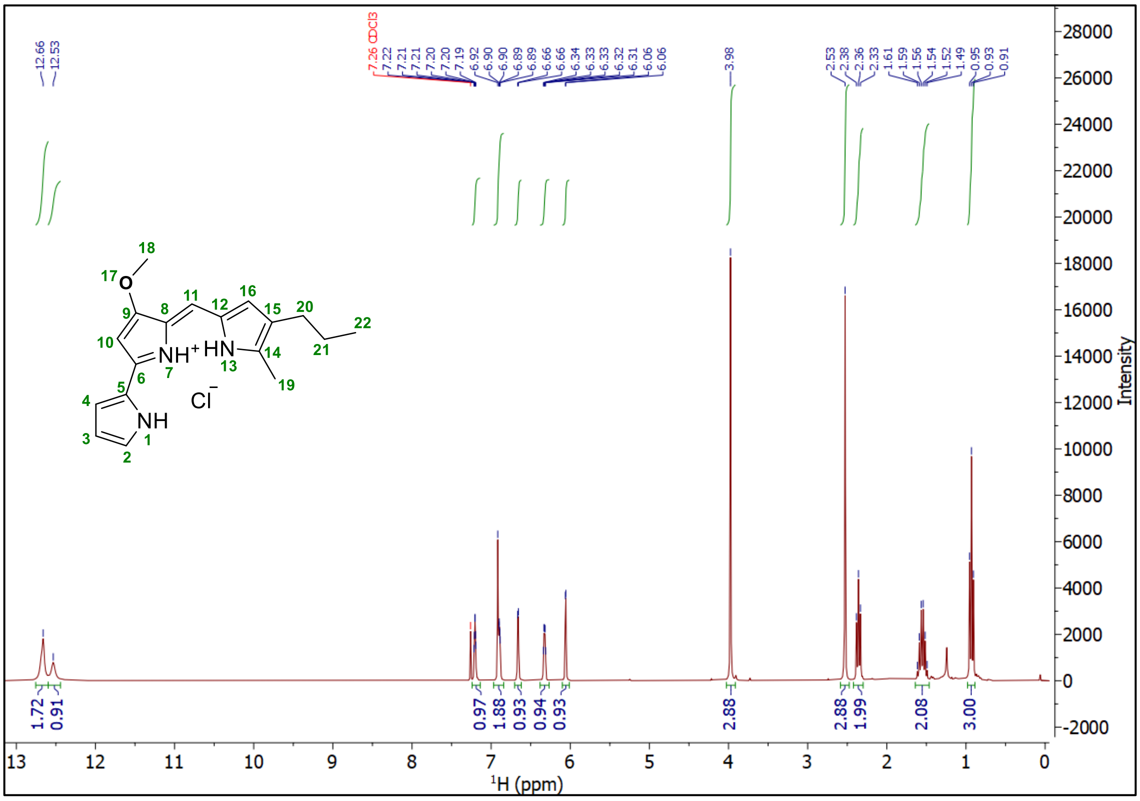

^{13}C NMR (76 MHz, $CDCl_3$)

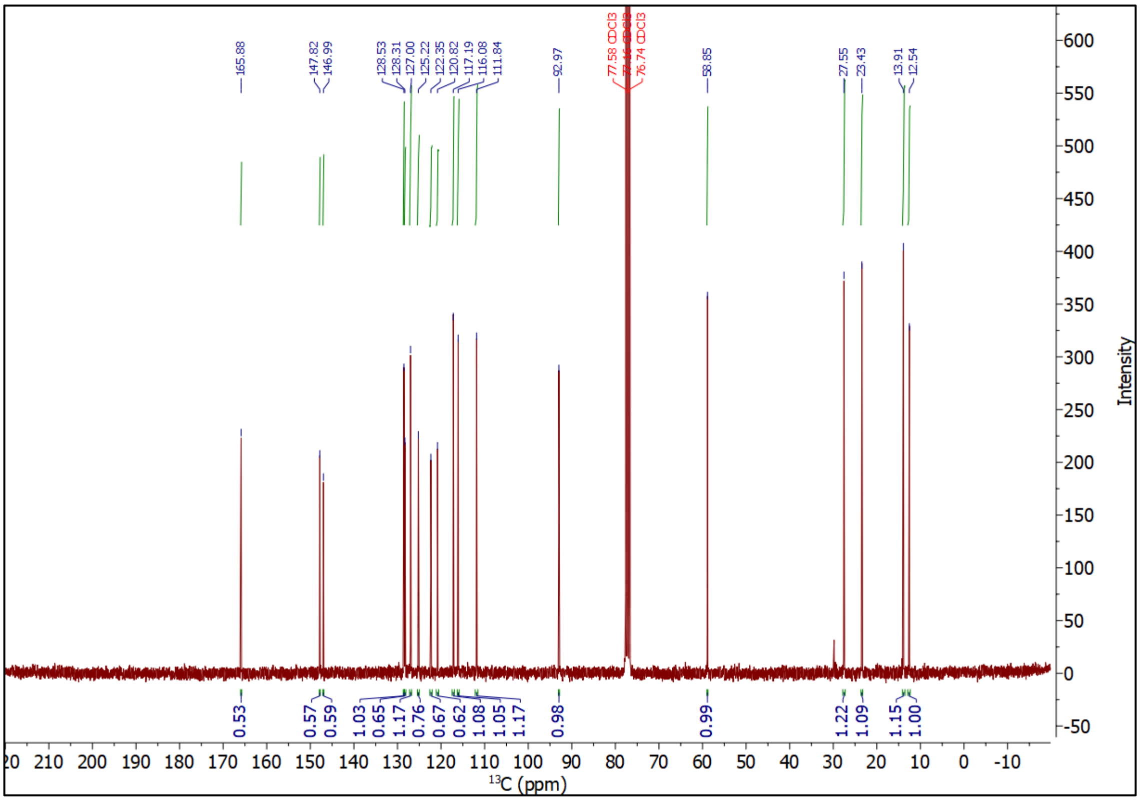

_N_-Methoxy-_N_-methylhex-5-enamide (S18)

^{1}H NMR (600 MHz, $CDCl_3$)

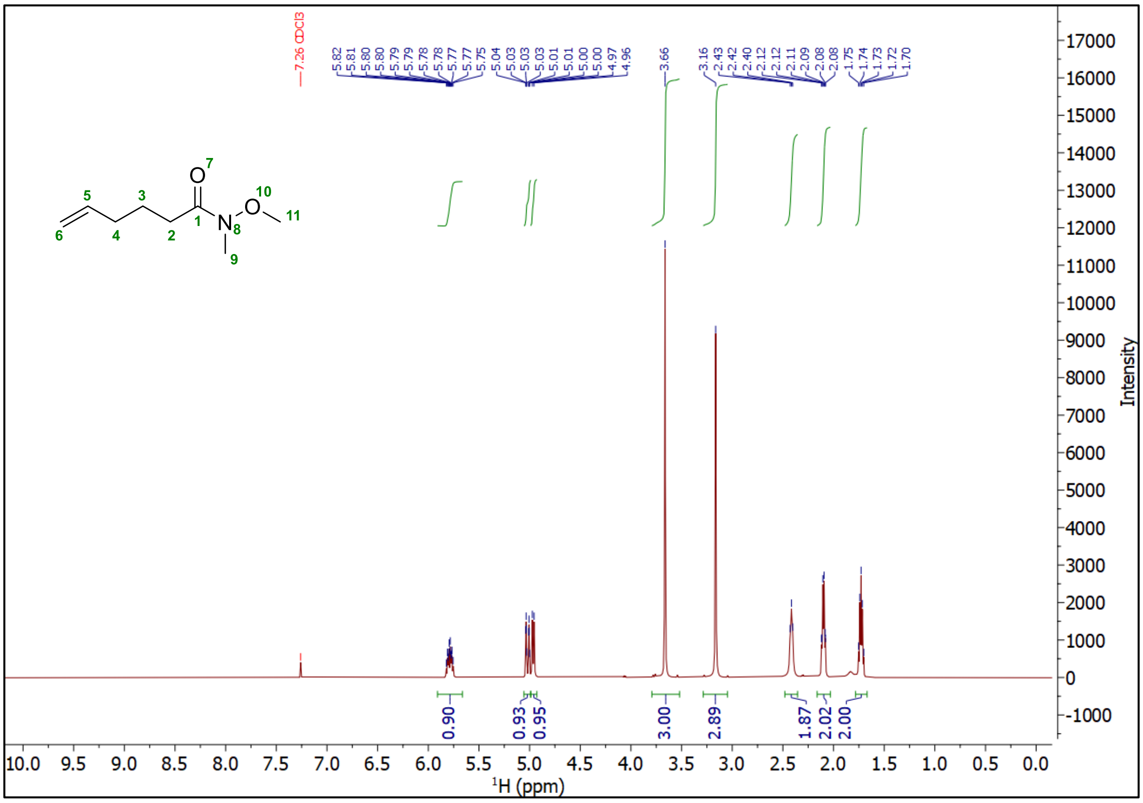

^{13}C NMR (151 MHz, $CDCl_3$)

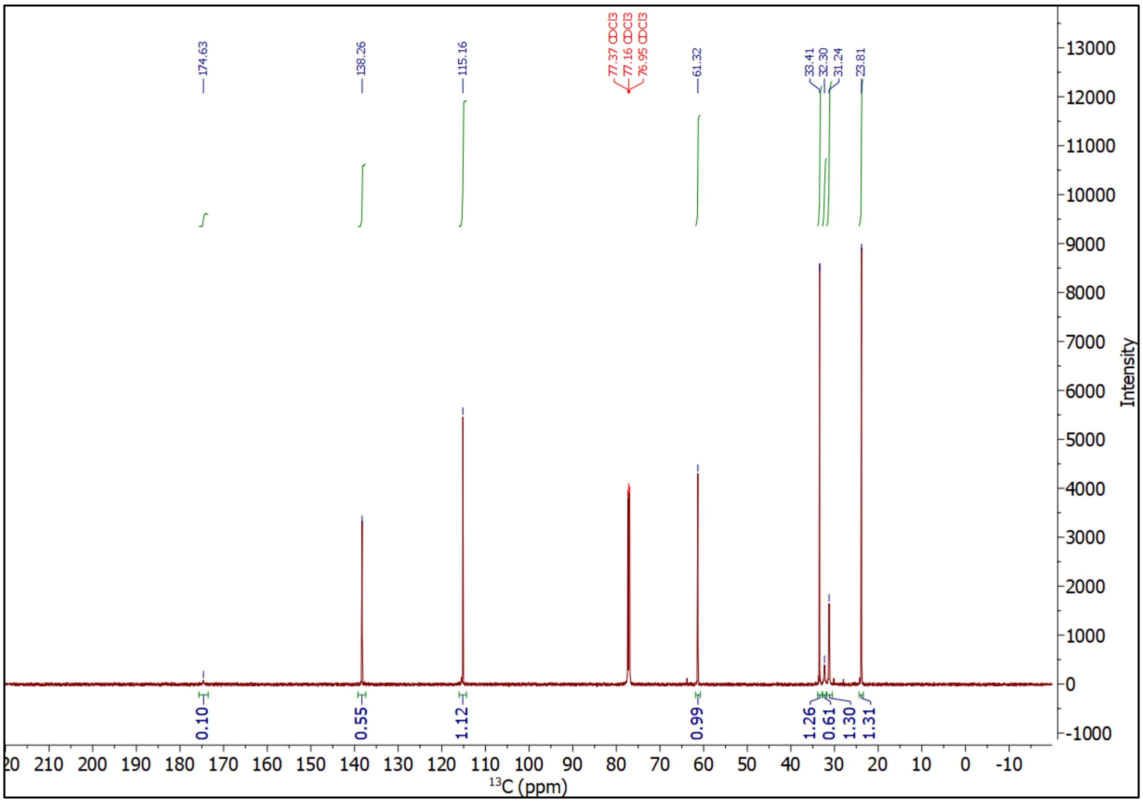

Hept-6-en-2-one (S19)

^{1}H NMR (600 MHz, $CDCl_3$)

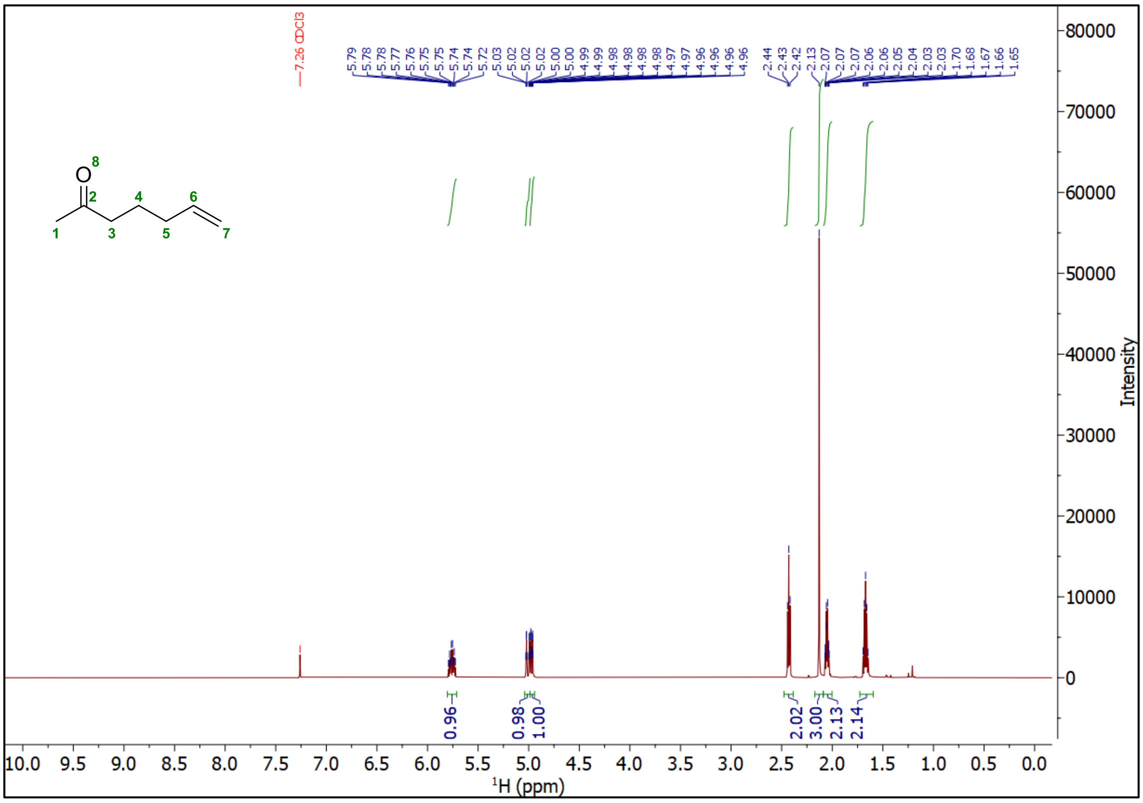

^{13}C NMR (151 MHz, $CDCl_3$)

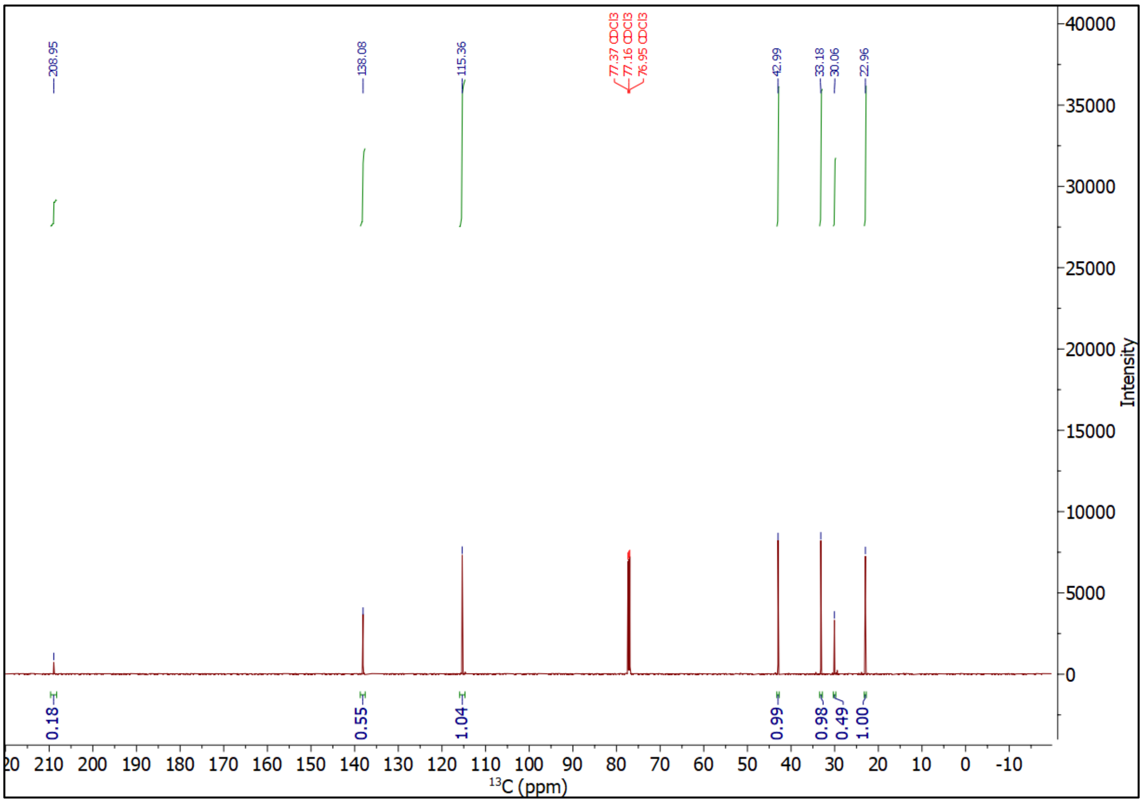

(*E*/*Z*)-Hept-6-en-2-one oxime (S20)

^{1}H NMR (600 MHz, $CDCl_3$)

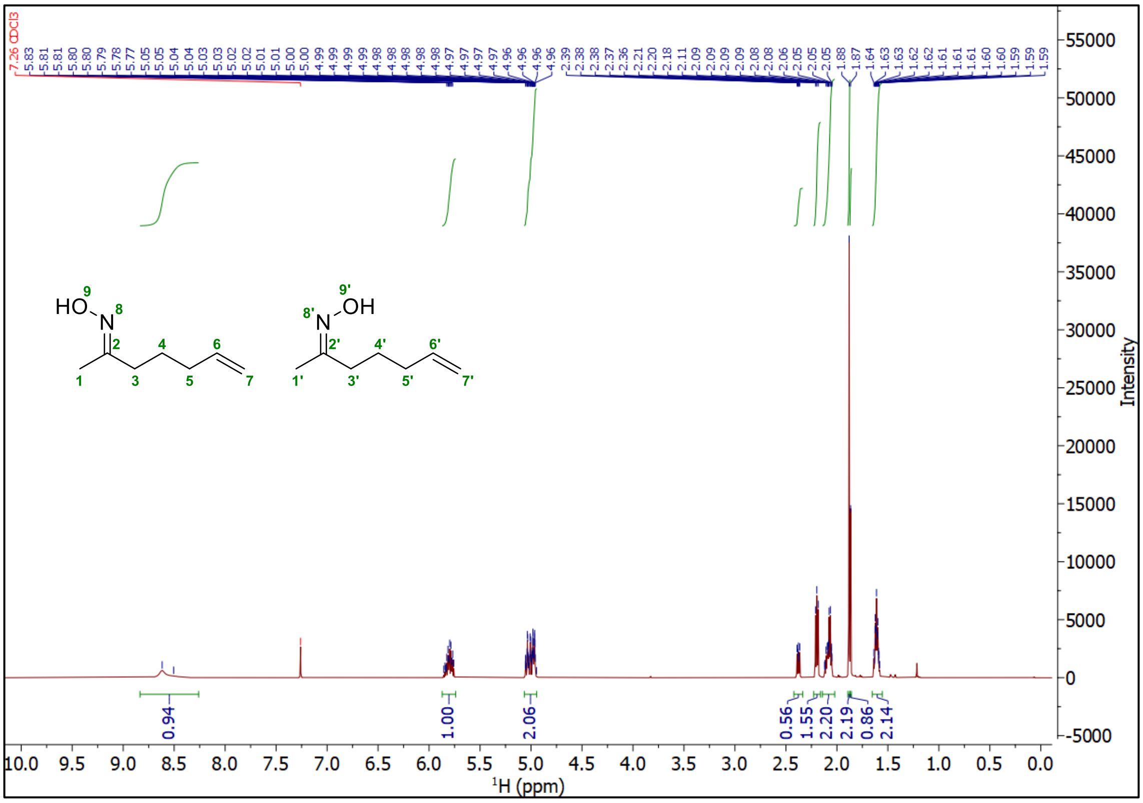

^{13}C NMR (151 MHz, $CDCl_3$)

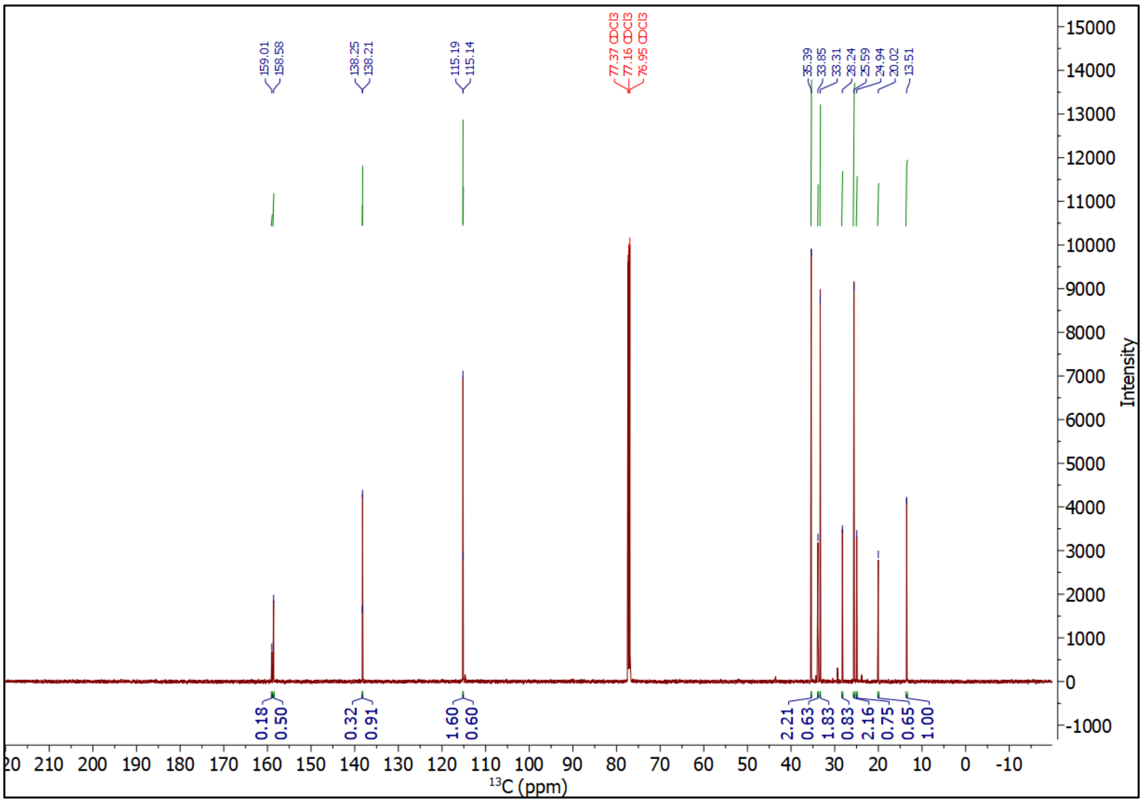

4-(2-Methyl-1*H*-pyrrol-3-yl)butan-1-ol (32) (from hydroboration-oxidation of alkene pyrrole **S9**)

^{1}H NMR (600 MHz, $CDCl_3$)

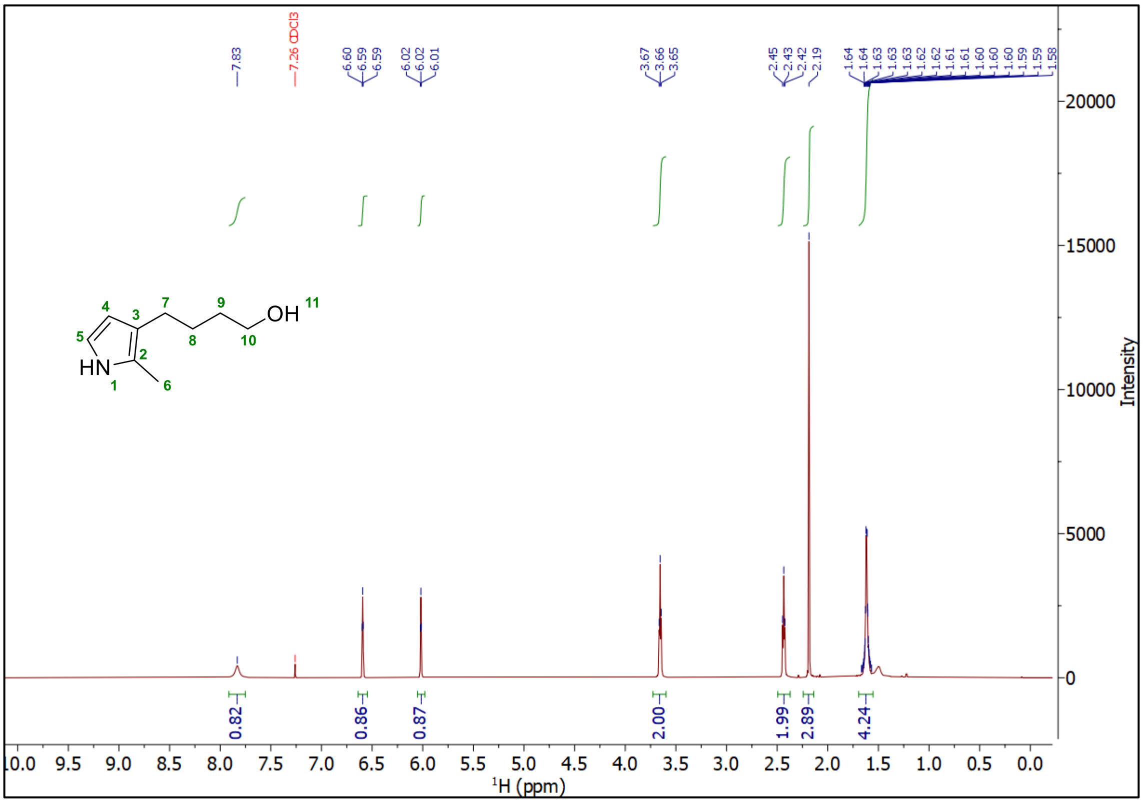

^{13}C NMR (151 MHz, $CDCl_3$)

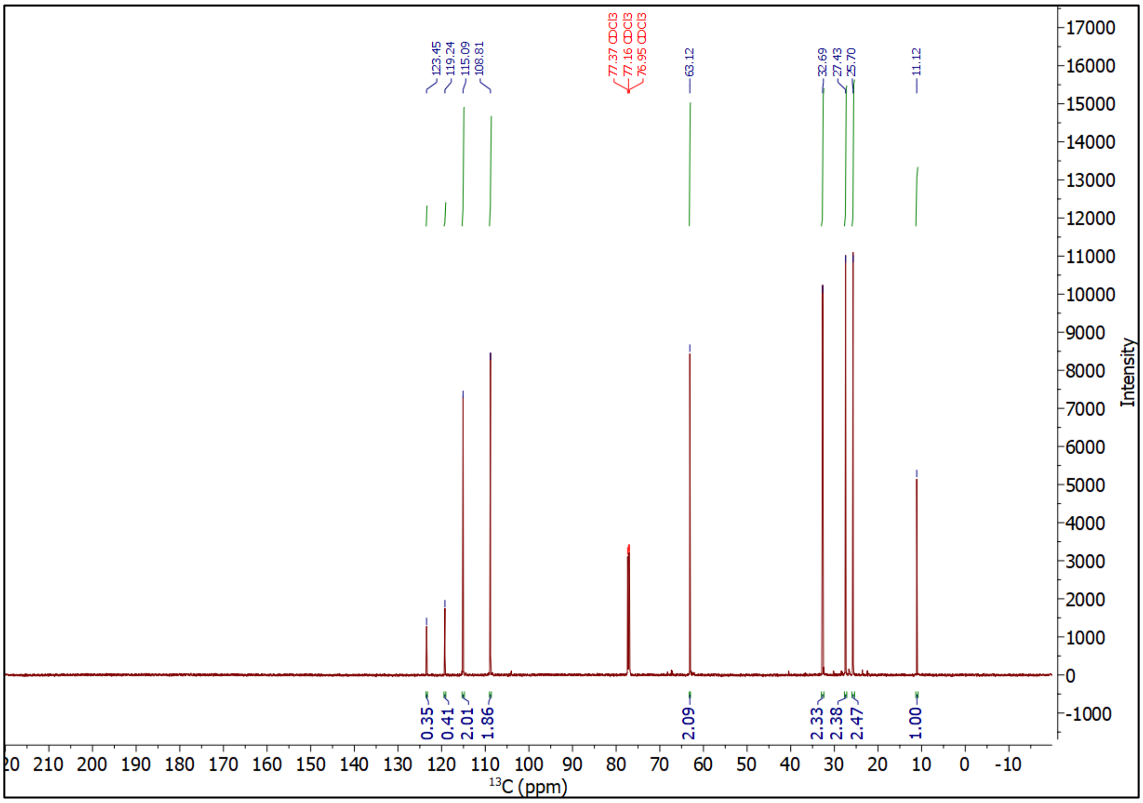

Supplement: Supplementary file 1 — Supporting Information [file CHEM-31-e202502066-s001.pdf]
